# Exploring the Pharmacokinetics of Drugs in Disabled Saudi Patients: A Systematic Review

**DOI:** 10.3390/ph18040582

**Published:** 2025-04-16

**Authors:** Faleh Alqahtani, Saeed A. Al Awadh, Muhammad Fawad Rasool

**Affiliations:** 1 Department of Pharmacology and Toxicology, College of Pharmacy, King Saud University, Riyadh 11451, Saudi Arabia; 2 Saudi Food and Drug Authority, Drug Sector, Riyadh 13312, Saudi Arabia; saeed19862@gmail.com; 3 King Salman Center for Disability Research, Riyadh 11614, Saudi Arabia; 4 Department of Pharmacy Practice, Faculty of Pharmacy, Bahauddin Zakariya University, Multan 60800, Pakistan

**Keywords:** absorption, disposition, drugs, clearance, cancer, epilepsy, Saudi Arabia

## Abstract

**Background/Objectives**: Disability is a term that involves mental, intellectual, or sensory impairment resulting in the loss of one’s ability to walk or perform the activities necessary to live in a society. This study aims to collect all the data regarding the absorption, distribution, and disposition of drugs in disabled Saudi patients, i.e., patients suffering from epilepsy, cancer, cardiovascular diseases, etc., and then compare these results with data reported in other ethnicities. **Methods**: An exhaustive online search used the key terms in Google Scholar, PubMed, Cochrane Library, and Science Direct to extract all articles that met the eligibility criteria. All research studies containing pharmacokinetic (PK) parameters (area under the curve from 0 to infinity (AUC_0–∞_), maximal plasma concentration (C_max_), clearance (CL), volume of distribution, time to reach maximum plasma concentration, and half-life) were included in this review. **Results**: In pediatric epileptic patients, carbamazepine showed a notable decrease in C_max_ with increasing age, which may be due to ontogenetic changes in its disposition. The AUC_0–∞_ of busulphan in adult hematopoietic stem cell transplantation patients was recorded as 4392.5 ± 1354.65 μg·h/mL, with high inter-individual variability. Moreover, the CL of vancomycin was reported to be 25% higher among cancer patients in comparison to non-cancer subjects. **Conclusions**: The complications in disabled patients due to alterations in cytochrome P450 enzymes, pathophysiology, genetics, and ethnicity emphasize the significance of patient-centered drug dosing. These findings may aid healthcare physicians in refining therapeutic care in this population.

## 1. Introduction

The World Health Organization (WHO) has defined disability in individuals as physical, mental, and cognitive impairments that affect their capabilities to follow routine activities and thus reduce their quality of life. About 1.3 billion people suffer from disability, which amounts to 16 percent of the global population. Moreover, the International Classification of Functioning, Disability, and Health (ICF) has explained disability as the restriction of participation and management of self-care activities, domestic and social life affairs, interactions with people, and acquiring and utilizing knowledge [1]. These disabilities include neurological (epilepsy, stroke, spinal cord injury), musculoskeletal (arthritis, muscle dystrophy), sensory (deafness, blindness), psychiatric (schizophrenia, bipolar disorder), and chronic health disorders (diabetes, cardiovascular disease, cancer, respiratory illness), etc. [2].

In terms of pharmacokinetics (PKs), renal and hepatic impairment, obesity, and organ transplantation also fall into disability categories. It is a broader term that describes the duration of drug uptake, distribution, and disposition [3]. Disabled patients have a higher rate of mortality than those with no disability across different patient populations (adults and pediatrics) [4]. These disabilities alter drug PKs, due to various physiological, pathological, and lifestyle variations [5]. Moreover, changes in PK parameters occur in these chronic diseases; therefore, comprehension of these scenarios may be beneficial for improving therapeutic care management and minimizing toxicity.

A person with a disability is defined according to Labor and Workman law in Saudi Arabia as ‘any person whose capacity to perform and maintain a suitable job has diminished as a result of a physical or mental infirmity’ [6]. In Saudi Arabia, genetic polymorphisms in different cytochrome P450 (CYP450) enzymes such as CYP2D6 [7], CYP2C9 [8], CYP2E1 [9], and CYP3A4 [10] have been recorded in previous papers, resulting in greater impacts on drug PKs in disabled patients. The metabolism of propranolol, valproic acid, carbamazepine, tacrolimus, and busulphan are influenced by CYP2D6, CYP2C9, CYP2E1, and CYP3A4 enzymes, respectively; thus, genetic variations in the latter may alter their safety and toxicity. Among these drugs, carbamazepine is a strong inducer [11], whereas valproic acid is an inhibitor drug [12] of different enzymes, which may affect the PK parameters such as maximal plasma concentration (C_max_), and clearance (CL), resulting in the need for dosage adjustments in drug–drug interactions. Moreover, inter-individual and inter-ethnic differences in Arabs change the PKs of various medications metabolized by enzymes with different allele expressions [7]. Disabilities, along with chronic diseases, continue to prevail, which poses a significant problem for healthcare providers in delivering individualized dosage regimens; therefore, pooling evidence-based information on variations in the PKs of drugs serves a vital role in tailoring drug doses.

In the context of the occurrence of drastic changes in PKs among individuals with disabilities, and due to limited data on disabled persons in Saudi Arabia in the previously published literature, there is a need to explore the various aspects of absorption, distribution, metabolism, and excretion (ADME) of drugs in this population. Furthermore, no systematic review has been published from this perspective. Therefore, the objective of this study is to collate all the data on drug PKs in disabled patients, focusing on the Saudi Arabian population. This is followed by a detailed comparison of these data with other similar studies presented for subjects of different countries in consideration of drug dosing optimization.

## 2. Materials and Methods

### 2.1. Study Protocol and Screening Strategy for Literature Review

**Figure 1:**
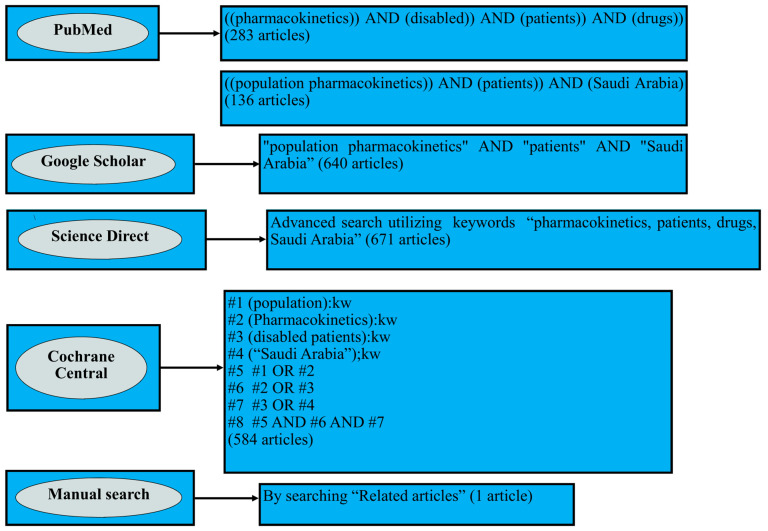
Strategy for extensive literature search.

The online databases Google Scholar, PubMed, Science Direct, and Cochrane Central Register of Controlled Trials were utilized to search for research articles on the PKs of various drugs in critically ill patients focused on the Saudi Arabian population until 10 November 2024. This systematic review followed the Preferred Reporting Items for Systematic Reviews and Meta-analysis (PRISMA) guidelines [13]. Different terms such as ‘population pharmacokinetics’, ‘pharmacokinetics’, ‘disabled’, ‘patients’, ‘drugs’, and ‘Saudi Arabia’ were utilized to identify the relevant articles, and two independent researchers reviewed this process. The details of this exhaustive literature search are presented in Figure 1.

### 2.2. Eligibility Criteria

All the literature search results from four extensive databases were then exported to EndNote version 20. The duplicate articles were screened using the option ‘Find duplicates’ and subsequently removed. Articles were included based on the following criteria: the targeted population was disabled patients; studies were conducted on individuals in Saudi Arabia; and at least one PK parameter, i.e., area under the concentration–time curve from 0 to infinity (AUC_0–∞_), CL, volume of distribution (V_d_), C_max_, half-life (T_1/2_), and time to reach maximal plasma concentration (T_max_) for drugs, was recorded. Moreover, clinical research studies on population PKs were also included. The articles were excluded if they were irrelevant in terms of title, abstract, and language, and if they were carried out on animals. Moreover, reviews, books, short commentaries, and letters to editors were also excluded. Following further review based on full-text reading, a remaining 9 articles were included in this review. The details of the exclusions are presented in Supplementary Table S1.

### 2.3. Data Extraction:

The following data were obtained from the studies: author name, population characteristics (adults or pediatrics), age, weight, gender, disabled condition, number of participants, drug, analytical method, and dosage regimen (dose, route, and frequency). The unit of AUC_0–inf_ was standardized to facilitate readability regarding uniformity in the data.

### 2.4. Evaluation of the Quality of the Included Articles

The quality of all the relevant clinical research studies was evaluated by the Jadad tool first, which was originally utilized in the case of clinical trials, but previous systematic reviews have mentioned it in the context of PK studies; therefore, this tool is included here as well. The scoring was based on randomization, blinding of individuals, and dropouts/withdrawals. The studies were assigned scores of >4, 3–4, or <3, corresponding to high, fair to moderate, or low quality, respectively [14]. Secondly, the Critical Appraisal Skills Programme (CASP) tool was employed to assess the clarity of research articles by considering the study protocol, including the design, sample collection, data analysis, and findings. A score of >6 indicates high quality, 4–6 indicates moderate quality, and <4 indicates low quality [15]. Following this, the Critical Appraisal Clinical Pharmacokinetic (CACPK) tool, a tool designed explicitly for PK studies, was used to check the quality of the included articles. This tool categorizes research studies as high, moderate, or low quality, for scores >13, between 12 and 13, or <12, respectively [16]. The scoring details of these quality assessment tools are presented in Supplementary Tables S2–S4.

### 2.5. Risk of Bias Assessment

Finally, the risk of bias was appraised using the Cochrane Collaboration Tool (CCT). The research studies with total scores of less than 3, 3 to 4, and greater than 4 were classified as high risk (HR), medium risk (MR), and low risk of bias (LR), respectively [17]. Moreover, the revised version of the risk of bias assessment tool for non-randomized studies (RoBANS 2) was employed in which clinical research studies with scores of <4, 4–5, and >5 were considered to be at HR, MR, and LR, respectively [18]. The risk of bias scoring is detailed in Supplementary Tables S5 and S6.

## 3. Results

### 3.1. Outcomes of the Literature Search

**Figure 2:**
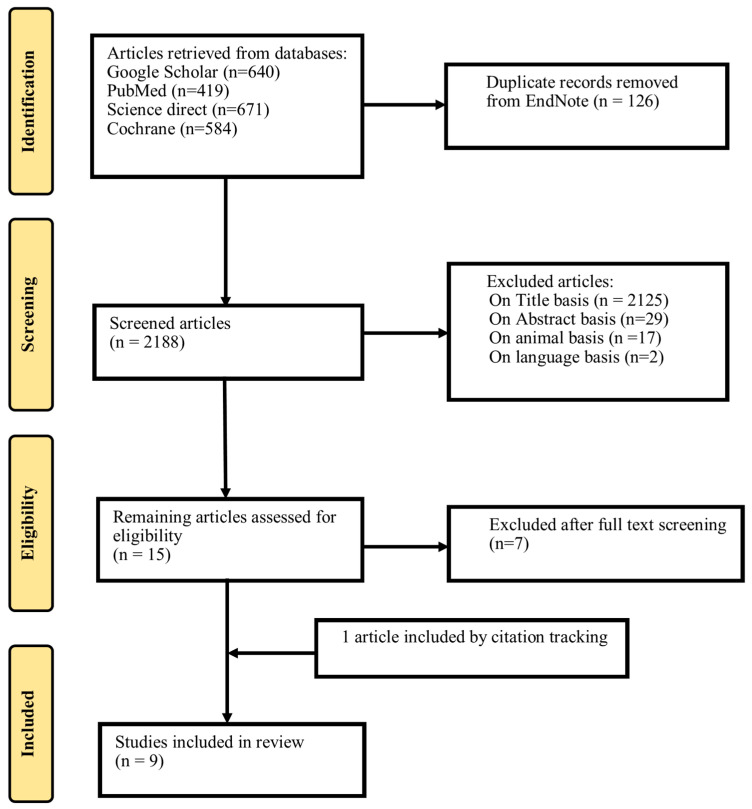
PRISMA workflow diagram.

After thoroughly screening different web databases, 2314 articles were retrieved: 640 from Google Scholar, 419 from PubMed, 671 from Science Direct, and 584 from Cochrane Central Library. After removing 126 duplicate records, the remaining 2188 articles were assessed, and 9 were included in the systematic review. The details of the inclusions and exclusions are presented in Figure 2.

### 3.2. Characteristics of Included Research Articles

The characteristics of the related articles, including author name, age, weight, population characteristics, gender distribution, number of subjects, dosage regimen, analytical method, relevant drug, and respective disabled conditions, are represented in Table 1.

### 3.3. Evaluation of Quality and Risk of Bias Findings

According to Jadad’s scoring, all articles were of low quality. In the CASP and CACPK scoring, all the clinical research studies were of high quality. Moreover, in the risk of bias assessment, the CCT indicated that all the articles had a low risk of bias. In contrast, in the case of the RoBANS 2 tool, eight articles had a low risk of bias, whereas two had a moderate risk of bias.

### 3.4. PK of Drugs in Adult Disabled Patients

Among the ten included clinical research studies, seven were conducted in adult disabled patients. In all of these studies, the CL and V_d_ values were reported as a population estimate (PE) with % relative standard error (RSE). Two of the studies reported CL values of 0.14 (12) L/h and 9.1 (6) L/h after per-oral (PO) administration of an 867 ± 514.2 mg/day dose of valproic acid among epileptic patients [20] and a 2.7 ± 1.2 mg/kg dose of tacrolimus in kidney transplant patients, respectively [25]. Another study recorded a CL value of 3.15 ± 0.37 L/h following PO propranolol administration in patients suffering from heart disease, hypertension, depressive disorder, and migraine [22].

Two different studies reported the CL and V_d_ of vancomycin after IV infusion among patients who underwent open heart surgery as 6.13 (19) L/h and 40 (15) L [23] and in cancer patients as 7.4 (20) L/h and 45 (15) L [26]. Moreover, in patients with coronary artery bypass graft (CABG) surgery, the CL of cefuroxime was reported as 2.23 (17) L/h after administration of 1.5 g by IV infusion [24]. Another study was conducted in cancer patients and reported the CL of micafungin as 1.2 (11.6) L/h following a dose of 100–150 mg/day via IV infusion [27]. The remaining values for the PK parameters are presented in Table 2.

### 3.5. PKs of Drugs in Pediatric Disabled Patients

The studies in pediatrics reported all the PK parameters, including AUC_0–∞_, CL, V_d_, C_max_, T_1/2_, and T_max_. Following administration of a 0.99 ± 0.15 mg/kg dose of busulphan via IV infusion, one study reported C_max_ of 1 ± 0.21 μg/mL in pediatric patients with hematopoietic stem cell transplantation [19].

Another study reported the PK parameters of carbamazepine among patients with partial or generalized epilepsy, with three different age groups, after administration of a PO dose of 4.19 ± 1.64 mg/kg. The values of CL for group 1 (1–5 years), group 2 (6–9 years), and group 3 (10–14 years) were 0.372 ± 0.154 L/h, 0.301 ± 0.133 L/h and 0.385 ± 0.391 L/h, respectively [21]. All other values of the PK parameters are detailed in Table 3.

## 4. Discussion

This comprehensive review has concentrated on gathering and evaluating the PK variables of all drugs in disabled patients among the Saudi Arabian population presented in the already reported clinical research articles. Out of nine studies, seven were recorded in adult patients suffering from epilepsy, heart diseases and cancer, as well as in patients who had undergone open heart surgery, CABG surgery or kidney transplant. The remaining three studies reported results for pediatric patients with disorders like hematopoietic stem cell transplantation, partial or generalized epilepsy, and cancer. Among the included drugs, five are metabolized by different CYP enzymes, among which tacrolimus, propranolol, and busulphan are high-extraction drugs, whereas carbamazepine and valproic acid are low-extraction drugs. Thus, the expression and activity of CYP enzymes change, in the case of these drugs, among patients with varying degrees of liver impairment [28], leading to therapeutic failure or adverse drug reactions.

Epilepsy is a neurological disease associated with a greater threat of functional disability (mental, physical, and social), and it has been reported that the risk is three times higher in comparison with non-epileptic individuals [29]. Keeping this in view, studies on the PKs of drugs used to treat partial or generalized epilepsy among the Saudi Arabian population are focused on in this review. Alqahtani et al. conducted a survey of the PKs of valproic acid [20] among adults, and the CL was found to be less than a previous study reported in Egyptian patients [30]. The decreased CL among the Saudi population may be due to the high dose of valproic acid used in their study. As it is a highly protein-bound drug, saturation occurs at increased plasma concentration, resulting in greater availability of the unbound drug on which metabolism occurs. Moreover, the CL was recorded to be the same among Chinese patients [31] (Table 4). Islam et al. reported the PKs of carbamazepine in another study among pediatrics with three age groups, i.e., Group 1(1–5 years), Group 2(6–9 years), and Group 3(10–14 years), finding a decreasing trend in values of C_max_ from group 1 to 3 [21], which may be due to the increased activity of hepatic enzymes in children. Moreover, substantial standard deviations are presented in Table 3, indicating significant variations in cumulative values for individual patients, as reported in the respective studies. This variation may be due to changes in patients’ underlying medical conditions. Therefore, the dose modification of these anti-epileptic drugs (valproic acid and carbamazepine) among adults and pediatrics should be monitored closely. In addition, a comparison of PK parameters with other studies is reported in Table 4 and shows dose-dependent increases in C_max_ and AUC_0–∞_ [32].

The incidence of disabilities among patients who have undergone open heart surgery or CABG surgery is higher, as reported in the previously published literature [44,45]. Super-infections resulting from microorganisms in surgeries are the main reason for disability; therefore, this review has included studies concentrating on the PKs of antibiotics used in the prophylaxis of these conditions. Alqahtani et al. reported vancomycin’s CL after 1 g of IV infusion [23]. Comparing their findings with those of another clinical study among Japanese subjects, there was a two-fold increase in the CL of vancomycin in the latter study [36], which suggests that changes in the dosage protocols due to differences in ethnicity are necessary. Moreover, comparing the results reported by Alqahtani et al. with those of two other studies [37,38], a three-fold decrease in CL among the population of Saudi Arabia can be observed (Table 4), suggesting the need for close dose monitoring. Moreover, a study in adult kidney transplant patients found that the CL of tacrolimus was lower than that reported in previous studies [39], suggesting that ethnicity and usage of other medications post-surgery were the main factors.

Physical and psychological disability is pronounced in patients suffering from cancer, in contrast to non-cancer patients, which leads to conditions like neutropenia and impacts on the quality of life [46]. Studies on cancer patients in the Saudi population are focused on in this comprehensive review, among which one reported the CL of vancomycin to be higher in adult cancer patients when compared with normal subjects [20]. These findings were similar to those reported in a previous study [40]. This may be due to the activation of organic cation and organic anion transporters by cytokines, which is amplified in patients suffering from cancer, and indicates the need for a 50% increase in doses. In another study, Alqahtani et al. [27] reported the increased CL of micafungin among adult cancer patients, possibly due to higher blood flow toward the liver as a result of interrupted liver vasculature activity. These findings were consistent with three previously published studies in different countries employing similar doses [41,42,43]. The strength of this review is that all articles published up till 10 November 2024 are included based on an exhaustive screening of the literature. Despite this, the current review has certain limitations. First, the total number of studies is insufficient to elaborate the variability of findings at the global level, and therefore, further studies are required for a more comprehensive review in future. Second, in this review, only a few drugs are focused on, and thus, the PK results may not be projected to other drugs. Third, the included studies have small groups of subjects, which may prevent validating outcomes. Fourth, to elaborate the variability of findings at the global level, further studies are required for a more comprehensive review in future, which is still not possible due to limited data.

## 5. Conclusions

This up-to-date review has summarized the PKs of various drugs in disabled patients (adults and pediatrics), the comprehension of which plays a pivotal role in optimizing the pharmacological therapy in the population of Saudi Arabia. The discrepancy observed in drug CL values, which decreased in patients with epilepsy and open heart surgery but increased among cancer patients, highlights the occurrence of alterations due to pathophysiological situations, thus paving the way for personalized dosage schedules. The complications addressed in these clinical research studies may be helpful for physicians to improve the safety and minimize the toxicity of each respective drug therapy.

## Figures and Tables

**Table 1:** Attributes of relevant articles included in the review.

S. No.	Reference	Population	Disabled Condition	Gender	Age (Years)	N	Analytical Method	Drug	Dose	Route	Frequency	Weight (kg)
1	Alsultan et al. (2020) [19]	Pediatrics	Hematopoietic stem cell transplantation	N/S	6.10 ± 3.17	59	LC-MS/MS	Busulphan	0.99 ± 0.15 mg/kg	IV infusion	QID for 4 days	N/S
2	Alqahtani et al. (2019) [20]	Adults	Epilepsy	42.5% M/57.5% F	36.3 ± 13.5	54	Fluorescence polarization immunoassay	Valproic acid	867 ± 514.2 mg	PO	OD	82.5 ± 26.8
3	Islam et al. (2013) [21]	Pediatrics	Partial or generalized epilepsy	50% M/50% F	8.23 ± 3.90	12	N/S	Carbamazepine	4.19 ± 1.64 mg/kg	PO	OD	26.18 ± 12.10
4	El-Yazigi et al. (1990) [22]	Adults	Heart disease, hypertension, depressive disorder, migraine	64.5% M/35.5% F	45.7 ± 1.8	48	N/S	Propranolol	85.8 ± 5.0 mg	PO	40 mg every 8 hr	73.3 ± 2.4
5	Alqahtani et al. (2018) [23]	Adults	Open heart surgery	61% M/39% F	51.7 ± 15.9	28	Architect i4000SR immunoassay analyzer	Vancomycin	1 g	IV infusion	q12 hr for 2 days	79.6 ± 17
6	Alqahtani et al. (2018) [24]	Adults	Coronary artery bypass graft surgery	76% M/24% F	54.2 ± 13.2	78	HPLC	Cefuroxime	1.5 g	IV infusion	TID	76.7 ± 14.7
7	Alqahtani et al. (2021) [25]	Adults	Kidney transplant	67%M/33% F	43.9 ± 13.5	139	Architect tacrolimus assay	Tacrolimus	2.7 ± 1.2 mg/kg	PO	OD	74.2 ± 19.5
8	Alqahtani et al. (2020) [26]	Adults	Cancer patients	58% M/42% F	53.8 ± 15.7	147	Architect iVancomycin i4000SR immunoassay analyzer	Vancomycin	1 g	IV infusion	BID	72.7 ± 16.2
9	Alqahtani et al. (2021) [27]	Adults	Cancer patients	60% M/40% F	47.3 ± 12.3	10	HPLC with UV detection	Micafungin	100–150 mg/day	IV infusion	OD	63.4 ± 18.2

M: male; F: female; N/S: not specified; IV: intravenous; PO: per-oral; N: number of subjects; OD: once a day; BID: twice a day; TID: thrice a day; QID: four times a day; LC-MS/MS: liquid chromatography-mass spectroscopy/mass spectroscopy; HPLC: high-pressure liquid chromatography.

**Table 2:** PK parameters of different drugs in adult disabled patients.

Sr. No.	Reference	Drug	Route	CL (L/h)	V_d_ (L)
1	Alqahtani et al. (2019) [20]	Valproic acid	PO	0.14 (12) ^a^	37.7
2	El-Yazigi et al. (1990) [22]	Propranolol	PO	3.15 ± 0.37 ^b^	N/S
3	Alqahtani et al. (2018) [23]	Vancomycin	IV Infusion	6.13 (19) ^a^	40 (15) ^a^
4	Alqahtani et al. (2018) [24]	Cefuroxime	IV Infusion	2.23 (17) ^a^	N/S
5	Alqahtani et al. (2021) [25]	Tacrolimus	PO	9.1 (6) ^a^	912 (3) ^a^
6	Alqahtani et al. (2020) [26]	Vancomycin	IV Infusion	7.4 (20) ^a^	45 (15) ^a^
7	Alqahtani et al. (2021) [27]	Micafungin	IV infusion	1.2 (11.6)	N/S

CL: clearance; V_d_: volume of distribution; N/S: not specified; IV: intravenous; PO: per-oral; ^a^ the values of CL and V_d_ are reported as population estimates along with % relative standard error (RSE) in brackets. ^b^ The values of CL are reported as mean ± standard error.

**Table 3:** PK parameters of different drugs in pediatric disabled patients.

Sr. No.	Reference	Drug	Route		Dose (mg/kg)	AUC_0–∞_ (μg h/mL)	C_max_ (μg/mL)	CL (L/h)	T_1/2_ (h)	V_d_ (L/kg)	T_max_ (h)
1	Alsultan et al. (2020) [19]	Busulphan	IV Infusion		0.99 ± 0.15	4392.5 ± 1354.65	1 ± 0.21	N/S	2.26 ± 0.47	N/S	N/S
2	Islam et al. (2013) [21]	Carbamazepine	PO	Group 1 ^a^	4.19 ± 1.64	13.63 ± 5.03	2.83 ± 1.12	0.37 ± 0.15	12.35 ± 9.22	7.91 ± 7.59	5.0 ± 2.58
				Group 2 ^b^		13.27 ± 7.79	2.64 ± 2.01	0.30 ± 0.13	10.48 ± 8.70	5.28 ± 6.53	5.5 ± 1.91
				Group 3 ^c^		13.58 ± 9.41	2.09 ± 1.39	0.38 ± 0.39	12.21 ± 3.20	5.28 ± 6.53	4.0 ± 1.63

AUC_0–∞_: area under curve from 0 to infinity; C_max_: maximal plasma concentration; CL: clearance; T_1/2_: half-life; V_d_: volume of distribution; T_max_: time at maximal plasma concentration; N/S: not specified; IV: intravenous; PO: per-oral; ^a^ Group 1: 1–5 years, ^b^ Group 2: 6–9 years, ^c^ Group 3: 10–14 years.

**Table 4:** PK parameters of different drugs in the Saudi Arabia population in comparison with parameters reported in different populations.

Sr. No.	Author	Drug	EV of SA	EV from Previous Reports	EV of SA	EV from Previous Reports	EV of SA	EV from Previous Reports
			C_max_ (μg/mL)		AUC_0–∞_ (μg∙h/mL)		CL (L/h)	
1	Alsultan et al. [19]	Busulphan	N/S	N/S	4392.35 ± 1354.65	4704.33 ± 767.63 [33]	N/S	N/S
2	Alqahtani et al. [20]	Valproic acid	N/S	N/S	N/S	N/S	0.14	0.09 [31]
								0.58 [30]
3	Islam et al. [21]	Carbamazepine	2.52 ± 1.447	7.10–9.92 [32]	13.49 ± 6.90	54.85–82.83 [32]	0.35 ± 0.23	0.01 ± 0.006 ^a^ [34]
4	El-Yazigi et al. [22]	Propranolol	N/S	N/S	N/S	N/S	3.15 ± 0.37	3.29 (0.54) ^b^ [35]
5	Alqahtani et al. [23]	Vancomycin	N/S	N/S	N/S	N/S	6.13 (19) ^b^	3.83 [36]
6	Alqahtani et al. [24]	Cefuroxime	N/S	N/S	N/S	N/S	2.23 (17) ^b^	7.27 (6) ^b^ [37]
								6.00 (3.2) ^b^ [38]
7	Alqahtani et al. [25]	Tacrolimus	N/S	N/S	N/S	N/S	9.1 (6) ^b^	10.01 [39]
8	Alqahtani et al. [26]	Vancomycin	N/S	N/S	N/S	N/S	7.4 (20) ^b^	71.2 + 22.2 ^c^ [40]
9	Alqahtani et al. [27]	Micafungin	N/S	N/S	N/S	N/S	1.2 (11.6) ^b^	1.27 [41]
								1.34 [42]
								1.10 [43]

AUC_0–∞_: area under curve from 0 to infinity; C_max_: maximal plasma concentration; CL: clearance; EV: estimated value; SA: Saudi Arabia; N/S: not specified; ^a^ CL is in L/h/kg. ^b^ Value in bracket is the relative standard error (RSE). ^c^ CL is in mL/min.

## Data Availability

All the data generated during the research are reported in the manuscript.

## Supplementary Materials

The following supporting information can be downloaded at: https://www.mdpi.com/article/10.3390/ph18040582/s1, Table S1: Screening and exclusion of articles based on title, abstract, animal, language, and full-text reading, Table S2: Quality assessment of included articles based on JADAD Scoring, Table S3: Quality assessment of included articles based on the Critical Appraisal Skill Program (CASP), Table S4: Quality assessment of included articles based on Critical Appraisal Clinical Pharmacokinetic (CACPK) tool, Table S5: Risk of bias assessment of included articles based on the Cochrane Collaboration tool (CCT), Table S6: Revised version of Risk of Bias Assessment for Nonrandomized Studies (RoBANS 2) Tool. References [19,20,21,22,23,24,25,26,27] are cited in Supplementary Materials.
