# Supplementary material for: Exploring the Pharmacokinetics of Drugs in Disabled Saudi Patients: A Systematic Review
Source: Pharmaceuticals (Basel). 2025 Apr 16;18(4):582. doi: 10.3390/ph18040582 (PMC12030500; doi:10.3390/ph18040582)
Supplement: Supplementary file 1 [file pharmaceuticals-18-00582-s001.zip › pharmaceuticals-3511413-supplementary.pdf]

# Exploring the pharmacokinetics of drugs in disabled Saudi patients: A systematic review

**Faleh Alqahtani [1,*], Saeed A. Al Awadh [2,3] and Muhammad Fawad Rasool [4,*]**

[1] Department of Pharmacology and Toxicology, College of Pharmacy, King Saud University, Riyadh 11451, Saudi Arabia
[2] Saudi Food and Drug Authority, Drug Sector, Riyadh, Saudi Arabia
[3] King Salman Center for Disability Research, Riyadh 11614, Saudi Arabia; saeed19862@gmail.com
[4] Department of Pharmacy Practice, Faculty of Pharmacy, Bahauddin Zakariya University, Multan 60800, Pakistan
* Correspondence: afaleh@ksu.edu.sa (F.A.); fawadrasool@bzu.edu.pk (M.F.R.)

**Supplementary Table S1: Screening and exclusion of articles based on title, abstract, animal, language, and full-text reading**

| Sr # | Title of the article | Exclusion basis |
|---|---|---|
| 1 | Aarons, L., et al. (2005). "Estimation of Population Pharmacokinetic Parameters of Free-Phenytoin in Adult Epileptic Patients." Archives of Medical Research 36(1): 49-53. | Full-text reading |
| 2 | Abanmy, N. O., et al. (2024). "Clinical pharmacists' knowledge, attitude, perception, and beliefs about the role of pharmacogenetic testing for genes polymorphisms when prescribing mercaptopurine." Saudi Pharmaceutical Journal 32(4): 102022. | Title |
| 3 | Abbas, A. E. F., et al. (2024). "Sustainable quantification of glycopyrronium, indacaterol, and mometasone along with two genotoxic impurities in a recently approved fixed-dose breezhaler formulations and biological fluids: A machine learning-augmented UV-spectroscopic approach." Microchemical Journal 206: 111586. | Title |
| 4 | Abbasi, A., et al. (2020). "Dear Reader, Welcome to the final issue of European Journal of Drug Metabolism and Pharmacokinetics for 2020. This year has presented many challenges and I would like to take this opportunity to thank all who have contributed to ensuring the journal has thrived despite the extraordinary circumstances that the science community, and the world in general, has faced."  45: 693-695. | Title |
| 5 | Abd El-Aziz, G., et al. (2021). "Betanin and Allicin Ameliorate Adriamycin-Induced Cardiotoxicity in Rats by Ameliorating Cardiac Ischemia and Improving Antioxidant Efficiency."  33(7): 39-56. | Title |
| 6 | Abdallah, M. S., et al. (2021). "The AMPK modulator metformin as adjunct to methotrexate in patients with rheumatoid arthritis: A proof-of-concept, randomized, double-blind, placebo-controlled trial." International Immunopharmacology 95: 107575. | Title |
| 7 | Abdallah, Q. M., et al. (2020). "Utilization of novel self-nanoemulsifying formulations (SNEFs) loaded paclitaxel for the treatment prosperity of bladder cancer." Journal of Drug Delivery Science and Technology 56: 101514. | Title |
| 8 | Abdel Salam, L., et al. (2021). "Whole exome sequencing (WES) of methotrexate response/adverse event profile in rheumatoid arthritis patients." The Egyptian Rheumatologist 43(4): 287-291. | Title |
| 9 | Abdel-Aziz, A. K., et al. (2022). "A critical review of chloroquine and hydroxychloroquine as potential adjuvant agents for treating people with cancer."  2(4): 431-443. | Title |
| 10 | Abdelgawad, M. A., et al. (2022). "Design, synthesis, and biological evaluation of novel pyrido-dipyrimidines as dual topoisomerase II/FLT3 inhibitors in leukemia cells." Bioorganic Chemistry 122: 105752. | Title |

| | | |
|---|---|---|
| 11 | Abdelmassih, M. M., et al. (2024). "Repurposing fusidic acid as an antimicrobial against enterococci with a low probability of resistance development." 1-13. | Title |
| 12 | Abdelraouf, K., et al. (2020). "In vivo pharmacodynamics of new-generation β-lactamase inhibitor taniborbactam (formerly VNRX-5133) in combination with cefepime against serine-β-lactamase-producing Gram-negative bacteria." 75(12): 3601-3610. | Title |
| 13 | Abdel-Salam Elgohary, M., et al. (2024). "Even one dose of tocilizumab could hinder bad prognosis of cytokines storm in COVID-19 patients." Cytokine 173: 156433. | Title |
| 14 | Abdelwahab, S. I., et al. (2024). "Insights into frankincense and myrrh research: A comprehensive analytical study of patterns and perspectives." Heliyon 10(19): e38102. | Title |
| 15 | Abdelwahab, S. I., et al. (2023). "Bibliometric mapping of solid lipid nanoparticles research (2012–2022) using VOSviewer." Medicine in Novel Technology and Devices 17: 100217. | Title |
| 16 | Abdulaal, W. H., et al. (2024). "Redirecting pantoprazole as a metallo-beta-lactamase inhibitor in carbapenem-resistant Klebsiella pneumoniae." 15: 1366459. | Title |
| 17 | Abdullah Alsultan, A. A., et al. (2017). "Population pharmacokinetics of pyrazinamide in patients with tuberculosis." | Abstract |
| 18 | Abdul-Mutakabbir, J. C., et al. (2020). 1295. activity of SPR206, a polymyxin B derivative, compared to colistin alone and in combination against multidrug-resistant Pseudomonas aeruginosa strains. Open Forum Infectious Diseases, Oxford University Press US. | Title |
| 19 | Abdulqader, G. M. R. J. O. M. (2024). "Frequency and clinicopathological correlation of gastrointestinal polyps: A six-year single center experience." 19(1): 20241022. | Title |
| 20 | Abdussalam, A., et al. (2018). "Dietary-induced obesity and changes in the biodistribution and metabolism of amiodarone in the rat." 107(11): 2938-2945. | Animal |
| 21 | Abebe, B. T., et al. (2019). "Effects of the P-Glycoprotein Inhibitor Clarithromycin on the Pharmacokinetics of Intravenous and Oral Trospium Chloride: a 4-Way Crossover Drug-Drug Interaction Study in Healthy Subjects." J Clin Pharmacol 59(10): 1319-1330. | Abstract |
| 22 | Abo El-Enin, H. A., et al. (2022). "Utilization of polymeric micelles as a lucrative platform for efficient brain deposition of olanzapine as an antischizophrenic drug via intranasal delivery." 15(2): 249. | Title |
| 23 | Aboelhadid, S. M., et al. (2024). "The efficacy of essential oil components with ivermectin against Rhipicephalus annulatus: An in-vitro study." Veterinary Parasitology 332: 110335. | Title |

| | | |
|---|---|---|
| 24 | Abou-Auda, H. S. (1998). "Comparative pharmacokinetics and pharmacodynamics of furosemide in Middle Eastern and in Asian subjects." International journal of clinical pharmacology and therapeutics 36(5): 275-281. | Abstract |
| 25 | Abou-Auda, H. S., et al. "Population pharmacokinetics of phenytoin from routine clinical data in Saudi Epileptic Patients." | Full-text reading |
| 26 | Abouelhassan, Y., et al. (2024). "Defining optimal sulbactam regimens for treatment of Acinetobacter baumannii pneumonia and impact of bla OXA-23 on efficacy." 79(9): 2306-2316. | Title |
| 27 | Abouelkheir, M., et al. (2024). "Evaluation of Pharmacokinetic Pharmacodynamic Target Attainment and Hematological Toxicity of Linezolid in Pediatric Patients." | Abstract |
| 28 | Abouelkheir, M., et al. (2023). "Evaluation of vancomycin individualized model-based dosing approach in neonates." Pediatrics & Neonatology 64(3): 327-334. | Abstract |
| 29 | Aboufaras, M., et al. (2023). "Efficacies and side effects of medicinal plants used by patients with cancer in Morocco: A retrospective treatment-outcome study." 301: 115783. | Title |
| 30 | Abou-Khalil, B. (2005). "Benefit-risk assessment of levetiracetam in the treatment of partial seizures." Drug Saf 28(10): 871-890. | Title |
| 31 | Abou-Taleb, H. A., et al. (2024). "HPMC-Zein Film-forming Gel Loaded with 5-Fluorouracil Coupled with CO(2) Laser Dermabrasion for Managing Stable Vitiligo." AAPS PharmSciTech 25(7): 225. | Title |
| 32 | Abou-Taleb, H. A., et al. (2024). "In vitro and in vivo evaluation of isoxsuprine loaded invasomes for efficient treatment of diabetes-accelerated atherosclerosis." Journal of Drug Delivery Science and Technology 96: 105686. | Title |
| 33 | Abouzid, M., et al. (2022). "Attitudes toward Receiving COVID-19 Booster Dose in the Middle East and North Africa (MENA) Region: A Cross-Sectional Study of 3041 Fully Vaccinated Participants." Vaccines (Basel) 10(8). | Title |
| 34 | Abroug, F., et al. (1999). "Serotherapy in scorpion envenomation: a randomised controlled trial." The Lancet 354(9182): 906-909. | Title |
| 35 | Abuasal, B., et al. (2022). "Clinical Pharmacology in Drug Development for Rare Diseases in Neurology: Contributions and Opportunities." Clin Pharmacol Ther 111(4): 786-798. | Title |
| 36 | Abu-Izneid, T., et al. (2024). "Discovery of new $\alpha$-glucosides, antiglycation agent, and in silico study of 2-(3,4-dihydroxyphenyl)-7,8-dihydroxy-3-methoxy-4H-chromen-4-one isolated from Pistacia chinensis." Heliyon 10(5): e27298. | Title |

| | | |
|---|---|---|
| 37 | Abulseoud, O. A., et al. (2022). "Ceftriaxone as a novel therapeutic agent for hyperglutamatergic states: Bridging the gap between preclinical results and clinical translation." 16: 841036. | Title |
| 38 | Abu-Qurain, H., et al. (2020). "A retrospective study on therapeutic drug monitoring of mood stabilizers in real-life clinical scenario." J Pharm Bioallied Sci 12(3): 351-355. | Title |
| 39 | Aburisheh, K. H., et al. (2024). "Neonatal Outcomes in Patients with Gestational Diabetes Mellitus Treated with Metformin: A Retrospective Study in Saudi Arabia." 12(9): 2040. | Abstract |
| 40 | Abu-Shaheen, A., et al. (2018). "Testing the validity and reliability of the Arabic version of the painDETECT questionnaire in the assessment of neuropathic pain." PLoS One 13(4): e0194358. | Title |
| 41 | Abutaha, N. and B. O. Almutairi (2023). "Exploring the therapeutic potential of GC–MS separated compounds from Dracaena cinnabari against dengue virus and Aedes aegypti using in silico tools." Journal of King Saud University - Science 35(2): 102478. | Title |
| 42 | Acharjee, S., et al. (2013). "Understanding Type 1 Diabetes: Etiology and Models." Canadian Journal of Diabetes 37(4): 269-276. | Title |
| 43 | Adams, D., et al. (2017). "Patisiran, an investigational RNAi therapeutic for patients with hereditary transthyretin-mediated (hATTR) amyloidosis with polyneuropathy: results from the phase 3 apollo study." Orphanet journal of rare diseases 12. | Title |
| 44 | Adams, J. L., et al. (2016). "Utilizing remote blood pressure monitoring in a phase III clinical drug trial for Parkinson's disease." Movement disorders 31: S687. | Title |
| 45 | Adas, M. A., et al. (2022). "The infection risks of JAK inhibition." 18(3): 253-261. | Title |
| 46 | Adatia, A., et al. (2012). "Osteoarthritis of the knee and hip. Part II: therapy with ibuprofen and a review of clinical trials." J Pharm Pharmacol 64(5): 626-636. | Title |
| 47 | Adéoti, O. M., et al. (2024). "Nonlinear mixed models and related approaches in infectious disease modeling: A systematic and critical review." | Title |
| 48 | Adey-Wakeling, Z., et al. (2013). "Suprascapular nerve block for shoulder pain in the first year after stroke: a randomised controlled trial." Arthritis Rheum 65: S464. | Title |
| 49 | Afsar, N. A., et al. (2019). "Implications of genetic variation of common drug metabolizing enzymes and ABC transporters among the Pakistani population." 9(1): 7323. | Title |

| | | |
|---|---|---|
| 50 | Agaliotis, M., et al. (2013). "A longitudinal study of work disability among people with chronic knee pain." Annals of the rheumatic disease 71. | Title |
| 51 | Agarwal, A., et al. (2020). "A living WHO guideline on drugs for covid-19." Bmj 370: m3379. | Title |
| 52 | Ageel, M. (2024). "Review of pediatric sedation and anesthesia for radiological diagnostic and therapeutic procedures." Journal of Radiation Research and Applied Sciences 17(1): 100833. | Title |
| 53 | Agrawal, L., et al. (2008). "HIV infection and AIDS." 21: 92-117. | Title |
| 54 | Aguilera-Alonso, D., et al. (2020). "Carbapenem-resistant gram-negative bacterial infections in children." 64(3): 10.1128/aac. 02183-02119. | Title |
| 55 | Ahad, A., et al. (2022). "Changes in pharmacokinetics and pharmacodynamics of losartan in experimental diseased rats treated with Curcuma longa and Lepidium sativum." 16(1): 33. | Animals |
| 56 | Ahmad, A., et al. (2016). "Synthesis, antimicrobial and antitubercular activities of some novel pyrazoline derivatives." Journal of Saudi Chemical Society 20(5): 577-584. | Title |
| 57 | Ahmad Khan, H., et al. (2004). "Metoclopramide attenuates iminodipropionitrile-induced oxidative stress and neurobehavioral toxicity in rats." Pharmacology Biochemistry and Behavior 79(3): 555-561. | Animal |
| 58 | Ahmad, M. Z., et al. (2022). "Nanoscale topical pharmacotherapy in management of psoriasis: Contemporary Research and scope." 14(1): 19. | Title |
| 59 | Ahmad, N., et al. (2023). "Beneficial effects of topical 6-gingerol loaded nanoemulsion gel for wound and inflammation management with their comparative dermatokinetic." Journal of Drug Delivery Science and Technology 80: 104094. | Title |
| 60 | Ahmad, S., et al. (2024). "Spectrum and management of rare Candida/yeast infections in Kuwait in the Middle East." 11: 20499361241263733. | Title |
| 61 | Ahmed, B., et al. (2021). "Synthesis of gallotannin capped iron oxide nanoparticles and their broad spectrum biological applications††Electronic supplementary information (ESI) available. See DOI: 10.1039/d1ra00220a." RSC Advances 11(17): 9880-9893. | Title |
| 62 | Ahmed, K. A., et al. (2024). "Population Pharmacokinetics and Model-Based Dose Optimization of Vancomycin in Sudanese Adult Patients with Renal Impairment." Drug Des Devel Ther 18: 81-95. | Title |
| 63 | Ahmed, M., et al. (2022). "Single Nucleotide Polymorphisms Associated with the Safety and Efficacy of Anti-Gout Medications." 2(2): 133-154. | Title |

| | | |
|---|---|---|
| 64 | Ahmed, M. H., et al. (2024). "Identification of therapeutic drug target of Shigella Flexneri serotype X through subtractive genomic approach and in-silico screening based on drug repurposing." Infection, Genetics and Evolution 122: 105611. | Title |
| 65 | Ahmed, M. M., et al. (2020). "Development and characterization of Brigatinib loaded solid lipid nanoparticles: In-vitro cytotoxicity against human carcinoma A549 lung cell lines." Chemistry and Physics of Lipids 233: 105003. | Title |
| 66 | Ahmed, O. A., et al. (2016). "Optimisation of microstructured biodegradable finasteride formulation for depot parenteral application." 33(3): 229-238. | Title |
| 67 | Ain, N. U., et al. (2024). "Bleeding Events Associated with Rivaroxaban Therapy in Naive Patients with Nonvalvular Atrial Fibrillation: A Longitudinal Study from a Genetic Perspective with INR Follow-Up." 60(10): 1712. | Title |
| 68 | Ajmal, M. R., et al. (2016). "Interaction of new kinase inhibitors cabozantinib and tofacitinib with human serum alpha-1 acid glycoprotein. A comprehensive spectroscopic and molecular Docking approach." Spectrochimica Acta Part A: Molecular and Biomolecular Spectroscopy 159: 199-208. | Title |
| 69 | Akash, S., et al. (2023). "Mechanistic inhibition of gastric cancer-associated bacteria Helicobacter pylori by selected phytocompounds: A new cutting-edge computational approach." Heliyon 9(10): e20670. | Title |
| 70 | Akash, S., et al. (2024). "Discovery of novel MLK4 inhibitors against colorectal cancer through computational approaches." Computers in Biology and Medicine 182: 109136. | Title |
| 71 | Akkawi El Edelbi, R., et al. (2021). "Estimation of body surface area in neonates, infants, and children using body weight alone." International Journal of Pediatrics and Adolescent Medicine 8(4): 221-228. | Title |
| 72 | Akosman, I., et al. (2023). "Is high-dose tranexamic safe in spine surgery? A systematic review and meta-analysis." 13(7): 2085-2095. | Title |
| 73 | Akrasi, W., et al. (2022). "Adverse drug effects among students following mass de-worming exercise involving administration of Praziquantel and Albendazole in KEEA Municipality, Ghana." 16(9): e0010680. | Title |
| 74 | Akuthota, V., et al. (2012). "Effectiveness of physical therapy as an adjunct to epidural steroid injections in the treatment of lumbar spinal stenosis: a pilot randomized controlled trial." Spine journal 12(9): 146S. | Title |
| 75 | Al Amer, H. S., et al. (2020). "Cross-cultural adaptation and psychometric testing of the Arabic version of the Modified Low Back Pain Disability Questionnaire." PLoS One 15(4): e0231382. | Title |

| | | |
|---|---|---|
| 76 | Al Ammari, M., et al. (2020). "The effect of the VKORC1 promoter variant on warfarin responsiveness in the Saudi WArfarin Pharmacogenetic (SWAP) cohort." Sci Rep 10(1): 11613. | Title |
| 77 | Al Bahrani, S., et al. (2021). "Safety and Reactogenicity of the ChAdOx1 (AZD1222) COVID-19 Vaccine in Saudi Arabia." International Journal of Infectious Diseases 110: 359-362. | Title |
| 78 | Al Faraj, A., et al. (2016). "Combination of drug-conjugated SWCNT nanocarriers for efficient therapy of cancer stem cells in a breast cancer animal model." Journal of Controlled Release 225: 240-251. | Title |
| 79 | Al Fayez, N., et al. (2022). "Hepatocyte-targeted delivery of imiquimod reduces hepatitis B virus surface antigen." Journal of Controlled Release 350: 630-641. | Title |
| 80 | Al Garadi, W., et al. (2024). "Indapamide analogue a promising drug: Synthesis, a novel crystal structure, HSA/DFT/XRD, greener pastures biological study." Journal of Molecular Structure 1295: 136593. | Title |
| 81 | Al Hassan, Y. T., et al. (2021). "Association of vaccine awareness and confidence on the influenza vaccination status of Al Ahsa, Saudi Arabia residents." Human vaccines & immunotherapeutics 17(7): 2190-2196. | Title |
| 82 | Al Jalali, V. and M. Zeitlinger (2020). "Systemic and Target-Site Pharmacokinetics of Antiparasitic Agents." Clin Pharmacokinet 59(7): 827-847. | Title |
| 83 | Al Khaja, K. A. and R. P. J. M. j. Sequeira (2021). "Drug treatment and prevention of malaria in pregnancy: a critical review of the guidelines." 20: 1-13. | Title |
| 84 | Al Nebaihi, H. (2019). "Impact of Obesity on Metabolism of Some Selected Anesthetic Agents." | Title |
| 85 | Al Odhayani, A., et al. (2017). "Potentially inappropriate medications prescribed for elderly patients through family physicians." Saudi Journal of Biological Sciences 24(1): 200-207. | Title |
| 86 | Al Rahmany, D., et al. (2019). "Exploring bacterial resistance in Northern Oman, a foundation for implementing evidence-based antimicrobial stewardship program." International Journal of Infectious Diseases 83: 77-82. | Title |
| 87 | Al Sulaiman, K., et al. (2023). "When antivirals backfire: An evaluation of favipiravir's clinical outcomes in critically ill patients with COVID-19: A multicenter cohort study." Journal of Infection and Public Health 16(9): 1492-1499. | Title |
| 88 | Al Sulaiman, K., et al. (2021). "The impact of early target attainment of vancomycin in critically ill patients with confirmed Gram-positive infection: A retrospective cohort study." BMC Infect Dis 21(1): 1182. | Title |

| | | |
|---|---|---|
| 89 | Al Wakeel, J., et al. (2012). "Cyclosporine Microemulsion Formulation (Sigmasporin Microral) Effect as First-Line Immunosuppressant on Renal Functions at 3 Years." Transplantation Proceedings 44(1): 94-100. | Title |
| 90 | Al Wakeel, J. S., et al. (2008). "Six-Month Clinical Outcome of Cyclosporine Microemulsion Formulation (Sigmasporin Microral) in Stable Renal Transplant Patients Previously Maintained on Sandimmun Neoral." Transplantation Proceedings 40(7): 2245-2251. | Title |
| 91 | Al Yami, M. S. (2017). "Comparison of the incidence of acute kidney injury during treatment with vancomycin in combination with piperacillin–tazobactam or with meropenem." Journal of Infection and Public Health 10(6): 770-773. | Title |
| 92 | Al-Abbas, N. S. and N. A. Shaer (2021). "Combination of coumarin and doxorubicin induces drug-resistant acute myeloid leukemia cell death." Heliyon 7(3): e06255. | Title |
| 93 | Alabdulkarim, A., et al. (2023). "Effect of tranexamic acid on the reduction of blood loss in craniosynostosis surgery: a systematic review and meta-analysis."  11(6): e5021. | Title |
| 94 | Alabdulwahab, A. S., et al. (2017). "The Dana Farber Consortium Protocol for the Treatment of Adolescents and Young Adults With Acute Lymphoblastic Leukemia: A Single Institution Experience in Saudi Arabia." Clinical Lymphoma Myeloma and Leukemia 17(5): 320-325. | Title |
| 95 | Al-Ajlan, N., et al. (2019). "One year prospective follow up of fluvastatin as an add-on disease modifying therapy in established relapsing multiple sclerosis." Multiple sclerosis journal 25: 874. | Title |
| 96 | Alakeel, Y. S., et al. (2024). "An evaluation of the empirical vancomycin dosing guide in pediatric cardiology."  24(1): 1-10. | Title |
| 97 | Al-Alaiyan, S., et al. (2001). "Caffeine metabolism in premature infants."  41(6): 620-627. | Title |
| 98 | Alam, S., et al. (2021). "Therapeutic effectiveness and safety of repurposing drugs for the treatment of COVID-19: position standing in 2021." 12: 659577. | Title |
| 99 | Alame, M. M., et al. (2016). "Peramivir: a novel intravenous neuraminidase inhibitor for treatment of acute influenza infections."  7: 450. | Title |
| 100 | Alammar, H., et al. (2021). "Deficiency in the treatment description of mTOR inhibitor resistance in medulloblastoma, a systematic review." 23(1): 464. | Title |
| 101 | Alamri, M. A. (2023). "Bioinformatics and network pharmacology-based study to elucidate the multi-target pharmacological mechanism of the indigenous plants of Medina valley in treating HCV-related hepatocellular carcinoma." Saudi Pharmaceutical Journal 31(6): 1125-1138. | Title |
| 102 | Alangari, A. A., et al. (2014). "Budesonide Nebulization Added to Systemic Prednisolone in the Treatment of Acute Asthma in Children: A Double-Blind, Randomized, Controlled Trial." Chest 145(4): 772-778. | Title |

| | | |
|---|---|---|
| 103 | Al-Asmari, A., et al. (2010). "Method for the quantification of diamorphine and its metabolites in pediatric plasma samples by liquid chromatography-tandem mass spectrometry." 34(4): 177-195. | Title |
| 104 | Alasmari, F., et al. (2023). "Physiologically-based pharmacokinetic modeling for single and multiple dosing regimens of ceftriaxone in healthy and chronic kidney disease populations: a tool for model-informed precision dosing." Front Pharmacol 14: 1200828. | Title |
| 105 | Alasmari, M. S., et al. (2022). "Development and Evaluation of a Physiologically Based Pharmacokinetic Model for Predicting Haloperidol Exposure in Healthy and Disease Populations." Pharmaceutics 14(9). | Title |
| 106 | Albabtain, M. A., et al. (2023). "Real-world Experience in Managing Atrial Fibrillation in Patients with Renal Impairment; Rivaroxaban versus Warfarin." 24(3): 136-140. | Title |
| 107 | Al-Balawi, R. S., et al. (2020). "Measuring the appropriateness of carbamazepine and valproic acid prescribing and utilization using a newly implemented online system in the Tabuk Region of Saudi Arabia." Saudi Pharmaceutical Journal 28(7): 844-849. | Abstract |
| 108 | Alballa, S. R., et al. (1992). "Randomized, double-blind, short-term trial of nabumetone versus diclofenac in osteoarthritis of the knee." Current Therapeutic Research 52(4): 581-586. | Title |
| 109 | Albanna, A. S. and D. J. D. Menzies (2011). "Drug-resistant tuberculosis: what are the treatment options?" 71(7): 815-825. | Title |
| 110 | Albassam, A. A., et al. (2021). "The potential of drug-herbal interaction among patients with chronic diseases in Saudi Arabia." Complementary Therapies in Clinical Practice 43: 101324. | Title |
| 111 | Albassam, A. A. and R. F. Frye (2019). "Effect of pterostilbene on in vitro drug metabolizing enzyme activity." Saudi Pharmaceutical Journal 27(3): 406-412. | Title |
| 112 | Albayrak, A., et al. (2023). "Impact of clinical pharmacist's interventions on clinical outcomes in appropriate use of Colistin: a prospective pre-post intervention study." 35(8): 712-720. | Title |
| 113 | Albert, S. M., et al. (2016). "Design and Recruitment for a Randomized Controlled Trial of Problem-Solving Therapy to Prevent Depression among Older Adults with Need for Supportive Services." American journal of geriatric psychiatry 24(1): 94-102. | Title |
| 114 | Albilal, S., et al. (2023). "Tacrolimus Trough Level Variation and Its Correlation to Clinical Outcomes and Consequences in Solid Organ Transplantation." 1-11. | Title |
| 115 | Albogami, S. M., et al. (2021). "Effects of neoadjuvant therapies on genetic regulation of targeted pathways in ER+ primary ductal breast carcinoma: A meta-analysis of microarray datasets." Saudi Pharmaceutical Journal 29(7): 656-669. | Title |

| | | |
|---|---|---|
| 116 | Albonico, M., et al. (1999). Control Strategies for Human Intestinal Nematode Infections. Advances in Parasitology. J. R. Baker, R. Muller and D. Rollinson, Academic Press. 42: 277-341. | Title |
| 117 | ALBrahim, Z. A. (2023). Molecular characterization of Klebsiella pneumoniae isolated from different clinical sources in Eastern and Western Provinces of Saudi Arabia Tertiary Hospitals, KING ABDULAZIZ UNIVERSITY. | Title |
| 118 | Aldeyab, M. A., et al. (2014). "A modified method for measuring antibiotic use in healthcare settings: implications for antibiotic stewardship and benchmarking." 69(4): 1132-1141. | Title |
| 119 | Al-Dhubiab, B. E. (2016). "In vitro and in vivo evaluation of nano-based films for buccal delivery of zolpidem." Braz Oral Res 30(1): e126. | Title |
| 120 | Al-Dhubiab, B. E., et al. (2015). "Formulation and evaluation of nano based drug delivery system for the buccal delivery of acyclovir." Colloids and Surfaces B: Biointerfaces 136: 878-884. | Title |
| 121 | Al-Dorzi, H. M., et al. (2014). "Antibiotic therapy of pneumonia in the obese patient: dosing and delivery." 27(2): 165-173. | Title |
| 122 | Al-Dorzi, H. M., et al. (2015). "Impact of empirical antimicrobial therapy on the outcome of critically ill patients with Acinetobacter bacteremia." 10(4): 256-262. | Title |
| 123 | Alegria, M. (2022). "The Challenge to Be Inclusive and Diversify Our Aging Populations." American journal of geriatric psychiatry 30(4): 475-477. | Title |
| 124 | Alehaideb, Z., et al. (2021). "Commiphora myrrha (Nees) Engl. resin extracts induce phase-I cytochrome P450 2C8, 2C9, 2C19, and 3A4 isoenzyme expressions in human hepatocellular carcinoma (HepG2) cells." Saudi Pharmaceutical Journal 29(5): 361-368. | Title |
| 125 | Aleissa, M. S., et al. (2022). "Comparative study of the anti-diabetic effect of mucilage and seed extract of Abelmoschus esculentus against streptozotocin-induced diabetes in rat model." Journal of King Saud University - Science 34(8): 102297. | Title |
| 126 | Al-Eitan, L. N., et al. (2019). "Genetic polymorphisms of CYP3A5, CHRM2, and ZNF498 and their association with epilepsy susceptibility: a pharmacogenetic and case-control study." Pharmgenomics Pers Med 12: 225-233. | Title |
| 127 | Al-Eitan, L. N., et al. (2021). "Influence of CYP4F2, ApoE, and CYP2A6 gene polymorphisms on the variability of Warfarin dosage requirements and susceptibility to cardiovascular disease in Jordan." Int J Med Sci 18(3): 826-834. | Title |
| 128 | Al-Eitan, L. N., et al. (2020). "Analysis of comprehensive pharmacogenomic profiling of VIP variants among the genetically isolated Chechen subpopulation from Jordan." 199-215. | Title |

| | | |
|---|---|---|
| 129 | Alemao, E., et al. (2014). "Impact of anti-IL-6 monoclonal antibody, clazakizumab, on patient-reported outcomes in patients with rheumatoid arthritis and an inadequate response to methotrexate in a phase IIB study." Ann Rheum Dis 73. | Title |
| 130 | Alenazi, A., et al. (2023). "Extended infusion versus intermittent infusion of Piperacillin/tazobactam: altering current methods to optimize future outcome." 21(3): 1-9. | Title |
| 131 | Alessandrini, M., et al. (2013). "Cytochrome P450 pharmacogenetics in African populations." 45(2): 253-275. | Title |
| 132 | Alessandrini, M. and M. S. J. P. Pepper (2014). "Priority pharmacogenetics for the African continent: focus on CYP450." 15(3): 385-400. | Title |
| 133 | Alexiou, A., et al. (2023). "The Fractal Viewpoint of Tumors and Nanoparticles." Curr Med Chem 30(3): 356-370. | Title |
| 134 | Al-Eyadhy, A. and M. R. Al-Jelaify (2022). "Suboptimal vancomycin levels in critically ill children with sickle cell disease and acute chest syndrome." Journal of Infection and Chemotherapy 28(9): 1304-1309. | Title |
| 135 | Alfadhel, M. M., et al. (2023). "Numerical Optimization of Prednisolone–Tacrolimus Loaded Ultraflexible Transethosomes for Transdermal Delivery Enhancement; Box–Behnken Design, Evaluation, Optimization, and Pharmacokinetic Study." 9(5): 400. | Title |
| 136 | Alfahad, W. A. and A. S. J. S. M. J. Omrani (2014). "Update on colistin in clinical practice." 35(1): 9-19. | Title |
| 137 | Alfarisi, O. (2019). PHARMACOKINETIC/PHARMACODYNAMIC (PK/PD) MODELLING: APPROACHES TO ASSESS THE INTERACTIONS BETWEEN TUBERCULOSIS AND DIABETES OR HIV, Johns Hopkins University. | Title |
| 138 | Alfehaid, F. S., et al. (2024). "Enhanced transdermal delivery of apremilast loaded ethosomes: Optimization, characterization and in vivo evaluation." Journal of Drug Delivery Science and Technology 91: 105211. | Title |
| 139 | Alfenim, S. C. T. (2021). Geriatric Medicines Development: Improving Regulatory Strategies Towards Establishing an Evidence-Based Benefit-Risk Balance in the Geriatric Population, Universidade de Lisboa (Portugal). | Title |
| 140 | Algabbani, A. M., et al. (2023). "The inadvertent consequences of drug recalls: A case study of a recall of pantoprazole generics from the markets." Saudi Pharmaceutical Journal 31(7): 1181-1185. | Title |
| 141 | Algahtani, M. S., et al. (2018). "Extrusion-Based 3D Printing for Pharmaceuticals: Contemporary Research and Applications." Curr Pharm Des 24(42): 4991-5008. | Title |
| 142 | Alghamdi, A. and S. J. C. P. R. Husain (2015). "Management of fungal infections in lung transplant recipients." 4: 63-70. | Title |
| 143 | Alghamdi, A. H., et al. (2023). "The use of medicinal plants in common ophthalmic disorders: A systematic review with meta-analysis." Heliyon 9(4): e15340. | Title |

| | | |
|---|---|---|
| 144 | Alghamdi, W. A., et al. (2020). "Population pharmacokinetics of linezolid in tuberculosis patients: dosing regimen simulation and target attainment analysis."  64(10): 10.1128/aac. 01174-01120. | Title |
| 145 | Alghamdi, W. A., et al. (2021). "Pharmacokinetics of bedaquiline, delamanid and clofazimine in patients with multidrug-resistant tuberculosis."  76(4): 1019-1024. | Title |
| 146 | Alghamdi, W. A., et al. (2019). "Cycloserine population pharmacokinetics and pharmacodynamics in patients with tuberculosis." Antimicrob Agents Chemother  63(5): 10.1128/aac. 00055-00019. | Abstract |
| 147 | Alghamdi, W. A., et al. (2019). "Population pharmacokinetics of efavirenz in HIV and TB/HIV coinfected children: the significance of genotype-guided dosing." J Antimicrob Chemother 74(9): 2698-2706. | Title |
| 148 | AlGharras, A., et al. (2020). "Imaging-Based Surrogate Markers of Epidermal Growth Factor Receptor Mutation in Lung Adenocarcinoma: A Local Perspective." Can Assoc Radiol J 71(2): 208-216. | Title |
| 149 | Alghasham, A. A. and M. C. J. A. o. p. Nahata (2000). "Clinical use of fluoroquinolones in children."  34(3): 347-359. | Title |
| 150 | Al-Hadiya, B. M. H., et al. (2002). Spironolactone. Analytical Profiles of Drug Substances and Excipients. H. G. Brittain, Academic Press. 29: 261-320. | Title |
| 151 | Al-Halafi, A. M. J. O. j. o. o. (2014). "Vascular endothelial growth factor trap-eye and trap technology: Aflibercept from bench to bedside." 7(3): 112-115. | Title |
| 152 | Alharbi, A. E., et al. (2024). "The Effect of Genetic Variants of SLC22A2 (rs662301 and rs315978) on the response to Metformin in type 2 Saudi diabetic patients." Gene 927: 148648. | Title |
| 153 | Al-Harbi, D., et al. (2022). "Linezolid vs vancomycin in induced thrombocytopenia."  11(4): 1649-1660. | Title |
| 154 | Alharbi, H. M., et al. (2024). "A novel zingerone-loaded zinc MOF coated by niosome nanocomposites to enhance antimicrobial properties and apoptosis in breast cancer cells." Materials Today Communications 41: 110245. | Title |
| 155 | Alharbi, H. M., et al. (2024). "Preparation, characterization, and anticancer evaluation of polydatin conjugated with zinc MOF and encapsulated by liponiosomes as a potential nanotool-induce apoptosis." Journal of Molecular Structure 1315: 138982. | Title |
| 156 | Al-Hashel, J. (2019). "Botulinum toxin in migraine: rationale and patient selection." Journal of the neurological sciences 405: 25-26. | Title |
| 157 | Alhashemi, J. A. and M. F. Daghistani (2006). "Effects of intraoperative i.v. acetaminophen vs i.m. meperidine on post-tonsillectomy pain in children." British Journal of Anaesthesia 96(6): 790-795. | Title |

| | | |
|---|---|---|
| 158 | Al-Hassany, L., et al. (2022). "Calcitonin gene-related peptide-targeting drugs for migraine: how pharmacology might inform treatment decisions." Lancet Neurol 21(3): 284-294. | Title |
| 159 | Alhawassi, T. M., et al. (2017). "Prevalence, management and control of hypertension in older adults on admission to hospital." Saudi Pharmaceutical Journal 25(8): 1201-1207. | Title |
| 160 | Alheshibri, M., et al. (2023). "Synthesis of highly stable Ag/Ta2O5 nanocomposite by pulsed laser ablation as an effectual antibacterial agent." Optics & Laser Technology 162: 109295. | Title |
| 161 | Al-Hilal, T. A., et al. (2021). "Design, synthesis and biological evaluations of a long-acting, hypoxia-activated prodrug of fasudil, a ROCK inhibitor, to reduce its systemic side-effects." Journal of Controlled Release 334: 237-247. | Title |
| 162 | Alhudaithi, S. S., et al. (2024). "Sorafenib and Piperine co-loaded PLGA nanoparticles: Development, characterization, and anti-cancer activity against hepatocellular carcinoma cell line." Saudi Pharmaceutical Journal 32(5): 102064. | Title |
| 163 | Al-Humaidi, J. Y., et al. (2024). "Design and synthesis of novel hybrids incorporating thiadiazole or thiazole-naphthalene: Anticancer assessment and molecular docking study." Results in Chemistry 7: 101475. | Title |
| 164 | Alhumaydhi, F. A., et al. (2021). "Probing the interaction of memantine, an important Alzheimer's drug, with human serum albumin: In silico and in vitro approach." Journal of Molecular Liquids 340: 116888. | Title |
| 165 | Ali, A. S., et al. (2012). "Pharmacokinetic approach for optimizing gentamicin use in neonates during the first week of life." 44(1): 36-40. | Title |
| 166 | Ali, I., et al. (2024). "Synthesis of calix (4) resorcinarene based amphiphilic macrocycle as an efficient nanocarrier for Amphotericin-B to enhance its oral bioavailability." Colloids and Surfaces B: Biointerfaces 238: 113918. | Title |
| 167 | Ali, I., et al. (2015). "Heterocyclic scaffolds: centrality in anticancer drug development." 16(7): 711-734. | Title |
| 168 | Ali, M. H., et al. (2019). "Isoniazid acetylation phenotypes in the Sudanese population; findings and implications." Journal of Clinical Tuberculosis and Other Mycobacterial Diseases 17: 100120. | Title |
| 169 | Ali, M. S. and H. A. Al-Lohedan (2017). "Deciphering the interaction of procaine with bovine serum albumin and elucidation of binding site: A multi spectroscopic and molecular docking study." Journal of Molecular Liquids 236: 232-240. | Title |
| 170 | Ali, R., et al. (2017). "Development and characterization of methoxy poly(ethylene oxide)-block-poly(ε-caprolactone) (PEO-b-PCL) micelles as vehicles for the solubilization and delivery of tacrolimus." Saudi Pharmaceutical Journal 25(2): 258-265. | Title |

| | | |
|---|---|---|
| 171 | Ali, R., et al. (2024). "Chrysin-loaded Soluplus-TPGS mixed micelles: Optimization, characterization and anticancer activity against hepatocellular carcinoma cell line." Journal of Drug Delivery Science and Technology 102: 106371. | Title |
| 172 | Ali, R., et al. (2015). "Preliminary results of a phase 2 trial of autologous mesenchymal cell therapy in MS (STREAMS)." Multiple sclerosis (Houndmills, Basingstoke, England) 23(11): 556. | Title |
| 173 | Ali, S. I., et al. (2024). "Rutin encapsulated decellularized earthworm granulation hydrogel promotes angiogenesis in wound healing of diabetic rabbit model by inhibiting TRAF1/NF-κB pathway." Journal of Drug Delivery Science and Technology 97: 105803. | Title |
| 174 | Ali Thorakkattil, S., et al. (2024). "Advancements in ambulatory care pharmacy practice in Saudi Arabia: A comprehensive review of innovations and best practices at Johns Hopkins Aramco Healthcare." Saudi Pharmaceutical Journal 32(10): 102170. | Title |
| 175 | Alipour, M., et al. (2009). "Activity and interactions of liposomal antibiotics in presence of polyanions and sputum of patients with cystic fibrosis." 4(5): e5724. | Title |
| 176 | Ali-Shtayeh, M. S., et al. (2013). "Complementary and alternative medicine (CAM) use among hypertensive patients in Palestine." Complementary Therapies in Clinical Practice 19(4): 256-263. | Title |
| 177 | Ali-Shtayeh, M. S., et al. (2016). "Complementary and alternative medicine use among cancer patients in Palestine with special reference to safety-related concerns." Journal of Ethnopharmacology 187: 104-122. | Title |
| 178 | Al-Jaid, M., et al. "Parental knowledge of RSV infection and attitude to infant immunization with monoclonal antibodies in western region, Saudi Arabia." | Title |
| 179 | Al-Jamea, L. H., et al. (2021). "Genetic analysis of TMPRSS6 gene in Saudi female patients with iron deficiency anemia." Hematol Oncol Stem Cell Ther 14(1): 41-50. | Title |
| 180 | Aljehani, M. N., et al. (2022). "Association Between Body Mass Index and Response to Disease-Modifying Therapies in Patients With Relapsing-Remitting Multiple Sclerosis at King Abdulaziz University Hospital: A Retrospective Study." Cureus 14(12): e32695. | Title |
| 181 | Aljimaee, Y. H. (2015). "FORMULATION AND EVALUATION OF CARVEDILOL ORODISPERSIBLE TABLETS." | Title |
| 182 | Aljohani, A., et al. (2024). "Impact of Obesity on Echinocandin Effectiveness in Treating Candida Infections: A Retrospective Observational Cohort Study." 2863-2871. | Title |
| 183 | Aljubran, A., et al. (2019). "Efficacy of regorafenib in metastatic colorectal cancer: a multi-institutional retrospective study." 13: 1179554918825447. | Title |

| | | |
|---|---|---|
| 184 | Aljutayli, A., et al. (2021). "3 Article II: An Update on Population Pharmacokinetic Analyses of Vancomycin, Part II: in Pediatric Patients." 56. | Title |
| 185 | Aljutayli, A., et al. (2020). "An update on population pharmacokinetic analyses of vancomycin, part I: in adults." 59: 671-698. | Title |
| 186 | Aljutayli, A., et al. (2021). "5 Article IV Critical Assessment of Vancomycin Monitoring Methods of the Revised Guidelines." 153. | Title |
| 187 | Aljutayli, A., et al. (2022). "Critical assessment of the revised guidelines for vancomycin therapeutic drug monitoring." Biomedicine & Pharmacotherapy 155: 113777. | Title |
| 188 | Alkabab, Y. M., et al. (2018). "Performance of computed tomography versus chest radiography in patients with pulmonary tuberculosis with and without diabetes at a tertiary hospital in Riyadh, Saudi Arabia." 37-43. | Title |
| 189 | Alkahtanib, H. M. and B. T. AlQuadeiba "Fulwah Yahya Alqahtania, Fadilah Sfouq Aleanizya, Eram El Tahira." | Language |
| 190 | Alkaltham, M. F., et al. (2024). "Activity against Mycobacterium tuberculosis of a new class of spirooxindolopyrrolidine embedded chromanone hybrid heterocycles††Electronic supplementary information (ESI) available. CCDC 2126482 (5a) and 2126502 (5g). For ESI and crystallographic data in CIF or other electronic format see DOI: https://doi.org/10.1039/d4ra01501k." RSC Advances 14(17): 11604-11613. | Title |
| 191 | Al-Karmalawy, A. A., et al. (2023). "Novel fused imidazotriazines acting as promising top. II inhibitors and apoptotic inducers with greater selectivity against head and neck tumors: Design, synthesis, and biological assessments." European Journal of Medicinal Chemistry 259: 115661. | Title |
| 192 | Alkazmi, L., et al. (2022). "Roxadustat for SARS-CoV-2 infection: old signaling raised new hopes." 22(3): 183-186. | Title |
| 193 | Alkharfy, K. M., et al. (2017). "Prevalence of UDP-glucuronosyltransferase polymorphisms (UGT1A6*2, 1A7*12, 1A8*3, 1A9*3, 2B7*2, and 2B15*2) in a Saudi population." Saudi Pharmaceutical Journal 25(2): 224-230. | Title |
| 194 | Alkholief, M., et al. (2020). "Thermoresponsive sol-gel improves ocular bioavailability of Dipivefrin hydrochloride and potentially reduces the elevated intraocular pressure in vivo." Saudi Pharmaceutical Journal 28(8): 1019-1029. | Title |
| 195 | Al-Kofide, H., et al. (2010). "Pharmacokinetics of vancomycin in adult cancer patients." J Oncol Pharm Pract 16(4): 245-250. | Title |
| 196 | Alla, D., et al. (2024). "Safety of Proton Pump Inhibitors in Pediatric Population: A Systematic Review." 11: 2333794X241248967. | Title |
| 197 | Allehdan, S., et al. (2021). "Gestational diabetes mellitus management: diet and lifestyle." 51(2): 300-322. | Title |
| 198 | Allen, J. M., et al. (2023). "Impact of piperacillin-tazobactam dosing in septic shock patients using real-world evidence: an observational, retrospective cohort study." 57(6): 653-661. | Title |

| | | |
|---|---|---|
| 199 | ALLENDE-BANDRÉS, M. D. L. Á., et al. (2024). "Population pharmacokinetics analyses and therapeutic drug monitoring of vancomycin: a scoping review." 14: 1. | Title |
| 200 | Al-Mahdawi, R. A. and H. A. J. A.-R. J. o. M. S. Al-Jumaily (2023). "Analgesic Efficacy of Bupivacaine as an Infiltration Injection Technique after Extraction of Impacted Mandibular Third Molars: A Randomized Controlled Study." 5: 166-171. | Title |
| 201 | Almalag, H., et al. (2020). "Risk factors associated with methotrexate intolerance in rheumatoid arthritis patients." 193-202. | Title |
| 202 | Almalag, H. M., et al. (2018). "The impact of old versus new antiepileptic drugs on costs and patient reported outcomes among older adults." Geriatric Nursing 39(6): 669-675. | Title |
| 203 | Almalag, H. M., et al. (2018). "The impact of old versus new antiepileptic drugs on costs and patient reported outcomes among older adults." Geriatric Nursing 39(6): 669-675. | Title |
| 204 | Almalki, A. H., et al. (2024). "A Box-Behnken response surface methodology for optimizing fluorescent detection of cenobamate using graphene quantum dots: Environmental impact assessment and pharmacokinetic applications." Microchemical Journal 205: 111381. | Title |
| 205 | Almalki, B., et al. (2021). "Evaluation of rejection, infection, and malignancy outcomes in elderly liver transplant recipients receiving a similar level of immunosuppression compared to a younger group." Transplant Immunology 69: 101485. | Title |
| 206 | Almalki, W. H. (2024). "An Up-to-date Review on Protein-based Nanocarriers in the Management of Cancer." Curr Drug Deliv 21(4): 509-524. | Title |
| 207 | Almangour, T. A. and M. A. Alrasheed (2024). "Dalbavancin for the treatment of bone and joint infections: A meta-analysis." Journal of Infection and Chemotherapy. | Title |
| 208 | Almangour, T. A., et al. (2019). "Dalbavancin for the management of gram-positive osteomyelitis: Effectiveness and potential utility." Diagn Microbiol Infect Dis 93(3): 213-218. | Title |
| 209 | Almansour, H. A., et al. (2017). "Pharmacists' perspectives about their role in care of patients with diabetes observing Ramadan." Research in Social and Administrative Pharmacy 13(1): 109-122. | Title |
| 210 | Almeida, R., et al. (2011). "Efficacy of back school in Brazil." Physiotherapy (united kingdom) 97: eS59-eS60. | Title |
| 211 | Al-Mir, H., et al. (2021). "Spread of ESC-, carbapenem-and colistin-resistant Escherichia coli clones and plasmids within and between food workers in Lebanon." 76(12): 3135-3143. | Title |
| 212 | Almodibeg, B. and P. Forget (2024). "Challenges of acute pain management in older patients." Age Ageing 53(4). | Title |

| | | |
|---|---|---|
| 213 | Almohaish, S., et al. (2023). "Personalized antiseizure medication therapy in critically ill adult patients." Pharmacotherapy 43(11): 1166-1181. | Title |
| 214 | Almohammde, S., et al. (2021). "A survey of therapeutic drug monitoring in a teaching hospital." Saudi Journal of Biological Sciences 28(1): 744-747. | Title |
| 215 | Almoosa, Z., et al. (2017). "Invasive candidiasis in pediatric patients at king Fahad Medical City in Central Saudi Arabia: a 5-year retrospective study." 38(11): 1118. | Title |
| 216 | Almoslem, M. J. A. (2023). Pharmacokinetic and Pharmacodynamic Modeling for Optimizing Combination Therapy, University of Florida. | Title |
| 217 | Almowallad, S. J. and L. S. Alqahtani (2024). "Synergistic antimicrobial action of chitosan-neem extracts nanoformulation as a promising strategy for overcoming multi-drug resistant bacteria." International Journal of Biological Macromolecules 272: 132337. | Title |
| 218 | Al-Mssallem, M. Q., et al. (2011). "A study of Hassawi rice (Oryza sativa L.) in terms of its carbohydrate hydrolysis (in vitro) and glycaemic and insulinaemic indices (in vivo)." European journal of clinical nutrition 65(5): 627-634. | Title |
| 219 | Almufarriji, F. M., et al. (2024). "Unveiling the multitargeted potential of deprodone and control comparison with linezolid against hydrolase and transferase enzymes of methicillin-resistant Staphylococcus aureus." International Journal of Biological Macromolecules 279: 135459. | Title |
| 220 | Al-Muhsen, S., et al. (2022). "Favipiravir effectiveness and safety in hospitalized moderate-severe COVID-19 patients: observational prospective multicenter investigation in Saudi Arabia." 9: 826247. | Title |
| 221 | Almuqbil, M., et al. (2022). "The role of drug information centers to improve medication safety in Saudi Arabia - a study from healthcare professionals' perspective." Saudi Pharmaceutical Journal 30(4): 377-381. | Title |
| 222 | Almuqbil, M., et al. (2023). "Comparison of medical documentation between pharmacist-led anticoagulation clinics and physician-led anticoagulation clinics: A retrospective study." Saudi Pharmaceutical Journal 31(11): 101795. | Title |
| 223 | Almutairy, R., et al. (2020). "Impact of colistin dosing on the incidence of nephrotoxicity in a tertiary care hospital in Saudi Arabia." 9(8): 485. | Title |
| 224 | Alnaim, L. S., et al. (2022). "The prevalence of drug–drug interactions in cancer therapy and the clinical outcomes." Life Sciences 310: 121071. | Title |
| 225 | Alnasser, A., et al. (2023). "Applicable pharmacokinetic study: Development and validation of bioanalytical LC-MS/MS method for the simultaneous quantification of cytarabine and glasdegib used for the treatment of acute myeloid leukemia." Arabian Journal of Chemistry 16(10): 105117. | Title |

| | | |
|---|---|---|
| 226 | Alnezary, F. S., et al. (2023). "The Significance of Bayesian Pharmacokinetics in Dosing for Critically Ill Patients: A Primer for Clinicians Using Vancomycin as an Example." Antibiotics (Basel) 12(9). | Title |
| 227 | Alnezary, F. S., et al. (2024). "Evaluating preceptorship during advanced pharmacy practice experiences in Saudi Arabia: A Cross-Sectional Study." Saudi Pharmaceutical Journal 32(3): 101970. | Title |
| 228 | Alnoman, M. M., et al. (2024). "New Co(II) Schiff base complexes of 3-ethoxy-4-hydroxybenzaldehyde and chlorophenyl ethylamine derivatives as potent antimicrobial agents: Design, synthesis, molecular docking, DFT calculations, and in silico ADME profiles." Journal of Molecular Structure 1307: 138021. | Title |
| 229 | Alobaid, A. S., et al. (2017). "Population pharmacokinetics of piperacillin in nonobese, obese, and morbidly obese critically ill patients." Antimicrob Agents Chemother 61(3): 10.1128/aac. 01276-01216. | Abstract |
| 230 | Alobaid, A. S., et al. (2016). "Effect of obesity on the population pharmacokinetics of fluconazole in critically ill patients." 60(11): 6550-6557. | Abstract |
| 231 | Alobaid, A. S., et al. (2016). "Effect of obesity on the population pharmacokinetics of meropenem in critically ill patients." 60(8): 4577-4584. | Abstract |
| 232 | Alodhaibi, I., et al. (2024). "An Open-Label Phase I Study of Metformin and Nelfinavir in Combination With Bortezomib in Patients With Relapsed and Refractory Multiple Myeloma." Clinical Lymphoma Myeloma and Leukemia 24(5): 298-304. | Title |
| 233 | Aloliqi, A. A. (2024). "Towards identification of therapeutics against multi-infections and cancers causing Propionibacterium acnes: Molecular modeling and dynamics simulation investigation." Journal of Molecular Liquids 415: 126373. | Title |
| 234 | Alomi, Y. A. and H. Y. Almudaiheem (2017). "Clinical outcomes of vancomycin therapeutic monitoring services at ministry of health hospital in Saudi Arabia." Value in health 20(5): A42. | Title |
| 235 | Al-Omran, A. M., et al. (2020). "Linezolid Add-On Rescue Therapy Cured MRSA Necrotizing Pneumonia: a case report in a preterm infant." | Title |
| 236 | Alosaimi, A. A. M., et al. (2022). "Perceived risks of over-the-counter medication use among pregnant Saudi mothers: A cross-sectional study." Journal of Taibah University Medical Sciences 17(5): 755-764. | Title |
| 237 | Alotaibi, S., et al. (2024). "Efficacy of Ciprofloxacin in Treating Gram-Negative Infections: Does Obesity Matter?" 12(5): 147. | Title |
| 238 | Al-Qadheeb, N. S., et al. (2012). "The First International Residency Program Accredited by the American Society of Health-System Pharmacists." American Journal of Pharmaceutical Education 76(10): 190. | Title |
| 239 | Alqadi, K., et al. (2020). "Effects of fasting during Ramadan on seizure control and quality of life in patients with epilepsy." Epilepsy & Behavior 112: 107440. | Title |

| | | |
|---|---|---|
| 240 | AlQahtani, A. A. (2020). Chemosensitizing and Cardioprotective Effects of Marine Astaxanthin in Doxorubicin Treated Animals, KING ABDULAZIZ UNIVERSITY JEDDAH. | Title |
| 241 | Alqahtani, A. A. H. "Chemosensitizing and Nephroprotective Effect of Dimethyl Sulfoxide in Cisplatin Treated Animals." | Title |
| 242 | Alqahtani, F., et al. (2023). "A Physiologically Based Pharmacokinetic Model to Predict Systemic Ondansetron Concentration in Liver Cirrhosis Patients." Pharmaceuticals (Basel) 16(12). | Title |
| 243 | Alqahtani, F., et al. (2023). "Predicting Hydroxychloroquine Clearance in Healthy and Diseased Populations Using a Physiologically Based Pharmacokinetic Approach." Pharmaceutics 15(4). | Title |
| 244 | Alqahtani, N., et al. (2017). "Clinicians attitudes and concerns toward pioglitazone-associated bladder malignancies." Pharmacoepidemiology and drug safety 26: 602-603. | Title |
| 245 | Alqahtani, S., et al. (2018). "Optimizing amikacin dosage in pediatrics based on population pharmacokinetic/pharmacodynamic modeling." 20: 265-272. | Title |
| 246 | Alqahtani, S., et al. (2020). "Estimation of lithium clearance in patients with bipolar disorder." Int Clin Psychopharmacol 35(3): 157-162. | Title |
| 247 | Alqahtani, S., et al. (2023). "Population Pharmacokinetics of Rivaroxaban in Real-World Patients." J Clin Pharmacol 63(8): 943-949. | Abstract |
| 248 | Alqahtani, S., et al. (2022). "Clinical pharmacokinetics of capecitabine and its metabolites in colorectal cancer patients." Saudi Pharmaceutical Journal 30(5): 527-531. | Abstract |
| 249 | Alqahtani, S., et al. (2019). "Estimation of Phenytoin Pharmacokinetic Parameters in Saudi Epileptic Patients." Pharmacology 104(1-2): 60-66. | Full text reading |
| 250 | Alqasmi, M. J. J. o. C. M. (2024). "Therapeutic Interventions for Pseudomonas Infections in Cystic Fibrosis Patients: A Review of Phase IV Trials." 13(21): 6530. | Title |
| 251 | Al-Quadeib, B. T., et al. (2014). "Therapeutic monitoring of amphotericin B in Saudi ICU patients using UPLC MS/MS assay." Biomed Chromatogr 28(12): 1652-1659. | Title |
| 252 | Al-Qubaisey, M. I., et al. (2023). "Evaluation of Drug Release Profiles of Titanium Plates Coated with PLGA or Chitosan with Meropenem Using UPLC: An In Vitro Study." 13(15): 8995. | Title |
| 253 | Al-Qurain, A. A., et al. (2021). "Population pharmacokinetic model of subcutaneous fentanyl in older acute care patients." Eur J Clin Pharmacol 77(9): 1357-1368. | Title |

| | | |
|---|---|---|
| 254 | Al-Qurain, A. A., et al. (2022). "Population Pharmacokinetic Model for Tramadol and O-desmethyltramadol in Older Patients." Eur J Drug Metab Pharmacokinet 47(3): 387-402. | Abstract |
| 255 | Al-Qurain, A. A., et al. (2021). "Simultaneous LC-MS/MS quantification of oxycodone, tramadol and fentanyl and their metabolites (noroxycodone, oxymorphone, O- desmethyltramadol, N- desmethyltramadol, and norfentanyl) in human plasma and whole blood collected via venepuncture and volumetric absorptive micro sampling." J Pharm Biomed Anal 203: 114171. | Title |
| 256 | Alquraini, A. J. T. J. o. P. R. (2023). "Dostarlimab: Novel paradigm shift in cancer therapy."  22(9): 2009-2015. | Title |
| 257 | AlRabiah, H., et al. (2019). "Automated flow fluorescent noncompetitive immunoassay for measurement of human plasma levels of monoclonal antibodies used for immunotherapy of cancers with KinExA™ 3200 biosensor."  192: 331-338. | Title |
| 258 | Alrabiah, Z., et al. (2019). "Evaluation of community pharmacists' knowledge about drug–drug interaction in Central Saudi Arabia." Saudi Pharmaceutical Journal 27(4): 463-466. | Title |
| 259 | Alrajeh, K., et al. (2023). "The frequency of major ABCG2, SLCO1B1 and CYP2C9 variants in Asian, Native Hawaiian and Pacific Islander women subgroups: implications for personalized statins dosing." Pharmacogenomics 24(7): 381-398. | Title |
| 260 | Statins are medications used to lower low-density lipoprotein ('bad') cholesterol. Variation in genes for proteins which transport drugs (SLCO1B1 and ABCG2) or metabolize drugs (CYP2C9) may significantly influence how much statin someone is exposed to. Genetic variants within SLCO1B1 can affect exposure to all statins, while variants within ABCG2 and CYP2C9 can affect exposure to rosuvastatin and fluvastatin, respectively. The prevalence of the decreased or no-function genetic variants is unknown among Filipino and Native Hawaiian and Pacific Islander (NHPI) subgroups. The major racial categorization of 'Asians and NHPI' (ANHPI) can miss potential genetic and ancestral differences among population subgroups. Our study used biobank data from 1064 women of ANHPI descent to estimate the frequencies of four important variants within SLCO1B1, ABCG2 and CYP2C9. Those of ANHPI ancestry were less likely to have variations in SLCO1B1 and CYP2C9 but significantly more likely to have nonfunctional ABCG2 than Europeans. Our findings provide insight into SLCO1B1 and CYP2C9 genetic variations among under-represented subgroups. Specifically, Filipinos and Koreans have the highest rates of higher risk genetic variants linked to high rosuvastatin and fluvastatin exposure and muscle-related side effects. Estimating the frequency of genetic variations in under-represented subgroups is pivotal in reducing health disparities in treatment outcomes, diversifying pharmacogenetic research and advancing personalized medicine. | Title |

| | | |
|---|---|---|
| 261 | Alrajeh, K. and Y. M. J. P. Roman (2023). "The frequency of rs2231142 in ABCG2 among Asian subgroups: implications for personalized rosuvastatin dosing." 24(1): 15-26. | Title |
| 262 | Al-Ramahi, R., et al. (2017). "Medication dosing errors and associated factors in hospitalized pediatric patients from the South Area of the West Bank - Palestine." Saudi Pharmaceutical Journal 25(6): 857-860. | Title |
| 263 | Alrashidi, A. A., et al. (2024). "Synergistic strategies for enhanced liver cancer therapy with sorafenib/resveratrol PEGylated liposomes in vitro and in vivo." Journal of Drug Delivery Science and Technology 96: 105703. | Title |
| 264 | Al-Riyami, I., et al. (2022). "Impact of glutathione S-transferase polymorphisms on busulfan pharmacokinetics and outcomes of hematopoietic stem cell transplantation." 44(4): 527-534. | Abstract |
| 265 | Alrubia, S., et al. (2022). "Quantitative Assessment of the Impact of Crohn's Disease on Protein Abundance of Human Intestinal Drug-Metabolising Enzymes and Transporters." Journal of Pharmaceutical Sciences 111(10): 2917-2929. | Title |
| 266 | Alrubia, S., et al. (2022). "Altered Bioavailability and Pharmacokinetics in Crohn's Disease: Capturing Systems Parameters for PBPK to Assist with Predicting the Fate of Orally Administered Drugs." Clin Pharmacokinet 61(10): 1365-1392. | Title |
| 267 | AlRuthia, Y., et al. (2019). "Drug-drug interactions and pharmacists' interventions among psychiatric patients in outpatient clinics of a teaching hospital in Saudi Arabia." Saudi Pharmaceutical Journal 27(6): 798-802. | Title |
| 268 | Al-Saadi, M. M. J. P. j. o. m. s. (2007). "The clinical utility of montelukast in paediatric respiratory diseases." 23(6): 962. | Title |
| 269 | Alsaedi, S. A. (2003). "Once daily gentamicin dosing in full term neonates." Saudi medical journal 24(9): 978-981. | Title |
| 270 | Alsaffar, R. M., et al. "Akt is the collective name of a set of three serine/threonine-specific protein kinases that play key roles in multiple cellular processes such as glucose metabolism, apoptosis, cell proliferation, transcription, and cell migration. Menu." | Title |
| 271 | Al-Said, M. S., et al. (2000). "Bioequivalence evaluation of two brands of cefuroxime 500 mg tablets (Cefuzime and Zinnat) in healthy human volunteers." Biopharmaceutics & drug disposition 21(6): 205-210. | Title |
| 272 | Alsaif, A. A., et al. (2013). "Association of multiple drug resistance-1 gene polymorphism with multiple drug resistance in breast cancer patients from an ethnic Saudi Arabian population." Cancer Epidemiol 37(5): 762-766. | Title |
| 273 | Alsaif, G., et al. (2024). "Network pharmacology and molecular docking analysis of Catharanthus roseus compounds: Implications for non-small cell lung cancer treatment." Journal of King Saud University - Science 36(4): 103134. | Title |

| | | |
|---|---|---|
| 274 | Alsarra, I. A. J. S. P. J. (2004). "Development of a stability-indicating HPLC method for the determination of montelukast in tablets and human plasma and its applications to pharmacokinetic and stability studies." 12(4): 136-143. | Title |
| 275 | Alshabeeb, M. A., et al. (2022). "Prevalence of exposure to pharmacogenetic drugs by the Saudis treated at the health care centers of the Ministry of National Guard." Saudi Pharmaceutical Journal 30(8): 1181-1192. | Title |
| 276 | Alshadfan, H., et al. "A Comparison between Metformin Immediate-release and Extended-release: A Review." | Title |
| 277 | Al-Shaer, M. H., et al. (2019). "Fluoroquinolones in drug-resistant tuberculosis: culture conversion and pharmacokinetic/pharmacodynamic target attainment to guide dose selection." Antimicrob Agents Chemother 63(7): 10.1128/aac. 00279-00219. | Title |
| 278 | Al-Shaer, M. H., et al. (2020). "Ethionamide population pharmacokinetic model and target attainment in multidrug-resistant tuberculosis." Antimicrob Agents Chemother 64(9): 10.1128/aac. 00713-00720. | Abstract |
| 279 | Alshami, I. and A. E. Alharbi (2014). "Hibiscus sabdariffa extract inhibits in vitro biofilm formation capacity of Candida albicans isolated from recurrent urinary tract infections." Asian Pacific Journal of Tropical Biomedicine 4(2): 104-108. | Title |
| 280 | Alshammari, E. (2019). "Pharmacokinetic and Pharmacodynamic Modelling in Paediatric Bone Marrow Transplantation." | Title |
| 281 | Alshammari, E. (2019). "Semi-mechanistic modelling of neutropenia." | Title |
| 282 | Alshammari, M. K., et al. (2023). "A systematic review of clinical pharmacokinetics of inhaled antiviral." 59(4): 642. | Title |
| 283 | Alshammari, T. M. (2016). "Drug safety: The concept, inception and its importance in patients' health." Saudi Pharm J 24(4): 405-412. | Title |
| 284 | Alshammari, T. M., et al. (2017). "Comparison of the safety information on drug labels in three developed countries: The USA, UK and Canada." Saudi Pharmaceutical Journal 25(8): 1103-1107. | Title |
| 285 | Alshamsan, A., et al. (2011). "STAT3 Knockdown in B16 Melanoma by siRNA Lipopolyplexes Induces Bystander Immune Response In Vitro and In Vivo." Translational Oncology 4(3): 178-188. | Title |
| 286 | Al-Shamsi, H. O., et al. (2024). "Cancer research in the United Arab Emirates from birth to present: A bibliometric analysis." Heliyon 10(6): e27201. | Title |
| 287 | AlSharari, S. D., et al. (2015). "The role of alpha5 nicotinic acetylcholine receptors in mouse models of chronic inflammatory and neuropathic pain." | Title |
| 288 | Alsharif, N. Z., et al. (2019). "Cultural Sensitivity and Global Pharmacy Engagement in the Arab World." American Journal of Pharmaceutical Education 83(4): 7228. | Title |

| | | |
|---|---|---|
| 289 | Alshatwi, A. A., et al. (2016). "Synergistic anticancer activity of dietary tea polyphenols and bleomycin hydrochloride in human cervical cancer cell: Caspase-dependent and independent apoptotic pathways." Chemico-Biological Interactions 247: 1-10. | Title |
| 290 | Alshaya, A. I. and A. M. J. C. C. M. Alhammad (2024). "How Reliable Are Serum Levetiracetam Levels to Be Used as a Primary Endpoint?" 52(4): e203-e204. | Title |
| 291 | Alshehri, A., et al. (2023). "Population pharmacokinetic model of ivermectin in mass drug administration against lymphatic filariasis." PLoS Negl Trop Dis 17(6): e0011319. | Title |
| 292 | Alshehri, A. F., et al. (2022). "Assessment of Caspofungin use at a Tertiary Teaching Hospital and compliance with IDSA guidelines and FDA labelings." Saudi Pharmaceutical Journal 30(3): 212-216. | Title |
| 293 | Alshehri, S., et al. (2020). "Morphological transition of M. tuberculosis and modulation of intestinal permeation by food grade cationic nanoemulsion: In vitro-ex vivo-in silico GastroPlus™ studies." Journal of Drug Delivery Science and Technology 60: 101971. | Title |
| 294 | Al-Shohaib, S., et al. (2010). "The Hematopoietic Effect of Epotin (Recombinant Human Erythropoietin-α) on Maintenance Hemodialysis End-Stage Kidney Disease Patients." Transplantation Proceedings 42(3): 753-759. | Title |
| 295 | Alshouli, B., et al. (2024). "Evaluation of knowledge and attitude concerning augmented renal clearance among physicians and clinical pharmacists in Al-Ain, UAE: A cross-sectional study." 19(9): e0310081. | Title |
| 296 | Alsifri, S., et al. (2023). "Safety of Empagliflozin in Patients with Type 2 Diabetes Mellitus in Saudi Arabia: A Post-Authorisation Safety Study." 14(3): 129-147. | Title |
| 297 | Al-Sofyani, K. A. J. A. S. P. (2020). "Comparative Analysis of Candida albicans Versus Candida Non-albicans Infection among Pediatric Patients at King Abdulaziz University Hospital." 3: 37-47. | Title |
| 298 | Alsous, M., et al. (2018). "Predictors of nonadherence in children and adolescents with epilepsy: A multimethod assessment approach." 85: 205-211. | Title |
| 299 | Alsowaida, Y. S., et al. (2024). "Evaluation of clinical outcomes of anidulafungin for the treatment of candidemia in hospitalized critically ill patients with obesity: A multicenter, retrospective cohort study." 148: 107234. | Title |
| 300 | Alsowaida, Y. S., et al. (2023). "Echinocandin exposures in obese patients: A scoping review and clinical perspectives." 80(8): 503-517. | Title |
| 301 | Alsowaida, Y. S., et al. (2024). "Evaluation of clinical outcomes of anidulafungin for the treatment of candidemia in hospitalized critically ill patients with obesity: A multicenter, retrospective cohort study." International Journal of Infectious Diseases 148: 107234. | Title |

| | | |
|---|---|---|
| 302 | Alsuhebany, N., et al. (2023). "Zanubrutinib in Mantle Cell Lymphoma Management: A Comprehensive Review." 67-76. | Title |
| 303 | Alsulaimany, F. A., et al. (2022). "Identification of novel mycobacterium tuberculosis leucyl-tRNA synthetase inhibitor using a knowledge-based computational screening approach." Journal of King Saud University - Science 34(4): 102032. | Title |
| 304 | Al-Sulaiti, F. K. (2018). Clinical And Pharmacokinetic Evaluation Of Optimal Monitoring Parameters And Sampling Schemes For Vancomycin Therapeutic Drug Monitoring In Qatar. | Title |
| 305 | Alsultan, A. (2019). "Determining therapeutic trough ranges for linezolid." Saudi Pharmaceutical Journal 27(8): 1061-1063. | Title |
| 306 | Alsultan, A., et al. (2020). "AUC-vs. trough-guided monitoring of vancomycin in infants." 87: 359-364. | Title |
| 307 | Alsultan, A., et al. (2018). "Optimizing Vancomycin Monitoring in Pediatric Patients." Pediatr Infect Dis J 37(9): 880-885. | Abstract |
| 308 | Alsultan, A., et al. (2019). "Optimizing Gentamicin Dosing in Pediatrics Using Monte Carlo Simulations." Pediatr Infect Dis J 38(4): 390-395. | Abstract |
| 309 | Alsultan, A., et al. (2023). "Population pharmacokinetics of vancomycin in very low birth weight neonates." 11: 1093171. | Full text reading |
| 310 | Alsultan, A., et al. (2023). "Interethnic differences in drug response: projected impact of genetic variations in the Saudi population." 24(12): 685-696. | Abstract |
| 311 | Alsultan, A., et al. (2022). "Can first-dose therapeutic drug monitoring predict the steady state area under the blood concentration-time curve of busulfan in pediatric patients undergoing hematopoietic stem cell transplantation?" 10: 834773. | Title |
| 312 | Alsultan, A., et al. (2024). "Evaluation of pharmacokinetic pharmacodynamic target attainment of meropenem in pediatric patients." Pediatrics & Neonatology 65(4): 386-390. | Abstract |
| 313 | Alsultan, A., et al. (2020). "Clinical pharmacology applications in clinical drug development and clinical care: a focus on Saudi Arabia." 28(10): 1217-1227. | Title |
| 314 | Alsultan, A., et al. (2024). "Evaluation of the predictive performance of an online voriconazole dose calculator in children." Eur J Clin Pharmacol 80(12): 1989-1993. | Title |
| 315 | Alsultan, A., et al. (2021). "Pharmacokinetics of meropenem in critically ill patients in Saudi Arabia." Saudi Pharmaceutical Journal 29(11): 1272-1277. | Full text reading |
| 316 | Alsultan, A., et al. (2024). "External Validation of Obese/Critically Ill Vancomycin Population Pharmacokinetic Models in Critically Ill Patients Who Are Obese." J Clin Pharmacol 64(3): 353-361. | Abstract |

| | | |
|---|---|---|
| 317 | Alsultan, A., et al. (2017). "Population pharmacokinetics of AZD-5847 in adults with pulmonary tuberculosis." Antimicrob Agents Chemother 61(10): 10.1128/aac. 01066-01017. | Abstract |
| 318 | Alsultan, A., et al. (2018).,"Population Pharmacokinetics of AZD-5847 in Adults with Pulmonary Tuberculosis"." 62(1): 10.1128/aac. 02229-02217. | Abstract |
| 319 | Alsultan, A., et al. (2020). "Pharmacokinetics of Anti-tuberculous Drugs Delivered Via Nasogastric Tube Feeding in a Critically Ill Patient: A Case Study." 40: 675-678. | Title |
| 320 | Alsultan, A., et al. (2017). "Population pharmacokinetics of pyrazinamide in patients with tuberculosis." Antimicrob Agents Chemother 61(6): 10.1128/aac. 02625-02616. | Abstract |
| 321 | Alsultan, A. J. S. P. J. (2019). "Determining therapeutic trough ranges for linezolid." 27(8): 1061-1063. | Title |
| 322 | Alsultan, M. S., et al. (2012). "Hospital pharmacy practice in Saudi Arabia: Prescribing and transcribing in the Riyadh region." Saudi Pharmaceutical Journal 20(3): 203-210. | Title |
| 323 | Altarawneh, H., et al. (2024). "Synergistic bactericidal activity of a novel dual β-lactam combination against methicillin-resistant Staphylococcus aureus." dkae165. | Title |
| 324 | AlTawari, A., et al. (2024). "Nusinersen Treatment for Spinal Muscular Atrophy: Retrospective Multicenter Study of Pediatric and Adult Patients in Kuwait." 16(3): 631-642. | Title |
| 325 | Al-Thawabieh, W., et al. (2024). "Tropicamide Versus Cyclopentolate for Cycloplegic Refraction in Pediatric Patients With Brown Irides: A Randomized Clinical Trial." American Journal of Ophthalmology 257: 218-226. | Title |
| 326 | Althof, S. E., et al. (2010). "International Society for Sexual Medicine's Guidelines for the Diagnosis and Treatment of Premature Ejaculation." The Journal of Sexual Medicine 7(9): 2947-2969. | Title |
| 327 | Al-Thubiani, W. S. A. (2021). The potential protective role of betanin and allicin against adriamycin induced cardiotoxicity in rats, KING ABDULAZIZ UNIVERSITY JEDDAH. | Animal |
| 328 | Alvarado, A. T., et al. (2023). "SLCO1B1 and CYP3A4 allelic variants associated with pharmacokinetic interactions and adverse reactions induced by simvastatin and atorvastatin used in Peru: Clinical implications." 11(6): 934-952. | Title |
| 329 | Álvarez García, F. J., et al. (2024). "Immunisation schedule of the Spanish Association of Pediatrics: 2024 recommendations." Anales de Pediatría (English Edition) 100(1): 34-45. | Title |

| | | |
|---|---|---|
| 330 | Alvarez, N., et al. (1998). "Use of antiepileptic drugs in the treatment of epilepsy in people with intellectual disability." J Intellect Disabil Res 42 Suppl 1: 1-15. | Title |
| 331 | Alwhaibi, A., et al. (2021). "Pharmacokinetic profile of sildenafil citrate in healthy Middle Eastern Males: Comparison with other ethnicities." Saudi Pharmaceutical Journal 29(12): 1498-1505. | Abstract |
| 332 | Al-Worafi, Y. M. (2020). Safety of medications in special population. Drug safety in developing countries, Elsevier: 143-162. | Title |
| 333 | Aly, S., et al. (2016). "Efficacy, costs and quality of life in the real world setting for patients with multiple sclerosis treated with fingolimod: intermediate results from the virgile study." Value in health 19(7): A430. | Title |
| 334 | Alzahrani, A. H. M. (2016). Enhancement of Efficacy and Reduced Toxicity of Cisplatin through Self Nanoemulsifying Drug Delivery System, KING ABDULAZIZ UNIVERSITY JEDDAH. | Title |
| 335 | Alzahrani, A. M., et al. (2023). "Altered pharmacokinetics parameters of vancomycin in patients with hematological malignancy with febrile neutropenia, a Bayesian software estimation." 12(6): 979. | Title |
| 336 | Alzahrani, A. M., et al. (2021). "Causes of vancomycin dosing error; problem detection and practical solutions; a retrospective, single-center, cross-sectional study." Saudi Pharmaceutical Journal 29(6): 616-624. | Title |
| 337 | Alzain, A. A., et al. (2021). "Bioinspired imidazo[1,2-a:4,5-c′]dipyridines with dual antiproliferative and anti-migrative properties in human cancer cells: The SAR investigation." European Journal of Medicinal Chemistry 218: 113258. | Title |
| 338 | Al-Zubairy, S. A. J. I. and D. Resistance (2023). "Microbiologic Cure with a Simplified Dosage of Intravenous Colistin in Adults: A Retrospective Cohort Study." 4237-4249. | Title |
| 339 | Amano, K., et al. (2013). "Long-term safety and efficacy of treatment with subcutaneous abatacept in japanese patients with RA who were MTX inadequate responders-76-week results." Ann Rheum Dis 72. | Title |
| 340 | Amaro-Álvarez, L., et al. (2024). "Exploring the impact of pharmacogenetics on personalized medicine: A systematic review." | Title |
| 341 | Ament, J. D., et al. (2013). "Development of a novel quality of life utility index using NDI and VAS in patients with multi-level cervical spondylosis." Clinical neurosurgery 60: 157. | Title |
| 342 | Amer, M., et al. (2021). "Adjunct low-dose ketamine infusion in critically ill patients at a Saudi Hospital (Attainment trial)." Critical care medicine 49(1 SUPPL 1): 346. | Title |

| | | |
|---|---|---|
| 343 | Amina, R., et al. (2024). "Ethnopharmacological survey of the therapeutic use of camel urine in the Guelmim-Oued Noun and Laayoune-Sakia El Hamra regions of Morocco." Scientific African 26: e02377. | Title |
| 344 | Amir, M., et al. (2023). "In vitro, molecular docking and in silico/ADMET study of cuminaldehyde against Candida, MDR bacteria and human colorectal and cervical carcinoma." South African Journal of Botany 163: 497-510. | Title |
| 345 | Amir, M., et al. (2023). "DRUG USE RECOMMENDATIONS FOR COLISTIN INJECTION IN THE ADULT POPULATION." 35(4). | Title |
| 346 | Ammari, M. A., et al. (2023). "Targeted next-generation sequencing of genes involved in Warfarin Pharmacodynamics and pharmacokinetics pathways using the Saudi Warfarin Pharmacogenetic study (SWAP)." Pharmacogenomics J 23(4): 82-88. | Title |
| 347 | Amritkar, A. M., et al. (2024). "Potentially active aspirin derivative to release nitric oxide: In-vitro, in-vivo and in-silico approaches." Saudi Pharmaceutical Journal 32(3): 101925. | Title |
| 348 | Anbarserry, D., et al. (2023). "The use of therapeutic drug monitoring for early identification of vedolizumab response in Saudi Arabian patients with inflammatory bowel disease." 13(1): 1771. | Title |
| 349 | Anbarserry, D. O. (2023). Pharmacogenomics Of Vedolizumab in Inflammatory Bowel Disease, King Abdulaziz University. | Title |
| 350 | Andersson, T. (2012). "Update on revascularisation results: thrombolysis vs. mechanical thrombectomy." Cardiovascular and interventional radiology 35: S35-S37. | Title |
| 351 | Andrade, F., et al. (2024). "Endophytic fungi-assisted biomass synthesis of eco-friendly formulated silver nanoparticles for enhanced antibacterial, antioxidant, and antidiabetic activities." Journal of Drug Delivery Science and Technology 97: 105749. | Title |
| 352 | Anita, C., et al. (2021). "Topical nanocarriers for management of Rheumatoid Arthritis: A review." Biomed Pharmacother 141: 111880. | Title |
| 353 | Ansar, R., et al. (2022). "Challenges and recent trends with the development of hydrogel fiber for biomedical applications." Chemosphere 287: 131956. | Title |
| 354 | Ansari, J., et al. (2018). "Efficacy of Nivolumab in a Patient with Metastatic Renal Cell Carcinoma and End-Stage Renal Disease on Dialysis: Case Report and Literature Review." Case Reports Immunol 2018: 1623957. | Title |
| 355 | Ansari, M. S., et al. (2024). "BBD assisted in-situ nanoliposomes of esculin hydrate via intranasal delivery for the amelioration of Parkinson's disease." Journal of Drug Delivery Science and Technology 96: 105658. | Title |
| 356 | Anwar, F., et al. (2021). "Targeting COVID-19 in Parkinson's Patients: Drugs Repurposed." Curr Med Chem 28(12): 2392-2408. | Title |

| | | |
|---|---|---|
| 357 | Anwar, S., et al. (2022). "Inhibition of PDK3 by artemisinin, a repurposed antimalarial drug in cancer therapy." Journal of Molecular Liquids 355: 118928. | Title |
| 358 | Anwar, Z., et al. (2019). "Metformin versus Insulin Treatment in Gestational Diabetes Mellitus: their effects on neonates and women in 24 months' follow-up." 13(4): 1348-1351. | Title |
| 359 | Aqil, M., et al. (2016). "Development of clove oil based nanoemulsion of olmesartan for transdermal delivery: Box–Behnken design optimization and pharmacokinetic evaluation." Journal of Molecular Liquids 214: 238-248. | Title |
| 360 | Arafa, M. H., et al. (2020). "Rho-Kinase inhibitors ameliorate diclofenac-induced cardiotoxicity in chloroquine-treated adjuvant arthritic rats." Life Sciences 254: 117605. | Title |
| 361 | Arafah, A. M., et al. (2018). "Pantoprazole reduces vascular relaxation in-vitro and ex-vivo and interferes with blood coagulation in an animal model." Biomedicine & Pharmacotherapy 104: 537-541. | Title |
| 362 | Arfat, Y., et al. (2023). "In silico designing of multiepitope-based-peptide (MBP) vaccine against MAPK protein express for Alzheimer's disease in Zebrafish." Heliyon 9(11): e22204. | Title |
| 363 | Arnaut, A., et al. (2022). "The Role of Morphometric Characteristics of Anterior Maxilla in Planning the Interventions Accompanied by Orthodontic Teeth Movement–An Overview." | Title |
| 364 | Arné, P., et al. (2021). "Aspergillosis in wild birds." 7(3): 241. | Title |
| 365 | Arnold, D., et al. (2013). "Magnetic resonance imaging results from the first year of the ADVANCE study, a pivotal phase 3 trial of peginterferon β-1a in patients with relapsing-remitting multiple sclerosis." Multiple sclerosis (Houndmills, Basingstoke, England) 19(11): 452-453. | Title |
| 366 | Arnold, D. L., et al. (2012). "Effects of BG-12 on magnetic resonance imaging and magnetisation transfer ratio outcomes in relapsing-remitting multiple sclerosis: findings from the phase 3 DEFINE study." Multiple sclerosis (Houndmills, Basingstoke, England) 18(4): 214-215. | Title |
| 367 | Arnold, D. L., et al. (2015). "Reduction in brain volume loss in patients receiving daclizumab HYP versus intramuscular interferon beta-1a: results of the DECIDE study." Multiple sclerosis (Houndmills, Basingstoke, England) 23(11): 257-258. | Title |
| 368 | Arockiaraj, M., et al. (2024). "QSPR analysis of distance-based structural indices for drug compounds in tuberculosis treatment." Heliyon 10(2): e23981. | Title |
| 369 | Aronson, J. K. (2009). Meyler's side effects of antimicrobial drugs, Elsevier. | Title |

| | | |
|---|---|---|
| 370 | Arora, K. K., et al. (2022). "Management of Lateral Epicondylitis: a Prospective Comparative Study Comparing the Local Infiltrations of Leucocyte Enriched Platelet-Rich Plasma (L-aPRP), Glucocorticoid and Normal Saline." Malaysian orthopaedic journal 16(1): 58-69. | Title |
| 371 | Arora, M. K., et al. (2021). "Potential role of nicotinamide analogues against SARS-COV-2 target proteins." Saudi Journal of Biological Sciences 28(12): 7567-7574. | Title |
| 372 | Arslan, E., et al. (2012). Plasmid-mediated quinolone resistance in Escherichia coli isolated from animals, Wiley. | Title |
| 373 | as Pretreatment, P. B. (2003). "US ARMY MEDICAL RESEARCH INSTITUTE OF CHEMICAL DEFENSE." | Title |
| 374 | Asadi, B., et al. (2011). "Neuroprotective effects of erythropoietin in acute ischaemic stroke." Eur J Neurol 18: 101. | Title |
| 375 | Asaduzzaman, S. A. I., et al. (2020). "A comparative study between the severe acute respiratory syndrome–Coronavirus-2, severe acute respiratory syndrome coronavirus, and the Middle East respiratory syndrome coronavirus." 4(Suppl 1): S65-S74. | Title |
| 376 | Asar, T. O., et al. (2024). "Metformin's dual impact on Gut microbiota and cardiovascular health: A comprehensive analysis." Biomedicine & Pharmacotherapy 178: 117128. | Title |
| 377 | Asdaq, S. M. B., et al. (2021). "Use of proton pump inhibitors: An exploration of awareness, attitude and behavior of health care professionals of Riyadh, Saudi Arabia." Saudi Pharmaceutical Journal 29(7): 713-718. | Title |
| 378 | Aseeri, M., et al. (2020). "Experiences of ICU Clinical Pharmacists in Saudi Arabia." (33). | Title |
| 379 | Asghar, B. H., et al. (2023). "Cross-linked quaternized chitosan nanoparticles for effective delivery and controllable release of O. europaea phenolic extract targeting cancer therapy." Journal of Drug Delivery Science and Technology 83: 104388. | Title |
| 380 | Ashina, M., et al. (2021). "Long-term efficacy and safety of erenumab in migraine prevention: results from a 5-year, open-label treatment phase of a randomized clinical trial." Eur J Neurol 28(5): 1716-1725. | Title |
| 381 | Ashkenazi, A., et al. (2006). "Zonisamide for migraine prophylaxis in refractory patients." Cephalalgia 26(10): 1199-1202. | Title |
| 382 | Asif, M., et al. (2024). "An Overview of Various Rifampicin Analogs against Mycobacterium tuberculosis and their Drug Interactions." 20(3): 268-292. | Title |
| 383 | Asiful Islam, M., et al. (2017). "Thrombotic management of antiphospholipid syndrome: towards novel targeted therapies." 15(4): 313-326. | Title |
| 384 | Asiri, Y. A. (1998). Pharmacokinetic modeling of vancomycin in children, pre-adolescent, and adolescent patients: Development, assessment, and application, University of the Pacific. | Title |

| | | |
|---|---|---|
| 385 | Asiri, Y. A. (2011). "Emerging frontiers of pharmacy education in Saudi Arabia: The metamorphosis in the last fifty years." Saudi Pharmaceutical Journal 19(1): 1-8. | Title |
| 386 | Asiri, Y. A., et al. (2005). "Comparative bioavailability study of cefixime (equivalent to 100 mg/5 ml) suspension (Winex vs Suprax) in healthy male volunteers." International journal of clinical pharmacology and therapeutics 43(10): 499-504. | Title |
| 387 | Aspiroz, E. L., et al. (2014). "Toxicogenetics of lopinavir/ritonavir in HIV-infected European patients." 11(3): 263-272. | Title |
| 388 | Asran, A. M., et al. (2023). "Green ecofriendly electrochemical sensing platform for the sensitive determination of doxycycline." Heliyon 9(4): e15223. | Title |
| 389 | Assaggaf, H., et al. (2024). "Exploring the antidiabetic and anti-inflammatory potential of Lavandula officinalis essential oil: In vitro and in silico insights." Heliyon 10(15): e34135. | Title |
| 390 | Assiri, A. and A. Noor (2020). "A computational approach to predict multi-pathway drug-drug interactions: A case study of irinotecan, a colon cancer medication." Saudi Pharmaceutical Journal 28(12): 1507-1513. | Title |
| 391 | Attia, Z. R., et al. (2024). "Pharmacogenetic insights into ABCB1, ABCC2, CYP1A2, and CYP2B6 variants with epilepsy susceptibility among Egyptian Children: A retrospective case-control study." International Immunopharmacology 142: 113073. | Title |
| 392 | Atzeni, F., et al. (2013). "Different effects of biological drugs in rheumatoid arthritis." Autoimmun Rev 12(5): 575-579. | Title |
| 393 | Avni, T., et al. (2021). "Tocilizumab in the treatment of COVID-19—A meta-analysis." 114(8): 577-586. | Title |
| 394 | Awais Ali Zaidi, S., et al. (2023). "Benzoic-D5 acid as D2 receptor agonist in the treatment of rotenone induced Parkinson's disease in mice." Arabian Journal of Chemistry 16(8): 104982. | Title |
| 395 | Awaji, A. A., et al. (2024). "N- and s-substituted Pyrazolopyrimidines: A promising new class of potent c-Src kinase inhibitors with prominent antitumor activity." Bioorganic Chemistry 145: 107228. | Title |
| 396 | Ayaz, A., et al. (2010). "Induction of Labor: A Comparative Study of Intravaginal Misoprostol and Dinoprostone." Taiwanese Journal of Obstetrics and Gynecology 49(2): 151-155. | Title |
| 397 | Aye, S. M., et al. (2020). "Polymyxin triple combinations against polymyxin-resistant, multidrug-resistant, KPC-producing Klebsiella pneumoniae." 64(8): 10.1128/aac. 00246-00220. | Title |
| 398 | Ayfan, A. K., et al. (2022). "Proof-of-concept, rapid, instrument-free molecular detection of Neisseria gonorrhoeae and ciprofloxacin susceptibility." 77(11): 2933-2936. | Title |

| | | |
|---|---|---|
| 399 | Ayoup, M. S., et al. (2024). "Challenging the anticolorectal cancer capacity of quinoxaline-based scaffold via triazole ligation unveiled new efficient dual VEGFR-2/MAO-B inhibitors." Bioorganic Chemistry 143: 107102. | Title |
| 400 | Azer, S. J. E. R. f. M. and P. Sciences (2014). "Understanding pharmacokinetics: are YouTube videos a useful learning resource?" 18(13). | Title |
| 401 | Aziz, N. D., et al. (2023). "aSSoCiatioN oF SolUtE CarriEr orGaNiC aNioN traNSPortEr 1B1 GENE PolyMorPHiSM witH rESPoNSE to atorvaStatiN aNd aSSoCiatEd MyoPatHy iN iraQi dySliPidEMia PatiENtS." 496. | Title |
| 402 | Aziz, S., et al. (2023). "Identifying non-nucleoside inhibitors of RNA-dependent RNA-polymerase of SARS-CoV-2 through per-residue energy decomposition-based pharmacophore modeling, molecular docking, and molecular dynamics simulation." Journal of Infection and Public Health 16(4): 501-519. | Title |
| 403 | Azzam, N., et al. (2021). "Impact of infliximab therapeutic drug level monitoring on outcomes of patients with inflammatory bowel disease: A real-world experience from a Middle Eastern cohort." Arab Journal of Gastroenterology 22(1): 66-72. | Title |
| 404 | Babar, Z.-U.-D., et al. (2011). "Examination of students' interest in a public health pharmacy course in Malaysia." Currents in Pharmacy Teaching and Learning 3(3): 199-207. | Title |
| 405 | Babatin, M., et al. (2008). "Amiodarone hepatotoxicity." 6(3): 228-236. | Title |
| 406 | Babelghaith, S. D., et al. (2024). "The use of complementary and alternative medicine for functional gastrointestinal disorders among the saudi population." Saudi Pharmaceutical Journal 32(6): 102084. | Title |
| 407 | Badia Ferrando, P., et al. (2011). "Lumbar fusion by mini-invasive TLIF: prospective study at 5 years' follow-up." European spine journal 20(11): 2079. | Title |
| 408 | Badr, M. Y., et al. (2021). "A polymeric aqueous tacrolimus formulation for topical ocular delivery." Int J Pharm 599: 120364. | Title |
| 409 | Badr, M. Y., et al. (2022). "The topical ocular delivery of rapamycin to posterior eye tissues and the suppression of retinal inflammatory disease." Int J Pharm 621: 121755. | Title |
| 410 | Badran, S. A., et al. (2020). "Megestrol acetate induced proliferation and differentiation of osteoblastic MC3T3-E1 cells: A drug repurposing approach." Steroids 157: 108607. | Title |
| 411 | Badwan, A. A., et al. (2002). Pantoprazole Sodium. Analytical Profiles of Drug Substances and Excipients. H. G. Brittain, Academic Press. 29: 213-259. | Title |

| | | |
|---|---|---|
| 412 | Ba-Essa, E. M., et al. (2019). "Attitude and safety of patients with diabetes observing the Ramadan fast." Diabetes Research and Clinical Practice 152: 177-182. | Title |
| 413 | Baggio, G., et al. (2013). "Gender medicine: a task for the third millennium." Clin Chem Lab Med 51(4): 713-727. | Title |
| 414 | Bagherzadeh, K., et al. (2020). "Proton pump inhibitors in Iranian population: from clinical regimens to pharmacogenomics." 24(4): 230-249. | Title |
| 415 | Bagirova, N., et al. (2004). "Patients with hematological malignancies." 8: S41-S49. | Title |
| 416 | Bahmani, M., et al. (2014). "The most common herbal medicines affecting Sarcomastigophora branches: a review study." Asian Pacific Journal of Tropical Medicine 7: S14-S21. | Title |
| 417 | Bai, J. P. F. and E. Y. Guo (2022). "Combating Viral Diseases in the Era of Systems Medicine." Methods Mol Biol 2486: 87-104. | Title |
| 418 | Baig, W. A., et al. (2022). "Synergistic anti-cancer effects of Nigella sativa seed oil and conventional cytotoxic agent against human breast cancer." 37(3): 315-321. | Title |
| 419 | Bair, M. J., et al. (2014). "Evaluation of stepped care for chronic pain (ESCAPE) in veterans of Iraq and Afghanistan: a randomized trial." Journal of general internal medicine 29: S89. | Title |
| 420 | Bajer-Kornek, B., et al. (2015). "Safety and effect of fingolimod on no evidence of disease activity (NEDA-4) in young adult patients with relapsing-remitting multiple sclerosis." European journal of paediatric neurology 19: S22. | Title |
| 421 | Bajer-Kornek, B., et al. (2014). "Oral fingolimod vs interferon-b 1a in paediatric multiple sclerosis: design of a double-blind trial." Developmental medicine and child neurology 56: 39. | Title |
| 422 | Bakır Ekinci, P., et al. (2021). "Colistin Induced Nephrotoxicity: Experience from a University Hospital." 10. | Title |
| 423 | Bakish, D., et al. (2012). "Levomilnacipran sr 40mg and 80 mg in major depressive disorder: a phase III, randomized, double-blind, fixed-dose, placebo-controlled study." Neuropsychopharmacology 38: S427-S428. | Title |
| 424 | Bakthavatchalam, Y. D., et al. (2018). "Polymyxin susceptibility testing, interpretative breakpoints and resistance mechanisms: An update." 12: 124-136. | Title |
| 425 | Balakrishnan, A., et al. (2022). "Metformin as an emerging concern in wastewater: Occurrence, analysis and treatment methods." Environmental Research 213: 113613. | Title |
| 426 | Balant, L. P. and P. G. J. P. o. D. Welling (1994). "Interethnic differences in drug disposition and response: relevance for drug development, licensing, and registration." 233-263. | Title |

| | | |
|---|---|---|
| 427 | Balkhi, B., et al. (2020). "Drug utilization and expenditure of anticancer drugs for breast cancer." Saudi Pharmaceutical Journal 28(6): 669-674. | Title |
| 428 | Ball, P., et al. (2001). "Efficacy and safety of gemifloxacin 320 mg once-daily for 7 days in the treatment of adult lower respiratory tract infections." International Journal of Antimicrobial Agents 18(1): 19-27. | Title |
| 429 | Bandarapalle, K., et al. (2024). "FUTURE JOURNAL OF PHARMACEUTICALS AND HEALTH SCIENCES." | Title |
| 430 | Banerjee, A., et al. (2023). "Understanding the bacteria in Mycobacterium avium complex (MAC) from a bioinformatic perspective–a review." 67(2): 203-220. | Title |
| 431 | Bannan, D. F., et al. (2019). "Understanding the causes of prescribing errors from a behavioural perspective." Research in Social and Administrative Pharmacy 15(5): 546-557. | Title |
| 432 | Bano, M., et al. (2024). "Utilizing Cinnamomum verum (a culinary spice), as a functional food ingredient ameliorating hypercholesterolemia: In-vivo, in-vitro, and in-silico multi-model analysis." Food Bioscience 62: 105153. | Title |
| 433 | Bansback, N. J., et al. (2005). "Cost effectiveness of adalimumab in the treatment of patients with moderate to severe rheumatoid arthritis in Sweden." Ann Rheum Dis 64(7): 995-1002. | Title |
| 434 | Baptista, L., et al. (2023). "What is new in augmented renal clearance in septic patients?" 25(11): 255-272. | Title |
| 435 | Barakat, A., et al. (2023). "Synthesis, In Vitro and in Cell Study of a New Spirooxindoles-Based N-Alkylated Maleimides Targeting HER2/3 Signaling Pathway." Polycyclic Aromatic Compounds 43(6): 5251-5275. | Title |
| 436 | Barnes, T. R. and D. A. Curson (1994). "Long-term depot antipsychotics. A risk-benefit assessment." Drug Saf 10(6): 464-479. | Title |
| 437 | Bar-Or, A., et al. (2013). "Effect of BG-12 (dimethyl fumarate) in subgroups of patients with relapsing-remitting multiple sclerosis: an integrated analysis of the phase 3 define and confirm studies." Neurology 80(1). | Title |
| 438 | Bar-Or, A., et al. (2012). "Clinical effects of BG-12 in subgroups of patients with relapsing-remitting multiple sclerosis: an integrated analysis of the phase 3 DEFINE and CONFIRM studies." Multiple sclerosis (Houndmills, Basingstoke, England) 18(4): 191. | Title |
| 439 | Bar-Or, A., et al. (2015). "Efficacy of delayed-release dimethyl fumarate for relapsing-remitting multiple sclerosis using a composite measure of disability: integrated analysis of the phase 3 DEFINE and CONFIRM studies." Multiple sclerosis (Houndmills, Basingstoke, England) 23(11): 303-304. | Title |
| 440 | Barral, M., et al. (2016). "Mechanical thrombectomy in octogenarian stroke patients is safe and effective." Stroke 47(no pagination). | Title |

| | | |
|---|---|---|
| 441 | Bartholomeusz, C. F., et al. (2012). "Social cognition training as an intervention for improving functional outcome in first episode psychosis: a pilot study." Schizophrenia Research 136: S172. | Title |
| 442 | Basco, S. A. and J. E. J. I. D. C. Girotto (2022). "Contemporary treatment of resistant gram-negative infections in pediatric patients." 36(1): 147-171. | Title |
| 443 | Basharat, Z., et al. (2022). "Differential analysis of Orientia tsutsugamushi genomes for therapeutic target identification and possible intervention through natural product inhibitor screening." Computers in Biology and Medicine 141: 105165. | Title |
| 444 | Basharat, Z., et al. (2022). "An in silico hierarchal approach for drug candidate mining and validation of natural product inhibitors against pyrimidine biosynthesis enzyme in the antibiotic-resistant Shigella flexneri." Infection, Genetics and Evolution 98: 105233. | Title |
| 445 | Basharat, Z. and A. Meshal (2024). "Pan-genome mediated therapeutic target mining in Kingella kingae and inhibition assessment using traditional Chinese medicinal compounds: an informatics approach." J Biomol Struct Dyn 42(6): 2872-2885. | Title |
| 446 | Basonbul, A. A. (2024). "Evaluation of Targeted Selective Inhibitors to Enhance Temozolomide Treatment Sensitivity in Acute Myeloid Leukemia." | Title |
| 447 | Bassetti, M., et al. (2020). "Optimal management of complicated infections in the pediatric patient: the role and utility of ceftazidime/avibactam." 1763-1773. | Title |
| 448 | Bassetti, M., et al. (2020). "Overcoming Antibiotic Resistance: New Perspectives." 457-480. | Title |
| 449 | Bassi, M., et al. (2024). "The synthetic cathinones MDPHP and MDPV: Comparison of the acute effects in mice, in silico ADMET profiles and clinical reports." NeuroToxicology 103: 230-255. | Animal |
| 450 | Bateman, R. M., et al. (2016). "36th International Symposium on Intensive Care and Emergency Medicine : Brussels, Belgium. 15-18 March 2016." Crit Care 20(Suppl 2): 94. | Title |
| 451 | Batista, L., et al. (2017). "Low efficacy of metronidazole in the eradication of Blastocystis hominis in symptomatic patients: Case series and systematic literature review." Gastroenterología y Hepatología (English Edition) 40(6): 381-387. | Title |
| 452 | Bauters, T., et al. (2008). "Platform Presentations." | Title |
| 453 | Bawadikji, A. A., et al. "Journal of Population Therapeutics & Clinical Pharmacology." | Title |
| 454 | Beg, S., et al. (2021). "Implications of phospholipid-based nanomixed micelles of olmesartan medoxomil with enhanced lymphatic drug targeting ability and systemic bioavailability." Journal of Drug Delivery Science and Technology 62: 102273. | Title |

| | | |
|---|---|---|
| 455 | Beghi, E. (2012). "Double-blind placebo-controlled trial on the use of acetyl-l-carnitine for the treatment of amyotrophic lateral sclerosis." Neurology 78(1). | Title |
| 456 | Bekele, F., et al. (2021). "“Childrens are not just “little adults”. The rate of medication related problems and its predictors among patients admitted to pediatric ward of southwestern Ethiopian hospital: A prospective observational study." Annals of Medicine and Surgery 70: 102827. | Title |
| 457 | Bélard, S. M. (2007). Efficacy, safety, tolerability and acceptability of two pediatric formulations of Artesunate-Mefloquine in African children with acute uncomplicated Plasmodium falciparum Malaria, Universität Tübingen. | Title |
| 458 | Bellos, I., et al. (2020). "Efficacy and safety of colistin loading dose: a meta-analysis." 75(7): 1689-1698. | Title |
| 459 | Bencsik, K., et al. (2015). "[TERIFLUNOMIDE: A NEW ORAL IMMUNOMODULATING AGENT FOR MULTIPLE SCLEROSIS]." Ideggyogy Sz 68(3-4): 79-87. | Title |
| 460 | Ben-Harari, R. R., et al. (2017). "Adverse event profile of pyrimethamine-based therapy in toxoplasmosis: a systematic review." 17: 523-544. | Title |
| 461 | Benini, F. and E. J. I. J. o. P. Barbi (2014). "Doing without codeine: why and what are the alternatives?" 40: 1-5. | Title |
| 462 | Bennett, J., et al. (2021). "SAkuraBONSAI: a prospective, open-label study of satralizumab investigating novel imaging, biomarker, and clinical outcomes in patients with AQP4-IgG seropositive NMOSD." Multiple sclerosis journal 27(2 SUPPL): 160-162. | Title |
| 463 | Bennett, J. L., et al. (2023). "SAkuraBONSAI: protocol design of a novel, prospective study to explore clinical, imaging, and biomarker outcomes in patients with AQP4-IgG-seropositive neuromyelitis optica spectrum disorder receiving open-label satralizumab." Frontiers in neurology 14. | Title |
| 464 | Bennouna, J., et al. (2013). "Continuation of bevacizumab after first progression in metastatic colorectal cancer (ML18147): a randomised phase 3 trial." The Lancet Oncology 14(1): 29-37. | Title |
| 465 | Benyettou, F., et al. (2021). "In vivo oral insulin delivery via covalent organic frameworks††Electronic supplementary information (ESI) available. See DOI: 10.1039/d0sc05328g." Chemical Science 12(17): 6037-6047. | Title |
| 466 | Bergvall, N., et al. (2013). "Cost-effectiveness of fingolimod compared to interferon β 1a based on patient transitions in TRANSFORMS." Multiple sclerosis (Houndmills, Basingstoke, England) 19(11): 276-277. | Title |
| 467 | Bergvall, N., et al. (2013). "Effects of fingolimod on disability progression in patients with disability as measured by edss at baseline: post-HOC analyses of freedoms I and II." Neurology 80(1). | Title |

| | | |
|---|---|---|
| 468 | Bernardini, F., et al. (2002). "Developmental disorders." 11: 2129-2142. | Title |
| 469 | Bertolini, A., et al. (2001). "Dual acting anti-inflammatory drugs: a reappraisal." Pharmacol Res 44(6): 437-450. | Title |
| 470 | Bertrand, X. and M. J. Dowzicky (2012). "Antimicrobial Susceptibility Among Gram-Negative Isolates Collected From Intensive Care Units in North America, Europe, the Asia-Pacific Rim, Latin America, the Middle East, and Africa Between 2004 and 2009 as Part of the Tigecycline Evaluation and Surveillance Trial." Clin Ther 34(1): 124-137. | Title |
| 471 | Besag, F. M. (1998). "Lamotrigine in the treatment of epilepsy in people with intellectual disability." J Intellect Disabil Res 42 Suppl 1: 50-56. | Title |
| 472 | Besag, F. M. (2011). "Rufinamide for the treatment of Lennox-Gastaut syndrome." Expert Opin Pharmacother 12(5): 801-806. | Title |
| 473 | Bettega, P. V. C., et al. (2020). "Lorazepam induces acinar cells apoptosis of rat parotid glands." The Saudi Dental Journal 32(6): 276-282. | Title |
| 474 | Bettelli, G. (2011). "Preoperative evaluation in geriatric surgery: comorbidity, functional status and pharmacological history." Minerva Anestesiol 77(6): 637-646. | Title |
| 475 | Bever, C. T. and S. I. Judge (2009). "Sustained-release fampridine for multiple sclerosis." Expert Opin Investig Drugs 18(7): 1013-1024. | Title |
| 476 | Bever, C. T., Jr., et al. (1994). "The effects of 4-aminopyridine in multiple sclerosis patients: results of a randomized, placebo-controlled, double-blind, concentration-controlled, crossover trial." Neurology 44(6): 1054-1059. | Title |
| 477 | Bharathiraja, P., et al. (2024). "Solasodine targets NF-κB signaling to overcome P-glycoprotein mediated multidrug resistance in cancer." Experimental Cell Research 441(1): 114153. | Title |
| 478 | Bhatnagar, M. E., et al. (2024). Regulatory considerations in the design and conduct of pediatric clinical trials. Essentials of Translational Pediatric Drug Development, Elsevier: 385-419. | Title |
| 479 | Bhattacharya, R., et al. (2022). "Strategies to Improve Insulin Delivery through Oral Route: A Review." Curr Drug Deliv 19(3): 317-336. | Title |
| 480 | Bhaumik, S., et al. (2024). "α-Glucosidase inhibitory potential of Oroxylum indicum using molecular docking, molecular dynamics, and in vitro evaluation." Saudi Pharmaceutical Journal 32(6): 102095. | Title |
| 481 | Bibi, S., et al. (2022). "Virtual screening and molecular dynamics simulation analysis of Forsythoside A as a plant-derived inhibitor of SARS-CoV-2 3CLpro." Saudi Pharmaceutical Journal 30(7): 979-1002. | Title |
| 482 | Bible, K. C., et al. (2010). "Efficacy of pazopanib in progressive, radioiodine-refractory, metastatic differentiated thyroid cancers: results of a phase 2 consortium study." The Lancet Oncology 11(10): 962-972. | Title |
| 483 | Bielory, L. (2002). "Update on ocular allergy treatment." Expert Opin Pharmacother 3(5): 541-553. | Title |

| | | |
|---|---|---|
| 484 | Biemond, B. J., et al. (2021). "Sevuparin for the treatment of acute pain crisis in patients with sickle cell disease: a multicentre, randomised, double-blind, placebo-controlled, phase 2 trial." The Lancet Haematology 8(5): e334-e343. | Title |
| 485 | Biemond, B. J., et al. (2019). "Efficacy and Safety of Sevuparin, a Novel Non-Anti-Coagulant Heparinoid, in Patients with Acute Painful Vaso-Occlusive Crisis; A Global, Multicenter Double-Blind, Randomized, Placebo-Controlled Phase 2 Trial (TVOC01)." Blood 134: 614. | Title |
| 486 | Bifari, N., et al. (2024). "Unraveling medication errors in enteral tube administration: A cross-sectional study in geriatric patients receiving home health care." Saudi Pharmaceutical Journal 32(2): 101938. | Title |
| 487 | Bilal, M., et al. (2024). "Assessment of body mass-related covariates for rifampicin pharmacokinetics in healthy Caucasian volunteers." Eur J Clin Pharmacol 80(9): 1271-1283. | Title |
| 488 | Binkhathlan, Z., et al. (2024). "Polycaprolactone – Vitamin E TPGS micelles for delivery of paclitaxel: In vitro and in vivo evaluation." International Journal of Pharmaceutics: X 7: 100253. | Title |
| 489 | Binsuwaidan, R., et al. (2022). "Antibacterial activity and wound healing potential of Cycas thouarsii R.Br n-butanol fraction in diabetic rats supported with phytochemical profiling." Biomedicine & Pharmacotherapy 155: 113763. | Title |
| 490 | Bird, P., et al. (2016). "A placebo controlled trial of vertebral fill technique vertebroplasty for acute painful osteoporotic fracture (vapour trial)." Arthritis & rheumatology 68: 413-414. | Title |
| 491 | Blanco, P. J., et al. (2019). "Exploring the Impact of Child-Centered Play Therapy on Academic Achievement of At-Risk Kindergarten Students." International journal of play therapy 28(3): 133-143. | Title |
| 492 | Blank, J. J., et al. (2018). "The impact of intravenous acetaminophen on pain after abdominal surgery: a meta-analysis." Journal of Surgical Research 227: 234-245. | Title |
| 493 | Blasi, F., et al. (2009). "Antibacterial activity of telithromycin and comparators against pathogens isolated from patients with community-acquired respiratory tract infections: the Prospective Resistant Organism Tracking and Epidemiology for the Ketolide Telithromycin study year 5 (2003–2004)." Diagn Microbiol Infect Dis 63(3): 302-308. | Title |
| 494 | Blattler, T., et al. (2012). "Impact of improving negative symptoms on functioning in patients with schizophrenia: a post-hoc analysis of a proof-of-concept study." European neuropsychopharmacology 22: S310-S311. | Title |
| 495 | Blauvelt, A., et al. (2014). "Secukinumab efficacy in subjects with moderate-to-severe plaque psoriasis and concomitant psoriatic arthritis: a subanalysis of the ERASURE study." Journal of the American Academy of Dermatology 70(5): AB2. | Title |

| | | |
|---|---|---|
| 496 | Błażewicz, A., et al. (2021). "Alterations of urinary perchlorate levels in euthyroid postpubertal children with autism spectrum disorder." Journal of Trace Elements in Medicine and Biology 68: 126800. | Title |
| 497 | Blin, P., et al. (2019). "Effectiveness and safety of rivaroxaban 15 or 20 mg versus vitamin K antagonists in nonvalvular atrial fibrillation: a population-based new users high-dimensional propensity score matched cohorts study." 50(9): 2469-2476. | Title |
| 498 | Block, S. L. (1998). "Attention-deficit disorder. A paradigm for psychotropic medication intervention in pediatrics." Pediatr Clin North Am 45(5): 1053-1083. | Title |
| 499 | Bodenstein, L., et al. "Cum Laude." | Title |
| 500 | Bogacz, A., et al. (2020). "The effect of genetic variations for interleukin-10 (IL-10) on the efficacy of immunosuppressive therapy in patients after kidney transplantation." International Immunopharmacology 89: 107059. | Title |
| 501 | Bogan, R. K., et al. (2012). "A post-HOC analysis examining the efficacy and tolerability of armodafinil in healthcare workers with excessive sleepiness associated with shift work disorder." Sleep 35: A209. | Title |
| 502 | Bogolepova, A. N. (2023). "[Cerebrolysin in the treatment of cognitive impairment]." Zh Nevrol Psikhiatr Im S S Korsakova 123(3): 20-25. | Title |
| 503 | Bolliger, C. T., et al. (2011). "Effects of Varenicline in Adult Smokers: A Multinational, 24-Week, Randomized, Double-Blind, Placebo-Controlled Study." Clin Ther 33(4): 465-477. | Title |
| 504 | Bombardieri, S., et al. (2009). "[Copernican revolution in the therapy of rheumatoid arthritis: the contribution of anti-TNFalpha drugs]." Reumatismo 61 Suppl 1: 1-23. | Title |
| 505 | Boonlue, T., et al. (2024). "Factors associated with subtherapeutic levels of valproic acid in hospitalized patients with epilepsy: A retrospective cohort study." 103(45): e40488. | Title |
| 506 | Booth, K., et al. (2012). "Safety, tolerabilityand efficacy of lecozotan sr in patients with mild-tomoderate Alzheimer's disease used as monotherapy or adjunctively with a cholinesterase inhibitor." Alzheimer's & dementia 8(4): P604. | Title |
| 507 | Boreham, P. F. L. and D. J. Stenzel (1993). Blastocystis in Humans and Animals: Morphology, Biology, and Epizootiology. Advances in Parasitology. J. R. Baker and R. Muller, Academic Press. 32: 1-70. | Title |
| 508 | Bose, A., et al. (2012). "The efficacy and safety of levomilnacipran in the treatment of major depressive disorder: results from a phase III clinical trial." International journal of neuropsychopharmacology 15: 182. | Title |

| | | |
|---|---|---|
| 509 | Boshkov, L. K. and J. G. Kelton (1989). "Use of Intravenous Gammaglobulin as an Immune Replacement and an Immune Suppressant." Transfusion Medicine Reviews 3(2): 82-120. | Title |
| 510 | Boster, A., et al. (2015). "Disease activity in the first year predicts longer-term clinical outcomes in the pooled population of the phase III freedoms and freedoms II studies." Neurology 84. | Title |
| 511 | Bouillon, R., et al. (2006). "Pharmacology, metabolism and nutrition." 174: 1319-1326. | Title |
| 512 | Boussery, K., et al. (2011). "Pharmacokinetics of two formulations of omeprazole administered through a gastrostomy tube in patients with severe neurodevelopmental problems." Br J Clin Pharmacol 72(6): 990-996. | Title |
| 513 | Bowie, C. R. and P. D. Harvey (2006). "Treatment of cognitive deficits in schizophrenia." Curr Opin Investig Drugs 7(7): 608-613. | Title |
| 514 | Boyce, E. G., et al. (2018). "Sarilumab: Review of a Second IL-6 Receptor Antagonist Indicated for the Treatment of Rheumatoid Arthritis." Ann Pharmacother 52(8): 780-791. | Title |
| 515 | Brabant, T. and D. Stichtenoth (2005). "[Pharmacological treatment of osteoarthritis in the elderly]." Z Rheumatol 64(7): 467-472. | Title |
| 516 | Brashear, A. (2001). "Botulinum toxin type B: a new injectable treatment for cervical dystonia." Expert Opin Investig Drugs 10(12): 2191-2199. | Title |
| 517 | Brauch, H., et al. (2009). "Pharmacogenomics of tamoxifen therapy." 55(10): 1770-1782. | Title |
| 518 | Brawman-Mintzer, O., et al. (2013). "Psychosocial characteristics and pain burden of patients with suspected sphincter of oddi dysfunction (SOD) enrolled in the episod trial." Gastrointestinal endoscopy 77(5): AB385. | Title |
| 519 | Brenes, G. A., et al. (2016). "Effects of Telephone-Delivered Cognitive-Behavioral Therapy and Nondirective Supportive Therapy on Sleep, Health-Related Quality of Life, and Disability." American journal of geriatric psychiatry 24(10): 846-854. | Title |
| 520 | Brennan, D. (2018). "EG-1962 for the treatment of aneurysmal subarachnoid hemorrhage." Neurotherapeutics 15(3): 823-824. | Title |
| 521 | Brenner, A., et al. (2019). "Tranexamic acid for acute gastrointestinal bleeding (the HALT-IT trial): statistical analysis plan for an international, randomised, double-blind, placebo-controlled trial." Trials 20(1): 467. | Title |
| 522 | Brink, A. J., et al. (2013). "Emergence of OXA-48 and OXA-181 carbapenemases among Enterobacteriaceae in South Africa and evidence of in vivo selection of colistin resistance as a consequence of selective decontamination of the gastrointestinal tract." 51(1): 369-372. | Title |
| 523 | Brixner, D., et al. (2018). "An Evidence Framework for Off-Patent Pharmaceutical Review for Health Technology Assessment in Emerging Markets." Value in Health Regional Issues 16: 9-13. | Title |
| 524 | Brochot, E., et al. (2010). "Ribavirin monitoring in chronic hepatitis C therapy: anaemia versus efficacy." 15(5): 687-695. | Title |

| | | |
|---|---|---|
| 525 | Brodie, M. J. (1999). "Monostars: an aid to choosing an antiepileptic drug as monotherapy." Epilepsia 40 Suppl 6: S17-22; discussion S73-14. | Title |
| 526 | Broste, S., et al. (2013). "Novel clinical trial design to study the benefits of a position-adaptive spinal cord stimulation feature." Regional anesthesia and pain medicine 38(1). | Title |
| 527 | Brousil, J. A., et al. (2006). "Cladribine: an investigational immunomodulatory agent for multiple sclerosis." Ann Pharmacother 40(10): 1814-1821. | Title |
| 528 | Brown, A., et al. (2002). "Effect of a national community intervention programme on healing rates of chronic leg ulcer: randomised controlled trial." Phlebology / Venous Forum of the Royal Society of Medicine 17(2): 47-53. | Title |
| 529 | Brown, M. L., et al. (2020). Evaluation of renal safety between imipenem/relebactam and colistin plus imipenem in patients with imipenem-nonsusceptible bacterial infections in the randomized, phase 3 RESTORE-IMI 1 study. Open Forum Infectious Diseases, Oxford University Press US. | Title |
| 530 | Brown, W. M. and S. P. Aiken (1998). "Felbamate: clinical and molecular aspects of a unique antiepileptic drug." Crit Rev Neurobiol 12(3): 205-222. | Title |
| 531 | Brunello, A., et al. (2007). "Ifosfamide-related encephalopathy in elderly patients : report of five cases and review of the literature." Drugs Aging 24(11): 967-973. | Title |
| 532 | Brunstein, C. G., et al. (2019). "Effect of Conditioning Regimen Dose Reduction in Obese Patients Undergoing Autologous Hematopoietic Cell Transplantation." Biology of Blood and Marrow Transplantation 25(3): 480-487. | Title |
| 533 | Buckley, L. F., et al. (2020). "Role for anti-cytokine therapies in severe coronavirus disease 2019." 2(8): e0178. | Title |
| 534 | Bukh, G., et al. (2011). "Continued tocilizumab infusion for rheumatoid arthritis is well tolerated and safe at an accelerated infusion rate." Arthritis Rheum 63(10). | Title |
| 535 | Burmeister, D. B., et al. (2013). "Management of benign paroxysmal positional vertigo: a randomized control trial." Annals of emergency medicine 62(4): S88. | Title |
| 536 | Burmester, G., et al. (2015). "Tocilizumab monotherapy in early rheumatoid arthritis: data from two phase 3 randomized controlled trials." Arthritis & rheumatology 67(no pagination). | Title |
| 537 | Burness, C. B. and E. D. Deeks (2014). "Dimethyl fumarate: a review of its use in patients with relapsing-remitting multiple sclerosis." CNS Drugs 28(4): 373-387. | Title |

| | | |
|---|---|---|
| 538 | Burtness, B. (2017). "Treatment de-intensification strategies for head and neck cancer." Clinical cancer research 23(23). | Title |
| 539 | Burzynski, H. E. (2023). "Progressive Neurochemical, Neuroinflammatory and Cognitive Deficits in an Experimental Model of Gulf War Illness." | Title |
| 540 | Butranova, O. I., et al. (2023). "Pharmacokinetics of Antibacterial Agents in the Elderly: The Body of Evidence." Biomedicines 11(6). | Title |
| 541 | Cacabelos, R. (2009). "Pharmacogenomics and therapeutic strategies for dementia." Expert Rev Mol Diagn 9(6): 567-611. | Title |
| 542 | Cacchio, A., et al. (2019). "Effectiveness and safety of a mixture of diosmin, coumarin and arbutin (Linfadren®) in addition to conventional treatment in the management of patients with post-trauma/surgery persistent hand edema: a randomized controlled trial." Clinical rehabilitation 33(5): 904-912. | Title |
| 543 | Cadavid, D., et al. (2013). "The MS-COG, a novel endpoint for measurement of cognitive function in multiple sclerosis clinical trials: baseline characteristics of the cognitive substudy of the ASCEND natalizumab secondary progressive multiple sclerosis study." Multiple sclerosis (Houndmills, Basingstoke, England) 19(11): 508. | Title |
| 544 | Cady, R. and D. W. Dodick (2002). "Diagnosis and treatment of migraine." Mayo Clin Proc 77(3): 255-261. | Title |
| 545 | Calabrese, J. R., et al. (2013). "Efficacy and safety of treatment with lurasidone adjunctive to lithium or valproate in bipolar I depression: results of two 6-week studies." Neuropsychopharmacology 38: S532-S533. | Title |
| 546 | Calabresi, P. A., et al. (2014). "Clinical efficacy of peginterferon beta-1a in relapsingremitting multiple sclerosis: 2-year data from the phase 3 ADVANCE study." Multiple sclerosis (Houndmills, Basingstoke, England) 20(1): 42-43. | Title |
| 547 | Cameron, A. P., et al. (2010). "National trends in the usage and success of sacral neuromodulation in the medicare population." Neurourology and urodynamics 29(2): 262-263. | Title |
| 548 | Cameron, I. D., et al. (2001). "Hip protectors in aged-care facilities: a randomized trial of use by individual higher-risk residents." Age Ageing 30(6): 477-481. | Title |
| 549 | Campbell, J. R., et al. (2020). "Adverse events in adults with latent tuberculosis infection receiving daily rifampicin or isoniazid: post-hoc safety analysis of two randomised controlled trials." The lancet. Infectious diseases 20(3): 318-329. | Title |
| 550 | Capi, M., et al. (2016). "Eletriptan in the management of acute migraine: an update on the evidence for efficacy, safety, and consistent response." Ther Adv Neurol Disord 9(5): 414-423. | Title |

| | | |
|---|---|---|
| 551 | Cardona-Hernandez, R., et al. (2023). "New therapies towards a better glycemic control in youths with type 1 diabetes." Pharmacol Res 195: 106882. | Title |
| 552 | Carmona, F., et al. (2023). "A polyherbal formulation containing Justicia pectoralis Jacq., Achyrocline satureioides (Lam.) DC., and Eclipta prostrata (L.) L. helped reducing symptom intensity during and after Covid-19 infection: A retrospective, uncontrolled observational study." Advances in Integrative Medicine 10(3): 113-121. | Title |
| 553 | Carroll, C. A. and M. A. Oleen-Burkey (2012). "Assessing variations in transitions in employment in relapsingremitting multiple sclerosis patients treated with either laquinimod, interferon beta 1-a or placebo: expoloratory evidence from the United States substudy of bravo." Value in health 15(4): A149. | Title |
| 554 | Carroll, S. M., et al. (2009). "Overcoming the challenges of modelling schizophrenia: a UK case study of the cost-effectiveness of olanzapine long-acting injection vs. risperidone long-acting injection." Value in health 12(7): A350. | Title |
| 555 | Carta, A. R., et al. (2008). "Behavioral and biochemical correlates of the dyskinetic potential of dopaminergic agonists in the 6-OHDA lesioned rat." Synapse 62(7): 524-533. | Title |
| 556 | Carter, N. J. and P. L. McCormack (2009). "Duloxetine: a review of its use in the treatment of generalized anxiety disorder." CNS Drugs 23(6): 523-541. | Title |
| 557 | Cazotti, L. A., et al. (2015). "Effectiveness of the Pilates method in the treatment of chronic mechanical-postural neck pain." Ann Rheum Dis 74: 1345-1346. | Title |
| 558 | Ceccato, A., et al. (2021). "Safety considerations of current drug treatment strategies for nosocomial pneumonia." 20(2): 181-190. | Title |
| 559 | Ceci, A., et al. (2019). "An overview of the efficacy and safety of deferiprone in paediatric patients with congenital haemoglobinopathies and chronic iron overload." 7(4): 181-197. | Title |
| 560 | Cedarbaum, J. M. (1990). "Pharmacokinetic and pharmacodynamic considerations in management of motor response fluctuations in Parkinson's disease." Neurol Clin 8(1): 31-49. | Title |
| 561 | Cedarbaum, J. M., et al. (1987). "Controlled-release levodopa/carbidopa. I. Sinemet CR3 treatment of response fluctuations in Parkinson's disease." Neurology 37(2): 233-241. | Title |
| 562 | Celestin, C., et al. (2014). "Cost-effectiveness of alemtuzumab vs subcutaneous interferon beta-1a for treatment of active relapsingremitting multiple sclerosis: payer perspective." Multiple sclerosis (Houndmills, Basingstoke, England) 20(1): 72. | Title |

| | | |
|---|---|---|
| 563 | Celik, I., et al. (2023). "Resveratrol and Its Natural Analogues Inhibit RNA Dependant RNA Polymerase (RdRp) of Rhizopus oryzae in Mucormycosis through Computational Investigations." Polycyclic Aromatic Compounds 43(5): 4426-4443. | Title |
| 564 | Chakrabarti, K., et al. (2021). "The use of mycophenolate mofetil area under the curve." 33(3): 221-232. | Title |
| 565 | Chalder, T., et al. (2003). "Predictors of outcome in a fatigued population in primary care following a randomized controlled trial." Psychological medicine 33(2): 283-287. | Title |
| 566 | Chambial, P., et al. (2024). "Sequential catalytic nanomedicinal utilization for synergistic drug delivery application in cancer nanotechnology." Journal of Molecular Structure 1312: 138388. | Title |
| 567 | Chamoun, K., et al. (2016). "Surveillance of antimicrobial resistance in Lebanese hospitals: retrospective nationwide compiled data." International Journal of Infectious Diseases 46: 64-70. | Title |
| 568 | Chan, A., et al. (2014). "Differential recovery from relapse between treatment groups in the CONFIRM study of delayed-release dimethyl fumarate." Multiple sclerosis (Houndmills, Basingstoke, England) 20(1): 110. | Title |
| 569 | Chan, A., et al. (2015). "Differential recovery from relapse between treatment groups in the CONFIRM study of delayed-release dimethyl fumarate." Eur J Neurol 22: 297. | Title |
| 570 | Chandra, H., et al. (2024). "COVID 19: Prevention and treatment through the Indian perspective." Cytokine 183: 156756. | Title |
| 571 | Charache, S. (1990). "Fetal Hemoglobin, Sickling, and Sickle Cell Disease." Advances in Pediatrics 37(1): 1-31. | Title |
| 572 | Charache, S., et al. (1995). "Design of the multicenter study of hydroxyurea in sickle cell anemia." Controlled Clinical Trials 16(6): 432-446. | Title |
| 573 | Charles-Schoeman, C., et al. (2014). "Efficacy and safety of tofacitinib following inadequate response to nonbiologic DMARD or biologic DMARD." Arthritis & rheumatology 66: S212-S213. | Title |
| 574 | Chartrain, A. G., et al. (2017). "Antiepileptics for Post-Traumatic Seizure Prophylaxis after Traumatic Brain Injury." Curr Pharm Des 23(42): 6428-6441. | Title |
| 575 | Chauzy, A., et al. (2022). "PKPD Modeling of the Inoculum Effect of Acinetobacter baumannii on Polymyxin B in vivo." 13: 842921. | Title |
| 576 | Chay, J., et al. (2023). "Cost-Effectiveness of a Multicomponent Intervention for Hypertension Control in Public Sector Primary Healthcare Services in Singapore." Circulation 148. | Title |
| 577 | Chen, C.-C., et al. (2010). "Effects of lipophilic emulsifiers on the oral administration of lovastatin from nanostructured lipid carriers: Physicochemical characterization and pharmacokinetics." European Journal of Pharmaceutics and Biopharmaceutics 74(3): 474-482. | Title |

| | | |
|---|---|---|
| 578 | Chendo, I. and J. J. Ferreira (2016). "Pimavanserin for the treatment of Parkinson's disease psychosis." Expert Opin Pharmacother 17(15): 2115-2124. | Title |
| 579 | Chhabra, S., et al. (2019). "Comparative Analysis of Calcineurin Inhibitor–Based Methotrexate and Mycophenolate Mofetil–Containing Regimens for Prevention of Graft-versus-Host Disease after Reduced-Intensity Conditioning Allogeneic Transplantation." Biology of Blood and Marrow Transplantation 25(1): 73-85. | Title |
| 580 | Chiaretti, A., et al. (2013). "Current practice and recent advances in pediatric pain management." Eur Rev Med Pharmacol Sci 17 Suppl 1: 112-126. | Title |
| 581 | Chiauzzi, E., et al. (2010). "PainACTION.com: an interactive self-management web site for chronic back pain patients." Pain medicine (Malden, Mass.) 11(2): 307-308. | Title |
| 582 | Chidiac, A. S., et al. (2023). "Paracetamol (acetaminophen) overdose and hepatotoxicity: mechanism, treatment, prevention measures, and estimates of burden of disease." 19(5): 297-317. | Title |
| 583 | Chien, H.-T., et al. (2020). "Is colistin-associated acute kidney injury clinically important in adults? A systematic review and meta-analysis." 55(3): 105889. | Title |
| 584 | Chithra, S., et al. (2022). "Anti-microbial activity, molecular profiling, electronic properties and molecular docking investigations of 5–[1-hydroxy–2–(isopropylamino)ethyl] benzene–1,3–diol." Journal of Molecular Structure 1247: 131299. | Title |
| 585 | Chitnis, T., et al. (2020). "Teriflunomide efficacy and safety in pediatric patients with relapsing forms of MS: interim analysis of open-label terikids trial extension." Multiple sclerosis journal 26(3 SUPPL): 5-6. | Title |
| 586 | Chitnis, T., et al. (2020). "TERIKIDS Study: teriflunomide efficacy and safety in paediatric patients with relapsing forms of MS." Eur J Neurol 27: 46. | Title |
| 587 | Chitnis, T., et al. (2014). "Fingolimod effect on clinical and MRI disease activity in young adult patients with relapsing multiple sclerosis." Multiple sclerosis (Houndmills, Basingstoke, England) 20(1): 208-209. | Title |
| 588 | Chiu, W. Y., et al. (2020). "Racial difference in bioavailability of oral ibandronate between Caucasian and Taiwanese postmenopausal women." Osteoporos Int 31(1): 193-201. | Title |

| | | |
|---|---|---|
| 589 | Chopra, A., et al. (2015). "Comparison of the stanford and indian health assessment questionnaires for disability outcomes in a phase 3, randomized, double-blind, active comparator study of infliximab and biosimilar infliximab BOW15 in rheumatoid arthritis." Arthritis & rheumatology 67. | Title |
| 590 | Choraria, A., et al. (2022). "Chicken egg yolk antibodies (IgY)-based antivenom for neutralization of snake venoms: a review." 41(3): 1018-1029. | Title |
| 591 | Christensen, P., et al. (2012). "Cardiovascular risk factor changes following three different maintenance programs in obese knee osteoarthritis patients after a major weight loss: a randomized controlled trial." Osteoarthritis and cartilage 20: S280. | Title |
| 592 | Christodoulou, G., et al. (2014). "A pilot study to establish reliable telephone-based cognitive testing for the ALS patient population." Amyotrophic lateral sclerosis and frontotemporal degeneration 15: 82-83. | Title |
| 593 | Chukwuma, I. F., et al. (2023). "Bioassay-guided identification of potential Alzheimer's disease therapeutic agents from Kaempferol-Enriched fraction of Aframomum melegueta seeds using in vitro and chemoinformatics approaches." Arabian Journal of Chemistry 16(9): 105089. | Title |
| 594 | Ciancio, S. G., et al. (2016). "Comparison of 3 intranasal mists for anesthetizing maxillary teeth in adults: A randomized, double-masked, multicenter phase 3 clinical trial." The Journal of the American Dental Association 147(5): 339-347.e331. | Title |
| 595 | Ciccia, A. H., et al. (2015). "Identification of neurodevelopmental disabilities in underserved children using telehealth (INvesT): clinical trial study design." Contemporary Clinical Trials 45(Pt B): 226-232. | Title |
| 596 | Cicin, I., et al. (2018). "An open-label, Multinational, Phase IIIb Study to Evaluate Patient and Satisfaction, Safety and Efficacy of Subcutaneous Administration of Trastuzumab in Patients with HER2-Positive Early Breast Cancer (ML28851) in Adjuvant/Neo-Adjuvant Setting." European Journal of Cancer 92: S102-S103. | Title |
| 597 | Cima, R., et al. (2009). "Cost-effectiveness of multidisciplinary management of Tinnitus at a specialized Tinnitus centre." BMC health services research 9. | Title |
| 598 | Cimolai, N. J. C. H. I. (2021). "Passive immunity should and will work for COVID-19 for some patients." 3(2): 47-68. | Title |
| 599 | Clark, J. P. and E. Langston (2003). Ketolides: a new class of antibacterial agents for treatment of community-acquired respiratory tract infections in a primary care setting. Mayo Clin Proc, Elsevier. | Title |
| 600 | Clark, W. G. and J. M. Lipton (1984). "Drug-related heatstroke." Pharmacology & Therapeutics 26(3): 345-388. | Title |

| | | |
|---|---|---|
| 601 | Claus, B. "Role of the pharmacist in a multidisciplinary infectious diseases team at the ICU: an exploratory trial of the potential added value." | Title |
| 602 | Cohen, J. A., et al. (2014). "Generic glatiramer acetate is equivalent to copaxone on efficacy and safety: results of the randomized doubleblind GATE trial in multiple sclerosis." Multiple sclerosis (Houndmills, Basingstoke, England) 20(1): 38-39. | Title |
| 603 | Cohen, J. Y., et al. (2019). "Serum Concentration of Paliperidone Palmitate Administered Every 3 Weeks." Psychopharmacol Bull 49(2): 57-62. | Title |
| 604 | Cohen, S. B., et al. (2006). "Rituximab for rheumatoid arthritis refractory to anti-tumor necrosis factor therapy: results of a multicenter, randomized, double-blind, placebo-controlled, phase III trial evaluating primary efficacy and safety at twenty-four weeks." Arthritis Rheum 54(9): 2793-2806. | Title |
| 605 | Collarile, M., et al. (2012). "Efficacy of biophysical stimulation in improving functional recovery in autologus chondrocyte implantation: a prospective, randomized, controlled study." Journal of orthopaedics and traumatology 13: S104-S105. | Title |
| 606 | Colosimo, C., et al. (1996). "Motor response to acute dopaminergic challenge with apomorphine and levodopa in Parkinson's disease: implications for the pathogenesis of the on-off phenomenon." J Neurol Neurosurg Psychiatry 60(6): 634-637. | Title |
| 607 | Colson, C. R. D. and M. E. De Broe (2005). "Kidney Injury From Alternative Medicines." Advances in Chronic Kidney Disease 12(3): 261-275. | Title |
| 608 | Coly-Mycin, M. "High alert medication." | Title |
| 609 | Comi, G., et al. (2012). "Effect of teriflunomide on lymphocyte and neutrophil levels in patients with relapsing multiple sclerosis: results from the TEMSO study." Multiple sclerosis (Houndmills, Basingstoke, England) 18(4): 460. | Title |
| 610 | Comi, G., et al. (2015). "Teriflunomide safety in subsets of patients with relapsing MS: results from the TEMSO and TOWER studies." Multiple sclerosis (Houndmills, Basingstoke, England) 23(11): 541. | Title |
| 611 | Comi, G., et al. (2013). "Cladribine tablets for the treatment of relapsing-remitting multiple sclerosis." Expert Opin Pharmacother 14(1): 123-136. | Title |
| 612 | Comi, G., et al. (2015). "Teriflunomide efficacy in subsets of patients with relapsing MS: results from TEMSO and TOWER studies." Multiple sclerosis (Houndmills, Basingstoke, England) 23(11): 535-536. | Title |
| 613 | Comi, G., et al. (2015). "Baseline characteristics of patients enrolled in concerto-a study of 0.6 and 1.2 mg/day oral laquinimod for relapsing-remitting multiple sclerosis." Neurology 84. | Title |

| | | |
|---|---|---|
| 614 | Conrad, K. J., et al. (2006). "Randomized trial of psychiatric care with representative payeeship for persons with serious mental illness." Psychiatric services (Washington, D.C.) 57(2): 197-204. | Title |
| 615 | Cook, G. C. (1979). "The D-xylose absorption test in different ethnic groups." Trop Geogr Med 31(1): 93-97. | Title |
| 616 | Cook, S., et al. (2016). "Safety and tolerability of cladribine tablets in patients with relapsing-remitting multiple sclerosis (RRMS): final results from the 120-week Phase IIIb extension trial to the CLARITY study." Swiss medical weekly 146: 78S. | Title |
| 617 | Cooper, D. (2016). "An Investigation into Formulation and Therapeutic Effectiveness of Nanoparticle Drug Delivery for Select Pharmaceutical Agents." | Title |
| 618 | Copelan, E. A., et al. (2015). "Comparison of Outcomes of Allogeneic Transplantation for Chronic Myeloid Leukemia with Cyclophosphamide in Combination with Intravenous Busulfan, Oral Busulfan, or Total Body Irradiation." Biology of Blood and Marrow Transplantation 21(3): 552-558. | Title |
| 619 | Copelan, E. A., et al. (2013). "Better leukemia-free and overall survival in AML in first remission following cyclophosphamide in combination with busulfan compared with TBI." Blood 122(24): 3863-3870. | Title |
| 620 | Corriveau, H., et al. (2011). "Efficacy of supervised Tai Chi exercises compared to physiotherapy program in fall prevention for frail older adults: a randomised trial." Physiotherapy (united kingdom) 97: eS239. | Title |
| 621 | Costa, E., et al. (2006). "Is the GDS-30 better than the GHQ-12 for screening depression in elderly people in the community? The Bambui Health Aging Study (BHAS)." International psychogeriatrics 18(3): 493-503. | Title |
| 622 | Coulter, D. L. (1991). "Carnitine, valproate, and toxicity." J Child Neurol 6(1): 7-14. | Title |
| 623 | Coutts, S. B., et al. (2015). "Thrombolysis for minor ischemic stroke with proven acute symptomatic occlusion using TNK-TPA (TEMPO-1)." International journal of stroke 10: 79-80. | Title |
| 624 | Coutts, S. B., et al. (2015). "Final results of the thrombolysis for minor ischemic stroke with proven acute symptomatic occlusion using TNK-TPA (TEMPO-1) trial." Stroke 46. | Title |
| 625 | Coutts, S. B., et al. (2015). "TEMPO-2: tNK-tPA for minor ischemic stroke with proven acute symptomatic occlusion trial-2." International journal of stroke 10: 36. | Title |
| 626 | Coyle, P. K., et al. (2016). "Improvements in patient-reported treatment satisfaction with teriflunomide: results from the Phase 4 Teri-PRO Study." Multiple sclerosis (Houndmills, Basingstoke, England) 22: 774-775. | Title |

| | | |
|---|---|---|
| 627 | Coyle, P. K., et al. (2016). "Teriflunomide real-world safety profile: results of the Phase 4 Teri-PRO Study." Multiple sclerosis (Houndmills, Basingstoke, England) 22: 308-309. | Title |
| 628 | Coyle, P. K., et al. (2017). "Patient-reported outcomes in relapsing forms of MS: real-world, global treatment experience with teriflunomide from the Teri-PRO study." Multiple sclerosis and related disorders 17: 107-115. | Title |
| 629 | Coyle, P. K., et al. (2019). "Teriflunomide real-world evidence: global differences in the phase 4 Teri-PRO study." Multiple sclerosis and related disorders 31: 157-164. | Title |
| 630 | Cramer, S., et al. (2013). "A single-blind study of the safety, pharmacokinetics, and pharmacodynamics of escalating repeat doses of GSK249320 in patients with stroke." Neurology 80(1). | Title |
| 631 | Cree, B., et al. (2017). "The RADIANCE and SUNBEAM phase 3 studies of ozanimod in relapsing multiple sclerosis: study design and baseline characteristics." Neurology 88(16). | Title |
| 632 | Cree, B. A. C., et al. (2017). "The RADIANCE and SUNBEAM phase 3 studies of ozanimod in relapsing multiple sclerosis: study design and baseline characteristics." Multiple sclerosis (Houndmills, Basingstoke, England) 23: 24-25. | Title |
| 633 | Creese, B., et al. (2019). "The development and use of the assessment of dementia awareness and person-centred care training tool in long-term care." Dementia (14713012) 18(7/8): 3059-3070. | Title |
| 634 | Cresswell, F. V., et al. (2019). "Intensified antibiotic treatment of tuberculosis meningitis." Expert Rev Clin Pharmacol 12(3): 267-288. | Title |
| 635 | Csépány, T. and D. Bereczki (2004). "[Immunomodulatory therapy in multiple sclerosis]." Ideggyogy Sz 57(11-12): 401-416. | Title |
| 636 | Curry, L., et al. (2024). "A guide to developing population files for physiologically-based pharmacokinetic modeling in the Simcyp Simulator." CPT Pharmacometrics Syst Pharmacol 13(9): 1429-1447. | Title |
| 637 | Curtis, J. R., et al. (2015). "Consistency of treatment effects across different high-risk clinical phenotypes in the tofacitinib clinical program." Arthritis & rheumatology 67(no pagination). | Title |
| 638 | Dadashi, M., et al. (2023). "Global prevalence and distribution of antibiotic resistance among clinical isolates of Stenotrophomonas maltophilia: a systematic review and meta-analysis." | Title |
| 639 | Dahmash, E. Z., et al. (2021). "Development of orally dissolving films for pediatric-centric administration of anti-epileptic drug topiramate – A design of experiments (DoE) study." Saudi Pharmaceutical Journal 29(7): 635-647. | Title |

| | | |
|---|---|---|
| 640 | Dai, C., et al. (2017). "Baicalein acts as a nephroprotectant that ameliorates colistin-induced nephrotoxicity by activating the antioxidant defence mechanism of the kidneys and down-regulating the inflammatory response." 72(9): 2562-2569. | Title |
| 641 | Dai, Z. (2014). "Effect of zoledronic acid on bone fusion after lumbar surgery for osteoporotic patients." Spine journal 14(11): S32-S33. | Title |
| 642 | Damanhouri, Z. A., et al. (2023). "A Review of the Impact of Pharmacogenetics and Metabolomics on the Efficacy of Metformin in Type 2 Diabetes." 20(1): 142. | Title |
| 643 | Damian, M. A., et al. (2020). "Pharmacokinetics of dexmedetomidine in infants and children after orthotopic liver transplantation." 130(1): 209-216. | Title |
| 644 | Damlaj, M., et al. (2016). "Fludarabine-Busulfan Reduced-Intensity Conditioning in Comparison with Fludarabine-Melphalan Is Associated with Increased Relapse Risk In Spite of Pharmacokinetic Dosing." Biology of Blood and Marrow Transplantation 22(8): 1431-1439. | Title |
| 645 | Daniels, S. R., et al. (1998). "Left ventricular geometry and severe left ventricular hypertrophy in children and adolescents with essential hypertension." 97(19): 1907-1911. | Title |
| 646 | Danso-Appiah, A., et al. (2022). "Safety of praziquantel in persons with and without schistosomiasis: systematic review and meta-analysis." 2022.2003. 2009.22270839. | Title |
| 647 | Daoudi, W., et al. (2024). "Synthesis, characterization, DFT, ADMET, MD analysis and molecular docking of C-3 functionalized imidazo[1,2-a]pyridine motifs." Journal of Molecular Structure 1312: 138658. | Title |
| 648 | Dapp, U., et al. (2014). "Various aspects of interventions on physical functioning in community dwelling older persons." European geriatric medicine 5: S26-S27. | Title |
| 649 | Darmstadt, G. L., et al. (2008). "Extended-interval dosing of gentamicin for treatment of neonatal sepsis in developed and developing countries." 26(2): 163. | Title |
| 650 | Darwish, H. W., et al. (2021). "Response surface methodology for optimization of micellar-enhanced spectrofluorimetric method for assay of foretinib in bulk powder and human urine." 257: 119811. | Title |
| 651 | Darwish, I. A., et al. (2018). "Development of new ELISA with high sensitivity and selectivity for bioanalysis of bevacizumab: a monoclonal antibody used for cancer immunotherapy." 14(2): 174-181. | Title |

| | | |
|---|---|---|
| 652 | Darwish, I. A., et al. (2023). "A Novel Highly Sensitive Chemiluminescence Enzyme Immunoassay with Signal Enhancement Using Horseradish Peroxidase-Luminol-Hydrogen Peroxide Reaction for the Quantitation of Monoclonal Antibodies Used for Cancer Immunotherapy." 11(4): 245. | Title |
| 653 | Das, M., et al. (2022). "A nano erythropoiesis stimulating agent for the treatment of anemia and associated disorders." iScience 25(9): 105021. | Title |
| 654 | D'Auria, S., et al. (2014). "GPi-DBS in dystonic patients using frameless versus frame-based stereotaxy: a single centre experience." Stereotactic and functional neurosurgery 92: s164. | Title |
| 655 | Davalos, A., et al. (2012). "Final Results of the International Citicoline Trial on acUte Stroke (ICTUS Study)." Cerebrovascular diseases (Basel, Switzerland) 33: 16. | Title |
| 656 | d'Avanzo, N., et al. (2024). "OX26-cojugated gangliosilated liposomes to improve the post-ischemic therapeutic effect of CDP-choline." Drug Deliv Transl Res 14(10): 2771-2787. | Title |
| 657 | Davies, P. S. and B. S. Galer (2004). "Review of lidocaine patch 5% studies in the treatment of postherpetic neuralgia." Drugs 64(9): 937-947. | Title |
| 658 | Davigo, A., et al. (2020). "PIPAC versus HIPEC: cisplatin spatial distribution and diffusion in a swine model." 37(1): 144-150. | Title |
| 659 | Davis, R. J., et al. (2014). "Two-level treatment with total disc replacement versus ACDF: results from a prospective randomized clinical trial with five years follow-up." Spine journal 14(11): S24. | Title |
| 660 | de Almeida, S. M. V., et al. (2020). "COVID-19 therapy: What weapons do we bring into battle?" Bioorganic & Medicinal Chemistry 28(23): 115757. | Title |
| 661 | De Andres, J., et al. (2012). "Botulinum toxin in chronic pain." Regional anesthesia and pain medicine 37(5): E101-E104. | Title |
| 662 | de Bragança, A. C., et al. (2010). "Carbamazepine can induce kidney water absorption by increasing aquaporin 2 expression." Nephrol Dial Transplant 25(12): 3840-3845. | Title |
| 663 | de Leon, J. and F. J. Diaz (2005). "A meta-analysis of worldwide studies demonstrates an association between schizophrenia and tobacco smoking behaviors." Schizophrenia Research 76(2): 135-157. | Title |
| 664 | de Melo Silva, A. J. J. E. and A. B. Research (2018). "Bcl-2 Family Overexpression and Chemoresistance in Acute Myeloid Leukemia." 19(4): 299-309. | Title |
| 665 | de Pauw, B. E., et al. (2002). "Achievements and goals of the EORTC Invasive Fungal Infections Group." European Journal of Cancer 38: 88-93. | Title |

| | | |
|---|---|---|
| 666 | De Quadros, C., et al. (2004). "Tropical and travel-associated diseases." 189(1): S81-S85. | Title |
| 667 | De Seze, J., et al. (2018). "A double-blind placebo-controlled study of satralizumab (SA237), a recycling anti-IL-6 receptor monoclonal antibody, as add-on therapy for neuromyelitis optica spectrum disorder (NMOSD)." Multiple sclerosis journal 24(2): 985-986. | Title |
| 668 | De Seze, J., et al. (2016). "Patient-reported outcomes in the phase III double-blind, placebo-controlled ORATORIO study of ocrelizumab in primary progressive multiple sclerosis." Multiple sclerosis (Houndmills, Basingstoke, England) 22: 677-678. | Title |
| 669 | De Stefano, N., et al. (2014). "Including threshold rates of brain volume loss in the definition of disease-activity-free in multiple sclerosis using fingolimod phase 3 data." Multiple sclerosis (Houndmills, Basingstoke, England) 20(1): 196-197. | Title |
| 670 | de Toffol, B., et al. (2020). "[Interictal psychosis of epilepsy]." Encephale 46(6): 482-492. | Title |
| 671 | de Weerd, H. A., et al. (2024). "Latent space arithmetic on data embeddings from healthy multi-tissue human RNA-seq decodes disease modules." | Title |
| 672 | De Wilde, A. H., et al. (2014). "Screening of an FDA-approved compound library identifies four small-molecule inhibitors of Middle East respiratory syndrome coronavirus replication in cell culture." 58(8): 4875-4884. | Title |
| 673 | Deedat, R. (2022). "How WEIRD are the South African acute pain guidelines? An analysis of the 2015 South African acute pain guidelines." | Title |
| 674 | del Río, N. G., et al. (2019). "Effects of a Gamified Educational Program in the Nutrition of Children with Obesity." Journal of medical systems 43(7): 1-12. | Title |
| 675 | Deleu, D., et al. (1989). "Clinical and pharmacokinetic evaluation of controlled-release levodopa/carbidopa (CR-4) in parkinsonian patients with severe motor fluctuations: a six month follow-up study." Clin Neurol Neurosurg 91(4): 303-309. | Title |
| 676 | Deleu, D., et al. (1989). "Controlled-release carbidopa/levodopa (CR) in parkinsonian patients with response fluctuations on standard levodopa treatment: clinical and pharmacokinetic observations." Neurology 39(11 Suppl 2): 88-92; discussion 95. | Title |
| 677 | Deleu, D., et al. (2019). "Oral disease-modifying therapies for multiple sclerosis in the Middle Eastern and North African (MENA) region: an overview." 35(2): 249-260. | Title |
| 678 | Deng, A., et al. (2021). "Effects of a transitional care program for individuals with limbs disabilities living in a rural community: a randomized controlled trial." Disability and health journal 14(1): 100946. | Title |
| 679 | Deng, J., et al. (2022). "Efficacy of lopinavir–ritonavir combination therapy for the treatment of hospitalized COVID-19 patients: A meta-analysis." 17(3): 169-189. | Title |

| | | |
|---|---|---|
| 680 | Deng, Y., et al. (2022). "Does monitoring total and free polymyxin B1 plasma concentrations predict polymyxin B-induced nephrotoxicity? A retrospective study in critically ill patients." 11(4): 1591-1608. | Title |
| 681 | Denteneer, L., et al. (2015). "Development of clinical prediction rules in chronic nonspecific low back pain patients: study protocol for a randomized controlled trial." European journal of epidemiology 30(8): 967-968. | Title |
| 682 | Desoky, E. S. E., et al. (2002). "Population pharmacokinetics of digoxin in Egyptian pediatric patients: impact of one data point utilization." 9(6): 492-498. | Title |
| 683 | Devanathadesikan Seshadri, V., et al. (2020). "In vitro antioxidant and cytotoxic activities of polyherbal extracts from Vetiveria zizanioides, Trichosanthes cucumerina, and Mollugo cerviana on HeLa and MCF-7 cell lines." Saudi Journal of Biological Sciences 27(6): 1475-1481. | Title |
| 684 | Dharitri, R., et al. (2015). "Stigma of mental illness: an interventional study to reduce its impact in the community." Indian journal of psychiatry 57(2): 165-173. | Title |
| 685 | Dheda, K., et al. (2017). "Outcomes, infectiousness, and transmission dynamics of patients with extensively drug-resistant tuberculosis and home-discharged patients with programmatically incurable tuberculosis: a prospective cohort study." The Lancet Respiratory Medicine 5(4): 269-281. | Title |
| 686 | Dhivya, L. S., et al. (2024). "Halogenated chalcones against Mycobacterium tuberculosis targeting InhA: Rational design, in silico and in vitro evaluation." Microbial Pathogenesis 196: 106945. | Title |
| 687 | Diamond, T., et al. (2019). "Percutaneous Vertebroplasty for Acute Painful Osteoporotic Vertebral Fractures-Benefits Shown in VAPOUR Trial Masked When Pooled With Other Clinical Trials." Journal of bone and mineral research 34(6): 1182-1184. | Title |
| 688 | Díaz-Borjón, A. (2009). "Guidelines for the use of conventional and newer disease-modifying antirheumatic drugs in elderly patients with rheumatoid arthritis." Drugs Aging 26(4): 273-293. | Title |
| 689 | Diep, B. A., et al. (2008). "Emergence of multidrug-resistant, community-associated, methicillin-resistant Staphylococcus aureus clone USA300 in men who have sex with men." 148(4): 249-257. | Title |
| 690 | Dilworth, T. J., et al. (2021). "Vancomycin advanced therapeutic drug monitoring: exercise in futility or virtuous endeavor to improve drug efficacy and safety?" 72(10): e675-e681. | Title |

| | | |
|---|---|---|
| 691 | Dixon, E. R., et al. (2017). "A 3-month safety and efficacy study of travoprost 0.004% ophthalmic solution compared with timolol in pediatric patients with glaucoma or ocular hypertension." Journal of American Association for Pediatric Ophthalmology and Strabismus 21(5): 370-374.e371. | Title |
| 692 | Dixon, L., et al. (1999). "Case managers' and clients' perspectives on a representative payee program." Psychiatric services (Washington, D.C.) 50(6): 781-786. | Title |
| 693 | Djaldetti, R. and E. Melamed (1996). "Levodopa ethylester: a novel rescue therapy for response fluctuations in Parkinson's disease." Ann Neurol 39(3): 400-404. | Title |
| 694 | Djuric, N., et al. (2019). "Disc inflammation and Modic changes show an interaction effect on recovery after surgery for lumbar disc herniation." European spine journal 28(11): 2579-2587. | Title |
| 695 | Doddapaneni, B. S., et al. (2019). "Dual-drug loaded micelle for combinatorial therapy targeting HIF and mTOR signaling pathways for ovarian cancer treatment." Journal of Controlled Release 307: 272-281. | Title |
| 696 | Dodick, D. W., et al. (2018). "Ubrogepant for the acute treatment of migraine: efficacy, safety, tolerability, and functional impact outcomes from a single attack phase III study, ACHIEVE i." Headache 58(8): 1287-1288. | Title |
| 697 | Dodick, D. W., et al. (2018). "Evaluating the impact of ubrogepant, an acute treatment for migraine, on patient-reported functionality and satisfaction: results from a single attack phase iii study, ACHIEVE i." Headache 58(8): 1314-1315. | Title |
| 698 | Dolžan, V. J. P. and i. therapy (2012). "Pharmacogenetics in drug metabolism: role of Phase I Enzymes." 13-80. | Title |
| 699 | Dong, J., et al. (2018). "Design and development of novel hyaluronate-modified nanoparticles for combo-delivery of curcumin and alendronate: fabrication, characterization, and cellular and molecular evidences of enhanced bone regeneration." International Journal of Biological Macromolecules 116: 1268-1281. | Title |
| 700 | Dong, Q., et al. (2014). "Natalizumab decreases progression of disability in RRMS patients as measured by the composite EDSSPlus in AFFIRM." Multiple sclerosis (Houndmills, Basingstoke, England) 20(1): 118. | Title |
| 701 | Donia, T., et al. (2024). "Synergistic anticancer efficacy of polydatin and sorafenib against the MCF-7 breast cancer cell line via inhibiting of PI3K/AKT/mTOR pathway and reducing resistance to treatment." Biochemical and Biophysical Research Communications 739: 150972. | Title |
| 702 | Dooley, M. and A. Markham (1998). "Pramipexole. A review of its use in the management of early and advanced Parkinson's disease." Drugs Aging 12(6): 495-514. | Title |

| | | |
|---|---|---|
| 703 | Dortet, L., et al. (2007). "Nosocomial and hospital-related infections."  60: 470-482. | Title |
| 704 | Douglas, L. R., et al. (1998). "Oral management of the patient with end-stage liver disease and the liver transplant patient." Oral Surgery, Oral Medicine, Oral Pathology, Oral Radiology, and Endodontology 86(1): 55-64. | Title |
| 705 | Douraghi, M., et al. (2016). "Comparative in vitro activity of carbapenems against clinical isolates of Acinetobacter baumannii."  121(2): 401-407. | Title |
| 706 | Dowaidar, M. (2024). "Uptake pathways of cell-penetrating peptides in the context of drug delivery, gene therapy, and vaccine development." Cellular Signalling 117: 111116. | Title |
| 707 | Down, G., et al. (2006). "Clinical pharmacology of Cilomilast." Clin Pharmacokinet 45(3): 217-233. | Title |
| 708 | Downar, J., et al. (2012). "Dorsomedial prefrontal rTMS in major depression: safety, efficacy, and tolerability." Biological psychiatry 71(8): 28S. | Title |
| 709 | Doyle, O., et al. (2014). "Home based educational intervention to improve perinatal outcomes for a disadvantaged community: a randomised control trial." European journal of obstetrics, gynecology, and reproductive biology 180(1): 162-167. | Title |
| 710 | Drusano, G., et al. (2021). "The funnel: a screening technique for identifying optimal two-drug combination chemotherapy regimens."  65(2): 10.1128/aac. 02172-02120. | Title |
| 711 | DRUSANO, G. J. C. (2012). "Plenary lectures, Keynotes and Oral Communications Session 1: Plenary lectures Monday 09–07: 13.30–15.30." 55: 3406-3412. | Title |
| 712 | Duarte, R., et al. (1995). "Interferon-alpha facilitates renal transplantation in hemodialysis patients with chronic viral hepatitis." American Journal of Kidney Diseases 25(1): 40-45. | Title |
| 713 | DuBuske, L. (2007). "Desloratadine for chronic idiopathic urticaria: a review of clinical efficacy." Am J Clin Dermatol 8(5): 271-283. | Title |
| 714 | Dukes, M. N. G. (2003). Sex hormones and related compounds, including hormonal contraceptives. Side Effects of Drugs Annual. J. K. Aronson, Elsevier. 26: 434-456. | Title |
| 715 | Dunayevich, E., et al. (2015). "Efficacy and safety of the glycine transporter type-1 inhibitor AMG 747 for the treatment of negative symptoms associated with schizophrenia." Neuropsychopharmacology 40: S206-S207. | Title |
| 716 | Duraffour, S., et al. (2011). "Camelpox virus." Antiviral Research 92(2): 167-186. | Title |

| | | |
|---|---|---|
| 717 | Durcan, L., et al. (2014). "The effect of exercise on sleep and fatigue in rheumatoid arthritis: a randomised controlled study." Irish journal of medical science 183(3): S110-S111. | Title |
| 718 | Durgin, T., et al. (2015). "Lacosamide monotherapy treatment pathways in epilepsy patients in a us managed care population." Epilepsy currents 15: 328. | Title |
| 719 | Durmus, D., et al. (2013). "A randomized single-blind controlled clinical trial of phonophoresis for the treatment of chronic neck pain." Ann Rheum Dis 72. | Title |
| 720 | Dutta, R. S., et al. (2024). "Enhanced efficacy of β-carotene loaded solid lipid nanoparticles optimized and developed via central composite design on breast cancer cell lines." Heliyon 10(7): e28457. | Title |
| 721 | Eardley, I., et al. (2010). "Pharmacotherapy for Erectile Dysfunction." The Journal of Sexual Medicine 7(1, Part 2): 524-540. | Title |
| 722 | Edan, G., et al. (2015). "Long-term impact of early ms treatment with interferon beta-1B (IFNB-1B): clinical, MRI, employment, and patient-reported outcomes (PROS) at the 11-year follow-up of benefit (benefit 11)." Neurology 84. | Title |
| 723 | Edan, G., et al. (2013). "Long term impact of early initiation of interferon beta-1B after a first clinical event suggestive of multiple sclerosis: additional relapse rate, edss, and msss analyses after 8 years." Neurology 80(1). | Title |
| 724 | Eeg-Olofsson, K., et al. (2016). "Glycaemic control and patient-reported outcome measures (PROMs) in type 1 diabetes." Diabetologia 59(1): S35-S36. | Title |
| 725 | Egelund, E. F., et al. (2016). "Isoniazid and rifampin pharmacokinetics in two Asian elephants (Elephas maximus) infected with Mycobacterium tuberculosis." 47(3): 868-871. | Title |
| 726 | Eid Moustapha, M., et al. (2016). "Technetium-labeled danofloxacin complex as a model for infection imaging." Arabian Journal of Chemistry 9: S1928-S1934. | Title |
| 727 | Eid, T., et al. (2014). "Nurses' Knowledge and Attitudes Regarding Pain in Saudi Arabia." Pain Management Nursing 15(4): e25-e36. | Title |
| 728 | Eida, M. (2010). "Chronic hepatitis C genotype 4 treatment in chronic haemodialysis patients: A retrospective study." Arab Journal of Gastroenterology 11(2): 83-87. | Title |
| 729 | Eissa, I. H., et al. (2023). "Computer-assisted drug discovery (CADD) of an anti-cancer derivative of the theobromine alkaloid inhibiting VEGFR-2." Saudi Pharmaceutical Journal 31(12): 101852. | Title |

| | | |
|---|---|---|
| 730 | Eissa, I. H., et al. (2023). "Design, semi-synthesis, anti-cancer assessment, docking, MD simulation, and DFT studies of novel theobromine-based derivatives as VEGFR-2 inhibitors and apoptosis inducers." Comput Biol Chem 107: 107953. | Title |
| 731 | Eissa, I. H., et al. (2023). "New theobromine derivative as apoptotic anti-triple-negative breast cancer targeting EGFR protein: CADD story." Journal of Molecular Structure 1294: 136336. | Title |
| 732 | Eissenberg, T. and A. Shihadeh (2009). "Waterpipe Tobacco and Cigarette Smoking: Direct Comparison of Toxicant Exposure." American Journal of Preventive Medicine 37(6): 518-523. | Title |
| 733 | Ekinci, B. (2021). "Mediterr J Infect Microb Antimicrob 2021; 10: 53 Erişim: http://dx. doi. org/10.4274/mjima. galenos. 2021.2021. 53." | Title |
| 734 | El fadili, M., et al. (2024). "In-silico investigations of novel tacrine derivatives potency against Alzheimer's disease." Scientific African 23: e02048. | Title |
| 735 | El Fakih, R., et al. (2023). "Successful restoration of checkpoint inhibitors efficacy after allogeneic hematopoietic cell transplant for classic Hodgkin lymphoma patients." Seminars in Oncology 50(3): 76-85. | Title |
| 736 | El Moutaouakil Ala Allah, A., et al. (2024). "Phenytoin from antiepileptic to covid-19: Synthesis, crystal structure, DFT, HSA, MEP and green biological study of phenytoin derivative as potential covid-19 drug candidates." Journal of Molecular Structure 1318: 139430. | Title |
| 737 | El Omari, N., et al. (2023). "Molecular mechanisms underlying the clinical efficacy of panobinostat involve Stochasticity of epigenetic signaling, sensitization to anticancer drugs, and induction of cellular cell death related to cellular stresses." Biomedicine & Pharmacotherapy 164: 114886. | Title |
| 738 | El Omari, N., et al. (2023). "Molecular mechanistic pathways underlying the anticancer therapeutic efficiency of romidepsin." 164: 114774. | Title |
| 739 | El Saftawy, E. A., et al. (2023). "Effects of Lactobacilli acidophilus and/or spiramycin as an adjunct in toxoplasmosis infection challenged with diabetes." Food and Waterborne Parasitology 32: e00201. | Title |
| 740 | Elbadawi, N. E. E. and W. Ibrahim (2017). "Effect of Quinine therapy on Liver Function Parameters in pregnant women infected with Plasmodium falciparum malaria in Gezira state." | Title |
| 741 | Elboim-Gabyzon, M., et al. (2011). "The effect of electrical stimulation to the quadriceps muscle combined with group exercise on pain and function in knee osteoarthritis." Physiotherapy (united kingdom) 97: S3S08. | Title |
| 742 | El-Deeb, I. M., et al. (2010). "Synthesis and antitumor evaluation of novel cyclic arylsulfonylureas: ADME-T and pharmacophore prediction." European Journal of Medicinal Chemistry 45(6): 2516-2530. | Title |

| | | |
|---|---|---|
| 743 | ElDesoky, E., et al. (2008). "Estimation of lithium clearance from routine clinical data in Egyptian bipolar patients. A population pharmacokinetic approach." 46(12): 617-626. | Title |
| 744 | ElFayoumi, R. I., et al. (2019). "The influence of polymorphisms in the drug transporter, ABCB1 on the toxicity of glucocorticoids in Saudi children with acute lymphoblastic leukaemia." Pharmacological Reports 71(1): 90-95. | Title |
| 745 | Elgammal, W. E., et al. (2023). "Rationale design and synthesis of new apoptotic thiadiazole derivatives targeting VEGFR-2: computational and in vitro studies††Electronic supplementary information (ESI) available. See DOI: https://doi.org/10.1039/d3ra07562a." RSC Advances 13(51): 35853-35876. | Title |
| 746 | Elia, N. and M. R. Tramèr (2005). "Ketamine and postoperative pain – a quantitative systematic review of randomised trials." Pain 113(1): 61-70. | Title |
| 747 | Elizebath, R., et al. (2021). "Cenobamate treatment of focal-onset seizures: quality of life and outcome during up to eight years of treatment." Epilepsy & Behavior 116: 107796. | Title |
| 748 | Eljaaly, K., et al. (2019). "Plazomicin: a novel aminoglycoside for the treatment of resistant Gram-negative bacterial infections." 79: 243-269. | Title |
| 749 | Eljaaly, K., et al. (2019). "Contraindicated drug–drug interactions associated with oral antimicrobial agents prescribed in the ambulatory care setting in the United States." Clinical Microbiology and Infection 25(5): 620-622. | Title |
| 750 | Elkady, E. F., et al. (2020). "Sequential liquid-liquid extraction coupled to LC-MS/MS for simultaneous determination of amlodipine, olmesartan and hydrochlorothiazide in plasma samples: Application to pharmacokinetic studies." Microchemical Journal 155: 104757. | Title |
| 751 | Elkady, H., et al. (2024). "New thiazolidine-2,4-diones as potential anticancer agents and apoptotic inducers targeting VEGFR-2 kinase: Design, synthesis, in silico and in vitro studies." Biochimica et Biophysica Acta (BBA) - General Subjects 1868(6): 130599. | Title |
| 752 | El-Kimary, E. I., et al. (2023). "Analytical methodologies for the estimation of oxazolidinone antibiotics as key members of anti-MRSA arsenal: a decade in review." 1-30. | Title |
| 753 | Elkomy, M. H. (2020). "Changing the Drug Delivery System: Does It Add to Non-Compliance Ramifications Control? A Simulation Study on the Pharmacokinetics and Pharmacodynamics of Atypical Antipsychotic Drug." Pharmaceutics 12(4). | Title |
| 754 | Elkomy, M. H., et al. (2019). "Assessment of Ketamine Adult Anesthetic Doses in Pediatrics Using Pharmacokinetic Modeling and Simulations." 39(4): 454-462. | Title |

| | | |
|---|---|---|
| 755 | Elkomy, M. H. J. P. (2020). "Changing the drug delivery system: does it add to non-compliance ramifications control? A simulation study on the pharmacokinetics and pharmacodynamics of atypical antipsychotic drug." 12(4): 297. | Title |
| 756 | Elkotamy, M. S., et al. (2024). "Novel imidazo[2,1-b]thiazoles and imidazo[1,2-a]pyridines tethered with indolinone motif as VEGFR-2 inhibitors and apoptotic inducers: Design, synthesis and biological evaluations." Bioorganic Chemistry 151: 107644. | Title |
| 757 | El-Lateef, H. M. A., et al. (2023). "Design, synthesis and tubulin polymerization inhibition activity of newly synthesized hydrazone-linked to combretastatin analogues as potential anticancer agents." Journal of Molecular Structure 1292: 136190. | Title |
| 758 | El-Malah, A., et al. (2024). "Design, synthesis, and antiproliferative activities of novel substitutedhydrazone/triazolo-linked quinazoline derivatives." 1306: 137822. | Title |
| 759 | El-Masry, S. M., et al. (2023). "Patient-friendly extemporaneous formulation of bisoprolol: application to stability and bioavailability studies." Drug Deliv Transl Res 13(3): 795-810. | Title |
| 760 | Elmedany, S. H., et al. (2019). "Efficacy and safety profile of intravenous tocilizumab versus intravenous abatacept in treating female Saudi Arabian patients with active moderate-to-severe rheumatoid arthritis." Clinical rheumatology 38(8): 2109-2117. | Title |
| 761 | ElMehy, A. E., et al. (2024). "Prognostic value of PGI score compared to poison severity score (PSS) and simplified acute physiology score (SAPS) II as predictors of mortality and other adverse outcomes in acute poisoning with aluminum phosphide." Toxicology Reports 13: 101718. | Title |
| 762 | El-Metwally, S. A., et al. (2023). "Design, synthesis, anti-proliferative evaluation, docking, and MD simulation studies of new thieno[2,3-d]pyrimidines targeting VEGFR-2††Electronic supplementary information (ESI) available. See DOI: https://doi.org/10.1039/d3ra03128d." RSC Advances 13(33): 23365-23385. | Title |
| 763 | Elnosary, M. E., et al. (2024). "Predictions based on inflammatory cytokine profiling of Egyptian COVID-19 with 2 potential therapeutic effects of certain marine-derived compounds." International Immunopharmacology 126: 111072. | Title |
| 764 | Elrggal, M. E., et al. (2023). "Dose optimization of vancomycin in obese patients: A systematic review." Front Pharmacol 14: 965284. | Title |
| 765 | El-Say, K. M., et al. (2023). "Incorporating valsartan in sesame oil enriched self-nanoemulsifying system-loaded liquisolid tablets to improve its bioavailability." Int J Pharm 639: 122966. | Title |
| 766 | El–Sayed, Y. J. J. o. c. p. and therapeutics (1995). "Predictive Performance of four pharmacokinetic methods for calculating digoxin dosage." 20(5): 297-304. | Title |

| | | |
|---|---|---|
| 767 | Elshami, S., et al. (2023). "Examining Pharmacy Alumni's Perceptions of Job Satisfaction, Achievements, and Preparedness: A Mixed-Methods Study." American Journal of Pharmaceutical Education 87(5): 100059. | Title |
| 768 | Elsherbiny, M. E., et al. (2024). "Hyperlipidemia Increases Nalbuphine Brain Accumulation with Multiple Dosing without Affecting Its Analgesic Response—Its Respiratory Depression Potential Should Be Investigated in Future Studies."  17(3): 282. | Title |
| 769 | El-Shoukrofy, M. S., et al. (2019). "Pyrazoles containing thiophene, thienopyrimidine and thienotriazolopyrimidine as COX-2 selective inhibitors: Design, synthesis, in vivo anti-inflammatory activity, docking and in silico chemo-informatic studies." Bioorganic Chemistry 85: 541-557. | Title |
| 770 | El-Tahan, M. R., et al. (2022). "European Association of Cardiothoracic Anesthesiology and Intensive Care Pediatric Cardiac Anesthesia Fellowship Curriculum: First Edition." Journal of Cardiothoracic and Vascular Anesthesia 36(3): 645-653. | Title |
| 771 | el-Yazigi, A. and K. Chaleby (1988). "Steady-state kinetics of doxepin and imipramine in Saudi patients with interethnic comparison." Psychopharmacology (Berl) 95(1): 63-67. | Abstract |
| 772 | Emery, P., et al. (2011). "Combination therapy with adalimumabmethotrexate significantly improved work ability, physical function, fatigue, and other patient-reported outcomes in early rheumatoid arthritis: results from a 26-week analysis." Arthritis Rheum 63(10). | Title |
| 773 | Englezos, K., et al. (2023). "3D printing for personalised medicines: implications for policy and practice." Int J Pharm 635: 122785. | Title |
| 774 | Erdoes, G., et al. (2024). "European Pediatric Cardiac Anesthesia Fellowship Program: A First Proof of Concept." Journal of Cardiothoracic and Vascular Anesthesia 38(5): 1088-1091. | Title |
| 775 | Ereshefsky, L., et al. (1991). "Thiothixene pharmacokinetic interactions: a study of hepatic enzyme inducers, clearance inhibitors, and demographic variables." J Clin Psychopharmacol 11(5): 296-301. | Title |
| 776 | Eriksson, B. I. and O. E. Dahl (2004). "Prevention of venous thromboembolism following orthopaedic surgery: clinical potential of direct thrombin inhibitors." Drugs 64(6): 577-595. | Title |
| 777 | Eriksson, J., et al. (2011). "Sick leave and disability pension in patients with early rheumatoid arthritis randomized to infliximab plus methotrexate or triple therapy: one-year results." Arthritis Rheum 63(10). | Title |
| 778 | Erman, M., et al. (2011). "Armodafinil for the treatment of excessive sleepiness associated with shift work disorder: effect on patient-reported functional impairment, treatment satisfaction, and quality of life." Sleep medicine 12: S28-S29. | Title |

| | | |
|---|---|---|
| 779 | Eryavuz Onmaz, D., et al. (2021). "Development and validation of a sensitive, fast and simple LC-MS / MS method for the quantitation of favipiravir in human serum." Journal of Chromatography B 1176: 122768. | Title |
| 780 | Essa, M. F., et al. (2021). "HLA-matched HSCT using targeted busulfan-based conditioning in children with primary hemophagocytic lymphohistiocytosis." 56(12): 3097-3099. | Title |
| 781 | Evans, T. G. (1993). "LEISHMANIASIS." Infectious Disease Clinics of North America 7(3): 527-546. | Title |
| 782 | Evers, A. W., et al. (2002). "Tailored cognitive-behavioral therapy in early rheumatoid arthritis for patients at risk: a randomized controlled trial." Pain 100(1-2): 141-153. | Title |
| 783 | Evstigneeva, L., et al. (2016). "Effect of exercise on balance and functional mobility in postmenopausal women with vertebral fractures." Osteoporosis international. Conference: world congress on osteoporosis, osteoarthritis and musculoskeletal diseases, WCO-IOF-ESCEO 2016. Malaga spain. Conference start: 20160414. Conference end: 20160417. Conference publication: (var.pagings) 27(1 SUPPL. 1): S520-S521. | Title |
| 784 | Ezzeldin, E., et al. (2020). "UPLC–MS/MS assay for quantification of an inhibitor of kinases (Foretinib) in plasma: Application to a pharmacokinetic study in rats." 28(4): 381-386. | Title |
| 785 | Ezzeldin, E., et al. (2022). "Eco-Friendly, Simple, Fast, and Sensitive UPLC-MS/MS Method for Determination of Pexidartinib in Plasma and Its Application to Metabolic Stability." 27(1): 297. | Title |
| 786 | Fabbrini, G., et al. (2010). "Soluble and controlled-release preparations of levodopa: do we really need them?" J Neurol 257(Suppl 2): S292-297. | Title |
| 787 | Fackrell, K., et al. (2016). "Psychometric properties of the Tinnitus Functional Index (TFI): assessment in a UK research volunteer population." Hearing research 335: 220-235. | Title |
| 788 | Fagiolini, A., et al. (2023). "Role of trazodone in treatment of major depressive disorder: an update." Ann Gen Psychiatry 22(1): 32. | Title |
| 789 | Faidah, H. J. C. P. D. (2024). "An Update on Colistin in Clinical Healthcare Unit in the Kingdom of Saudi Arabia: A Narrative Review." 30(36): 2829-2834. | Title |
| 790 | Faidah, H. S. J. A. i. I. D. (2018). "Colistin Use and Its Resistance in Kingdom of Saudi Arabia: A Narrative Review." 8(4): 255-261. | Title |
| 791 | Faisal, M. S., et al. (2024). "Distribution pattern of UGT1A6 and UGT2B7 gene polymorphism and its impact on the pharmacokinetics of valproic acid and carbamazepine: Prospective genetic association study conducted in Pakistani patients with epilepsy." 892: 147886. | Title |

| | | |
|---|---|---|
| 792 | Faisal, S., et al. (2021). "Knowledge, attitudes, and practices (KAP) towards COVID-19 among university students in Pakistan: a cross-sectional study." 32(4): 681-686. | Title |
| 793 | Faizan, S., et al. (2024). "Anticancer potential of novel symmetrical and asymmetrical dihydropyridines against breast cancer via EGFR inhibition: molecular design, synthesis, analysis and screening††Electronic supplementary information (ESI) available. See DOI: https://doi.org/10.1039/d4ra01424c." RSC Advances 14(16): 11368-11387. | Title |
| 794 | Faizan, S., et al. (2024). "Novel dihydropyrimidines as promising EGFR & HER2 inhibitors: Insights from experimental and computational studies." European Journal of Medicinal Chemistry 275: 116607. | Title |
| 795 | Fakoorziba, M., et al. (2012). "Surrey on some epidemiological factors of cutaneous leishmaniasis in Fars Province of Iran." 16: e158-e159. | Title |
| 796 | Falagas, M. E., et al. (2008). "Fosfomycin: use beyond urinary tract and gastrointestinal infections." 46(7): 1069-1077. | Title |
| 797 | Fan, M., et al. (2018). "DCE-MRI texture analysis with tumor subregion partitioning for predicting Ki-67 status of estrogen receptor-positive breast cancers." J Magn Reson Imaging 48(1): 237-247. | Title |
| 798 | Fan, P. L. and D. M. Meyer (2007). "FDI report on adverse reactions to resin-based materials." International Dental Journal 57(1): 9-12. | Title |
| 799 | Faris, K., et al. (2022). "Genetic blood disorders in Saudi Arabia." 58: 1-26. | Title |
| 800 | Farooq, A., et al. (2024). "Clinical pharmacokinetics of cefpodoxime: a systematic review." Expert Opin Drug Metab Toxicol 20(10): 989-1001. | Title |
| 801 | Farooq, J., et al. (2023). "Polypharmacy in chronic liver disease patients: Implications for disease severity, drug-drug interaction, and quality of life." Saudi Pharmaceutical Journal 31(8): 101668. | Title |
| 802 | Farooq, T., et al. (2024). "Neuroprotective Effect of Brivaracetam and Perampanel Combination on Electrographic Seizures and Behavior Anomalies in Pentylenetetrazole-Kindled Mice." | Title |
| 803 | Fatani, W. K., et al. (2023). "Erlotinib-loaded dendrimer nanocomposites as a targeted lung cancer chemotherapy." 28(9): 3974. | Title |
| 804 | Fatima, M., et al. (2023). "Outcome, risk factors and therapeutic strategies in carbapenem-resistant Gram-negative bacteraemia from Pakistan." 5(3): dlad076. | Title |
| 805 | Fattouche, M., et al. (2024). "Computational studies of pyrimidine derivatives as inhibitors of human σ1 receptor using 3D-QSAR analysis, molecular docking, ADMET properties and DFT investigation." Chemical Physics Impact 8: 100463. | Title |
| 806 | Fauzi, R., et al. (2024). "The Practice of Prolonging Meropenem Infusion: A Narrative Review of Literatures Over the Last Decade: The Practice of Prolonging Meropenem Infusion: A Narrative Review of Literatures Over the Last Decade." 10(1): 114-124. | Title |

| | | |
|---|---|---|
| 807 | Fawzy, M. S., et al. (2018). "Longevity-Related Gene Transcriptomic Signature in Glioblastoma Multiforme." Oxid Med Cell Longev 2018: 8753063. | Title |
| 808 | Fayed, D., et al. (1996). "Efficacy and safety of once-daily amikacin in combination with ceftazidime in critically ill adults with severe gram-negative infections." 8(6): 457-464. | Title |
| 809 | Felmingham, D., et al. (2002). "Surveillance of resistance in bacteria causing community-acquired respiratory tract infections." Clinical Microbiology and Infection 8: 12-42. | Title |
| 810 | Felmingham, D. and R. N. J. J. o. A. C. Grüneberg (2000). "The Alexander Project 1996–1997: latest susceptibility data from this international study of bacterial pathogens from community-acquired lower respiratory tract infections." 45(2): 191-203. | Title |
| 811 | Feng, X., et al. (2022). Biologics and Biosimilars: Drug Discovery and Clinical Applications, CRC Press. | Title |
| 812 | Fernandez, O., et al. (2012). "Historical overview of the rationale for the pharmacological use of prolonged-release fampridine in multiple sclerosis." Expert Rev Clin Pharmacol 5(6): 649-665. | Title |
| 813 | Fernandez, O., et al. (2014). "Efficacy of delayed-release dimethyl fumarate for relapsing-remitting multiple sclerosis (RRMS) in "non-responders" to prior treatment with interferon beta." J Neurol 261: S19-S20. | Title |
| 814 | Fernández-Mayoralas, D. M., et al. (2012). "Treatment with paliperidone in children with behavior disorders previously treated with risperidone: an open-label trial." Clin Neuropharmacol 35(5): 227-230. | Title |
| 815 | Ferrari, M., et al. (2015). "Trojan horses and guided missiles: targeted therapies in the war on arthritis." Nat Rev Rheumatol 11(6): 328-337. | Animal |
| 816 | Ferrarotto, R. and K. A. Gold (2014). "Afatinib in the treatment of head and neck squamous cell carcinoma." Expert Opin Investig Drugs 23(1): 135-143. | Title |
| 817 | Ferreira, L., et al. (2024). "Antibiotics with antibiofilm activity–rifampicin and beyond." 15: 1435720. | Title |
| 818 | Ferrucci, L., et al. (2004). "Designing Randomized, Controlled Trials Aimed at Preventing or Delaying Functional Decline and Disability in Frail, Older Persons: a Consensus Report." Journal of the American Geriatrics Society 52(4): 625-634. | Title |
| 819 | Feunaing, R. T., et al. (2024). "3,3’4-trimethoxy-4’-rutinosylellagic acid and its acetylated derivative: Antioxidant activity and antiproliferative effects on breast cancer cells and molecular docking study." Biomedicine & Pharmacotherapy 179: 117370. | Title |
| 820 | Fibach, E. (2001). "Cell culture and animal models to screen for promising fetal hemoglobin-stimulating compounds." Seminars in Hematology 38(4): 374-381. | Title |

| | | |
|---|---|---|
| 821 | File, T. M., et al. (2002). "Outcome of treatment of respiratory tract infections due to Streptococcus pneumoniae, including drug-resistant strains, with pharmacokinetically enhanced amoxycillin/clavulanate." International Journal of Antimicrobial Agents 20(4): 235-247. | Title |
| 822 | Finazzi, S., et al. (2022). "Tissue penetration of antimicrobials in intensive care unit patients: a systematic review—part I." 11(9): 1164. | Title |
| 823 | Findlay, J., et al. (2016). "KPC enzymes in the UK: an analysis of the first 160 cases outside the North-West region." 71(5): 1199-1206. | Title |
| 824 | Fishbein, M., et al. (2017). "Constipation in toddlers: the untold story." Journal of pediatric gastroenterology and nutrition 65: S145-S146. | Title |
| 825 | Fitzgerald, P. B. (2010). "BL-1020, an oral antipsychotic agent that reduces dopamine activity and enhances GABAA activity, for the treatment of schizophrenia." Curr Opin Investig Drugs 11(1): 92-100. | Title |
| 826 | Fleischmann, R., et al. (2013). "Remission, low disease activity, and associated changes in physical function and radiographic outcomes with subcutaneous abatacept or adalimumab: results from the ample trial." Ann Rheum Dis 72. | Title |
| 827 | Fleischmann, R. M., et al. (2013). "No differences in patient-reported outcomes by methotrexate dose among early rheumatoid arthritis patients treated concomitantly with adalimumab: results from the concerto trial." Arthritis Rheum 65: S574-S575. | Title |
| 828 | Fleischmann, R. M., et al. (2011). "Is long-term etanercept monotherapy ever an option in a patient with moderate to severe rheumatoid arthritis (RA)?" Arthritis Rheum 63(10). | Title |
| 829 | Flockhart, D. A. and J. R. Oesterheld (2000). "Cytochrome P450-Mediated Drug Interactions." Child and Adolescent Psychiatric Clinics of North America 9(1): 43-76. | Title |
| 830 | Floden, L., et al. (2020). "149 Evaluation of Individual Items on the PHQ-9 and SDS in Patients with Treatment-Resistant Depression Treated with Esketamine Nasal Spray." CNS Spectr 25(2): 295-. | Title |
| 831 | Floden, L., et al. (2020). "Evaluation of individual items on the PHQ-9 and SDS in patients with treatment-resistant depression treated with esketamine nasal spray." CNS Spectr 25(2): 295. | Title |
| 832 | Fodor, A., et al. (2020). "Multidrug resistance (MDR) and collateral sensitivity in bacteria, with special attention to genetic and evolutionary aspects and to the perspectives of antimicrobial peptides—a review." 9(7): 522. | Title |
| 833 | Fonseca, R., et al. (2006). "681 DCIS 681 Surgery 682 Diagnosis (imaging) 682 Adjuvant endocrine 682 Adjuvant chemotherapy." 17: U2. | Title |
| 834 | Ford, J. H., et al. (2020). "Migraine Headache Day Response Rates and the Implications to Patient Functioning: an Evaluation of 3 Randomized Phase 3 Clinical Trials of Galcanezumab in Patients With Migraine." Headache 60(10): 2304-2319. | Title |

| | | |
|---|---|---|
| 835 | Foster, C. C., et al. (2017). "Caring for Children with Medical Complexity: perspectives of Primary Care Providers." Journal of pediatrics 182: 275-282.e274. | Title |
| 836 | Fouad, M. N., et al. (2004). "Special populations recruitment for the Women's Health Initiative: successes and limitations." Controlled Clinical Trials 25(4): 335-352. | Title |
| 837 | Fox, E. J., et al. (2011). "Alemtuzumab for multiple sclerosis in patients who have relapsed on therapy: CARE-MS II Baseline Demographics and Disease Characteristics." Multiple sclerosis (Houndmills, Basingstoke, England) 17(10): S417. | Title |
| 838 | Fox, R. J., et al. (2014). "Lymphocyte count reductions with delayed-release dimethyl fumarate: integrated analysis of the phase 2, phase 3, and extension studies." Multiple sclerosis (Houndmills, Basingstoke, England) 20(1): 101-102. | Title |
| 839 | Fox, R. J., et al. (2017). "Comparative effectiveness using a matching-adjusted indirect comparison between delayed-release dimethyl fumarate and fingolimod for the treatment of multiple sclerosis." Curr Med Res Opin 33(2): 175-183. | Title |
| 840 | Fox, R. J., et al. (2013). "Lymphocyte count reductions in relapsing remitting multiple sclerosis (RRMS) patients treated with oral BG-12 (dimethyl fumarate): integrated analysis of the placebocontrolled studies." Multiple sclerosis (Houndmills, Basingstoke, England) 19(11): 469. | Title |
| 841 | Freedman, M., et al. (2016). "Long-term efficacy of teriflunomide in patients recently diagnosed with relapsing forms of MS." Eur J Neurol 23: 666. | Title |
| 842 | Freedman, M. S., et al. (2012). "Efficacy of alemtuzumab in relapsing-remitting multiple sclerosis patients who relapsed on prior therapy (CARE-MSII): subgroup analyses by previous DMT use." Multiple sclerosis (Houndmills, Basingstoke, England) 18(4): 199-200. | Title |
| 843 | Freedman, M. S., et al. (2014). "Evaluating the effect of teriflunomide in subgroups defined by prior treatment: pooled analyses of the phase 3 TEMSO and TOWER studies." Multiple sclerosis (Houndmills, Basingstoke, England) 20(1): 87. | Title |
| 844 | Freedman, M. S., et al. (2015). "Long-term safety of teriflunomide: 2.5-year follow-up in the TOWER extension study in patients with relapsing MS." Multiple sclerosis (Houndmills, Basingstoke, England) 23(11): 761-762. | Title |
| 845 | Freedman, M. S., et al. (2016). "Efficacy of fingolimod on no evidence of disease activity (NEDA)-4 in pooled FREEDOMS and FREEDOMS II studies by subgroups of baseline characteristics." Multiple sclerosis (Houndmills, Basingstoke, England) 22(6): NP21. | Title |
| 846 | Freedman, M. S., et al. (2015). "Efficacy of fingolimod on NEDA-4 in pooled FREEDOMS and FREEDOMS II studies by subgroups of baseline characteristics." Multiple sclerosis (Houndmills, Basingstoke, England) 23(11): 301-302. | Title |

| | | |
|---|---|---|
| 847 | Freye, E. and J. V. Levy (2004). "[Use of opioids in the elderly -- pharmacokinetic and pharmacodynamic considerations]." Anasthesiol Intensivmed Notfallmed Schmerzther 39(9): 527-537. | Title |
| 848 | Friedman, B. W., et al. (2017). "Predicting three-month functional outcomes after an ED visit for acute low back pain." American journal of emergency medicine 35(2): 299-305. | Title |
| 849 | Friedman, B. W., et al. (2017). "Diazepam Is No Better Than Placebo When Added to Naproxen for Acute Low Back Pain." Annals of emergency medicine 70(2): 169-176.e161. | Title |
| 850 | Frith, P., et al. (2013). "Health outcomes in carer-patient dyads of a randomized control trial of carer training for patients receiving long term domiciliary oxygen therapy." American journal of respiratory and critical care medicine 187. | Title |
| 851 | Froim, D. (2005). Cisplatin cytotoxicity associated with tetracycline resistance determinants in Escherichia coli, Massachusetts Institute of Technology. | Title |
| 852 | Fujihara, K., et al. (2023). "METEOROID: a Randomised, Double-Blind, Placebocontrolled, Multicentre Phase 3 Study of Satralizumab in Patients with Myelin Oligodendrocyte Glycoprotein Antibody-associated Disease (MOGAD)." Multiple sclerosis journal 29(7): NP25-NP26. | Title |
| 853 | Fulco, C. E., et al. (2000). Pyridostigmine Bromide. Gulf War and Health: Volume 1. Depleted Uranium, Sarin, Pyridostigmine Bromide, Vaccines, National Academies Press (US). | Title |
| 854 | Gaber, A. A., et al. (2024). "Multi-target rational design and synthesis of novel diphenyl-tethered pyrazolopyrimidines targeting EGFR and topoisomerase II with potential DNA intercalation and apoptosis induction." Bioorganic Chemistry 145: 107223. | Title |
| 855 | Gaertner, J., et al. (2014). "Fingolimod in paediatric multiple sclerosis: design of a double-blind study versus interferon beta-1a IM." J Neurol 261: S93. | Title |
| 856 | Galatage, S. T., et al. (2023). "Oral self-nanoemulsifying drug delivery systems for enhancing bioavailability and anticancer potential of fosfestrol: In vitro and in vivo characterization." European Journal of Pharmaceutics and Biopharmaceutics 193: 28-43. | Title |
| 857 | Gandara, D. R., et al. (1991). "Rapidly alternating radiotherapy and high dose cisplatin chemotherapy in stage IIIB non-small cell lung cancer: Results of a phase I/II study." International Journal of Radiation Oncology*Biology*Physics 20(5): 1047-1052. | Title |
| 858 | Gandhi, D., et al. (2015). "Family-led rehabilitation after stroke in India: the ATTEND trial." International journal of stroke 10: 174. | Title |
| 859 | Ganesh, A., et al. (2018). "Late functional improvement after lacunar stroke: a population-based study." J Neurol Neurosurg Psychiatry 89(12): 1301-1307. | Title |

| | | |
|---|---|---|
| 860 | Gao, K., et al. (2015). "Baseline differences in two depressed populations: major depression with mixed features vs bipolar i depression." Neuropsychopharmacology 40: S310-S311. | Title |
| 861 | Garazzino, S., et al. (2020). "Ceftolozane/tazobactam for treating children with exacerbations of cystic fibrosis due to Pseudomonas aeruginosa: a review of available data."  8: 173. | Title |
| 862 | Garba, M. H., et al. (2015). "In vivo trypanocidal activity of Nymphaea lotus Linn. methanol extract against Trypanosoma brucei brucei." Asian Pacific Journal of Tropical Disease 5(10): 808-812. | Title |
| 863 | García de Yébenes, J., et al. (1997). "[The effect of controlled release of DOPA and carbidopa on clinical response and plasma pharmacokinetics of DOPA in parkinsonian patients]." Neurologia 12(4): 145-156. | Title |
| 864 | García, M. A., et al. (2022). "Predicting Pharmacokinetics of Multisource Acyclovir Oral Products Through Physiologically Based Biopharmaceutics Modeling." Journal of Pharmaceutical Sciences 111(1): 262-273. | Title |
| 865 | Garcia-Bournissen, F., et al. (2009). "Pediatric clinical pharmacology studies in Chagas disease: focus on Argentina." Paediatr Drugs 11(1): 33-37. | Title |
| 866 | Garnock-Jones, K. P. (2014). "Alemtuzumab: a review of its use in patients with relapsing multiple sclerosis." Drugs 74(4): 489-504. | Title |
| 867 | Gartner, J., et al. (2020). "Benefit-risk of ofatumumab in treatment-naïve early relapsing multiple sclerosis patients." Multiple sclerosis journal 26(3 SUPPL): 210. | Title |
| 868 | Gartner, J., et al. (2015). "Disease activity on individual components of “no evidence of disease activity” (NEDA-4) in young adult patients with relapsing-remitting multiple sclerosis." Multiple sclerosis (Houndmills, Basingstoke, England) 23(11): 591. | Title |
| 869 | Garver, D. L. (2006). "Evolution of antipsychotic intervention in the schizophrenic psychosis." Curr Drug Targets 7(9): 1205-1215. | Title |
| 870 | Gatasheh, M. K. (2024). "Identifying key genes against rutin on human colorectal cancer cells via ROS pathway by integrated bioinformatic analysis and experimental validation." Comput Biol Chem 112: 108178. | Title |
| 871 | Gazal, G., et al. (2022). "A comparison of pre-emptive co-amoxiclav, postoperative amoxicillin, and metronidazole for prevention of postoperative complications in dentoalveolar surgery: a randomized controlled trial."  19(7): 4178. | Title |
| 872 | Gazal, G. J. J. o. o. and m. research (2018). "Is articaine more potent than mepivacaine for use in oral surgery?"  9(3). | Title |
| 873 | Geddes, A. M., et al. (2017). Ampicillin and amoxicillin. Kucers' The Use of Antibiotics, CRC Press: 100-135. | Title |
| 874 | Gemignani, M. L., et al. (1999). "BREAST CANCER AND PREGNANCY." Surgical Clinics of North America 79(5): 1157-1169. | Title |

| | | |
|---|---|---|
| 875 | Genovese, M. C., et al. (2015). "Efficacy of sarilumab in moderate and severe rheumatoid arthritis as defined by baseline das28-crp scores: the mobility study." Ann Rheum Dis 74: 486. | Title |
| 876 | Genovese, M. C., et al. (2010). "LY2439821, a humanized anti-interleukin-17 monoclonal antibody, in the treatment of patients with rheumatoid arthritis: A phase I randomized, double-blind, placebo-controlled, proof-of-concept study." Arthritis Rheum 62(4): 929-939. | Title |
| 877 | George, S. Z., et al. (2017). "Biopsychosocial influence on shoulder pain: rationale and protocol for a pre-clinical trial." Contemporary Clinical Trials 56: 9-17. | Title |
| 878 | Ghamri, M., et al. (2020). "Carbazole derivatives containing chalcone analogues targeting topoisomerase II inhibition: First principles characterization and QSAR modelling." Spectrochimica Acta Part A: Molecular and Biomolecular Spectroscopy 242: 118724. | Title |
| 879 | Ghasan Abood Al-Ashoor, S., et al. (2022). "Analysis of OCT1, OCT2 and OCT3 gene polymorphisms among Type 2 diabetes mellitus subjects in Indian ethnicity, Malaysia." Saudi Journal of Biological Sciences 29(1): 453-459. | Title |
| 880 | Ghazy, R. M., et al. (2021). "Evaluation of praziquantel effectivenss after decades of prolonged use in an endemic area in Egypt." 66: 81-90. | Title |
| 881 | Ghelardi, E., et al. (2004). "A mucoadhesive polymer extracted from tamarind seed improves the intraocular penetration and efficacy of rufloxacin in topical treatment of experimental bacterial keratitis." Antimicrob Agents Chemother 48(9): 3396-3401. | Title |
| 882 | Ghezzi, A., et al. (2015). "Effect of fingolimod on no evidence of disease activity (NEDA-4) and safety in young adult patients with relapsing-remitting multiple sclerosis." Neurology 84. | Title |
| 883 | Ghezzi, A., et al. (2015). "Safety and effect of fingolimod on no evidence of disease activity (NEDA-4) in young adult patients with relapsingremitting multiple sclerosis." Eur J Neurol 22: 824. | Title |
| 884 | Ghoneim, R. H., et al. (2021). "Optimizing gentamicin dosing in different pediatric age groups using population pharmacokinetics and Monte Carlo simulation." Ital J Pediatr 47(1): 167. | Title |
| 885 | Ghoneim, R. H. J. S. J. o. C. P. (2023). "Therapeutic Drug monitoring of colistin in critically ill patients: A narrative review." 2(4): 141-146. | Title |
| 886 | Ghosh, P., et al. (2022). "A decade's worth of impact: Dox loaded liposomes in anticancer activity." Materials Today Advances 16: 100313. | Title |
| 887 | Giagulli, V. A., et al. (2011). "Evidence-based medicine update on testosterone replacement therapy (TRT) in male hypogonadism: focus on new formulations." Curr Pharm Des 17(15): 1500-1511. | Title |
| 888 | Giguere, A., et al. (2011). "Treatment of hepatitis C virus infection in patients on maintenance hemodialysis: A single United Arab Emirates center experience." European Journal of Internal Medicine 22(6): 582-586. | Title |

| | | |
|---|---|---|
| 889 | Giha, H. A., et al. (2010). "Clustering of malaria treatment failure (TF) in Daraweesh: Hints for host genetic susceptibility to TF with emphasis on immune-modulating SNPs." Infection, Genetics and Evolution 10(4): 481-486. | Title |
| 890 | Gilks, C. and S. J. y. c. Squire (2001). "Tropical and travel-associated diseases." 20: 525ą530. | Title |
| 891 | Gill, C. M., et al. (2021). "Elevated MICs of susceptible antipseudomonal cephalosporins in non-carbapenemase-producing, carbapenem-resistant Pseudomonas aeruginosa: implications for dose optimization." 65(11): 10.1128/aac. 01204-01221. | Title |
| 892 | Gill, T. M., et al. (2016). "Effect of Structured Physical Activity on Overall Burden and Transitions Between States of Major Mobility Disability in Older Persons: secondary Analysis of a Randomized Trial." Annals of internal medicine 165(12): 833-840. | Title |
| 893 | Gilles, H. M. (1981). "The Liverpool School of Tropical Medicine." Transactions of the Royal Society of Tropical Medicine and Hygiene 75: 21-26. | Title |
| 894 | Gillessen, S., et al. (2024). "Management of Patients with Advanced Prostate Cancer. Report from the 2024 Advanced Prostate Cancer Consensus Conference (APCCC)." European Urology. | Title |
| 895 | Gilligan, C. (2020). "Treatment of radicular pain with epidural clonidine micropellets: a phase 3 prospective, randomized, multicenter, double-blind, sham-controlled study." Pain practice 20(SUPPL 1): 62. | Title |
| 896 | Ginsburg, B. C. J. T. A. j. o. d. and a. abuse (2019). "Strengths and limitations of two cannabis-impaired driving detection methods: a review of the literature." 45(6): 610-622. | Title |
| 897 | Giovannoni, G., et al. (2012). "Analysis of clinical and radiological disease activity-free status in patients with relapsing-remitting multiple sclerosis treated with BG-12: findings from the DEFINE study." J Neurol 259(1): S106. | Title |
| 898 | Giovannoni, G., et al. (2015). "Efficacy of delayed-release dimethyl fumarate in early multiple sclerosis: post-hoc analysis of the phase 3 DEFINE and CONFIRM studies according to baseline cognitive function." Multiple sclerosis (Houndmills, Basingstoke, England) 23(11): 252-253. | Title |
| 899 | Gladman, D., et al. (2013). "Effect of certolizumab pegol on the multiple facets of psoriatic arthritis as reported by patients with and without prior anti-TNF exposure: 24-week patient-reported outcome results of rapid-PSA study." Ann Rheum Dis 72. | Title |
| 900 | Gladman, J., et al. (1995). "Hospital- and home-based rehabilitation after discharge from hospital for stroke patients: analysis of two trials." Age Ageing 24(1): 49-53. | Title |

| | | |
|---|---|---|
| 901 | Glauser, T., et al. (2016). "Evidence-based guideline: treatment of convulsive status epilepticus in children and adults: report of the Guideline Committee of the American Epilepsy Society." 16(1): 48-61. | Title |
| 902 | Gold, R., et al. (2022). "Long-term safety and efficacy of dimethyl fumarate for up to 13 years in patients with relapsing-remitting multiple sclerosis: final ENDORSE study results." Multiple sclerosis (Houndmills, Basingstoke, England) 28(5): 801-816. | Title |
| 903 | Gold, R., et al. (2020). "Safety and efficacy in patients treated with dimethyl fumarate and followed for 13 years: final results of endorse." Multiple sclerosis journal 26(3 SUPPL): 6. | Title |
| 904 | Gold, R., et al. (2019). "Overall safety and efficacy through 10 years of treatment with delayed-release dimethyl fumarate in patients with relapsingremitting multiple sclerosis." Multiple sclerosis journal 25: 772-773. | Title |
| 905 | Gold, R., et al. (2015). "Long-term efficacy of delayed-release dimethyl fumarate in newly diagnosed patients with RRMS: an integrated analysis of define, confirm, and endorse." Neurology 84. | Title |
| 906 | Gold, R., et al. (2015). "Efficacy of delayed-release dimethyl fumarate in early multiple sclerosis: post-hoc analysis of the phase 3 DEFINE and CONFIRM studies according to baseline disability." Multiple sclerosis (Houndmills, Basingstoke, England) 23(11): 263-264. | Title |
| 907 | Gold, R., et al. (2014). "Long-term efficacy of delayed-release dimethyl fumarate in newly diagnosed patients with RRMS: an integrated analysis of DEFINE, CONFIRM, and ENDORSE." Multiple sclerosis (Houndmills, Basingstoke, England) 20(1): 95-96. | Title |
| 908 | Gold, R., et al. (2015). "Long-term efficacy of delayed-release dimethyl fumarate in newly diagnosed patients with RRMS: an integrated analysis of define, confirm, and endorse." Eur J Neurol 22: 27. | Title |
| 909 | Gold, R., et al. (2016). "Stable disability and patient-reported performance outcomes over 48 weeks of teriflunomide treatment: results from the Phase 4 Teri-PRO Study." Multiple sclerosis (Houndmills, Basingstoke, England) 22: 307. | Title |
| 910 | Gold, R., et al. (2012). "Clinical efficacy of BG-12 in relapsing-remitting multiple sclerosis: an integrated analysis of the phase 3 DEFINE and CONFIRM studies." Multiple sclerosis (Houndmills, Basingstoke, England) 18(4): 48-49. | Title |
| 911 | Goldstein, D. B., et al. (2007). "Potential Genetic Causes of Heterogeneity of Treatment Effects." The American Journal of Medicine 120(4, Supplement 1): S21-S25. | Title |
| 912 | Goldstein, J., et al. (2013). "The fixed combination of acetaminophen, acetylsalicylic acid, and caffeine is faster and more effective than ibuprofen for acute treatment of patients with severe migraine." Cephalalgia 33: 37-38. | Title |

| | | |
|---|---|---|
| 913 | Gommans, L. N. M., et al. (2015). "Supervised exercise therapy for intermittent claudication: gender differences following twelve months of follow up." European journal of epidemiology 30(8): 760-761. | Title |
| 914 | Gommoll, C., et al. (2013). "Post hoc analyses of levomilnacipran SR 40, 80, and 120mg on functional outcomes in major depressive disorder." European neuropsychopharmacology 23: S329. | Title |
| 915 | Gordon, C. E., et al. (2008). "Interferon Treatment in Hemodialysis Patients With Chronic Hepatitis C Virus Infection: A Systematic Review of the Literature and Meta-analysis of Treatment Efficacy and Harms." American Journal of Kidney Diseases 51(2): 263-277. | Title |
| 916 | Gottlieb, A., et al. (2017). "Cohort-specific imputation of gene expression improves prediction of warfarin dose for African Americans." Genome Med 9(1): 98. | Title |
| 917 | Gottlieb, A., et al. (2013). "Improvement in psoriasis symptoms and physical functioning with secukinumab compared with placebo and etanercept in subjects with moderate-to-severe plaque psoriasis and psoriatic arthritis: results of a subanalysis from the phase 3 fixture study." Arthritis Rheum 65(12): 3322. | Title |
| 918 | Gottlieb, A. B., et al. (2015). "Ixekizumab improves physical function, quality of life, and work productivity in biologic disease-modifying antirheumatic drug-naive patients with active psoriatic arthritis." Arthritis & rheumatology 67(no pagination). | Title |
| 919 | Gottlieb, A. B., et al. (2014). "Ustekinumab improves physical function, general as well as arthritis-related and skinrelated quality of life and work productivity of patients with active psoriatic arthritis who were naïve to MTX, despite MTX therapy or previously treated with anti-TNF$\alpha$: results from PSUMMIT I and PSUMMIT II." Rheumatology (united kingdom) 53: i142. | Title |
| 920 | Gottlieb, A. B., et al. (2015). "Secukinumab improves skin symptoms and physical functioning compared with ustekinumab in patients with moderate to severe psoriasis with concomitant psoriatic arthritis: subanalysis of a randomized, double blind, parallel-group, active comparator-controlled phase 3b trial." Arthritis & rheumatology 67(no pagination). | Title |
| 921 | Gougnard, T., et al. (2000). "Surveillance thérapeutique des traitements anticancéreux." Immuno-analyse & Biologie Spécialisée 15(4): 258-261. | Title |
| 922 | Gouveia, T. I. A., et al. (2023). "Multi-target analysis of cytostatics in hospital effluents over a 9-month period." Journal of Hazardous Materials 448: 130883. | Title |
| 923 | Gowda, A. C. J. J. o. t. S. C. A. o. S. (2024). "A Comparative Analysis of Chemotherapeutic Administrations in Inhibiting Glioblastoma Multiforme Cellular Growth Utilizing an Integration of Differentiation-Based Growth Models and Pharmacokinetic Equations." 22(2): 3. | Title |

| | | |
|---|---|---|
| 924 | Graciaa, D. S., et al. (2022). "Linezolid exposure is associated with cytopenias in patients treated for multidrug-resistant tuberculosis." 66(9): e00408-00422. | Title |
| 925 | Grahn, B., et al. (2015). "Workup-structured care in physiotherapy practice including workplace interventions to improve work ability in patients with neck and/or back pain." Physiotherapy (united kingdom) 101: eS481-eS482. | Title |
| 926 | Grand'Maison, F., et al. (2018). "Sequencing of high-efficacy disease-modifying therapies in multiple sclerosis: perspectives and approaches." Neural Regen Res 13(11): 1871-1874. | Title |
| 927 | Gras-Martín, L., et al. (2024). "Risk Factors Associated with Antibiotic Exposure Variability in Critically Ill Patients: A Systematic Review." 13(9): 801. | Title |
| 928 | Greaves, A. (2016). "The use of Midazolam as an Intranasal Sedative in Dentistry." SAAD Dig 32: 46-49. | Title |
| 929 | Gréen, H., et al. (2010). "CYP3A activity influences imatinib response in patients with chronic myeloid leukemia: a pilot study on in vivo CYP3A activity." 66: 383-386. | Title |
| 930 | Green, O., et al. (2022). "Recruitment of pregnant women to randomised trials of COVID 19 treatments, and pharmaceutical treatments received outside such trials: A research article." European Journal of Obstetrics & Gynecology and Reproductive Biology 275: 12-16. | Title |
| 931 | Greenwood, B. (1999). "Meningococcal meningitis in Africa." Transactions of the Royal Society of Tropical Medicine and Hygiene 93(4): 341-353. | Title |
| 932 | Grignolo, S., et al. (2017). "Good tolerability of high dose colistin-based therapy in patients with haematological malignancies." 45: 505-511. | Title |
| 933 | Groll, A. H., et al. (2004). Antifungal drugs. Side Effects of Drugs Annual. J. K. Aronson, Elsevier. 27: 276-288. | Title |
| 934 | Gross, A. E. and M. L. J. A. o. P. Bryson (2015). "Oral ribavirin for the treatment of noninfluenza respiratory viral infections: a systematic review." 49(10): 1125-1135. | Title |
| 935 | Grossmann, N. C., et al. (2022). "Neoadjuvant Chemotherapy in Elderly Patients With Upper Tract Urothelial Cancer: Oncologic Outcomes From a Multicenter Study." Clinical Genitourinary Cancer 20(3): 227-236. | Title |
| 936 | Grounds, M. D. and E. M. J. J. o. N. D. Lloyd (2023). "Considering the promise of vamorolone for treating Duchenne muscular dystrophy." (Preprint): 1-18. | Title |
| 937 | Grüneberg, R. N. and D. Felmingham (1996). "Results of the Alexander Project: A continuing, multicenter study of the antimicrobial susceptibility of community-acquired lower respiratory tract bacterial pathogens." Diagn Microbiol Infect Dis 25(4): 169-181. | Title |

| | | |
|---|---|---|
| 938 | Guang-zhi, S. J. C. J. o. C. N. and Neurosurgery (2024). "Chinese expert consensus on the use of sulbactam to treat patients infected with Acinetobacter baumannii in the neurosurgical intensive care unit." 24(6). | Title |
| 939 | Guendouzi, A., et al. (2024). "Identification of tuberculosis inhibitors through QSAR-based virtual screening and molecular dynamics simulation of novel pyrimidine derivatives." Journal of the Indian Chemical Society 101(10): 101298. | Title |
| 940 | Gulson, B. (2008). "Stable lead isotopes in environmental health with emphasis on human investigations." Science of The Total Environment 400(1): 75-92. | Title |
| 941 | Guo, X., et al. (2021). "Efficacy and safety of treating chronic nonspecific low back pain with radial extracorporeal shock wave therapy (rESWT), rESWT combined with celecoxib and eperisone (C + E) or C + E alone: a prospective, randomized trial." Journal of orthopaedic surgery and research 16(1): 705. | Title |
| 942 | Gupta, A., et al. (2021). "Therapeutic approaches for SARS-CoV-2 infection." Methods 195: 29-43. | Title |
| 943 | Gupta, A. K., et al. (1997). "Onychomycosis in children: Prevalence and treatment strategies." Journal of the American Academy of Dermatology 36(3): 395-402. | Title |
| 944 | Gupta, A. K., et al. (2024). "Exploring novel Apalutamide analogues as potential therapeutics for prostate cancer: design, molecular docking investigations and molecular dynamics simulation." 12: 1418975. | Title |
| 945 | Gupta, S., et al. (2020). "Computational screening of promising beta-secretase 1 inhibitors through multi-step molecular docking and molecular dynamics simulations - Pharmacoinformatics approach." Journal of Molecular Structure 1205: 127660. | Title |
| 946 | Gutman, J., et al. (2012). "Combination of probenecid-sulphadoxine-pyrimethamine for intermittent preventive treatment in pregnancy." 11: 1-10. | Title |
| 947 | Habeeb, E., et al. (2023). "Potential Effects of Remdesivir on Tacrolimus Exposure in Transplant Recipients With COVID-19 Infection." Kidney International Reports 8(7): 1315-1322. | Title |
| 948 | Hackett, G. I. (2002). "What Do Patients Expect from Erectile Dysfunction Therapy?" European Urology Supplements 1(8): 4-11. | Title |
| 949 | Hafez, S. H., et al. (2024). "Nursing-based intervention to optimize the self-prescribed and the misuse of antibiotics among mothers of children less than 5 years." International Journal of Africa Nursing Sciences 20: 100644. | Title |
| 950 | Hafsa, H., et al. (2022). "Development and Evaluation of a Physiologically Based Pharmacokinetic Model of Labetalol in Healthy and Diseased Populations." Pharmaceutics 14(11). | Title |

| | | |
|---|---|---|
| 951 | Haider, M., et al. (2024). "Erlotinib and curcumin-loaded nanoparticles embedded in thermosensitive chitosan hydrogels for enhanced treatment of head and neck cancer." Int J Pharm 666: 124825. | Title |
| 952 | Haikal, A. and A. R. Ali (2024). "Chemical composition and toxicity studies on Lantana camara L. flower essential oil and its in silico binding and pharmacokinetics to superoxide dismutase 1 for amyotrophic lateral sclerosis (ALS) therapy††Electronic supplementary information (ESI) available. See DOI: https://doi.org/10.1039/d4ra04281f." RSC Advances 14(33): 24250-24264. | Title |
| 953 | Hajibeygi, R., et al. (2022). "Effect of a diet based on Iranian traditional medicine on inflammatory markers and clinical outcomes in COVID-19 patients: A double-blind, randomized, controlled trial." European Journal of Integrative Medicine 55: 102179. | Title |
| 954 | Hajjar, J., et al. (2022). "Selected Abstracts from the 13 th Annual Meeting of the Clinical Immunology Society: 2022 Annual Meeting: Immune Deficiency and Dysregulation North American Conference." 1(42): 42. | Title |
| 955 | Hakeam, H. A. and N. Al-Sanea (2017). "Effect of major gastrointestinal tract surgery on the absorption and efficacy of direct acting oral anticoagulants (DOACs)." J Thromb Thrombolysis 43(3): 343-351. | Title |
| 956 | Hakeam, H. A., et al. (2018). "Incidence of leukopenia and thrombocytopenia with cisplatin plus mitomycin-c versus melphalan in patients undergoing cytoreductive surgery (CRS) and hyperthermic intraperitoneal chemotherapy (HIPEC)." 81: 697-704. | Title |
| 957 | Hakeam, H. A., et al. (2022). "Treatment of multidrug-resistant Pseudomonas aeruginosa bacteremia using ceftolozane-tazobactam-based or colistin-based antibiotic regimens: A multicenter retrospective study." Journal of Infection and Public Health 15(10): 1081-1088. | Title |
| 958 | Hakimullah, et al. (2024). "Integrated insights into the synthesis and biological significances of novel benzofuran based oxadiazole/thiadiazole derivatives: A comprehensive computational and experimental study." Journal of Molecular Structure 1314: 138726. | Title |
| 959 | Halaseh, R. M., et al. (2024). "Risk Factors and Outcomes Associated With Re-Intubation Secondary to Respiratory Failure in Patients With COVID-19 ARDS." 69(1): 50-60. | Title |
| 960 | Halayal, R. Y., et al. (2024). "Exploring the therapeutic mechanism of potential phytocompounds from Kalanchoe pinnata in the treatment of diabetes mellitus by integrating network pharmacology, molecular docking and simulation approach." Saudi Pharmaceutical Journal 32(5): 102026. | Title |
| 961 | Halder, J., et al. (2022). "Nanotherapeutics approaches to overcome P-glycoprotein-mediated multi-drug resistance in cancer." Nanomedicine: Nanotechnology, Biology and Medicine 40: 102494. | Title |

| | | |
|---|---|---|
| 962 | Hallidy, M., et al. (2015). "Short-term effects of mckenzie vs. motor control approach for patients with chronic lowback pain and a derangement classification." Physiotherapy (united kingdom) 101: eS506-eS507. | Title |
| 963 | Halwani, A. A., et al. (2023). "Current status and vision of local pharmaceutical industries in Saudi Arabia: The focus on nanomedicines." Saudi Pharmaceutical Journal 31(8): 101674. | Title |
| 964 | Halwani, A. A. J. P. (2022). "Development of pharmaceutical nanomedicines: from the bench to the market." 14(1): 106. | Title |
| 965 | Hamed, S. A. (2006). "Drug evaluation: PTC-124--a potential treatment of cystic fibrosis and Duchenne muscular dystrophy." IDrugs 9(11): 783-789. | Title |
| 966 | Hamed, S. A. and T. Nabeshima (2005). "The High Atherosclerotic Risk Among Epileptics: the Atheroprotective Role of Multivitamins." Journal of Pharmacological Sciences 98(4): 340-353. | Title |
| 967 | Hampton, J. R. (1996). "Mega-trials and equivalence trials: experience from the INJECT study." European heart journal 17 Suppl E(SUPPL. E): 28-34. | Title |
| 968 | Hamza, E., et al. (2023). "Successful treatment of carbapenem-resistant Klebsiella pneumoniae meningitis with combination therapy of meropenem and amikacin." | Title |
| 969 | Han, C., et al. (2015). "A comparison of EQ5D index from the UK, US, and Japan preference weights model, and mapping algorithm from clinical outcomes in patients with rheumatoid arthritis: results of from simponi aria study." Ann Rheum Dis 74: 225-226. | Title |
| 970 | Han, C., et al. (2014). "Impact of golimumab on physical function and employability of patients with rheumatoid arthritis: 5-year data from 3 phase iii clinical trials." Ann Rheum Dis 73. | Title |
| 971 | Han, C., et al. (2008). "Fluoxetine versus sertraline in the treatment of patients with undifferentiated somatoform disorder: A randomized, open-label, 12-week, parallel-group trial." Progress in Neuro-Psychopharmacology and Biological Psychiatry 32(2): 437-444. | Title |
| 972 | Hang, W., et al. (2024). "The efficacy and safety of hydroxychloroquine at different doses and courses for COVID-19 prevention: a systematic review and network meta-analysis." (just-accepted). | Title |
| 973 | Hanif, M., et al. (2021). "Formulation, characterization, and pharmacokinetic evaluation of Ivabradine-Nebivolol co-encapsulated lipospheres." Journal of Molecular Liquids 344: 117704. | Title |
| 974 | Hankin, C. S., et al. (2011). "Agitation in the inpatient psychiatric setting: a review of clinical presentation, burden, and treatment." J Psychiatr Pract 17(3): 170-185. | Title |

| | | |
|---|---|---|
| 975 | Hanna, P. A., et al. (2024). "Development of a novel intramuscular liposomal injection for advanced meloxicam delivery: Preparation, characterization, in vivo pharmacokinetics, pharmacodynamics, and pain assessment in an orthopedic pain model." International Journal of Pharmaceutics: X 8: 100284. | Title |
| 976 | Hanssens, Y., et al. (2006). "Efficacy and tolerability of antiepileptic drugs in an Omani epileptic population." Clin Neurol Neurosurg 108(6): 532-538. | Title |
| 977 | Haque, S., et al. (2022). "CURRENT SCENARIO AND FUTURE ORCHESTRATIONS IN THE BATTLE AGAINST DYSLIPIDEMIA: REVIEW ON NOVEL HYPOLIPIDEMIC DRUGS."  9(7): 162-185. | Title |
| 978 | Hara, R., et al. (2019). "Intravenous abatacept in Japanese patients with polyarticular-course juvenile idiopathic arthritis: results from a phase III open-label study." Pediatr Rheumatol Online J 17(1): 17. | Title |
| 979 | Haraoui, B., et al. (2011). "Change in CRP at 12 weeks predicts the risk of rapid radiographic progression at two years in methotrexate-treated patients with early rheumatoid arthritis." Rheumatology 50: iii125-iii126. | Title |
| 980 | Hark, L. A., et al. (2012). "A novel home-based, behavioral intervention to improve access to diabetes eye care." Diabetes 61: A578. | Title |
| 981 | Haroun, L. A. K. (2022). The Relationship between Cytomegalovirus Infection and Cardiovascular Diseases in Patients attending Sudan Heart Centre, Sudan University of Science & Technology. | Title |
| 982 | Hartung, H., et al. (2014). "Fingolimod (fty720) oral for the treatment of chronic inflammatory demyelinating polyradiculoneuropathy (CIDP): study design of the phase 3 forcidp trial." Neurology 82(10). | Title |
| 983 | Hartung, H. P., et al. (2012). "Disability outcomes for alemtuzumab in RRMS patients who relapsed on prior therapy: CARE-MS II." J Neurol 259(1): S47-S48. | Title |
| 984 | Harvey, P., et al. (2014). "Effects of levomilnacipran er on cognition and functioning in patients with major depressive disorder: post hoc analysis of a phase 3 trial." Neuropsychopharmacology 39: S361-S362. | Title |
| 985 | Hasan, T., et al. (2019). "Rapid and sustained improvements in patient-reported outcomes with ixekizumab in biologic-naïve and TNF-inadequate responder patients with psoriatic arthritis." Swiss medical weekly 149: 17S. | Title |
| 986 | Hasan, T., et al. (2024). "Mechanisms of Castanopsis tribuloides targeting $\alpha$-glucosidase for the management of type-2 diabetes: Experimental and computational approaches." Process Biochemistry 145: 41-49. | Title |

| | | |
|---|---|---|
| 987 | Haseeb, A., et al. (2022). "Trimethoprim-sulfamethoxazole (bactrim) dose optimization in Pneumocystis jirovecii pneumonia (PCP) management: a systematic review." 19(5): 2833. | Title |
| 988 | Haseeb, A., et al. (2022). "Dose optimization of β-lactams antibiotics in pediatrics and adults: A systematic review." 13: 964005. | Title |
| 989 | Haseeb, A., et al. (2021). "Dose optimization of colistin: A systematic review." 10(12): 1454. | Title |
| 990 | Haseeb, M. T., et al. (2024). "Flaxseed (Linum usitatissimum) mucilage: A versatile stimuli–responsive functional biomaterial for pharmaceuticals and healthcare." International Journal of Biological Macromolecules 278: 134817. | Title |
| 991 | Hashiguchi, Y., et al. (2023). "Population pharmacokinetics and AUC-guided dosing of tobramycin in the treatment of infections caused by glucose-nonfermenting gram-negative bacteria." 45(5): 400-414. e402. | Abstract |
| 992 | Haslund-Krog, S. S., et al. (2019). "Pharmacokinetics of prednisolone in children: an open-label, randomised, two-treatment cross-over trial investigating the bioequivalence of different prednisolone formulations in children with airway disease." 3(1). | Title |
| 993 | Hassan, S., et al. (2022). "Knowledge, attitudes and practices of Egyptian healthcare professionals toward therapeutic drug monitoring service as a principal component of personalized medicine." 19(6): 509-521. | Title |
| 994 | Hassanein, M., et al. (2020). "A real-world study in patients with type 2 diabetes mellitus treated with gliclazide modified-release during fasting: DIA-RAMADAN." Diabetes Research and Clinical Practice 163: 108154. | Title |
| 995 | Hatoum, H. T., et al. (2013). "Assessment of the health-related quality of life impact of euflexxa (1% SODIUM HYALURONATE) using the short form (SF)-36 data collected in a randomized clinical trial." Osteoarthritis and cartilage 21: S246. | Title |
| 996 | Hauser, S., et al. (2015). "Baseline demographics and disease characteristics from OPERA I and II, two phase III trials evaluating ocrelizumab in patients with relapsing multiple sclerosis." Eur J Neurol 22: 751. | Title |
| 997 | Hauser, S. L., et al. (2015). "Efficacy and safety of ocrelizumab in relapsing multiple sclerosis-results of the interferon-beta-1a-controlled, doubleblind, Phase III OPERA I and II studies." Multiple sclerosis (Houndmills, Basingstoke, England) 23(11): 61-62. | Title |
| 998 | Havrdova, E., et al. (2015). "Efficacy and safety of delayed-release dimethyl fumarate in relapsing-remitting multiple sclerosis patients with cardiovascular disease: integrated analysis of the phase 3 DEFINE and CONFIRM studies." Multiple sclerosis (Houndmills, Basingstoke, England) 23(11): 551-552. | Title |
| 999 | Havrdova, E., et al. (2013). "BG-12 (dimethyl fumarate) treatment for relapsing-remitting multiple sclerosis (RRMS) increases the proportion of patients free of measured clinical and neuroradiologic disease activity in the phase 3 studies." Neurology 80(1). | Title |

| | | |
|---|---|---|
| 1000 | Havrdova, E., et al. (2015). "Association between no evidence of disease activity (NEDA) and long-term clinical efficacy of delayed-release dimethyl fumarate (DMF) in patients with relapsing-remitting multiple sclerosis from the phase 3 study, ENDORSE." Multiple sclerosis (Houndmills, Basingstoke, England) 23(11): 256-257. | Title |
| 1001 | Havrdova, E., et al. (2012). "Relapses requiring intravenous steroid use and MS-related hospitalisations: findings from the phase 3 DEFINE and CONFIRM studies." Multiple sclerosis (Houndmills, Basingstoke, England) 18(4): 204-205. | Title |
| 1002 | Havrdova, E., et al. (2013). "Effect of BG-12 (dimethyl fumarate) on freedom from measured clinical and neuroradiological disease activity over time in patients with relapsing remitting multiple sclerosis: results from the phase 3 studies." Multiple sclerosis (Houndmills, Basingstoke, England) 19(11): 211-212. | Title |
| 1003 | Havrdova, E., et al. (2014). "Delayed-release dimethyl fumarate and freedom from measured clinical and neuroradiologic disease activity in relapsing-remitting multiple sclerosis (RRMS) patients: integrated analysis of DEFINE and CONFIRM." Eur J Neurol 21: 459. | Title |
| 1004 | Havrdova, E., et al. (2012). "Clinical and neuroimaging outcomes with BG-12 treatment in CONFIRM (comparator and an oral fumarate in relapsing-remitting multiple sclerosis), a multicenter, randomized, placebocontrolled, phase-3 study." Eur J Neurol 19: 87. | Title |
| 1005 | Hayee, R., et al. (2024). "Levofloxacin loaded chitosan and poly-lactic-co-glycolic acid nano-particles against resistant bacteria: Synthesis, characterization and antibacterial activity." Journal of Infection and Public Health 17(5): 906-917. | Title |
| 1006 | Hayward, K., et al. (2011). "Use of the smart arm with outcome-triggered electrical stimulation to retrain reaching after stroke during acute inpatient rehabilitation: pilot RCT." Physiotherapy (united kingdom) 97: eS465. | Title |
| 1007 | He, N. (1998). Drug metabolism studies on cytochrome P450 2D6: Polymorphism of dextromethorphan metabolism in African Americans and the effects of H1 receptor antihistamine agents on burforalol metabolism, Meharry Medical College. | Title |
| 1008 | He, T., et al. (2022). "Bibliometric and visual analysis of nephrotoxicity research worldwide."  13: 940791. | Title |
| 1009 | Heeney, M. M., et al. (2019). "Ticagrelor versus placebo for the reduction of vaso-occlusive crises in pediatric sickle cell disease: Rationale and design of a randomized, double-blind, parallel-group, multicenter phase 3 study (HESTIA3)." Contemporary Clinical Trials 85: 105835. | Title |
| 1010 | Hefnawy, M., et al. (2020). "Effective quantification of ravidasvir (an NS5A inhibitor) and sofosbuvir in rat plasma by validated LC-MS/MS method and its application to pharmacokinetic study." Arabian Journal of Chemistry 13(11): 8160-8171. | Title |

| | | |
|---|---|---|
| 1011 | Hefny, S. M., et al. (2024). "Discovery and Mechanistic Studies of Dual-Target Hits for Carbonic Anhydrase IX and VEGFR-2 as Potential Agents for Solid Tumors: X-ray, In Vitro, In Vivo, and In Silico Investigations of Coumarin-Based Thiazoles." Journal of Medicinal Chemistry 67(9): 7406-7430. | Title |
| 1012 | Heimans, L., et al. (2012). "Patient reported outcomes in early arthritis patients." Arthritis Rheum 64: S1103-S1104. | Title |
| 1013 | Henderson, A., et al. (2021). "Association between minimum inhibitory concentration, beta-lactamase genes and mortality for patients treated with piperacillin/tazobactam or meropenem from the MERINO study." 73(11): e3842-e3850. | Title |
| 1014 | Henthorn, T. K. and M. van Velzen (2020). "Ketamine pharmacokinetics: a systematic review of the literature, meta-analysis and population analysis." | Title |
| 1015 | Hernandez, L., et al. (2015). "Clinical efficacy of delayed-release dimethyl fumarate in Hispanic patients with relapsing-remitting multiple sclerosis: integrated analysis of define and confirm." Journal of the neurological sciences 357: e306. | Title |
| 1016 | Hernandez-Vargas, E. A. and J. X. Velasco-Hernandez (2020). "In-host Mathematical Modelling of COVID-19 in Humans." Annual Reviews in Control 50: 448-456. | Title |
| 1017 | Herrick, A. L., et al. (2020). "Clinical trial protocol: pRednisolone in early diffuse cutaneous Systemic Sclerosis (PRedSS)." Journal of scleroderma and related disorders. | Title |
| 1018 | Herron, E. K., et al. (2019). "Effect of case study versus video simulation on nursing students' satisfaction, self-confidence, and knowledge: a quasi-experimental study." Nurse education today 79: 129-134. | Title |
| 1019 | Hervey, P. S. and S. J. Keam (2006). "Abatacept." BioDrugs 20(1): 53-61; discussion 62. | Title |
| 1020 | Heybeli, C., et al. (2021). "Acute kidney injury following colistin treatment in critically-ill patients: may glucocorticoids protect?" 33(2): 85-94. | Title |
| 1021 | Hida, A., et al. (2023). "Participation of a pharmacist in patient care for critically ill Covid-19 patients in Morocco: A prospective study." Le Pharmacien Clinicien 58(4): 329-338. | Title |
| 1022 | Hiemke, C., et al. (2011). "AGNP Consensus Guidelines for Therapeutic Drug Monitoring in Psychiatry: Update 2011." Pharmacopsychiatry 44(6): 195-235. | Title |
| 1023 | Hiemke, C., et al. (2018). "Consensus Guidelines for Therapeutic Drug Monitoring in Neuropsychopharmacology: Update 2017." Pharmacopsychiatry 51(1-02): 9-62. | Title |

| | | |
|---|---|---|
| 1024 | Hill, J. (2012). "Can we choose the best treatment for back pain patients?" Rheumatology (united kingdom) 51: iii11. | Title |
| 1025 | Hisey, M. S., et al. (2014). "Sagittal alignment of one-level TDR and acdf patients: an analysis of patient outcomes from a randomized, prospective, clinical trial." Spine journal 14(11): S124-S125. | Title |
| 1026 | Hisey, M. S., et al. (2014). "One-level treatment with total disc replacement and ACDF: five-year results from a prospective randomized clinical trial." Spine journal 14(11): S25. | Title |
| 1027 | Hochman, S., et al. (2015). "Clinical efficacy of delayed-release dimethyl fumarate in Asian patients with relapsing-remitting multiple sclerosis: integrated analysis of define and confirm." Journal of the neurological sciences 357: e306-e307. | Title |
| 1028 | Hodes, C. (2014). "Scopes: From Stethoscope to Microscope-To the Scope of the College, Fall 2014; 2013-2014 Annual Report." | Title |
| 1029 | Hoenigl, M. and R. J. C. p. d. Krause (2013). "Antifungal therapy of aspergillosis of the central nervous system and Aspergillus endophthalmitis." 19(20): 3648-3668. | Title |
| 1030 | Hofstetter, A. and R. Waidelich (2003). "15th World Congress International Society for Laser Surgery and Medicine (ISLSM), 14th Congress International Nd:YAG Laser Society, and 14th Annual Meeting Deutsche Gesellschaft für Lasermedizin (DGLMe.V.)." Medical Laser Application 18(2): 136-184. | Title |
| 1031 | Holkenborg, J., et al. (2013). "Closed reduction of distal radius fractures: is finger trap traction superior to manual traction?" Annals of emergency medicine 62(4): S66. | Title |
| 1032 | Holzer, P. (2012). "Non-analgesic effects of opioids: management of opioid-induced constipation by peripheral opioid receptor antagonists: prevention or withdrawal?" Curr Pharm Des 18(37): 6010-6020. | Title |
| 1033 | Honeycutt, W., et al. (2015). "Teriflunomide significantly increased time to first relapse in Temso, tower and topic." Neurology 84. | Title |
| 1034 | Hong, B.-L., et al. (2020). "A Systematic Review and Meta-analysis of Isoniazid Pharmacokinetics in Healthy Volunteers and Patients with Tuberculosis." Clin Ther 42(11): e220-e241. | Title |
| 1035 | Horita, Y., et al. (2018). "Evaluation of the adequacy of WHO revised dosages of the first-line antituberculosis drugs in children with tuberculosis using population pharmacokinetic modeling and simulations." 62(9): 10.1128/aac. 00008-00018. | Title |
| 1036 | Horlenko, O., et al. (2022). "E-Poster Viewing-Neonatology AS02-15. Infectious diseases. Markers of inflammatory response in infants with intrauterine infection from mothers with identified torch infection." | Title |

| | | |
|---|---|---|
| 1037 | Hosford, D., et al. (2009). "Mecamylamine improved disability of depressed subjects who did not respond adequately to citalopram." European neuropsychopharmacology 19: S404-S405. | Title |
| 1038 | Howard, J. F., Jr., et al. (2021). "Zilucoplan: An Investigational Complement C5 Inhibitor for the Treatment of Acetylcholine Receptor Autoantibody-Positive Generalized Myasthenia Gravis." Expert Opin Investig Drugs 30(5): 483-493. | Title |
| 1039 | Hoy, S. M. (2015). "Peginterferon beta-1a: a review of its use in patients with relapsing-remitting multiple sclerosis." CNS Drugs 29(2): 171-179. | Title |
| 1040 | Hsin, C.-h., et al. (2020). "Combinations of common SNPs of the transporter gene ABCB1 influence apparent bioavailability, but not renal elimination of oral digoxin." 10(1): 12457. | Title |
| 1041 | Hsu, B., et al. (2013). "Sirukumab, a human anti-IL-6 monoclonal antibody, improves physical function in patients with active ra despite methotrexate therapy: results from a 2-part, proof-of-concept, dose-ranging, randomized, double-blind, placebo-controlled, phase 2 study." Annals of the rheumatic disease 71. | Title |
| 1042 | Hu, J., et al. (2023). "Side-effects of hyperthermic intraperitoneal chemotherapy in patients with gastrointestinal cancers." 11: e15277. | Title |
| 1043 | Hubbard, J. W., et al. (1993). "Metabolism of phenothiazine and butyrophenone antipsychotic drugs. A review of some recent research findings and clinical implications." Br J Psychiatry Suppl(22): 19-24. | Title |
| 1044 | Hubbard, R. E., et al. (2013). "Medication prescribing in frail older people." Eur J Clin Pharmacol 69(3): 319-326. | Title |
| 1045 | Hubble, J. P. (1999). "Novel drugs for Parkinson's disease." Med Clin North Am 83(2): 525-536. | Title |
| 1046 | Hübner, M., et al. (2020). "Guidelines for Perioperative Care in Cytoreductive Surgery (CRS) with or without hyperthermic IntraPEritoneal chemotherapy (HIPEC): Enhanced recovery after surgery (ERAS®) Society Recommendations — Part I: Preoperative and intraoperative management." European Journal of Surgical Oncology 46(12): 2292-2310. | Title |
| 1047 | Hudgens, S., et al. (2020). "175 Determining Meaningful Change in Depression Symptoms Assessed with PHQ-9 and SDS in Treatment-resistant Depression Trials of Esketamine Nasal Spray." CNS Spectr 25(2): 311-312. | Title |
| 1048 | Hudgens, S., et al. (2020). "Determining meaningful change in depression symptoms assessed with PHQ-9 and SDS in treatment-resistant depression trials of esketamine nasal spray." CNS Spectr 25(2): 311-312. | Title |
| 1049 | Hudu, S. A., et al. (2023). "Trofinetide for Rett syndrome: highlights on the development and related inventions of the first USFDA-approved treatment for rare pediatric unmet medical need." 12(15): 5114. | Title |

| | | |
|---|---|---|
| 1050 | Huebner, J. L., et al. (2012). "Inflammatory biomarkers of OA, IL-6 and leptin are modifiable in overweight/obese OA patients with a protocol that combines training in pain coping skills and weight management." Osteoarthritis and cartilage 20: S38. | Title |
| 1051 | Hughes, R. A. C., et al. (2013). "Oral fingolimod (FTY720) for the treatment of chronic inflammatory demyelinating polyradiculoneuropathy (CIDP): study design of the phase 3 forcidp trial." Journal of the peripheral nervous system 18: S48-S49. | Title |
| 1052 | Huppke, B., et al. (2019). "Association of Obesity With Multiple Sclerosis Risk and Response to First-line Disease Modifying Drugs in Children." JAMA Neurol 76(10): 1157-1165. | Title |
| 1053 | Huraib, S., et al. (1999). "Interferon-$\alpha$ in chronic hepatitis C infection in dialysis patients." American Journal of Kidney Diseases 34(1): 55-60. | Title |
| 1054 | Hurst, S., et al. (2002). "Methotrexate, hydroxychloroquine, and intramuscular gold in rheumatoid arthritis: relative area under the curve effectiveness and sequence effects." J Rheumatol 29(8): 1639-1645. | Title |
| 1055 | Huskisson, E. C. (2008). "Glucosamine and chondroitin for osteoarthritis." J Int Med Res 36(6): 1161-1179. | Title |
| 1056 | Hussain, A., et al. (2021). "Biocompatible solvent selection based on thermodynamic and computational solubility models, in-silico GastroPlus prediction, and cellular studies of ketoconazole for subcutaneous delivery." Journal of Drug Delivery Science and Technology 65: 102699. | Title |
| 1057 | Hussain, A., et al. (2022). "Preferential Solvation Study of the Synthesized Aldose Reductase Inhibitor (SE415) in the {PEG 400 (1)+ Water (2)} Cosolvent Mixture and GastroPlus-Based Prediction." 7(1): 1197-1210. | Title |
| 1058 | Hussain, A., et al. (2023). "GastroPlus-and HSPiP-oriented predictive parameters as the basis of valproic acid-loaded mucoadhesive cationic nanoemulsion gel for improved nose-to-brain delivery to control convulsion in humans." 9(8): 603. | Title |
| 1059 | Hutana, G. and K. Stewart (2016). "Intrathecal baclofen therapy for management of spasticity: 5-year data from the Australian paediatric multicentre audit." Developmental medicine and child neurology 58: 62. | Title |
| 1060 | Hutchinson, D. R. (1989). "Modified release tizanidine: a review." J Int Med Res 17(6): 565-573. | Title |
| 1061 | Hutchinson, M., et al. (2015). "Efficacy of delayed-release dimethyl fumarate in multiple sclerosis patients with moderate disability: an integrated analysis of the phase 3 define and confirm studies." Neurology 84. | Title |
| 1062 | Hutchinson, M., et al. (2013). "Efficacy of BG-12 (dimethyl fumarate) in relapsing remitting multiple sclerosis in patients from Europe: an integrated analysis of the phase 3 DEFINE and CONFIRM studies." Multiple sclerosis (Houndmills, Basingstoke, England) 19(11): 466. | Title |

| | | |
|---|---|---|
| 1063 | Hutchinson, M., et al. (2012). "Effect of BG-12 in subgroups of patients with relapsing-remitting multiple sclerosis: findings from the CONFIRM (comparator and an oral fumarate in relapsing-remitting multiple sclerosis) study." Eur J Neurol 19: 355. | Title |
| 1064 | Hutchinson, M., et al. (2013). "Clinical efficacy of BG-12 (dimethyl fumarate) for relapsing remitting multiple sclerosis according to prior therapy: an integrated analysis of the phase 3 DEFINE and CONFIRM studies." Multiple sclerosis (Houndmills, Basingstoke, England) 19(11): 235. | Title |
| 1065 | Hutchinson, M., et al. (2014). "Clinical efficacy of delayed-release dimethyl fumarate in minority patients with relapsing-remitting multiple sclerosis (RRMS): an integrated analysis of the phase 3 define and confirm studies." Neurology 82(10). | Title |
| 1066 | Hutchinson, M., et al. (2014). "Efficacy of delayed-release dimethyl fumarate in multiple sclerosis patients with moderate disability: an integrated analysis of the phase 3 studies." Multiple sclerosis (Houndmills, Basingstoke, England) 20(1): 116. | Title |
| 1067 | Hutson, P., et al. (2022). "Safety, Pharmacokinetic, and Pharmacodynamic Study of a Sublingual Formula for the Treatment of Vasovagal Syncope." Drugs R D 22(1): 61-70. | Title |
| 1068 | Hyrich, K. L., et al. (2006). "Predictors of response to anti-TNF-alpha therapy among patients with rheumatoid arthritis: results from the British Society for Rheumatology Biologics Register." Rheumatology (Oxford) 45(12): 1558-1565. | Title |
| 1069 | I.K, K., et al. (2023). "Perspectives on systematic generation of antibiotic resistance with special emphasis on modern antibiotics." Total Environment Research Themes 8: 100068. | Title |
| 1070 | Iannone, L. F., et al. (2024). "Association of anti-calcitonin gene-related peptide with other monoclonal antibodies for different diseases: A multicenter, prospective, cohort study." Eur J Neurol 31(12): e16450. | Title |
| 1071 | Ibrahim, I. A. A., et al. (2023). "Chitosan biopolymer functionalized with graphene oxide and titanium dioxide with Escin metallic nanocomposites for anticancer potential against colon cancer." International Journal of Biological Macromolecules 253: 127334. | Title |
| 1072 | Ibrahim, K. A., et al. (2017). "Formulation, Evaluation and release rate characteristics of medicated jelly of vitamin C." Pak J Pharm Sci 30(2(Suppl.)): 579-583. | Title |
| 1073 | Ibrahim, N. (2019). "Global Journal of Medical Therapeutics." | Title |
| 1074 | Ibrahim, N. A., et al. (2024). "Medication self-management among older adults with cognitive frailty." Research in Social and Administrative Pharmacy 20(2): 172-181. | Title |
| 1075 | Ibrahim, N. J. G. J. M. T. (2019). "Cabozantinib: cabometyx versus cometriq. Are they interchangeable?" 1: 5-7. | Title |

| | | |
|---|---|---|
| 1076 | Ibrahim, W. and E. Nour Eldaim (2015). "Effect of Quinine Therapy on Renal Function Parameters in Pregnant Women Infected with Plasmodium." | Title |
| 1077 | Imam, F., et al. (2024). "Formulation and characterization of polymeric nanoparticle of Rivastigmine for effective management of Alzheimer's disease." Saudi Pharmaceutical Journal 32(5): 102048. | Title |
| 1078 | Imam, F., et al. (2020). "Determination of isoniazid acetylation patterns in tuberculosis patients receiving DOT therapy under the Revised National tuberculosis Control Program (RNTCP) in India." Saudi Pharmaceutical Journal 28(6): 641-647. | Title |
| 1079 | Imhof, A. and R. Walter (2002). Miscellaneous antibacterial drugs. Side Effects of Drugs Annual. J. K. Aronson, Elsevier. 25: 287-330. | Title |
| 1080 | in 't Veld, A. E., et al. (2023). "Immunosuppression by hydroxychloroquine: Mechanistic proof in in vitro experiments but limited systemic activity in a randomized placebo-controlled clinical pharmacology study." 71(4): 617-627. | Title |
| 1081 | Ingelman-Sundberg, M. (2001). "Genetic and environmental causes for interindividual variability in drug pharmacokinetics." International Congress Series 1220: 175-186. | Title |
| 1082 | Iqbal, A., et al. (2024). "In silico identification and virtual screening to discover potent therapeutic phytochemicals against CMT2A." Journal of the Indian Chemical Society 101(11): 101403. | Title |
| 1083 | Iqbal, M., et al. (2018). "High throughput μ-SPE based elution coupled with UPLC–MS/MS for determination of eluxadoline in plasma sample: Application in pharmacokinetic characterization of PLGA nanoparticle formulations in rats." J Pharm Biomed Anal 149: 172-178. | Title |
| 1084 | Iqbal, M., et al. (2020). "Development and validation of a novel UPLC-MS/MS method for quantification of delafloxacin in plasma and aqueous humour for pharmacokinetic analyses." Journal of Chromatography B 1138: 121961. | Title |
| 1085 | Irham, L. M., et al. (2020). "Integration of genetic variants and gene network for drug repurposing in colorectal cancer." Pharmacol Res 161: 105203. | Title |
| 1086 | Irshad, M. J. A., Pain and I. Care (2019). "Intravenous paracetamol in pediatrics: A global perspective." | Title |
| 1087 | Islam, K., et al. (2022). "Pharmacodynamic evaluation of piperacillin/tazobactam versus meropenem against extended-spectrum β-lactamase-producing and non-producing Escherichia coli clinical isolates in a hollow-fibre infection model." 77(9): 2448-2455. | Title |
| 1088 | Islam, M. R., et al. (2024). "Identification of new inhibitors for the avian H1N1 virus through molecular docking and dynamic simulation approaches." Journal of the Indian Chemical Society 101(10): 101274. | Title |

| | | |
|---|---|---|
| 1089 | Islam, M. T., et al. (2024). "GabaAergic sedative prospection of sclareol-linalool co-treatment: An antagonistic intervention through in vivo and in silico studies." Neuroscience Letters: 138060. | Title |
| 1090 | Islam, S. I. (2015). Glimpse of My Scientific Path: The Quest Continues. Jobs, Collaborations, and Women Leaders in the Global Chemistry Enterprise, ACS Publications: 353-372. | Title |
| 1091 | Islam, S. I., et al. (2001). "An evaluation of theophylline dosing." 22(12): 1092-1095. | Title |
| 1092 | Ismail, S., et al. (2021). "Pan-vaccinomics approach towards a universal vaccine candidate against WHO priority pathogens to address growing global antibiotic resistance." Computers in Biology and Medicine 136: 104705. | Title |
| 1093 | Israili, Z. H. (2011). "Advances in the treatment of type 2 diabetes mellitus." Am J Ther 18(2): 117-152. | Title |
| 1094 | Iwasaki, K., et al. (2004). "Evaluation of Fast Disintegrating Lansoprazole Tablet in Human Subjects." Drug Metabolism and Pharmacokinetics 19(3): 227-235. | Title |
| 1095 | Iwata, O. and S. Iwata (2011). "Filling the evidence gap: how can we improve the outcome of neonatal encephalopathy in the next 10 years?" Brain Dev 33(3): 221-228. | Title |
| 1096 | Jackson, J. C., et al. (2010). "The returning to everyday tasks utilizing rehabilitation networks (RETURN) trial: a pilot, feasibility trial including in-home cognitive rehabilitation of ICU survivors." American journal of respiratory and critical care medicine 181(1). | Title |
| 1097 | Jackson, M., et al. (1994). "The hazards of prescribing from serum levels." Seizure 3(3): 225-233. | Title |
| 1098 | Jacky, K. Y., et al. (2019). "Using pharmacokinetics for tailoring prophylaxis in people with hemophilia switching between clotting factor products: a scoping review." 3(3): 528-541. | Title |
| 1099 | Jacob, S. and A. B. Nair (2016). "An Updated Overview on Therapeutic Drug Monitoring of Recent Antiepileptic Drugs." Drugs R D 16(4): 303-316. | Title |
| 1100 | Jacob, S., et al. (2017). "A review on therapeutic drug monitoring of the mTOR class of immunosuppressants: everolimus and sirolimus." 33: 290-301. | Title |
| 1101 | Jafar, M., et al. (2023). "Development of apigenin loaded gastroretentive microsponge for the targeting of Helicobacter pylori." Saudi Pharmaceutical Journal 31(5): 659-668. | Title |
| 1102 | Jafar, T. H., et al. (2017). "Large cardiovascular risk burden in rural Bangladesh, Pakistan, and Sri Lanka: design and preliminary findings in a Cluster Randomized Controlled Trial (COBRA-BPS)." European heart journal 38: 1335. | Title |

| | | |
|---|---|---|
| 1103 | Jain, K. K. (2000). "Evaluation of mitoxantrone for the treatment of multiple sclerosis." Expert Opin Investig Drugs 9(5): 1139-1149. | Title |
| 1104 | Jairath, V., et al. (2017). "Evolving concepts in phases I and II drug development for Crohn's disease." 11(2): 246-255. | Title |
| 1105 | Jakimovski, D., et al. (2022). "Multiple Sclerosis in Children: Differential Diagnosis, Prognosis, and Disease-Modifying Treatment." CNS Drugs 36(1): 45-59. | Title |
| 1106 | Jan, A. M. and F. M. J. T. J. o. C. D. P. Jadu (2020). "A Case Report of maxillary aspergillosis with unusual clinical and imaging presentations." 21: 211-214. | Title |
| 1107 | Jan, M. M. S., et al. (2009). "Pregabalin: Preliminary Experience in Intractable Childhood Epilepsy." Pediatric Neurology 40(5): 347-350. | Title |
| 1108 | Jang, S. B., et al. (2010). "Pharmacokinetic comparison of controlled-release and immediate-release oral formulations of simvastatin in healthy Korean subjects: a randomized, open-label, parallel-group, single- and multiple-dose study." Clin Ther 32(1): 206-216. | Title |
| 1109 | Jankovic, J. and M. Stacy (2007). "Medical management of levodopa-associated motor complications in patients with Parkinson's disease." CNS Drugs 21(8): 677-692. | Title |
| 1110 | Jastaniah, W., et al. (2018). "High-dose methotrexate vs. Capizzi methotrexate for the treatment of childhood T-cell acute lymphoblastic leukemia." Leukemia Research Reports 10: 44-51. | Title |
| 1111 | Jean, S.-S., et al. (2018). "Carbapenem-resistant Enterobacteriaceae infections: Taiwan aspects." 9: 2888. | Title |
| 1112 | Jean, S.-S., et al. (2015). "Carbapenemase-producing Gram-negative bacteria: current epidemics, antimicrobial susceptibility and treatment options." 10(3): 407-425. | Title |
| 1113 | Jenghua, K., et al. (2025). "Prevalence, determinants, and health outcomes of potentially inappropriate medication use according to the 2023 Beers criteria among hospitalised older patients." Archives of Gerontology and Geriatrics 129: 105693. | Title |
| 1114 | Jeong, Y.-J., et al. (2021). "Prospective observational study of the impact of plasma colistin levels in patients with carbapenem-resistant Acinetobacter baumannii pneumonia." 27: 315-323. | Title |
| 1115 | Jha, V. and K. S. Chugh (2003). "Nephropathy Associated With Animal, Plant, and Chemical Toxins in the Tropics." Seminars in Nephrology 23(1): 49-65. | Title |
| 1116 | Jha, V., et al. (2000). "Infections in dialysis and transplant patients in tropical countries." Kidney International 57: S85-S93. | Title |
| 1117 | Jiang, H., et al. (2023). "Carbon nanomaterials: A growing tool for the diagnosis and treatment of diabetes mellitus." Environmental Research 221: 115250. | Title |

| | | |
|---|---|---|
| 1118 | Jimenez, M. E., et al. (2017). "A Pilot Randomized Trial of a Video Patient Decision Aid to Facilitate Early Intervention Referrals From Primary Care." Clinical pediatrics 56(3): 268-277. | Title |
| 1119 | Jin, L., et al. (2021). "A synthetic peptide AWRK6 ameliorates metabolic associated fatty liver disease: involvement of lipid and glucose homeostasis." Peptides 143: 170597. | Title |
| 1120 | Jin, Y., et al. (2023). "The effect of anti-tuberculosis drug pharmacokinetics on QTc prolongation." International Journal of Antimicrobial Agents 62(4): 106939. | Title |
| 1121 | Jin, Z.-b., et al. (2022). "Population pharmacokinetics and dosing regimen of lithium in Chinese patients with bipolar disorder." 13: 913935. | Title |
| 1122 | Jing, C. J. and S. Syafiie (2021). "Multi-model generalised predictive control for intravenous anaesthesia under inter-individual variability." J Clin Monit Comput 35(5): 1037-1045. | Title |
| 1123 | Joag, K., et al. (2020). "Atmiyata, a community-led intervention to address common mental disorders: study protocol for a stepped wedge cluster randomized controlled trial in rural Gujarat, India." Trials 21(1): 212. | Title |
| 1124 | Johnson, R. C., et al. (2022). "Comparison of 8 weeks standard treatment (rifampicin plus clarithromycin) vs. 4 weeks standard plus amoxicillin/clavulanate treatment [RC8 vs. RCA4] to shorten Buruli ulcer disease therapy (the BLMs4BU trial): study protocol for a randomized controlled multi-centre trial in Benin." Trials 23(1): 559. | Title |
| 1125 | Jonas, M. M. (2000). 20 Hepatitis C in children. Biomedical Research Reports. T. J. Liang and J. H. Hoofnagle, Academic Press. 2: 389-403. | Title |
| 1126 | Jones, R. S. and D. Sims (2013). "An audit of 10 mechanical clot retrievals for acute ischaemic stroke to evaluate the potential benefits in preparation for randomised controlled trials." International journal of stroke 8: 28. | Title |
| 1127 | JORDAN, E., et al. "Optimization in the Context of COVID-19 Prediction and Control: A Literature." | Title |
| 1128 | Jordan, E., et al. (2021). "Optimization in the context of COVID-19 prediction and control: A literature review." 9: 130072-130093. | Title |
| 1129 | Joshi, T., et al. (2024). "ML-based technologies in sustainable agro-food production and beyond: Tapping the (semi) arid landscape for bioactives-based product development." Journal of Agriculture and Food Research 18: 101350. | Title |
| 1130 | Jovin, T. G., et al. (2015). "Thrombectomy within 8 hours after symptom onset in ischemic stroke." New England journal of medicine 372(24): 2296-2306. | Title |
| 1131 | Kaakkola, S., et al. (1994). "Effect of entacapone, a COMT inhibitor, on clinical disability and levodopa metabolism in parkinsonian patients." Neurology 44(1): 77-80. | Title |

| | | |
|---|---|---|
| 1132 | KACHAYANGYUEN, S. and W. Saelim (2023). Clinical outcomes of pharmacokinetic and pharmacodynamic meropenem dosing in critically ill patients with sepsis or septic shock, Silpakorn University. | Title |
| 1133 | Kadri, O. and K. Manai (2016). "NEURAL NETWORK MODELLING OF CARDIAC DOSE CONVERSION COEFFICIENT FOR ARBITRARY X-RAY SPECTRA." Radiat Prot Dosimetry 171(4): 438-444. | Title |
| 1134 | Kadri, O., et al. (2016). "MONTE CARLO STUDY OF THE CARDIAC ABSORBED DOSE DURING X-RAY EXAMINATION OF AN ADULT PATIENT." Radiat Prot Dosimetry 171(4): 431-437. | Title |
| 1135 | Kafri, R., et al. (2014). "A randomized trial comparing rehabilitation and drug therapy for urgency urinary incontinence: 1 year follow up." Neurourology and urodynamics 33(6): 869-870. | Title |
| 1136 | Kalagi, N. A., et al. (2019). "Modulation of Circulating Trimethylamine N-Oxide Concentrations by Dietary Supplements and Pharmacological Agents: A Systematic Review." Advances in Nutrition 10(5): 876-887. | Title |
| 1137 | Kalam, M. A., et al. (2024). "Quercetin-loaded transliposomal gel for effective management of skin cancer: In vitro and cell line efficacy studies." Journal of Drug Delivery Science and Technology 96: 105659. | Title |
| 1138 | Kalam, M. N., et al. (2021). "Development and Evaluation of a Physiologically Based Pharmacokinetic Drug-Disease Model of Propranolol for Suggesting Model Informed Dosing in Liver Cirrhosis Patients." Drug Des Devel Ther 15: 1195-1211. | Title |
| 1139 | Kalow, W. (1986). "Genetics of drug transformation." Clinical Biochemistry 19(2): 76-82. | Title |
| 1140 | Kalsoom, S., et al. (2024). "A Comprehensive Physiologically Based Pharmacokinetic Model of Nadolol in Adults with Renal Disease and Pediatrics with Supraventricular Tachycardia." Pharmaceuticals (Basel) 17(2). | Title |
| 1141 | Kälviäinen, R. (1998). "Tiagabine: a new therapeutic option for people with intellectual disability and partial epilepsy." J Intellect Disabil Res 42 Suppl 1: 63-67. | Title |
| 1142 | Kamal, L., et al. (2022). "The pill of recovery; Molnupiravir for treatment of COVID-19 patients; a systematic review." Saudi Pharmaceutical Journal 30(5): 508-518. | Title |
| 1143 | Kamal, M. A., et al. (2006). "Kinetic analysis of the inhibition of human butyrylcholinesterase with cymserine." Biochimica et Biophysica Acta (BBA) - General Subjects 1760(2): 200-206. | Title |
| 1144 | Kamali, F. and F. Panahi (2010). "Comparison between massage and modality in women with non specific low back pain (20-55 y/o) referred to phyiotherpy clinic of shiraz university of medical sciences." European spine journal 19(11): 2042. | Title |

| | | |
|---|---|---|
| 1145 | Kammerer, W. S. and P. M. Schantz (1993). "ECHINOCOCCAL DISEASE." Infectious Disease Clinics of North America 7(3): 605-618. | Title |
| 1146 | Kamp, J., et al. (2020). "Ketamine pharmacokinetics: a systematic review of the literature, meta-analysis, and population analysis."  133(6): 1192-1213. | Title |
| 1147 | Kamran, M., et al. (2016). "Design, formulation and optimization of novel soft nano-carriers for transdermal olmesartan medoxomil delivery: In vitro characterization and in vivo pharmacokinetic assessment." Int J Pharm 505(1): 147-158. | Title |
| 1148 | Kamstrup, M., et al. (2017). "Psoriasis Gene to Clinic, 8th International Congress. The Queen Elizabeth II Conference Centre, London, UK, 30th November–2nd December 2017 Free communications." | Title |
| 1149 | Kamyab, H., et al. (2023). "Exploring the potential of metal and metal oxide nanomaterials for sustainable water and wastewater treatment: A review of their antimicrobial properties." Chemosphere 335: 139103. | Title |
| 1150 | Kandzari, D. E. (2021). "Catheter-Based Renal Denervation Therapy: evolution of Evidence and Future Directions." Circulation. Cardiovascular interventions 14(12): e011130. | Title |
| 1151 | Kanj, S. S., et al. (2022). "Survival outcome of empirical antifungal therapy and the value of early initiation: A review of the last decade." 8(11): 1146. | Title |
| 1152 | Kanjanasilp, J., et al. (2021). "A meta-analysis of effects of CYP2C9 and CYP2C19 polymorphisms on phenytoin pharmacokinetic parameters."  22(10): 629-640. | Title |
| 1153 | Kanter, J., et al. (2014). "A double-blind placebo controlled study of the effect of beta-alanine, a nonessential amino-acid, on neurologic, motor function, quality of life, and fatigue in patients diagnosed with multiple sclerosis." Neurology 82(10). | Title |
| 1154 | Kaplan, P. W. (2004). "Reproductive health effects and teratogenicity of antiepileptic drugs." Neurology 63(10 Suppl 4): S13-23. | Title |
| 1155 | kapoor, D. u., et al. (2023). "Polymeric nanoparticles approach and identification and characterization of novel biomarkers for colon cancer." Results in Chemistry 6: 101167. | Title |
| 1156 | Kappos, L., et al. (2015). "Siponimod (BAF312) for the treatment of secondary progressive multiple sclerosis (SPMS): baseline characteristics of the EXPAND study population." Multiple sclerosis (Houndmills, Basingstoke, England) 23(11): 317-318. | Title |
| 1157 | Kappos, L., et al. (2013). "Pooled efficacy data from two phase 3 placebo-controlled trials of oral, once-daily teriflunomide." Multiple sclerosis (Houndmills, Basingstoke, England) 19(11): 266-267. | Title |

| | | |
|---|---|---|
| 1158 | Kappos, L., et al. (2015). "Teriflunomide efficacy on annualized relapse rate and expanded disability status scale scores: 2.5-year follow-up in the TOWER extension study in patients with relapsing MS." Multiple sclerosis (Houndmills, Basingstoke, England) 23(11): 567-568. | Title |
| 1159 | Kappos, L., et al. (2012). "Five-year safety and efficacy data of natalizumab from the STRATA study." Multiple sclerosis (Houndmills, Basingstoke, England) 18(4): 221-222. | Title |
| 1160 | Kappos, L., et al. (2014). "Inclusion of brain volume loss in a revised measure of multiple sclerosis disease-activity freedom: the effect of fingolimod." Multiple sclerosis (Houndmills, Basingstoke, England) 20(1): 40. | Title |
| 1161 | Karakonstantis, S., et al. (2021). "Systematic review of antimicrobial combination options for pandrug-resistant Acinetobacter baumannii." 10(11): 1344. | Title |
| 1162 | Karaoui, M., et al. (2018). "Chloral Hydrate Administered by a Dedicated Sedation Service Can Be Used Safely and Effectively for Pediatric Ophthalmic Examination." American Journal of Ophthalmology 192: 39-46. | Title |
| 1163 | Karthick Raja Namasivayam, S., et al. (2023). "Biocompatible nanoscale silica particles fabricated from aminopropyltriethoxysilane functionalized brick ash induced versatile pesticidal activity." Environmental Research 238: 117090. | Title |
| 1164 | Kashour, T. and I. M. Tleyjeh (2020). "It is time to drop hydroxychloroquine from our COVID-19 armamentarium." Medical Hypotheses 144: 110198. | Title |
| 1165 | Kaski, M., et al. (1991). "Treatment of epilepsy in mentally retarded patients with a slow-release carbamazepine preparation." J Ment Defic Res 35 ( Pt 3): 231-239. | Title |
| 1166 | Kassem, M. A., et al. (2017). "Maximizing the Therapeutic Efficacy of Imatinib Mesylate–Loaded Niosomes on Human Colon Adenocarcinoma Using Box-Behnken Design." Journal of Pharmaceutical Sciences 106(1): 111-122. | Title |
| 1167 | Katoue, M. G., et al. (2016). "Parenteral nutrition in hospital pharmacies: Exploring the practices and identifying opportunities for quality improvement." 29(6): 664-674. | Title |
| 1168 | Kavanaugh, A., et al. (2013). "Improvements in productivity at paid work and within household, and increased participation in daily activities after 24 weeks of certolizumab pegol in patients with psoriatic arthritis: results of rapid-PSA study." Ann Rheum Dis 72. | Title |
| 1169 | Kavanaugh, A., et al. (2015). "Clinical responses in joint and skin outcomes and patient-reported outcomes are associated with increased productivity in the workplace and at home in psoriatic arthritis patients treated with certolizumab pegol." Ann Rheum Dis 74: 864-865. | Title |

| | | |
|---|---|---|
| 1170 | Kavanaugh, A., et al. (2013). "Ustekinumab improves arthritis-related and skin-related quality of life in patients with active psoriatic arthritis: patient reported outcomes from randomized and double blinded phase III psummit I trial." Value in health 16(3): A227-A228. | Title |
| 1171 | Kay, J., et al. (2015). "BOW015, a biosimilar infliximab: disease activity and disability outcomes from a phase 3 active comparator study in patients with active rheumatoid arthritis on stable methotrexate doses." Ann Rheum Dis 74: 462-463. | Title |
| 1172 | Kazi, M., et al. (2024). "Nutraceutically-enhanced oral delivery of vitamin D3 via Bio-SNEDDS: Demonstrating in vivo superiority over pediatric formulations." Biochemical and Biophysical Research Communications 709: 149852. | Title |
| 1173 | Kelesidis, T. and M. E. J. E. o. o. d. s. Falagas (2015). "The safety of polymyxin antibiotics." 14(11): 1687-1701. | Title |
| 1174 | Kempker, R. R., et al. (2019). "A pharmacology perspective on simultaneous tuberculosis and hepatitis C treatment." 63(12): 10.1128/aac. 01215-01219. | Title |
| 1175 | Kepler, C., et al. (2011). "Do epidural steroid injections affect the outcome of patients treated for lumbar stenosis? A subgroup analysis of the SPORT." Spine journal 11(10): 24S. | Title |
| 1176 | Keystone, E., et al. (2011). "Initial combination therapy with adalimumab plus methotrexate leads to better long-term outcomes in patients with advanced rheumatoid arthritis: analysis of the final 10-year results of an open-label extension of a phase 1-7 trial." Arthritis Rheum 63(10). | Title |
| 1177 | Khafagy, E.-S., et al. (2020). "Defining design space for optimization of escitalopram ultra-fast melting tablet using suspension spray-coating technique: In-vitro and in-vivo evaluation." Journal of Drug Delivery Science and Technology 57: 101631. | Title |
| 1178 | Khalid, S., et al. (2023). "Application of a physiologically based pharmacokinetic model in predicting captopril disposition in children with chronic kidney disease." Sci Rep 13(1): 2697. | Title |
| 1179 | Khalil, M. A., et al. (2013). "The role of intravenous paracetamol in conscious sedation during Internal Cardioverter Defibrillator (ICD) insertion in geriatric patients." Egyptian Journal of Anaesthesia 29(1): 41-45. | Title |
| 1180 | Khalil, R., et al. (2024). "Intranasal transfersomal based in situ gel for augmenting methylphenidate bioavailability and brain delivery: In vitro and in vivo evaluation." Journal of Drug Delivery Science and Technology 101: 106125. | Title |
| 1181 | Khan, F., et al. (2011). "Effectiveness of theta burst stimulation (TBS), functional electrical stimulation (FES) and physiotherapy in post-stroke motor rehabilitation: randomised control trial." Physiotherapy (united kingdom) 97: eS607. | Title |

| | | |
|---|---|---|
| 1182 | Khan, H., et al. (2023). "Nonlinear dynamics of a piecewise modified ABC fractional-order leukemia model with symmetric numerical simulations." 15(7): 1338. | Title |
| 1183 | Khan, H., et al. (2018). "Evidence and prospective of plant derived flavonoids as antiplatelet agents: Strong candidates to be drugs of future." Food and Chemical Toxicology 119: 355-367. | Title |
| 1184 | Khan, H., et al. (2024). "Clinical pharmacokinetics of glipizide: a systematic review." Expert Opin Drug Metab Toxicol: 1-11. | Title |
| 1185 | Khan, L. M., et al. (2013). "Adverse drug reactions in hospitalized pediatric patients of Saudi Arabian University Hospital and impact of pharmacovigilance in reporting ADR." Saudi Pharmaceutical Journal 21(3): 261-266. | Title |
| 1186 | Khan, M. A., et al. (2015). "Maternal colonization of group B streptococcus: prevalence, associated factors and antimicrobial resistance." 35(6): 423-427. | Title |
| 1187 | Khan, S., et al. (2024). "Targeting Refractory Triple-negative Breast Cancer Metastasis with Sacituzumab Govitecan: A new era in Precision Medicine." | Title |
| 1188 | Khan, S., et al. (2024). "Exploring effective diagnosis of Alzheimer disease: Experimental and computational analysis of hybrid benzimidazole based thiazolidinone derivatives." Results in Chemistry 9: 101663. | Title |
| 1189 | Khan, S. and A. J. D. M. R. Shahzadi (2019). "Clinical pharmacokinetics of drugs in cardiopulmonary associated cachexia without hepatorenal pathology: a systematic review." 51(1): 1-11. | Title |
| 1190 | Khandia, R., et al. (2024). "Relative synonymous codon usage and codon pair analysis of depression associated genes." 14(1): 3502. | Title |
| 1191 | Kherroubi, L., et al. (2024). "Navigating fluoroquinolone resistance in Gram-negative bacteria: a comprehensive evaluation." 6(4): dlae127. | Title |
| 1192 | Khoo, S. H. and T. Walley (1998). Miscellaneous antibacterial drugs. Side Effects of Drugs Annual. J. K. Aronson, Elsevier. 21: 265-281. | Title |
| 1193 | Kieburtz, K. D. (2012). "Design innovations and baseline findings in a long-term Parkinson's trial: NET-PD LS-1 The NINDS NET-PD Investigators." Movement disorders 27: S124. | Title |
| 1194 | Kieseier, B., et al. (2013). "Effect of peginterferon β-1a on disability progression in patients with relapsing remitting multiple sclerosis: year 1 data from the pivotal phase 3 ADVANCE study." Multiple sclerosis (Houndmills, Basingstoke, England) 19(11): 223. | Title |
| 1195 | Kieseier, B., et al. (2015). "The efficacy of peginterferon Beta-1a in early and later-stage patients with multiple sclerosis: a 1-year subgroup analysis based on disease duration from the advance study." Neurology 84. | Title |

| | | |
|---|---|---|
| 1196 | Kieseier, B., et al. (2014). "Peginterferon beta-1a may improve recovery following relapses: data from the pivotal phase 3 advance study in patients with relapsing remitting multiple sclerosis." Neurology 82(10). | Title |
| 1197 | Kim, D.-H., et al. (2023). "Human exposure to persistent and mobile chemicals: A review of sources, internal levels and health implications." Science of The Total Environment 893: 164764. | Title |
| 1198 | Kim, K., et al. (2020). "Prediction of Response to Tumor Necrosis Value-$\alpha$ Blocker Is Suggested by (18)F-NaF SUV(max) But Not by Quantitative Pharmacokinetic Analysis in Patients With Ankylosing Spondylitis." AJR Am J Roentgenol 214(6): 1352-1358. | Title |
| 1199 | Kim, S. B., et al. (2020). "Interim guidelines on antiviral therapy for COVID-19."  52(2): 281. | Title |
| 1200 | Kingsbury, D., et al. (2013). "PReS-FINAL-2161: safety and effectiveness of adalimumab in children with polyarticular juvenile idiopathic arthritis aged 2 to <4 years or >=4 years weighing <15 kg." Pediatric rheumatology 11. | Title |
| 1201 | Kiosses, D. N., et al. (2014). "Moving psychosocial interventions to populations with challenging problems." American journal of geriatric psychiatry 22(3): S24-S25. | Title |
| 1202 | Kira, J. I., et al. (2020). "Efficacy and safety of ofatumumab versus placebo in relapsing multiple sclerosis patients in japan and russia: results from the phase 2 apolitos study." Multiple sclerosis journal 26(3 SUPPL): 219. | Title |
| 1203 | Kirwan, M., et al. (2021). "Exploring population pharmacokinetic models in patients treated with vancomycin during continuous venovenous haemodiafiltration (CVVHDF)." Crit Care 25(1): 443. | Abstract |
| 1204 | Kishimoto, M., et al. (2023). "Upadacitinib for moderate-to-severe atopic dermatitis, in adults and adolescents 12 years and older: review of international and Japanese populations."  19(1): 19-35. | Title |
| 1205 | Kisu, I., et al. (2013). "Current status of uterus transplantation in primates and issues for clinical application." Fertility and Sterility 100(1): 280-294. | Title |
| 1206 | Kita, M., et al. (2013). "Clinical and neuroradiologic efficacy of BG-12 (dimethyl fumarate) in us patients with relapsing-remitting multiple sclerosis (RRMS): an integrated analysis of the phase 3 define and confirm studies." Neurology 80(1). | Title |
| 1207 | Kita, M., et al. (2012). "Effects of BG-12 on quality of life in patients with relapsing-remitting multiple sclerosis: findings from the CONFIRM study." Multiple sclerosis (Houndmills, Basingstoke, England) 18(4): 254. | Title |

| | | |
|---|---|---|
| 1208 | Klassen, P. D., et al. (2018). "Post-lumbar discectomy reoperations that are associated with poor clinical and socioeconomic outcomes can be reduced through use of a novel annular closure device: results from a 2-year randomized controlled trial." ClinicoEconomics and outcomes research 10: 349-357. | Title |
| 1209 | Kleine-Brueggeney, M., et al. (2010). "Pharmacogenetics in palliative care." Forensic Science International 203(1): 63-70. | Title |
| 1210 | Knibbe, C. A., et al. (2015). "Drug disposition in obesity: toward evidence-based dosing." 55(1): 149-167. | Title |
| 1211 | Koam, A. N. A., et al. (2024). "Connection number-based molecular descriptors of skin cancer drugs." Ain Shams Engineering Journal 15(6): 102750. | Title |
| 1212 | Koch, M., et al. (2015). "Comparative utility of different progression metrics in PPMS: re-analysis of the promise clinical trial dataset." Neurology 84. | Title |
| 1213 | Koduah, P., et al. (2018). "Vitamin D supplementation in multiple sclerosis: primary efficacy endpoint and safety of a randomized, controlled, double-blind phase II trial (EVIDIMS)." Multiple sclerosis journal 24(2): 1016-1017. | Title |
| 1214 | Kollef, M., et al. (2023). "Prospective role of cefiderocol in the management of carbapenem-resistant Acinetobacter baumannii infections: review of the evidence." 62(2): 106882. | Title |
| 1215 | Komatsu, T., et al. (2016). "Population pharmacokinetic analysis and dosing regimen optimization of penicillin G in patients with infective endocarditis." 2: 1-8. | Title |
| 1216 | Kondo, T. and T. Ohshima (1995). "Retrospective investigation of medico-legal autopsy cases involving mentally handicapped individuals." Nihon Hoigaku Zasshi 49(6): 478-483. | Title |
| 1217 | Kontoghiorghes, G. J. J. I. J. o. M. S. (2023). "Drug Selection and Posology, Optimal Therapies and Risk/Benefit Assessment in Medicine: The Paradigm of Iron-Chelating Drugs." 24(23): 16749. | Title |
| 1218 | Kotb, E. S., et al. (2024). "Examining the quaternary ammonium chitosan Schiff base-ZnO nanocomposite's potential as protective therapy for rats' cisplatin-induced hepatotoxicity." International Journal of Biological Macromolecules 276: 133616. | Title |
| 1219 | Krawczyk, S., et al. (2022). "Congenital hyperinsulinaemic hypoglycaemia—a review and case presentation." 11(20): 6020. | Title |
| 1220 | Kreins, A. Y., et al. (2024). "Favipiravir induces HuNoV viral mutagenesis and infectivity loss with clinical improvement in immunocompromised patients." Clinical Immunology 259: 109901. | Title |

| | | |
|---|---|---|
| 1221 | Kremenchutzky, M., et al. (2015). "Efficacy of delayed-release dimethyl fumarate vs glatiramer acetate on a novel composite outcome measure of inflammatory disease activity: post-hoc analysis of the CONFIRM study." Multiple sclerosis (Houndmills, Basingstoke, England) 23(11): 545-546. | Title |
| 1222 | Krieger, S., et al. (2014). "Alemtuzumab reduces disease activity in treatmentnaive patients with highly active relapsing-remitting multiple sclerosis." Multiple sclerosis (Houndmills, Basingstoke, England) 20(1): 106-107. | Title |
| 1223 | Krūmiņa, A. (2023). "Colistin Use Pattern and Nephrotoxicity in Critically Ill Patients with Carbapenem-Resistant Gram-Negative Bacterial Infections. Summary of the Doctoral Thesis." | Title |
| 1224 | Kubba, R. and Y. Al-Gindan (1989). "Leishmaniasis." Dermatologic Clinics 7(2): 331-352. | Title |
| 1225 | Kucuk, M., et al. (2023). "Dexmedetomidine may reduce the risk of acute kidney injury development in critically ill patients during colistin therapy." 29(7): 673-677. | Title |
| 1226 | Kulkarni, R. V., et al. (2014). "Efficacy and safety of delayed-release Dimethyl Fumarate in patients with relapsingremitting multiple sclerosis from India: an integrated analysis of the phase 3 define and confirm studies." Annals of Indian Academy of Neurology 17: S208-S209. | Title |
| 1227 | KUMAR JAIN, N., et al. (2024). "A comprehensive overview of selective and novel fibroblast growth factor receptor inhibitors as a potential anticancer modality." 74(1): 1-36. | Title |
| 1228 | Kumar, K., et al. (2009). "Relationship between quality of life, disability and pain in patients with failed back surgery syndrome." Pain medicine (Malden, Mass.) 10(1): 278. | Title |
| 1229 | Kumar, P., et al. (2022). "Limitations of current chemotherapy and future of nanoformulation-based AmB delivery for visceral leishmaniasis—An updated review." 10: 1016925. | Title |
| 1230 | Kumar, R. R., et al. (2021). "Piperazine, a Key Substructure for Antidepressants: Its Role in Developments and Structure-Activity Relationships." ChemMedChem 16(12): 1878-1901. | Title |
| 1231 | Kumar, S. and P. Adhikari (2011). "Efficacy of impairment-based manual physical therapy intervention for painful stiff shoulder in type-ii diabetes mellitus subjects-a randomized clinical trial." Physiotherapy (united kingdom) 97: eS642-S646S643. | Title |
| 1232 | Kumar, S., et al. (2022). "An update on advancements in treatment options for managing Klebsiella pneumoniae infections." 8(6): 439-449. | Title |

| | | |
|---|---|---|
| 1233 | Kumaran, M., et al. (2024). "Investigation of the molecular structure of CHBP, biological activities and SARS-CoV-2 protein binding interaction by molecular and biomolecular spectroscopy approaches." Spectrochimica Acta Part A: Molecular and Biomolecular Spectroscopy 322: 124853. | Title |
| 1234 | Kumaraswamy, M., et al. (2023). "Adverse Drug Reaction Tools Used in Causality Assessment." 16(4). | Title |
| 1235 | Kuo, K. H. M., et al. (2022). "Safety and efficacy of mitapivat, an oral pyruvate kinase activator, in adults with non-transfusion dependent α-thalassaemia or β-thalassaemia: an open-label, multicentre, phase 2 study." The Lancet 400(10351): 493-501. | Title |
| 1236 | Kursumovic, A. and S. Rath (2014). "Can anular closure device improve the outocme of discectomies? Clinical and real world experience with anular closure at a single site." European spine journal 23(11): 2557-2558. | Title |
| 1237 | Kursumovic, A. and S. Rath (2018). "Effectiveness of an annular closure device inpatients that meet RCT screening criteria VS. A real-world population: retrospective analysis of a prospective registry." European spine journal 27: S649-S650. | Title |
| 1238 | Kurt Ammer, M. J. H. (2013). "Thermology." 33(3): 165-169. | Title |
| 1239 | Kuusalo, L., et al. (2015). "Depressive symptoms and the risk of work disability in early rheumatoid arthritis." Ann Rheum Dis 74: 1014. | Title |
| 1240 | Kwan, A. and J. M. J. C. P. R. Puck (2015). "Newborn screening for severe combined immunodeficiency." 3: 34-42. | Title |
| 1241 | Kwara, A., et al. (2019). "Effect of Rifampin-Isoniazid-Containing Antituberculosis Therapy on Efavirenz Pharmacokinetics in HIV-Infected Children 3 to 14 Years Old." Antimicrob Agents Chemother 63(1). | Title |
| 1242 | Kwong, Y. L., et al. (2009). "Intrathecal chemotherapy for hematologic malignancies: drugs and toxicities." Ann Hematol 88(3): 193-201. | Title |
| 1243 | Kyprianou, M., et al. (2023). "Macrolides for better resolution of community-acquired pneumonia: A global meta-analysis of clinical outcomes with focus on microbial aetiology." International Journal of Antimicrobial Agents 62(4): 106942. | Title |
| 1244 | Laakso, M. L., et al. (2005). "Urinary 6-hydroxymelatonin sulfate excretion in intellectually disabled subjects with sleep disorders and multiple medications: validation of measurements in urine extracted from diapers." Scand J Clin Lab Invest 65(5): 413-431. | Title |
| 1245 | Laamari, Y., et al. (2025). "Thymol-1,2,3-triazole derivatives: Network pharmacology, molecular simulations and synthesis targeting breast cancer." Journal of Molecular Structure 1321: 140060. | Title |
| 1246 | Lachmann, H., et al. (2019). "Canakinumab improves patient-reported outcomes in patients with recurrent fever syndromes: results from a phase 3 trial." Arthritis & rheumatology 71: 1374-1376. | Title |
| 1247 | Ladenheim, B., et al. (2011). "Robot-aided therapy in pediatrics: 6 months after." Developmental medicine and child neurology 53: 83-84. | Title |

| | | |
|---|---|---|
| 1248 | LaGanke, C., et al. (2015). "Real-world fingolimod first-dose effects in patients with pre-existing hypertension, pre-existing cardiac conditions and in those receiving selective serotonin-reuptake inhibitors." Multiple sclerosis (Houndmills, Basingstoke, England) 23(11): 699-700. | Title |
| 1249 | Laila, O., et al. (2023). "Enhancement of nutraceutical and anti-diabetic potential of fenugreek (Trigonella foenum-graecum). Sprouts with natural elicitors." Saudi Pharmaceutical Journal 31(1): 1-13. | Title |
| 1250 | Lambert, P. R., et al. (2015). "Ph2b efficacy and safety of intratympanic OTO-104 in meniere's disease." Otolaryngology--head and neck surgery 153(1): 108. | Title |
| 1251 | Lamoth, F., et al. (2021). "Role and interpretation of antifungal susceptibility testing for the management of invasive fungal infections." 7(1): 17. | Title |
| 1252 | Lamy, C. and J. L. Mas (2013). "[Anticoagulant treatment in patients with atrial fibrillation, according to risk factors for stroke]." Geriatr Psychol Neuropsychiatr Vieil 11(1 Suppl): 23-33. | Title |
| 1253 | Langaee, T., et al. (2021). "Pharmacogenetic predictors of nevirapine pharmacokinetics in Ghanaian children living with HIV with or without TB coinfection." Infection, Genetics and Evolution 92: 104856. | Title |
| 1254 | Lansinger, B. (2011). "Qigong and exercise therapy in patients with long-term neck pain: a prospective randomized trial." Physiotherapy (united kingdom) 97(var.pagings): eS660. | Title |
| 1255 | Lapadula, G. and G. F. Ferraccioli (2012). "Biosimilars in rheumatology: pharmacological and pharmacoeconomic issues." Clin Exp Rheumatol 30(4 Suppl 73): S102-106. | Title |
| 1256 | Lapchak, P. A. (2012). "A series of novel neuroprotective blood brain barrier penetrating flavonoid drugs to treat acute ischemic stroke." Curr Pharm Des 18(25): 3694-3703. | Title |
| 1257 | Lapetina, D. L., et al. (2020). Pharmacogenomics and psychopharmacology. Seminars in Clinical Psychopharmacology, Cambridge University Press London. | Title |
| 1258 | Lassiter, H. A. (1992). "Intravenous Immunoglobulin in the Prevention and Treatment of Neonatal Bacterial Sepsis." Advances in Pediatrics 39(1): 71-99. | Title |
| 1259 | Lattanzio, F., et al. (2012). "Geriatric conditions and the risk of adverse drug reactions in older adults: a review." Drug Saf 35 Suppl 1: 55-61. | Title |
| 1260 | Lauche, R., et al. (2014). "Efficacy of alexander technique in the treatment of chronic non-specific neck pain: a randomized controlled trial." Journal of alternative and complementary medicine (New York, N.Y.) 20(5): A59. | Title |

| | | |
|---|---|---|
| 1261 | Lavery, M. J. and R. Wolf (2019). "The life-threatening rash of poisoning." Clinics in Dermatology 37(2): 136-147. | Title |
| 1262 | Law, S., et al. (2016). "Sumatriptan plus naproxen for the treatment of acute migraine attacks in adults." Cochrane Database Syst Rev 4(4): Cd008541. | Title |
| 1263 | Lee, C.-R., et al. (2017). "Biology of Acinetobacter baumannii: pathogenesis, antibiotic resistance mechanisms, and prospective treatment options." 7: 55. | Title |
| 1264 | Lee, E., et al. (2019). "A herbal medicine, gongjindan, in subjects with chronic dizziness: a prospective, multicenter, randomized, double-blind, placebo-controlled clinical trial for effectiveness, safety, and cost-effectiveness." European stroke journal 4: 805. | Title |
| 1265 | Lee, H. C., et al. (2011). "Use of antiarrhythmic drugs in elderly patients." J Geriatr Cardiol 8(3): 184-194. | Title |
| 1266 | Lee, J. K., et al. (2015). "Optimizing pharmacotherapy in elderly patients: the role of pharmacists." Integr Pharm Res Pract 4: 101-111. | Title |
| 1267 | Lee, Y., et al. (2019). "Effects of education and support groups organized by IBCLCs in early postpartum on breastfeeding." Midwifery 75: 5-11. | Title |
| 1268 | Lees, A. J. (1987). "A sustained-release formulation of L-dopa (Madopar HBS) in the treatment of nocturnal and early-morning disabilities in Parkinson's disease." Eur Neurol 27 Suppl 1: 126-134. | Title |
| 1269 | Lefranc, F., et al. (2019). "Algae metabolites: from in vitro growth inhibitory effects to promising anticancer activity." Natural Product Reports 36(5): 810-841. | Title |
| 1270 | Leist, T., et al. (2015). "Assessing comparative outcomes from teriflunomide and dimethyl fumarate studies in relapsing MS: use of “number needed to treat” analysis." Neurology 84. | Title |
| 1271 | Lenhard, J. R., et al. (2016). "High-intensity meropenem combinations with polymyxin B: new strategies to overcome carbapenem resistance in Acinetobacter baumannii." 72(1): 153-165. | Title |
| 1272 | Lenhardt, R., et al. (2012). "Strict glycemic control in patients with intracranial hemorrhage, a preliminary report." European journal of anaesthesiology 29: 112. | Title |
| 1273 | Lennox, N. G., et al. (2013). "General practitioners' views on perceived and actual gains, benefits and barriers associated with the implementation of an Australian health assessment for people with intellectual disability." Journal of intellectual disability research 57(10): 913-922. | Title |

| | | |
|---|---|---|
| 1274 | Leon-Djian, C. B., et al. (2011). "Étude coût-efficacité de l'adaptation bayésienne des posologies des aminosides en gériatrie." Therapies 66(5): 445-452. | Language |
| 1275 | Lerdpornkulrat, T., et al. (2019). "The Positive Effect of Intrinsic Feedback on Motivational Engagement and Self-Efficacy in Information Literacy." Journal of psychoeducational assessment 37(4): 421-434. | Title |
| 1276 | Lereclus, A., et al. (2023). "External Evaluation of Population Pharmacokinetics Models of Lithium in the Bipolar Population." 16(11): 1627. | Title |
| 1277 | Lestner, J., et al. (2015). "Systemic antifungal prescribing in neonates and children: outcomes from the Antibiotic Resistance and Prescribing in European Children (ARPEC) Study." 59(2): 782-789. | Title |
| 1278 | Levey, A. S., et al. (2006). "Using standardized serum creatinine values in the modification of diet in renal disease study equation for estimating glomerular filtration rate." 145(4): 247-254. | Title |
| 1279 | Levey, A. S., et al. (2005). "Definition and classification of chronic kidney disease: A position statement from Kidney Disease: Improving Global Outcomes (KDIGO)." Kidney International 67(6): 2089-2100. | Title |
| 1280 | Lewis, R. A., et al. (2017). "Bimatoprost Sustained-Release Implants for Glaucoma Therapy: 6-Month Results From a Phase I/II Clinical Trial." American Journal of Ophthalmology 175: 137-147. | Title |
| 1281 | LeWitt, P. A., et al. (1989). "Controlled-release carbidopa/levodopa (Sinemet 50/200 CR4): clinical and pharmacokinetic studies." Neurology 39(11 Suppl 2): 45-53; discussion 59. | Title |
| 1282 | Li, D., et al. (2020). "AACC guidance document on biotin interference in laboratory tests." 5(3): 575-587. | Title |
| 1283 | Li, D., et al. (2023). "Predicting busulfan exposure in patients undergoing hematopoietic stem cell transplantation using machine learning techniques." 16(8): 751-761. | Title |
| 1284 | Li, J., et al. (2017). "In vitro activity of various antibiotics in combination with tigecycline against Acinetobacter baumannii: A systematic review and meta-analysis." 23(8): 982-993. | Title |
| 1285 | Li, L., et al. (2023). "Meropenem model-informed precision dosing in the treatment of critically ill patients: can we use it?" 12(2): 383. | Title |
| 1286 | Li, Q., et al. (2023). "Reporting standards for child health research were few and poorly implemented." Journal of Clinical Epidemiology 158: 141-148. | Title |
| 1287 | Li, X., et al. (2024). "Evaluation of Population Pharmacokinetic Models of Micafungin: Implications for Dosing Regimen Optimization in Critically Ill Patients." 16(9): 1145. | Title |

| | | |
|---|---|---|
| 1288 | Li, Z. G., et al. (2018). "Efficacy and Safety of Tofacitinib in Chinese Patients with Rheumatoid Arthritis." Chinese medical journal 131(22): 2683-2692. | Title |
| 1289 | Lieberman, A. N., et al. (1984). "Combined use of benserazide and carbidopa in Parkinson's disease." Neurology 34(2): 227-229. | Title |
| 1290 | Lilly, C. M., et al. (2018). "Comparative Effectiveness of Proton Pump Inhibitors vs Histamine Type 2 Receptor Blockers for Preventing Clinically Important Gastrointestinal Bleeding During Intensive Care: A Population-Based Study." Chest 154(3): 557-566. | Title |
| 1291 | Lim, A. C. (2010). "Prevention strategies for premature delivery in multiple pregnancy? Facts and fiction." Twin research and human genetics 13(3): 273. | Title |
| 1292 | Lim, S. Y. M., et al. (2022). "In vitro and In silico studies of interactions of cathinone with human recombinant cytochrome P450 CYP(1A2), CYP2A6, CYP2B6, CYP2C8, CYP2C19, CYP2E1, CYP2J2, and CYP3A5." Toxicology Reports 9: 759-768. | Title |
| 1293 | Lin, S., et al. (2024). "Exploring the therapeutic potential of layered double hydroxides and transition metal dichalcogenides through the convergence of rheumatology and nanotechnology using generative adversarial network." Environmental Research 241: 117262. | Title |
| 1294 | Linares, M., et al. (2019). "Collaborative intelligence and gamification for on-line malaria species differentiation." 18: 1-9. | Title |
| 1295 | Lines, R., et al. (2023). "Enhancing Safety of Drug-Device Combinations for Cardiovascular Disease: A Focus on Post-Market Safety Surveillance and Regulatory Considerations in the EU and US." 46(11): 1194-1195. | Title |
| 1296 | Ling, C. W., et al. (2024). "Systematic Review on Treatment and Outcomes of Tuberculous Peritonitis in Patients on Peritoneal Dialysis." Kidney International Reports 9(2): 277-286. | Title |
| 1297 | Linker, R. A. and G. Wendt (2016). "Cardiac safety profile of first dose of fingolimod for relapsingremitting multiple sclerosis in real world settings: data from a German prospective multicenter observational study." Multiple sclerosis (Houndmills, Basingstoke, England) 22: 795. | Title |
| 1298 | Lionetto, L., et al. (2012). "Sumatriptan succinate : pharmacokinetics of different formulations in clinical practice." Expert Opin Pharmacother 13(16): 2369-2380. | Title |
| 1299 | Lip, G. Y., et al. (2012). "Indirect comparisons of new oral anticoagulant drugs for efficacy and safety when used for stroke prevention in atrial fibrillation." 60(8): 738-746. | Title |
| 1300 | Lipner, S. R. J. J. o. t. A. A. o. D. (2018). "Rethinking biotin therapy for hair, nail, and skin disorders." 78(6): 1236-1238. | Title |

| | | |
|---|---|---|
| 1301 | Lipton, R. B., et al. (2017). "Faster Improvement in Migraine Pain Intensity and Migraine-Related Disability at Early Time Points with AVP-825 (Sumatriptan Nasal Powder Delivery System) versus Oral Sumatriptan: A Comparative Randomized Clinical Trial Across Multiple Attacks from the COMPASS Study." Headache 57(10): 1570-1582. | Title |
| 1302 | Lipton, R. B., et al. (2019). "DFN-02, Sumatriptan 10 mg Nasal Spray with Permeation Enhancer, for the Acute Treatment of Migraine: A Randomized, Double-Blind, Placebo-Controlled Study Assessing Functional Disability and Subject Satisfaction with Treatment." CNS Drugs 33(4): 375-382. | Title |
| 1303 | Liria, P., et al. (2012). "Arandomised control trial on the effectiveness of osteopathic manipulative treatment in reducing pain and improving the quality of life in elderly patients affected by osteoporosis." European Journal of Integrative Medicine 4: 27. | Title |
| 1304 | Lissitchkov, T., et al. (2020). "Fibrinogen concentrate for treatment of bleeding and surgical prophylaxis in congenital fibrinogen deficiency patients." Journal of Thrombosis and Haemostasis 18(4): 815-824. | Title |
| 1305 | Litjens, C. H. C., et al. (2020). "Preclinical models to optimize treatment of tuberculous meningitis - A systematic review." Tuberculosis (Edinb) 122: 101924. | Title |
| 1306 | Little, P., et al. (2014). "Alexander technique and supervised physiotherapy exercises in back pain (aspen) feasibility trial." Journal of alternative and complementary medicine (New York, N.Y.) 20(5): A60. | Title |
| 1307 | Liu, J. J., et al. (2015). "Functional spinal unit height and sagittal alignment of two-level CDA and ACDF patients." Spine journal 15(10 SUPPL. 1): S146. | Title |
| 1308 | Liu, Z. (2020). "Prediction of economic burden of disease treatment for diabetic patients in rural residents." Indian journal of pharmaceutical sciences 82(SUPPL 1): 50. | Title |
| 1309 | Liu, Z.-G., et al. (2022). "Is tranexamic acid beneficial in open spine surgery? and its effects vary by dosage, age, sites, and locations: a meta-analysis of randomized controlled trials." 166: 141-152. | Title |
| 1310 | Loebis, R., et al. (2021). "Correlation between the exposure time to mobile devices and the prevalence of evaporative dry eyes as one of the symptoms of computer vision syndrome among senior high school students in east Java, Indonesia." 32(4): 541-545. | Title |
| 1311 | Lohle, P. N. M., et al. (2016). "Update on VERTOS IV." Cardiovascular and interventional radiology 39(3): S123-S124. | Title |
| 1312 | Lomaestro, B. M. and L. L. Briceland (1998). "Streptogramins and their potential role in geriatric medicine." Drugs Aging 13(6): 443-465. | Title |

| | | |
|---|---|---|
| 1313 | Loonen, A. J., et al. (1989). "A comparison of carbamazepine divitabs with carbamazepine normal formulation in psychiatric and oligophrenic patients. Preliminary pharmacokinetic results." Pharm Weekbl Sci 11(1): 23-26. | Title |
| 1314 | López Aspiroz, E., et al. (2014). "Population pharmacokinetic/pharmacogenetic model of lopinavir/ritonavir in HIV-infected patients." Per Med 11(7): 693-704. | Title |
| 1315 | Lopez, J. I., et al. (2017). "Effects of onabotulinumtoxina treatment on disability and quality of life in patients with chronic migraine with baseline headache every day: a COMPEL subanalysis." Cephalalgia 37(1): 99-100. | Title |
| 1316 | López-Anguita, S., et al. (2022). "[Epilepsy in cancer patients: primary prevention and the importance of high-risk patient screening]." Rev Neurol 75(11): 349-356. | Title |
| 1317 | López-Cortés, A., et al. "CANCER PHARMACOGENOMIC STUDIES IN LATIN AMERICA." 153. | Title |
| 1318 | Lories, R. J. (2012). "Etoricoxib and the treatment of ankylosing spondylitis." Expert Opin Drug Metab Toxicol 8(12): 1599-1608. | Title |
| 1319 | Löscher, W. and P. Klein (2021). "The Pharmacology and Clinical Efficacy of Antiseizure Medications: From Bromide Salts to Cenobamate and Beyond." CNS Drugs 35(9): 935-963. | Title |
| 1320 | Louw, A. and I. Diener (2015). "Preoperative neuroscience education for lumbar radiculopathy patients-a randomised control trial." Physiotherapy (united kingdom) 101: eS317. | Title |
| 1321 | Lu, D. and W. Mao (2023). "Efficacy and safety of intravenous combined with aerosolised polymyxin versus intravenous polymyxin alone in the treatment of multidrug-resistant gram-negative bacterial pneumonia: A systematic review and meta-analysis." Heliyon 9(5): e15774. | Title |
| 1322 | Luque-Moreno, C., et al. (2012). "[Analysis of sustained attention in patients with Parkinson's disease being treated with dopamine precursors]." Rev Neurol 55(5): 257-262. | Title |
| 1323 | Luukinen, H., et al. (2006). "Prevention of disability by exercise among the elderly: a population-based, randomized, controlled trial." Scandinavian journal of primary health care 24(4): 199-205. | Title |
| 1324 | Lycke, J., et al. (2023). "Teriflunomide Concentrations in Cerebrospinal Fluid and Plasma in Patients with Multiple Sclerosis: A Pharmacokinetic Study." CNS Drugs 37(2): 181-188. | Title |
| 1325 | Maarse, B. C. E., et al. (2023). "supplementary figures and tables." 1: 104. | Title |
| 1326 | Macdonell, R., et al. (2013). "Teriflunomide reduces relapse-related sequelae, severe relapses, hospitalisations and corticosteroid use: pooled data from the phase 3 TEMSO and TOWER studies." Multiple sclerosis (Houndmills, Basingstoke, England) 19(11): 512-513. | Title |

| | | |
|---|---|---|
| 1327 | Mackay, L., et al. (2009). "Obstetric and gynaecological anaesthesia." 37: 748-752. | Title |
| 1328 | MacMaster, L. J. J. o. P. and P. Sciences (2022). "The 2022 Canadian Society for Pharmaceutical Sciences/Canadian Chapter of Controlled Release Society Annual Symposium was held virtually from June 1-2, 2022." 25: 80s-115s. | Title |
| 1329 | Maenhout, A., et al. (2011). "Eccentric training in patients with rotator cuff tendinopathy: a randomized controlled trial." Physiotherapy (united kingdom) 97: eS738. | Title |
| 1330 | Maertens, J., et al. (2016). "ECIL guidelines for preventing Pneumocystis jirovecii pneumonia in patients with haematological malignancies and stem cell transplant recipients." 71(9): 2397-2404. | Title |
| 1331 | Maghawry, K. M., et al. (2015). "Cerebral oxygen saturation monitoring during hypotensive anesthesia in shoulder arthroscopy: A comparative study between dexmedetomidine and esmolol." Egyptian Journal of Anaesthesia 31(1): 43-52. | Title |
| 1332 | Magrath, I., et al. (2005). "Treatment of acute lymphoblastic leukaemia in countries with limited resources; lessons from use of a single protocol in India over a twenty year peroid." European Journal of Cancer 41(11): 1570-1583. | Title |
| 1333 | Mahar, N., et al. (2022). "Spectroanalytical SERS-based detection of trace-level procainamide using green-synthesized gold nanoparticles." Surfaces and Interfaces 31: 102059. | Title |
| 1334 | Maher, H. M., et al. (2016). "Simultaneous determination of selected tyrosine kinase inhibitors with corticosteroids and antiemetics in rat plasma by solid phase extraction and ultra-performance liquid chromatography–tandem mass spectrometry: Application to pharmacokinetic interaction studies." J Pharm Biomed Anal 124: 216-227. | Title |
| 1335 | Maher, H. M., et al. (2017). "Comparative pharmacokinetic profiles of selected irreversible tyrosine kinase inhibitors, neratinib and pelitinib, with apigenin in rat plasma by UPLC–MS/MS." J Pharm Biomed Anal 137: 258-267. | Title |
| 1336 | Maher, T., et al. (2016). "Safety and tolerability of nintedanib in patients with idiopathic pulmonary fibrosis (IPF): one-year data from post-marketing surveillance in the United States." Thorax 71: A175. | Title |
| 1337 | Mahmoud, I., et al. (2017). "Efficacy and Safety of Rituximab in the Management of Pediatric Systemic Lupus Erythematosus: A Systematic Review." The Journal of Pediatrics 187: 213-219.e212. | Title |
| 1338 | Mai, N. T. and G. E. Thwaites (2017). "Recent advances in the diagnosis and management of tuberculous meningitis." Curr Opin Infect Dis 30(1): 123-128. | Title |

| | | |
|---|---|---|
| 1339 | Maiers, M., et al. (2012). "Spinal manipulative therapy, supervised rehabilitative exercise and home exercise for seniors with neck pain." BMC complementary and alternative medicine 12. | Title |
| 1340 | Maiti, D., et al. (2023). "Evaluation of solid-lipid nanoparticles formulation of methotrexate for anti-psoriatic activity." Saudi Pharmaceutical Journal 31(6): 834-844. | Title |
| 1341 | Makki, H. M. A. (2016). Effect of Pomegranate Juice on Cyclosporine Pharmacokinetic and Possible Nephroprotective Effect on Cyclosporine-Induced Nephrotoxicity in Rats, KING ABDULAZIZ UNIVERSITY JEDDAH. | Title |
| 1342 | Malakouti, S. K., et al. (2021). "A Systematic Review of Potentially Inappropriate Medications Use and Related Costs Among the Elderly." Value in Health Regional Issues 25: 172-179. | Title |
| 1343 | Malebari, A. M., et al. (2022). "Development of advanced 3D-printed solid dosage pediatric formulations for HIV treatment." 15(4): 435. | Title |
| 1344 | Malhotra, R., et al. (2023). "Older adult patient preferences for the content and format of prescription medication labels – A best-worst scaling and discrete choice experiment study." Research in Social and Administrative Pharmacy 19(11): 1455-1464. | Title |
| 1345 | Maliyakkal, N., et al. (2021). "Nanoparticles of cisplatin augment drug accumulations and inhibit multidrug resistance transporters in human glioblastoma cells." Saudi Pharmaceutical Journal 29(8): 857-873. | Title |
| 1346 | Malki, M. A., et al. (2021). "Utilizing Large Electronic Medical Record Data Sets to Identify Novel Drug-Gene Interactions for Commonly Used Drugs." Clin Pharmacol Ther 110(3): 816-825. | Title |
| 1347 | Malone, R. S., et al. (1999). "The Effect of Hemodialysis on Cycloserine, Ethionamide, Para-Aminosalicylate, and Clofazimine." Chest 116(4): 984-990. | Title |
| 1348 | Maltz, L. A., et al. (2013). "Off-label drug use in a single-center pediatric cardiac intensive care unit." 4(3): 262-266. | Title |
| 1349 | Malykh, A. G. and M. R. Sadaie (2010). "Piracetam and piracetam-like drugs: from basic science to novel clinical applications to CNS disorders." Drugs 70(3): 287-312. | Title |
| 1350 | Mambrin, A., et al. (2021). "A curious case of opticneuritis in hidradenitis suppurativa patient, successfullytreated with adalimumab." 30(S1): 77-77. | Title |
| 1351 | Manchikanti, L., et al. (2013). "Assessment of effectiveness of percutaneous adhesiolysis in managing chronic low back pain secondary to lumbar central spinal canal stenosis." Int J Med Sci 10(1): 50-59. | Title |

| | | |
|---|---|---|
| 1352 | Mandrusiak, A., et al. (2011). "A novel exercise program for young people with cystic fibrosis: moving physiotherapy forward through targeted design." Physiotherapy (united kingdom) 97: eS1550. | Title |
| 1353 | Mangoni, A. A. and A. Pilotto (2016). "New drugs and patient-centred end-points in old age: setting the wheels in motion." Expert Rev Clin Pharmacol 9(1): 81-89. | Title |
| 1354 | Mankar, S., et al. (2023). "Pharmacometrics: Application in Drug Development and Clinical Practice." 13(3): 210-216. | Title |
| 1355 | Manninen, H. (2011). "Endovascular is the first choice for long SFA occlusion in claudication: con." Cardiovascular and interventional radiology 34: 381-382. | Title |
| 1356 | Manning, E. and R. J. J. o. C. N. Emerson (2024). "Intraoperative Monitoring of Scoliosis Surgery in Young Patients." 41(2): 138-147. | Title |
| 1357 | Mansell, G., et al. (2016). "Exploring What Factors Mediate Treatment Effect: example of the STarT Back Study High-Risk Intervention." The journal of pain 17(11): 1237-1245. | Title |
| 1358 | Mansour, A., et al. (2023). "Fortified anti-proliferative activity of niclosamide for breast cancer treatment: In-vitro and in-vivo assessment." Life Sciences 316: 121379. | Title |
| 1359 | Maranchick, N. F., et al. (2023). "Impact of Beta-Lactam Target Attainment on Resistance Development in Patients with Gram-Negative Infections." 12(12): 1696. | Title |
| 1360 | Marantz, J., et al. (2015). "Longer-term follow-up of the efficacy of delayedrelease dimethyl fumarate in newly diagnosed patients with RRMS: an integrated analysis of DEFINE, CONFIRM, and ENDORSE." Multiple sclerosis (Houndmills, Basingstoke, England) 23(11): 263. | Title |
| 1361 | Marazziti, D., et al. (2019). "Pharmacokinetics of serotonergic drugs: focus on OCD." Expert Opin Drug Metab Toxicol 15(4): 261-273. | Title |
| 1362 | Marchetti, A., et al. (1996). "Pharmacoeconomic analysis of oral therapies for onychomycosis: a US model." Clin Ther 18(4): 757-777. | Title |
| 1363 | Mariee, A. D. and O. Al-Shabanah (2006). "Protective ability and binding affinity of captopril towards serum albumin in an in vitro glycation model of diabetes mellitus." J Pharm Biomed Anal 41(2): 571-575. | Title |
| 1364 | Marin, J. C. and P. J. Goadsby (2010). "Glutamatergic fine tuning with ADX-10059: a novel therapeutic approach for migraine?" Expert Opin Investig Drugs 19(4): 555-561. | Title |
| 1365 | Markatseli, T. E., et al. (2014). "Certolizumab for rheumatoid arthritis." Clin Exp Rheumatol 32(3): 415-423. | Title |
| 1366 | Marklund, N. and L. Hillered (2011). "Animal modelling of traumatic brain injury in preclinical drug development: where do we go from here?" Br J Pharmacol 164(4): 1207-1229. | Title |

| | | |
|---|---|---|
| 1367 | Marra, A. R., et al. (2020). "Modest Clostridiodes difficile infection prediction using machine learning models in a tertiary care hospital." Diagn Microbiol Infect Dis 98(2): 115104. | Title |
| 1368 | Marsden, D., et al. (2010). "A multidisciplinary group programme in rural settings for community-dwelling chronic stroke survivors and their carers: a pilot randomized controlled trial." Clinical rehabilitation 24(4): 328-341. | Title |
| 1369 | Marsden, P. D. and M. G. Schultz (1969). "Intestinal Parasites." Gastroenterology 57(6): 724-750. | Title |
| 1370 | Martí-Cabrera, M., et al. (2011). "[Chronic use of proton pump inhibitors: is the risk of osteoporosis and fractures real?]." Gastroenterol Hepatol 34(4): 271-277. | Title |
| 1371 | Martin, T. D., et al. (2019). "Six weeks of oral Echinacea purpurea supplementation does not enhance the production of serum erythropoietin or erythropoietic status in recreationally active males with above-average aerobic fitness." Applied physiology, nutrition & metabolism 44(7): 791-795. | Title |
| 1372 | Martinelli, D., et al. (2021). "Lasmiditan: an additional therapeutic option for the acute treatment of migraine." Expert Rev Neurother 21(5): 491-502. | Title |
| 1373 | Martínez-Castrillo, J. C., et al. (2024). "[Inhaled levodopa: from evidence to experience]." Rev Neurol 78(S01): S1-s10. | Title |
| 1374 | Martinez-Nunez, A. E. and P. A. LeWitt (2023). "Drugs to the Rescue: Comparison of On-Demand Therapies for OFF Symptoms in Parkinson's Disease." J Parkinsons Dis 13(4): 441-451. | Title |
| 1375 | Masana, L., et al. (2024). "Lomitapide for the treatment of paediatric patients with homozygous familial hypercholesterolaemia (APH-19): results from the efficacy phase of an open-label, multicentre, phase 3 study." The Lancet Diabetes & Endocrinology. | Title |
| 1376 | Masand, P. S., et al. (2004). "Safety considerations in pharmacotherapy of bipolar disorder." CNS Spectr 9(11 Suppl 12): 16-26. | Title |
| 1377 | Matar, K. M., et al. (2014). "Pharmacokinetics of artesunate alone and in combination with sulfadoxine/pyrimethamine in healthy Sudanese volunteers." 90(6): 1087. | Title |
| 1378 | Matear, D. W. and D. Clarke (1999). "Considerations for the use of oral sedation in the institutionalized geriatric patient during dental interventions: a review of the literature." Spec Care Dentist 19(2): 56-63. | Title |
| 1379 | Matovic, S., et al. (2018). "Population pharmacokinetics of 25-hydroxy vitamin D in children with asthma." 51(9). | Title |
| 1380 | Matthews, S. J. and C. McCoy (2004). "Peginterferon alfa-2a: A review of approved and investigational uses." Clin Ther 26(7): 991-1025. | Title |

| | | |
|---|---|---|
| 1381 | Mattson, R. H. (1996). "The role of the old and the new antiepileptic drugs in special populations: mental and multiple handicaps." Epilepsia 37 Suppl 6: S45-53. | Title |
| 1382 | Mauri-Llerda, J. A. (2004). "[Treatment of the epileptic patient in special situations]." Rev Neurol 38(2): 156-161. | Title |
| 1383 | May, T. W., et al. (2011). "Serum concentrations of rufinamide in children and adults with epilepsy: the influence of dose, age, and comedication." Ther Drug Monit 33(2): 214-221. | Title |
| 1384 | Mayet, A. Y., et al. (2018). "Rivaroxaban prescribing in a Saudi tertiary care teaching hospital." Saudi Pharmaceutical Journal 26(6): 775-779. | Title |
| 1385 | Mazri, R., et al. (2024). "Exploring potential therapeutics: Targeting dengue virus NS5 through molecular docking, ADMET profiling, and DFT analysis." Chemical Physics Impact 8: 100468. | Title |
| 1386 | Mazumdar-Shaw, K. (2018). "Leveraging affordable innovation to tackle India's healthcare challenge." IIMB Management Review 30(1): 37-50. | Title |
| 1387 | Mazzuca, S. A., et al. (1997). "Effects of self-care education on the health status of inner-city patients with osteoarthritis of the knee." Arthritis Rheum 40(8): 1466-1474. | Title |
| 1388 | Mc Crudden, M. T. C., et al. (2019). "Design, Formulation, and Evaluation of Novel Dissolving Microarray Patches Containing Rilpivirine for Intravaginal Delivery." Adv Healthc Mater 8(9): e1801510. | Title |
| 1389 | Mc Crudden, M. T. C., et al. (2018). "Design, formulation and evaluation of novel dissolving microarray patches containing a long-acting rilpivirine nanosuspension." J Control Release 292: 119-129. | Title |
| 1390 | McAllister, P., et al. (2015). "Breath-powered ™ nasal delivery of 22 mg powdered sumatriptan (AVP-825): migraine disability and functional outcome in a phase 3 study (TARGET)." Neurology 84. | Title |
| 1391 | McAllister, P., et al. (2014). "Breath-powered™ nasal delivery of powdered sumatriptan (AVP-825): migraine disability and functional outcome in a phase 3 study (TARGET)." Headache 54: 32-33. | Title |
| 1392 | McClurg, D., et al. (2011). "Incontinence stroke project inspiring rehabilitation excellence (inspire)-a mixed methods approach to piloting a complex intervention to improve continence care following stroke." Neurourology and urodynamics 30(6): 903-905. | Title |
| 1393 | McClurg, D., et al. (2013). "Feasibility of abdominal massage for the alleviation of symptoms of constipation in people with parkinson's." Neurourology and urodynamics 32(6): 685-686. | Title |

| | | |
|---|---|---|
| 1394 | McCormack, P. L. (2013). "Natalizumab: a review of its use in the management of relapsing-remitting multiple sclerosis." Drugs 73(13): 1463-1481. | Title |
| 1395 | McCune, J. S., et al. (2019). "Association of Antiepileptic Medications with Outcomes after Allogeneic Hematopoietic Cell Transplantation with Busulfan/Cyclophosphamide Conditioning." Biology of Blood and Marrow Transplantation 25(7): 1424-1431. | Title |
| 1396 | McEneny-King, A., et al. (2019). "Development and evaluation of a generic population pharmacokinetic model for standard half-life factor VIII for use in dose individualization." 46: 411-426. | Title |
| 1397 | McGinn, R. J., et al. (2022). "Levetiracetam vs phenytoin prophylaxis in severe traumatic brain injury: Systematic review and meta-analysis." Interdisciplinary Neurosurgery 27: 101394. | Title |
| 1398 | McGorry, P. D., et al. (2016). "NEURAPRO-E: a multicentre randomized controlled trial of omega-3 fatty acids and cognitive-behavioural case management for patients at ultra high risk of schizophrenia and other psychotic disorders." Early intervention in psychiatry 10: 40. | Title |
| 1399 | McIntosh, A. M., et al. (2023). "Comorbidities in newly diagnosed epilepsy: Pre-existing health conditions are common and complex across age groups." Epilepsy & Behavior 138: 108960. | Title |
| 1400 | McIntyre, A., et al. (2013). "A double-blind, randomized, placebocontrolled study of quetiapine XR for the treatment of major depression and fibromyalgia." European neuropsychopharmacology 23: S415. | Title |
| 1401 | McIntyre, A. W., et al. (2013). "A double-blind, randomized, placebo-controlled study evaluating the efficacy and safety of quetiapine XR for treatment of major depression and fibromyalgia." European psychiatry 28. | Title |
| 1402 | McMahon, J. H., et al. (2022). "Favipiravir in early symptomatic COVID-19, a randomised placebo-controlled trial." eClinicalMedicine 54: 101703. | Title |
| 1403 | McManus, D. P. and M. Hope (1993). "Molecular variation in the human schistosomes." Acta Tropica 53(3): 255-276. | Title |
| 1404 | Md, S. and S. Kotta (2024). "Advanced drug delivery technologies for postmenopausal effects." Journal of Controlled Release 373: 426-446. | Title |
| 1405 | Mease, P., et al. (2020). "Efficacy and Safety of Tildrakizumab, a High-Affinity Anti-Interleukin-23p19 Monoclonal Antibody, in Patients with Active Psoriatic Arthritis in a Randomized, Double-Blind, Placebo-Controlled, Multiple-Dose, Phase 2b Study." Arthritis & rheumatology 72(SUPPL 10): 4073-4075. | Title |
| 1406 | Mease, P. J., et al. (2013). "Effect of adalimumab on physical function, health-related quality of life, and work productivity in patients with peripheral spondyloarthritis: results from the ability-2 clinical trial." Ann Rheum Dis 72. | Title |

| | | |
|---|---|---|
| 1407 | Mease, P. J., et al. (2015). "A randomized, double-blind, active-and placebo-controlled phase 3 study of efficacy and safety of ixekizumab, adalimumab, and placebo therapy in patients naive to biologic disease modifying anti-rheumatic drugs with active psoriatic arthritis." Arthritis & rheumatology 67(no pagination). | Title |
| 1408 | Mechanick, J. I., et al. (2013). "Clinical Practice Guidelines For The Perioperative Nutritional, Metabolic, And Nonsurgical Support Of The Bariatric Surgery Patient2013 Update: Cosponsored By American Association Of Clinical Endocrinologists, The Obesity Society, And American Society For Metabolic & Bariatric Surgery." Endocrine Practice 19(2): 337-372. | Title |
| 1409 | Meghairbi, K. (2012). The association of antibiotic pharmacodynamic indices with survival in human septic shock, University of Manitoba (Canada). | Title |
| 1410 | Mehta, P., et al. (2015). "Meta-analysis of randomised controlled trials of biologics in DMARD-naïve and DMARD-inadequate responder subjects with rheumatoid arthritis: efficacy and safety." Ann Rheum Dis 74: 239. | Title |
| 1411 | Meilianti, S., et al. (2023). "How can pharmacists contribute to anaemia management? A review of literature and exploratory study on pharmacists' role in anaemia." Exploratory Research in Clinical and Social Pharmacy 9: 100231. | Title |
| 1412 | Melamed, E., et al. (1999). "Current management of motor fluctuations in patients with advanced Parkinson's disease treated chronically with levodopa." J Neural Transm Suppl 56: 173-183. | Title |
| 1413 | Memish, Z. A., et al. (2002). "Guidelines for the management of Community-Acquired Pneumonia in Saudi Arabia: a model for the Middle East region." International Journal of Antimicrobial Agents 20: 1-12. | Title |
| 1414 | Merae Alshahrani, M., et al. (2024). "Mechanistic and dynamic insight into novel IL7 receptor activators as immunotherapy for the treatment of tuberculosis." Journal of Molecular Liquids 414: 126040. | Title |
| 1415 | Merli, G., et al. (2001). "Subcutaneous enoxaparin once or twice daily compared with intravenous unfractionated heparin for treatment of venous thromboembolic disease." 134(3): 191-202. | Title |
| 1416 | Merschhemke, M., et al. (2015). "Fingolimod efficacy in second-line treatment for RRMS: treatment outcome is independent from previous MRI or clinical activity." Multiple sclerosis (Houndmills, Basingstoke, England) 23(11): 319-320. | Title |
| 1417 | Meyer, U. A. (2000). "Pharmacogenetics and adverse drug reactions." The Lancet 356(9242): 1667-1671. | Title |
| 1418 | Michelon, H., et al. (2021). "Atropine-induced toxicity after off-label sublingual administration of eyedrop for sialorrhoea treatment in neurological disabled patients." Br J Clin Pharmacol 87(8): 3364-3369. | Title |

| | | |
|---|---|---|
| 1419 | Mickymaray, S., et al. (2023). "Chitosan-encapsulated nickel oxide, tin dioxide, and farnesol nanoparticles: Antimicrobial and anticancer properties in breast cancer cells." International Journal of Biological Macromolecules 248: 125799. | Title |
| 1420 | Midha, K. K., et al. (1994). "Impact of clinical pharmacokinetics on neuroleptic therapy in patients with schizophrenia." J Psychiatry Neurosci 19(4): 254-264. | Title |
| 1421 | Mihai, D. P., et al. (2019). "Computational Drug Repurposing Algorithm Targeting TRPA1 Calcium Channel as a Potential Therapeutic Solution for Multiple Sclerosis." Pharmaceutics 11(9). | Title |
| 1422 | Mikol, D., et al. (2014). "Correlations between patient-reported ambulatory function (MSWS-12) and objective disability measurements in SPMS: analysis of ASCEND baseline data." Multiple sclerosis (Houndmills, Basingstoke, England) 20(1): 408. | Title |
| 1423 | Mikol, D., et al. (2013). "Ascend study of natalizumab efficacy on disability in patients with secondary progressive multiple sclerosis (SPMS): baseline demographics and disease characteristics." Ann Neurol 74: S59-S60. | Title |
| 1424 | Mikol, D., et al. (2015). "The 9-hole peg test (9-HPT) has a stronger correlation than the expanded disability status scale (EDSS) with patient-reported upper extremity impairment as assessed using ABILHAND in patients with secondary progressive multiple sclerosis (SPMS): analysis of baseline data from the ascend study." Neurology 84. | Title |
| 1425 | Millar, N. L., et al. (2021). "Efficacy and safety of secukinumab in patients with rotator cuff tendinopathy: a 24-week, randomised, double-blind, placebocontrolled, phase ii proof-of-concept trial." Ann Rheum Dis 80(SUPPL 1): 211-212. | Title |
| 1426 | Miller, A. E. (2017). "Teriflunomide in multiple sclerosis: an update." Neurodegenerative disease management 7(1): 9-29. | Title |
| 1427 | Miller, D. H., et al. (2012). "Effect of BG-12 on magnetic resonance imaging activity in subgroups of patients with relapsing-remitting multiple sclerosis: findings from the CONFIRM study." Multiple sclerosis (Houndmills, Basingstoke, England) 18(4): 189-190. | Title |
| 1428 | Mills, I. S., et al. (2018). "Rates of early intervention services in children born extremely preterm/extremely low birthweight." Journal of paediatrics and child health 54(1): 74-79. | Title |
| 1429 | Milyani, A. A. and A. E. Al-Agha "The onset of asymptomatic amiodarone-induced thyrotoxicosis in an adolescent male." | Title |
| 1430 | Minardi, M., et al. (2021). Common and rare hematological manifestations and adverse drug events during treatment of active TB: a state of art. Microorganisms 2021; 9: 1477, s Note: MDPI stays neutral with regard to jurisdictional claims in published …. | Title |
| 1431 | Minassian, V. A., et al. (2007). "Randomized Trial of Oxybutynin Extended Versus Immediate Release for Women Aged 65 and Older with Overactive Bladder: Lessons Learned from Conducting a Trial." Journal of Obstetrics and Gynaecology Canada 29(9): 726-732. | Title |

| | | |
|---|---|---|
| 1432 | Minhas, J. S., et al. (2018). "Lipid-Lowering Pretreatment and Outcome Following Intravenous Thrombolysis for Acute Ischaemic Stroke: a Post Hoc Analysis of the Enhanced Control of Hypertension and Thrombolysis Stroke Study Trial." Cerebrovascular diseases (Basel, Switzerland) 45(5-6): 213-220. | Title |
| 1433 | Miralles, R., et al. (2007). "Neonatology and perinatology." 34: 247-249. | Title |
| 1434 | Mirza, R., et al. (2024). "Brain targeting of cefepime loaded transfersomes based thermosensitive in situ gel via intranasal delivery: In vitro and in vivo studies." Journal of Drug Delivery Science and Technology 95: 105585. | Title |
| 1435 | Mitragotri, S. and J. W. Yoo (2011). "Designing micro- and nano-particles for treating rheumatoid arthritis." Arch Pharm Res 34(11): 1887-1897. | Title |
| 1436 | Mittal, M., et al. (2011). "Mild strokes with large vessel occlusion." Stroke 42(3): e323. | Title |
| 1437 | Mittal, P., et al. (2024). "Evaluation of lavender and rose aromatherapies on the success of inferior alveolar nerve block in symptomatic irreversible pulpitis: A randomized clinical trial." Heliyon 10(14): e34514. | Title |
| 1438 | Mo, Y. and D. J. J. o. A. C. Fisher (2016). "A review of treatment modalities for Middle East Respiratory Syndrome." 71(12): 3340-3350. | Title |
| 1439 | Moghddam, S. R. M., et al. (2016). "Formulation and optimization of niosomes for topical diacerein delivery using 3-factor, 3-level Box-Behnken design for the management of psoriasis." Materials Science and Engineering: C 69: 789-797. | Title |
| 1440 | Mohamadpour, M., et al. (2019). "The Importance of Therapeutic Time Window in the Treatment of Traumatic Brain Injury." Front Neurosci 13: 07. | Title |
| 1441 | Mohamed, T. (2018). A Comparative Study of Cutaneous Manifestations in Obese Patients and Non-Obese Controls at Vims, Ballari, Rajiv Gandhi University of Health Sciences (India). | Title |
| 1442 | Mohammad, T., et al. (2024). "Identifying potential inhibitors of phosphatidylinositol 4,5-bisphosphate 3-kinase: Molecular dynamic insights into the interaction and inhibitory mechanism." Chemical Physics Impact 8: 100458. | Title |
| 1443 | Mohammed Alkreathy, H., et al. (2020). "Bisoprolol responses (PK/PD) in hypertensive patients: A cytochrome P450 (CYP) 2D6 targeted polymorphism study." Saudi Journal of Biological Sciences 27(10): 2727-2732. | Title |
| 1444 | Mohammed, A. T., et al. (2020). "The role of sulpiride in attenuating the cardiac, renal, and immune disruptions in rats receiving clozapine: mRNA expression pattern of the genes encoding Kim-1, TIMP-1, and CYP isoforms." 27: 25404-25414. | Title |

| | | |
|---|---|---|
| 1445 | Mohd Sazlly Lim, S., et al. (2021). "Semi-mechanistic PK/PD modelling of meropenem and sulbactam combination against carbapenem-resistant strains of Acinetobacter baumannii."  40: 1943-1952. | Title |
| 1446 | Mohd Siddique, M. U., et al. (2021). "Non-carboxylic acid inhibitors of aldose reductase based on N-substituted thiazolidinedione derivatives." European Journal of Medicinal Chemistry 223: 113630. | Title |
| 1447 | Mok, C. C. (2018). "Morning Stiffness in Elderly Patients with Rheumatoid Arthritis: What is Known About the Effect of Biological and Targeted Agents?" Drugs Aging 35(6): 477-483. | Title |
| 1448 | Mokhtari, T. and A. El-Meghawry El-Kenawy (2024). "Molecular mechanisms of Schisandra chinensis in treating depression-neuropathic pain comorbidity by network pharmacology and molecular docking analysis." Neuroscience 555: 92-105. | Title |
| 1449 | Molina, R. F., et al. (2024). "“Population pharmacokinetics analyses and therapeutic drug monitoring of vancomycin: a scoping review”." | Title |
| 1450 | Momattin, H., et al. (2018). "Benchmarking of antibiotic usage: An adjustment to reflect antibiotic stewardship program outcome in a hospital in Saudi Arabia." Journal of Infection and Public Health 11(3): 310-313. | Title |
| 1451 | Momin, Y. H., et al. (2024). "Computational investigation of 2, 4-Di Tert Butyl Phenol as alpha amylase inhibitor isolated from Coccinia grandis (L.) Voigt using molecular docking, and ADMET parameters." Comput Biol Chem 110: 108087. | Title |
| 1452 | Montagnese, S., et al. (2021). "A pilot study of golexanolone, a new GABA-A receptor-modulating steroid antagonist, in patients with covert hepatic encephalopathy." J Hepatol 75(1): 98-107. | Title |
| 1453 | Montalban, X., et al. (2015). "Baseline demographics and disease characteristics from ORATORIO, a phase III trial evaluating ocrelizumab in patients with primary progressive multiple sclerosis." Eur J Neurol 22: 705. | Title |
| 1454 | Montalban, X., et al. (2015). "Efficacy and safety of ocrelizumab in primary progressive multiple sclerosis-results of the placebo-controlled, double-blind, Phase III ORATORIO study." Multiple sclerosis (Houndmills, Basingstoke, England) 23(11): 781-782. | Title |
| 1455 | Montalban, X., et al. (2021). "Effectiveness and tolerability of ofatumumab versus first-line DMTs in early RMS patients: phase 3b STHENOS study design." Eur J Neurol 28(SUPPL 1): 357-358. | Title |
| 1456 | Montorsi, F., et al. (2005). "Frequently asked questions about tadalafil for treating men with erectile dysfunction." The Journal of Men's Health & Gender 2(1): 141-157. | Title |
| 1457 | Moondra, P. and J. Jimenez-Shahed (2024). "Profiling deutetrabenazine extended-release tablets for tardive dyskinesia and chorea associated with Huntington's disease." Expert Rev Neurother 24(9): 849-863. | Title |

| | | |
|---|---|---|
| 1458 | Moore, J. E. and B. C. J. L. Millar (2024). "Readability of Patient-Facing Information of Antibiotics Used in the WHO Short 6-Month and 9-Month All Oral Treatment for Drug-Resistant Tuberculosis." 202(5): 741-751. | Title |
| 1459 | MOORE, R. D., et al. (1984). "Risk factors for nephrotoxicity in patients treated with aminoglycosides." 100(3): 352-357. | Title |
| 1460 | Mori, L., et al. (2016). "Sensitive outcome measures in charcot-marie-tooth type 1A (CMT1A) neuropathy." Journal of the peripheral nervous system. Conference: 6th annual meeting of the italian association for the study of the peripheral nervous system, ASNP 2016. Palermo italy. Conference start: 20160414. Conference end: 20160416. Conference publication: (var.pagings) 21: S21. | Title |
| 1461 | Morrow, A., et al. (2014). "Bright Hearts: development of a biofeedback controlled interactive artwork for the management of procedural pain and anxiety in children." Developmental medicine and child neurology 56: 14. | Title |
| 1462 | Moss, B. P., et al. (2018). "Real-world experience with ocrelizumab." Multiple sclerosis journal 24(1): 31. | Title |
| 1463 | Mostafa, G. A. E., et al. (2020). Chapter Seven - Piroxicam. Profiles of Drug Substances, Excipients and Related Methodology. H. G. Brittain, Academic Press. 45: 199-474. | Title |
| 1464 | Mostafa, M. Y. A., et al. (2021). "Assessment of absorbed dose for Zr-89, Sm-153 and Lu-177 medical radioisotopes: IDAC-Dose2.1 and OLINDA experience." Applied Radiation and Isotopes 176: 109841. | Title |
| 1465 | Mostafa, T. and M. F. Alghobary (2022). "Recreational Use of Oral PDE5 Inhibitors: The Other Side of Midnight." Sexual Medicine Reviews 10(3): 392-402. | Title |
| 1466 | Mostofa, M. G., et al. (2024). "Apoptosis-inducing anti-proliferative and quantitative phytochemical profiling with in silico study of antioxidant-rich Leea aequata L. leaves." Heliyon 10(1): e23400. | Title |
| 1467 | Moura, R. A. and J. E. Fonseca (2020). "JAK Inhibitors and Modulation of B Cell Immune Responses in Rheumatoid Arthritis." Front Med (Lausanne) 7: 607725. | Title |
| 1468 | Mousa, D. H., et al. (2004). "Alpha-interferon with ribavirin in the treatment of hemodialysis patients with hepatitis c." Transplantation Proceedings 36(6): 1831-1834. | Title |
| 1469 | Moustafa, A. H., et al. (2024). "Novel guanidine derivatives targeting leukemia as selective Src/Abl dual inhibitors: Design, synthesis and anti-proliferative activity." Bioorganic Chemistry 147: 107410. | Title |
| 1470 | Moustafa, B., et al. (2024). "Egyptian pediatric kidney transplantation: highlights on post-transplant follow-up and management of complications by the Egyptian Pediatric Clinical Practice Guidelines Committee (EPG) Nephrology Group." 72(1): 85. | Title |

| | | |
|---|---|---|
| 1471 | Moyade, P. and S. J. N. M. C. Vinjamuri (2019). "British Nuclear Medicine Society 47 th Annual Spring Meeting, Oxford 1 st–3rd April 2019." 40(4): 393-453. | Title |
| 1472 | Mphahlele, M. J., et al. (2024). "Synthesis, structure of the N-(Alkyl/Arylsulfonyl) substituted 5-(Bromo/Iodo)-3-methylindazoles and bioactivity screening against some of the biochemical targets linked to type 2 diabetes mellitus." Journal of Molecular Structure 1312: 138636. | Title |
| 1473 | Mphahlele, M. J., et al. (2024). "Design, synthesis and evaluation of the 2′-hydroxy-3′-iodo-5′-nitrochalcones for cytotoxicity (MCF-7 & A549) and potential to inhibit tyrosine kinase (VEGFR-2) activity." Journal of Molecular Structure 1305: 137785. | Title |
| 1474 | Mu, R., et al. (2013). "Effectiveness and safety of hydrogel patch containing loxoprofen sodium in patients with knee osteoarthritis." Int J Rheum Dis 16: 88-89. | Title |
| 1475 | Mubeen, B., et al. (2021). "Nanotechnology as a novel approach in combating microbes providing an alternative to antibiotics." 10(12): 1473. | Title |
| 1476 | Mueller, B. A., et al. (2019). "Pregnancy course, infant outcomes, rehospitalization, and mortality among women with intellectual disability." Disability and health journal 12(3): 452-459. | Title |
| 1477 | Mulberg, A. E., et al. (2013). Pediatric drug development, John Wiley & Sons. | Title |
| 1478 | Müller, T. (2012). "Drug therapy in patients with Parkinson's disease." Transl Neurodegener 1(1): 10. | Title |
| 1479 | Müller, T. (2013). "Pharmacokinetic considerations for the use of levodopa in the treatment of Parkinson disease: focus on levodopa/carbidopa/entacapone for treatment of levodopa-associated motor complications." Clin Neuropharmacol 36(3): 84-91. | Title |
| 1480 | Müller, T. and J. D. Möhr (2019). "Recent Clinical Advances in Pharmacotherapy for Levodopa-Induced Dyskinesia." Drugs 79(13): 1367-1374. | Title |
| 1481 | Munawar, Z., et al. (2024). "Synthesis, characterization, and molecular docking studies of novel hippuric acid anhydrides as potential antiurolithic, analgesic and free radical scavenging agents." Journal of Saudi Chemical Society 28(5): 101902. | Title |
| 1482 | Munshi, A. and V. Sharma (2015). "Genetic signatures in the treatment of stroke." Curr Pharm Des 21(3): 343-354. | Title |
| 1483 | Murphy, G. S. and E. C. Oldfield (1996). "FALCIPARUM MALARIA* *The opinions expressed in this chapter are those of the authors and are not to be construed as representing the official policy of the U.S. Navy or the Naval Service at large." Infectious Disease Clinics of North America 10(4): 747-775. | Title |
| 1484 | Murphy, S. E., et al. (2013). "The effectiveness of a stratified group intervention using the STarTBack screening tool in patients with LBP--a non randomised controlled trial." BMC musculoskeletal disorders 14: 342. | Title |

| | | |
|---|---|---|
| 1485 | Murugesan, A., et al. (2024). "Design, synthesis and anticancer evaluation of novel arylhydrazones of active methylene compounds." International Journal of Biological Macromolecules 254: 127909. | Title |
| 1486 | Musher, D. M. and A. R. J. N. E. J. o. M. Thorner (2014). "Community-acquired pneumonia." 371(17): 1619-1628. | Title |
| 1487 | Mushtaq, S., et al. (2022). "Magnetoelectric core–shell CoFe2O4@BaTiO3 nanorods: their role in drug delivery and effect on multidrug resistance pump activity in vitro††Electronic supplementary information (ESI) available. See https://doi.org/10.1039/d2ra03429h." RSC Advances 12(38): 24958-24979. | Title |
| 1488 | Mushtaq, S., et al. (2021). "Biocompatibility and cytotoxicity in vitro of surface-functionalized drug-loaded spinel ferrite nanoparticles." Beilstein Journal of Nanotechnology 12: 1339-1364. | Title |
| 1489 | Musshoff, F., et al. (2010). "Pharmacogenetics and forensic toxicology." Forensic Science International 203(1): 53-62. | Title |
| 1490 | Mustafa, A. A. and I. A. J. J. o. T. U. M. S. Al-Hoqail (2013). "Biologic systemic therapy for moderate-to-severe psoriasis: a review." 8(3): 142-150. | Title |
| 1491 | Muthuri, S. G., et al. (2014). "Effectiveness of neuraminidase inhibitors in reducing mortality in patients admitted to hospital with influenza A H1N1pdm09 virus infection: a meta-analysis of individual participant data." The Lancet Respiratory Medicine 2(5): 395-404. | Title |
| 1492 | Mylius, V., et al. (2021). "Diagnosis and Management of Pain in Parkinson's Disease: A New Approach." Drugs Aging 38(7): 559-577. | Title |
| 1493 | Nagle, A., et al. (2011). "Efficacy study of vesicular gel containing methotrexate and menthol combination on parakeratotic rat skin model." J Liposome Res 21(2): 134-140. | Title |
| 1494 | Nagler, A., et al. (2015). "Outcomes after use of two standard ablative regimens in patients with refractory acute myeloid leukaemia: a retrospective, multicentre, registry analysis." The Lancet Haematology 2(9): e384-e392. | Title |
| 1495 | Naguib, M., et al. (1997). "Clinically significant drug interactions with general anesthetics--incidence, mechanims and management." Middle East J Anaesthesiol 14(3): 127-183. | Title |
| 1496 | Nahhas, A. F. and T. J. Webster (2024). "Applications of peptide-functionalized or unfunctionalized selenium nanoparticles for the passivation of SARS-CoV-2 variants and the respiratory syncytial virus (RSV)." Colloids and Surfaces B: Biointerfaces 233: 113638. | Title |
| 1497 | Nair, A. B., et al. (2020). "Mucoadhesive buccal film of almotriptan improved therapeutic delivery in rabbit model." Saudi Pharmaceutical Journal 28(2): 201-209. | Title |

| | | |
|---|---|---|
| 1498 | Nair, A. B., et al. (2018). "Development and evaluation of palonosetron loaded mucoadhesive buccal films." Journal of Drug Delivery Science and Technology 47: 351-358. | Title |
| 1499 | Nair, A. B., et al. (2018). "Influence of skin permeation enhancers on the transdermal delivery of palonosetron: An in vitro evaluation." Journal of Applied Biomedicine 16(3): 192-197. | Title |
| 1500 | Nair, R., et al. (2019). "Psychometric properties of the Child Oral-care Performance Assessment Scale." Community dentistry and oral epidemiology 47(5): 424-430. | Title |
| 1501 | Najjar, T. A., et al. (2009). "Mechanism and implication of cephalosporin penetration into oropharyngeal mucosa." Journal of Infection and Chemotherapy 15(2): 70-74. | Title |
| 1502 | Nakimuli-Mpungu, E., et al. (2020). "Effectiveness and cost-effectiveness of group support psychotherapy delivered by trained lay health workers for depression treatment among people with HIV in Uganda: a cluster-randomised trial." The Lancet. Global health 8(3): e387-e398. | Title |
| 1503 | Nam, R., et al. "Selected Abstracts from Pharmacology 2022." 2480(86): 2680-2150. | Title |
| 1504 | Nantasanti, S., et al. (2016). "Rb and p53 Liver Functions Are Essential for Xenobiotic Metabolism and Tumor Suppression." PLoS One 11(3): e0150064. | Title |
| 1505 | Narapureddy, B. and D. Dubey (2019). "Clinical evaluation of dimethyl fumarate for the treatment of relapsing-remitting multiple sclerosis: efficacy, safety, patient experience and adherence." Patient preference and adherence 13: 1655-1666. | Title |
| 1506 | Naser, M., et al. (2022). "Nanotechnology in Sepsis: Diagnosis and Treatment." | Title |
| 1507 | Nash, P., et al. (2019). "Tofacitinib as monotherapy following methotrexate withdrawal in patients with psoriatic arthritis previously treated with open-label tofacitinib + methotrexate: a randomized, placebo-controlled sub-study of opal balance." Arthritis & rheumatology 71: 5250-5253. | Title |
| 1508 | Nassir, A. M., et al. (2019). "Surface functionalized folate targeted oleuropein nano-liposomes for prostate tumor targeting: In vitro and in vivo activity." Life Sciences 220: 136-146. | Title |
| 1509 | Nasykhova, Y. A., et al. (2020). "Pharmacogenetics of type 2 diabetes—progress and prospects." 21(18): 6842. | Title |
| 1510 | Nation, R. L., et al. (2019). "Polymyxin acute kidney injury: dosing and other strategies to reduce toxicity." 8(1): 24. | Title |
| 1511 | Naushad, S. M., et al. (2019). "Recipient ABCB1, donor and recipient CYP3A5 genotypes influence tacrolimus pharmacokinetics in liver transplant cases." 71: 385-392. | Title |

| | | |
|---|---|---|
| 1512 | Naveed, M., et al. (2023). "Artificial intelligence assisted pharmacophore design for philadelphia chromosome-positive leukemia with gamma-tocotrienol: A toxicity comparison approach with asciminib." 11(4): 1041. | Title |
| 1513 | Nawaz, A., et al. (2023). "Quercetin and chlorogenic acid as bioactive compounds show promising docking site interaction and reveal these bioactive compounds as potential targets for rheumatoid arthritis." Informatics in Medicine Unlocked 43: 101388. | Title |
| 1514 | Nayak, A., et al. (2024). "Novel ibuprofen prodrug: A possible promising agent for the management of complications of Alzheimer's disease." Saudi Pharmaceutical Journal 32(1): 101888. | Title |
| 1515 | Nayak-Rao, S. (2011). "Achieving effective pain relief in patients with chronic kidney disease: a review of analgesics in renal failure." J Nephrol 24(1): 35-40. | Title |
| 1516 | Nederfors, T., et al. (2004). "Effects of furosemide and bendroflumethiazide on saliva flow rate and composition." Archives of Oral Biology 49(7): 507-513. | Title |
| 1517 | Negru, P. A., et al. (2022). "Therapeutic dilemmas in addressing SARS-CoV-2 infection: Favipiravir versus Remdesivir." 147: 112700. | Title |
| 1518 | Neonates, I. and L. Fernandez "Infections in the Immunocompromised Host June 27—30, 2004, Granada, Spain." | Title |
| 1519 | Nerenstone, S. and M. Friedman (1987). "Medical Treatment of Hepatocellular Carcinoma." Gastroenterology Clinics of North America 16(4): 603-612. | Title |
| 1520 | Nerenstone, S. R., et al. (1988). "Clinical trials in primary hepatocellular carcinoma: current status and future directions." Cancer Treatment Reviews 15(1): 1-31. | Title |
| 1521 | NEU, H. C. J. A. o. I. M. (1982). "The new beta-lactamase-stable cephalosporins." 97(3): 408-419. | Title |
| 1522 | Neva, M., et al. (2015). "Quality of life and disability: can they be improved by active postoperative rehabilitation after spinal fusion surgery? a randomised controlled trial with 12-month follow-up." European spine journal 24(6): S692. | Title |
| 1523 | Neviani, F., et al. (2017). "Physical exercise for late life depression: effects on cognition and disability." International psychogeriatrics 29(7): 1105-1112. | Title |
| 1524 | Newsome, S., et al. (2015). "Peginterferon beta-1a is effective as early as twelve weeks following treatment initiation in patients with relapsing multiple sclerosis." Neurology 84. | Title |
| 1525 | Newsome, S., et al. (2012). "Evaluating the effects of functional electrical stimulation on ambulation in individuals with secondary progressive multiple sclerosis." Multiple sclerosis (Houndmills, Basingstoke, England) 18(4): 484-485. | Title |

| | | |
|---|---|---|
| 1526 | Newsome, S. D., et al. (2023). "Subcutaneous Ocrelizumab in Patients With Multiple Sclerosis: results of the Phase III OCARINA II Study." Multiple sclerosis journal 29(3): 1060-1061. | Title |
| 1527 | Newsome, S. D., et al. (2023). "Subcutaneous Ocrelizumab in Patients with Multiple Sclerosis: results of the Phase III OCARINA II Study Authors." Multiple sclerosis and related disorders 80. | Title |
| 1528 | Ng, K. T., et al. (2020). "The effect of melatonin on delirium in hospitalised patients: A systematic review and meta-analyses with trial sequential analysis." Journal of Clinical Anesthesia 59: 74-81. | Title |
| 1529 | Ng, T. P., et al. (2010). "Cognitive function in the elderly." Asia-Pacific psychiatry 2(3): A21-A22. | Title |
| 1530 | Nguyen, T.-N., et al. (2017). "Community perceptions of targeted anti-malarial mass drug administrations in two provinces in Vietnam: a quantitative survey." 16: 1-12. | Title |
| 1531 | Nicholas, J., et al. (2013). "Combination interferon-beta 1a and mycophenolate mofetil in relapsing remitting multiple sclerosis: effects on safety, relapse rate and disability." Neurology 80(1). | Title |
| 1532 | NIGERIANS, H. I. D. (2014). "ANALYSIS OF THE USE OF MEDICINE DURING PREGNANCY IN KYRGYZSTAN." | Title |
| 1533 | Nooh, N., et al. (2013). "Effect of remifentanil on the hemodynamic responses and recovery profile of patients undergoing single jaw orthognathic surgery." International Journal of Oral and Maxillofacial Surgery 42(8): 988-993. | Title |
| 1534 | Noorulla, K. M., et al. (2024). "Syrupy herbal formulation of green bean pod extract of Phaseolus vulgaris L.: Formulation optimization by central composite design, and evaluation for anti-urolithiatic activity." Heliyon 10(5): e27330. | Title |
| 1535 | Noth, I., et al. (2016). "Safety and tolerability of nintedanib in patients with idiopathic pulmonary fibrosis (IPF): one-year data from post-marketing surveillance in the united states." QJM : monthly journal of the Association of Physicians 109: S51-S52. | Title |
| 1536 | Nourbakhsh, B., et al. (2016). "Fatigue and depression predict quality of life in patients with early multiple sclerosis: a longitudinal study." Eur J Neurol 23(9): 1482-1486. | Title |
| 1537 | Nowacki, M., et al. (2018). "Multicenter comprehensive methodological and technical analysis of 832 pressurized intraperitoneal aerosol chemotherapy (PIPAC) interventions performed in 349 patients for peritoneal carcinomatosis treatment: An international survey study." European Journal of Surgical Oncology 44(7): 991-996. | Title |
| 1538 | Nur, A. O., et al. (2015). "Pharmaceutical Evaluation of Different Tablet Brands of Letrozole and Imatinib Mesylate Marketed in Libya." | Title |

| | | |
|---|---|---|
| 1539 | Nüßlein, H. G., et al. (2016). "Efficacy and prognostic factors of treatment retention with intravenous abatacept for rheumatoid arthritis: 24-month results from an international, prospective, real-world study." Clin Exp Rheumatol 34(3): 489-499. | Title |
| 1540 | Nwanosike, E. M. (2023). "DIRECT ORAL ANTICOAGULANTS (DOACs) USE IN PATIENTS WITH RENAL INSUFFICIENCY AND OBESITY." | Title |
| 1541 | Nzila, A., et al. (2014). "Impact of folate supplementation on the efficacy of sulfadoxine/pyrimethamine in preventing malaria in pregnancy: the potential of 5-methyl-tetrahydrofolate." 69(2): 323-330. | Title |
| 1542 | O'Brien, J. D., et al. (1988). "Effect of codeine and loperamide on upper intestinal transit and absorption in normal subjects and patients with postvagotomy diarrhoea." Gut 29(3): 312-318. | Title |
| 1543 | O'Connor, P., et al. (2013). "Teriflunomide reduces relapse-related sequelae, hospitalizations and corticosteroid use: a post-HOC analysis of the phase 3 tower study." Neurology 80(1). | Title |
| 1544 | O'Dell, M. W., et al. (2015). "Abobotulinumtoxina (Dysport®) in the treatment of adult patients with upper limb spasticity due to traumatic brain injury." PM and r 7(9): S103. | Title |
| 1545 | O'Donnell, J., et al. (1995). "Preventing school failure, drug use, and delinquency among low-income children: long-term intervention in elementary schools." American journal of orthopsychiatry 65(1): 87-100. | Title |
| 1546 | OH, N. "GABA Pathway." | Title |
| 1547 | Olds, D. L., et al. (2007). "Programs for parents of infants and toddlers: recent evidence from randomized trials." Journal of child psychology and psychiatry, and allied disciplines 48(3-4): 355-391. | Title |
| 1548 | Olsson, T., et al. (2014). "Patients free of clinical ms activity in temso and tower: pooled analyses of two phase 3 placebo-controlled trials." Neurology 82(10). | Title |
| 1549 | Omar, A. M., et al. (2020). "Novel molecular discovery of promising amidine-based thiazole analogues as potent dual Matrix Metalloproteinase-2 and 9 inhibitors: Anticancer activity data with prominent cell cycle arrest and DNA fragmentation analysis effects." Bioorganic Chemistry 101: 103992. | Title |
| 1550 | Omar, A. M., et al. (2020). "The rational design, synthesis, and antimicrobial investigation of 2-Amino-4-Methylthiazole analogues inhibitors of GlcN-6-P synthase." Bioorganic Chemistry 99: 103781. | Title |
| 1551 | Omran, Z., et al. (2020). "Repurposing disulfiram as an anti-obesity drug: Treating and preventing obesity in high-fat-fed rats." 1473-1480. | Title |

| | | |
|---|---|---|
| 1552 | Omrani, A. S., et al. (2015). "High dose intravenous colistin methanesulfonate therapy is associated with high rates of nephrotoxicity; a prospective cohort study from Saudi Arabia." 14: 1-6. | Title |
| 1553 | Ong, P.-S., et al. (2016). "A novel combinatorial strategy using Seliciclib® and Belinostat® for eradication of non-small cell lung cancer via apoptosis induction and BID activation." Cancer Letters 381(1): 49-57. | Title |
| 1554 | Oomens, M. A. and T. Forouzanfar (2015). "Pharmaceutical Management of Trigeminal Neuralgia in the Elderly." Drugs Aging 32(9): 717-726. | Title |
| 1555 | Orbai, A. M., et al. (2018). "Efficacy and safety of ixekizumab in patients with active psoriatic arthritis and previous inadequate response to TNF inhibitors: two-year follow-up from a phase 3 study." Arthritis & rheumatology 70: 2850-2852. | Title |
| 1556 | Orwig, D., et al. (2013). "The relationship between sarcopenia and bone mineral density over the year post hip fracture." Journal of bone and mineral research 28. | Title |
| 1557 | Oshikoya, K. A., et al. (2009). "Pathophysiological changes that affect drug disposition in protein-energy malnourished children." 6: 1-7. | Title |
| 1558 | Osman, K. M., et al. (2018). "Poultry hatcheries as potential reservoirs for antimicrobial-resistant Escherichia coli: A risk to public health and food safety." 8(1): 5859. | Title |
| 1559 | Otani, K., et al. (1990). "Biperiden and piroheptine do not affect the serum level of zotepine, a new antipsychotic drug." Br J Psychiatry 157: 128-130. | Title |
| 1560 | Otręba, M., et al. (2020). "Antiviral activity of chlorpromazine, fluphenazine, perphenazine, prochlorperazine, and thioridazine towards RNA-viruses. A review." European Journal of Pharmacology 887: 173553. | Title |
| 1561 | Oumar, I. H. I. (2022). Formulation of Tacrolimus Oral Delivery System with Enhanced Pharmacological Profile, King Abdulaziz University Jeddah-Saudi Arabia. | Title |
| 1562 | Overdevest, G., et al. (2010). "(Cost) effectiveness of surgery versus prolonged conservative treatment in lumbar stenosis: design of a randomized controlled trial." Osteoarthritis and cartilage 18: S230-S231. | Title |
| 1563 | Owens, N. J., et al. (1994). "Distinguishing between the fit and frail elderly, and optimising pharmacotherapy." Drugs Aging 4(1): 47-55. | Title |
| 1564 | Oxley, K. and P. J. J. A. C. C. Hawkey (1999). "Antimicrobial agents: bacterial/fungal." 34: 1435-1439. | Title |
| 1565 | Oxman, T. E. (1996). "Antidepressants and cognitive impairment in the elderly." J Clin Psychiatry 57 Suppl 5: 38-44. | Title |

| | | |
|---|---|---|
| 1566 | Pacchiarotti, I. P., et al. (2009). "Predominant polarity as an outcome predictor in a clinical trial for bipolar depression." European neuropsychopharmacology 19: S448-S449. | Title |
| 1567 | Pacifico, L. and C. Chiesa (2002). "Azithromycin in children: A critical review of the evidence." Current Therapeutic Research 63(1): 54-76. | Title |
| 1568 | Pai, M. P. J. A. P. (2016). "Pharmacodynamics and Obesity." 599-620. | Title |
| 1569 | Paíga, P., et al. (2019). "Assessment of 83 pharmaceuticals in WWTP influent and effluent samples by UHPLC-MS/MS: Hourly variation." Science of The Total Environment 648: 582-600. | Title |
| 1570 | Paliwal, A., et al. (2024). "Predictive Modelling in pharmacokinetics: from in-silico simulations to personalized medicine." 20(4): 181-195. | Title |
| 1571 | Pan, A., et al. (2008). "Registered and investigational drugs for the treatment of methicillin-resistant Staphylococcus aureus infection." 3(1): 10-33. | Title |
| 1572 | Pan, F., et al. (2023). "DFT, molecular docking, and ADMET studies for the adsorption behavior and anti-inflammatory activity of thiazole by B12N12 and OH-B12N12 nanoclusters." Diamond and Related Materials 136: 110044. | Title |
| 1573 | Pan, T.-Y., et al. (2021). "Rapid simultaneous clinical monitoring of five oral anti-coagulant drugs in human urine using green microextraction technique coupled with LC–MS/MS." Journal of King Saud University - Science 33(8): 101602. | Title |
| 1574 | Paneerselvam, G. S., et al. (2024). "Pharmacist and veterinarian collaboration in Klang Valley, Malaysia: A veterinarians' perception." Heliyon 10(19): e38423. | Title |
| 1575 | Panikar, S., et al. (2021). "Essential oils as an effective alternative for the treatment of COVID-19: Molecular interaction analysis of protease (Mpro) with pharmacokinetics and toxicological properties." Journal of Infection and Public Health 14(5): 601-610. | Title |
| 1576 | Panitch, H. S., et al. (2006). "Randomized, controlled trial of dextromethorphan/quinidine for pseudobulbar affect in multiple sclerosis." Ann Neurol 59(5): 780-787. | Title |
| 1577 | Panjasawatwong, N., et al. (2020). "Population Pharmacokinetic Properties of Antituberculosis Drugs in Vietnamese Children with Tuberculous Meningitis." Antimicrob Agents Chemother 65(1). | Title |
| 1578 | Paolisso, G. (2010). "Pathophysiology of diabetes in elderly people." Acta Biomed 81 Suppl 1: 47-53. | Title |
| 1579 | Papastergiou, J., et al. (2021). "Pharmacogenomics guided versus standard antidepressant treatment in a community pharmacy setting: a randomized controlled trial." Clinical and translational science 14(4): 1359-1368. | Title |

| | | |
|---|---|---|
| 1580 | Paradisi, J., et al. (2001). "Streptococcus pneumoniae as an agent of nosocomial infection: treatment in the era of penicillin-resistant strains." Clinical Microbiology and Infection 7: 34-42. | Title |
| 1581 | Paramarta, J. E., et al. (2012). "Efficacy and safety of adalimumab for the treatment of peripheral arthritis in spondyloarthritis patients without ankylosing spondylitis or psoriatic arthritis." Arthritis Rheum 64: S235. | Title |
| 1582 | Paranos, P., et al. (2022). "Assessing clinical potential of old antibiotics against severe infections by multi-drug-resistant gram-negative bacteria using in silico modelling." 15(12): 1501. | Title |
| 1583 | Paredes, I., et al. (2012). "Albatross-II - A locally injected bradykinin antagonist for treatment of osteoarthritis. An ongoing, sequential ascending-dose study, to evaluate the safety, tolerability, PK and PD of intra-articular doses of fasitibant inpatients with symptomatic osteoarthritis of the knee." Basic & clinical pharmacology & toxicology 111: 12. | Title |
| 1584 | Park, W., et al. (2014). "Clinical response of disease activity, disability and mobility indices in relation to anti-drug antibody in the planetas." Ann Rheum Dis 73. | Title |
| 1585 | Parvez, A., et al. (2023). "PD-1 and PD-L1: architects of immune symphony and immunotherapy breakthroughs in cancer treatment." 14: 1296341. | Title |
| 1586 | Pasha, M., et al. (2023). "A systematic review on the clinical pharmacokinetics of vildagliptin in healthy and disease populations." Expert Opin Drug Metab Toxicol 19(12): 991-1003. | Title |
| 1587 | Pasquini, M. C., et al. (2020). "Worldwide Network for Blood and Marrow Transplantation (WBMT) recommendations for establishing a hematopoietic cell transplantation program (Part I): Minimum requirements and beyond." Hematol Oncol Stem Cell Ther 13(3): 131-142. | Title |
| 1588 | Pasquini, M. C., et al. (2019). "Worldwide Network for Blood and Marrow Transplantation Recommendations for Establishing a Hematopoietic Cell Transplantation Program, Part I: Minimum Requirements and Beyond." Biology of Blood and Marrow Transplantation 25(12): 2322-2329. | Title |
| 1589 | Pastrana, T., et al. (2024). "Use of Essential Medicines for Pain Relief and Palliative Care: A Global Consensus Process." Journal of Pain and Symptom Management. | Title |
| 1590 | Patel, V. and C. Andrade (2003). "Pharmacological treatment of severe psychiatric disorders in the developing world : lessons from India." CNS Drugs 17(15): 1071-1080. | Title |

| | | |
|---|---|---|
| 1591 | Pathan, A. J. N. J. (2020). "Current off-label Pharmacotherapeutics in the management of novel coronavirus disease (COVID-19)."  5(1): 108-112. | Title |
| 1592 | Patnaik, S., et al. (2021). "Recent update of toxicity aspects of nanoparticulate systems for drug delivery." European Journal of Pharmaceutics and Biopharmaceutics 161: 100-119. | Title |
| 1593 | Patra, S., et al. (2022). "Epigenetic dysregulation in autophagy signaling as a driver of viral manifested oral carcinogenesis." Biochimica et Biophysica Acta (BBA) - Molecular Basis of Disease 1868(11): 166517. | Title |
| 1594 | Patsalos, P. N. and J. W. Sander (1994). "Newer antiepileptic drugs. Towards an improved risk-benefit ratio." Drug Saf 11(1): 37-67. | Title |
| 1595 | Paul, F., et al. (2022). "Meteoroid: a randomised, double-blind, placebocontrolled, multicentre phase 3 study of satralizumab in patients with myelin oligodendrocyte glycoprotein antibody-associated disease." Multiple sclerosis journal 28(3): 835. | Title |
| 1596 | Paul-Emile, K. J. G. W. L. R. (2011). "The regulation of race in science."  80: 1115. | Title |
| 1597 | Pavelka, K., et al. (2012). "Maintenance of response with conventional- or reduced-dose etanercept therapy or biologic free after induction of response with etanercept-methotrexate therapy in patients with moderately active rheumatoid arthritis participating in the PRESERVE study in Europe, Latin America, and Asia." Int J Rheum Dis 15: 54-55. | Title |
| 1598 | Pearson, H. A. (1996). "Pharmacologic Manipulation of Fetal Hemoglobin Levels in Sickle Cell Diseases and Thalassemia: Promise and Reality." Advances in Pediatrics 43(1): 309-334. | Title |
| 1599 | Pechlivani, N., et al. (2024). "Use of Affimer Technology for Inhibition of $\alpha$2-antiplasmin and Enhancement of Fibrinolysis." Blood Advances. | Title |
| 1600 | Peden, E. (2010). "PAD incidence in HD patients." Journal of vascular access 11(3): 225-226. | Title |
| 1601 | Peitz, G. J. and D. J. J. A. Murry (2023). "The influence of extracorporeal membrane oxygenation on antibiotic pharmacokinetics."  12(3): 500. | Title |
| 1602 | Pellicano, R. and L. Guerra (2012). "[Skin ulcer pain]." Minerva Med 103(6): 525-531. | Title |
| 1603 | Peng, L., et al. (2013). "RNA-sequencing quantification of hepatic ontogeny of phase-I enzymes in mice." Drug Metab Dispos 41(12): 2175-2186. | Title |
| 1604 | Pereira, G. R. C., et al. (2023). "In Silico Analyses of a Promising Drug Candidate for the Treatment of Amyotrophic Lateral Sclerosis Targeting Superoxide Dismutase I Protein." Pharmaceutics 15(4). | Title |

| | | |
|---|---|---|
| 1605 | Pérez de la Ossa, N., et al. (2022). "Effect of Direct Transportation to Thrombectomy-Capable Center vs Local Stroke Center on Neurological Outcomes in Patients With Suspected Large-Vessel Occlusion Stroke in Nonurban Areas: the RACECAT Randomized Clinical Trial." JAMA 327(18): 1782-1794. | Title |
| 1606 | Perez, F., et al. (2016). "Treatment options for infections caused by carbapenem-resistant Enterobacteriaceae: can we apply “precision medicine” to antimicrobial chemotherapy?" 17(6): 761-781. | Title |
| 1607 | Pérez-López, A., et al. (2021). "Fecal Carriage and Molecular Characterization of Carbapenemase-Producing Enterobacterales in the Pediatric Population in Qatar." Microbiology Spectrum 9(3). | Title |
| 1608 | Perry, B. N., et al. (2013). "Mirror therapy as a phantom limb pain treatment for upper-extremity amputees." J Neurol 260: S19. | Title |
| 1609 | Perry, C. M. and A. Markham (1998). "Sumatriptan. An updated review of its use in migraine." Drugs 55(6): 889-922. | Title |
| 1610 | Peter, T., et al. (2016). "Stimulation of eryptosis by caspofungin." 39(3): 939-949. | Title |
| 1611 | Peterlin, B. L. and A. M. Rapoport (2007). "Clinical pharmacology of the serotonin receptor agonist, zolmitriptan." Expert Opin Drug Metab Toxicol 3(6): 899-911. | Title |
| 1612 | Petersen, T. (2011). "The mckenzie method compared with manipulation in low back pain patients presenting with centralization or peripheralization: a randomized controlled trial." Physiotherapy (united kingdom) 97: eS990. | Title |
| 1613 | Petrek, J. and V. Seltzer (2003). "Breast Cancer in Pregnant and Postpartum Women." Journal of Obstetrics and Gynaecology Canada 25(11): 944-950. | Title |
| 1614 | Pham, H. N., et al. (2024). "Antibiotic Resistance, Biofilm Formation, and Persistent Phenotype of Klebsiella pneumoniae in a Vietnamese Tertiary Hospital: A Focus on Amikacin." 30(5): 203-209. | Title |
| 1615 | Phillips, A. L., et al. (2014). "Cost-effectiveness of subcutaneous interferon beta-1a in a sub-population of multiple sclerosis patients (kurtzke expanded disability status scale [EDSS]: > 3.5-5.0)." Value in health 17(7): A398. | Title |
| 1616 | Phillips, A. L., et al. (2015). "Cost-effectiveness of 44 mcg subcutaneous interferon beta-1a (scIFNβ1a) and 30 mcg intramuscular interferon beta-1a (imIFNβ1a) using clinical endpoints of disease activity." Multiple sclerosis (Houndmills, Basingstoke, England) 23(11): 421. | Title |
| 1617 | Phillips, J. T., et al. (2016). "Efficacy of delayed-release dimethyl fumarate in newly diagnosed patients with relapsing- remitting multiple sclerosis using a composite measure of disability-Integrated analysis of the phase 3 define and confirm studies." Multiple sclerosis (Houndmills, Basingstoke, England) 22(6): NP3-NP4. | Title |

| | | |
|---|---|---|
| 1618 | Picozzi, A. and N. M. Ross (1989). "A survey of dentists' drug prescribing practices." Am J Dent 2(6): 338-340. | Title |
| 1619 | Pien, F. D., et al. (2001). "MYCOBACTERIAL INFECTIONS IN PATIENTS WITH CHRONIC RENAL DISEASE." Infectious Disease Clinics of North America 15(3): 851-876. | Title |
| 1620 | Pinhas-Hamiel, O. and P. Zeitler (2005). "Advances in Epidemiology and Treatment of Type 2 Diabetes in Children." Advances in Pediatrics 52: 223-259. | Title |
| 1621 | Pirinen, S. and H. J. I. D. Alakomi (2001). "Antimicrobial agents: bacterial/fungal." 32(1178ą1187): 35. | Title |
| 1622 | Pleuvry, B. J. (2005). "Pharmacogenetics: familial variation in drug response." Anaesthesia & Intensive Care Medicine 6(7): 243-244. | Title |
| 1623 | Poehlau, D., et al. (2015). "Hippotherapy improves symptoms of multiple sclerosis-results of a randomised controlled multicentre study." Multiple sclerosis (Houndmills, Basingstoke, England) 23(11): 795-796. | Title |
| 1624 | Poewe, W. H., et al. (1986). "Treatment of motor fluctuations in Parkinson's disease with an oral sustained-release preparation of L-dopa: clinical and pharmacokinetic observations." Clin Neuropharmacol 9(5): 430-439. | Title |
| 1625 | Poggi, C. and C. J. A. Dani (2023). "New antimicrobials for the treatment of neonatal sepsis caused by multi-drug-resistant bacteria: a systematic review." 12(6): 956. | Title |
| 1626 | Pohl, O., et al. (2018). "Pharmacokinetics, safety and tolerability of OBE022, a selective prostaglandin F2$\alpha$ receptor antagonist tocolytic: A first-in-human trial in healthy postmenopausal women." Br J Clin Pharmacol 84(8): 1839-1855. | Title |
| 1627 | Polachek, A., et al. (2019). "Late onset psoriatic arthritis in a longitudinal cohort: Disease presentation, activity over time and prognosis." Seminars in Arthritis and Rheumatism 48(5): 834-839. | Title |
| 1628 | Politi, C., et al. (2018). "Genetics and Treatment Response in Parkinson's Disease: An Update on Pharmacogenetic Studies." Neuromolecular Med 20(1): 1-17. | Title |
| 1629 | Pollock, B. G. and B. H. Mulsant (1995). "Antipsychotics in older patients. A safety perspective." Drugs Aging 6(4): 312-323. | Title |
| 1630 | Polman, C. H., et al. (2012). "Trial design and baseline data of the INFORMS (fingolimod in patients with primary progressive multiple sclerosis) study." Multiple sclerosis (Houndmills, Basingstoke, England) 18(4): 437-438. | Title |
| 1631 | Pondugula, S. R., et al. (2022). "Cardioprotective effects of Oroxylum indicum extract against doxorubicin and cyclophosphamide-induced cardiotoxicity." 1-11. | Title |
| 1632 | Poply, K. (2019). "Sphenopalatine ganglion stimulation therapy: procedural challenges." Neuromodulation 22(7): e329. | Title |

| | | |
|---|---|---|
| 1633 | Pradana, A., et al. (2024). "Influence of Solute Carrier Family 22 Member 1 (SLC22A1) Gene Polymorphism on Metformin Pharmacokinetics and HbA1c Levels: A Systematic Review." 20(4): 62-74. | Title |
| 1634 | Prajapati, J., et al. (2021). "Endophytic fungi: A treasure trove of novel anticancer compounds." Current Research in Pharmacology and Drug Discovery 2: 100050. | Title |
| 1635 | Prakash, A. and B. Jarvis (1999). "Leflunomide: a review of its use in active rheumatoid arthritis." Drugs 58(6): 1137-1164. | Title |
| 1636 | Prashantha, C. N., et al. (2021). "Molecular screening of antimalarial, antiviral, anti-inflammatory and HIV protease inhibitors against spike glycoprotein of coronavirus." Journal of Molecular Graphics and Modelling 102: 107769. | Title |
| 1637 | Predel, H. G., et al. (2013). "Efficacy and safety of diclofenac diethylamine 1.16% gel in the treatment of acute neck pain: a randomized, double-blind, placebo-controlled study." Annals of the rheumatic disease 71. | Title |
| 1638 | Price Evans, D. A. (1989). "N-acetyltransferase." Pharmacology & Therapeutics 42(2): 157-234. | Title |
| 1639 | Prince, M. J. (2009). "Treatment for patients and caregivers in the developing world." Alzheimer's & dementia 5(4): 117-118. | Title |
| 1640 | Privitera, A. P., et al. (2015). "OCDB: a database collecting genes, miRNAs and drugs for obsessive-compulsive disorder." Database (Oxford) 2015: bav069. | Title |
| 1641 | Priya, P. S., et al. (2023). "Graphene oxide decorated daidzein as an oral drug to ameliorate the oxidative stress and glucocorticoid-induced osteoporosis in vivo zebrafish model." Journal of Drug Delivery Science and Technology 81: 104278. | Title |
| 1642 | Priyadarshini, N., et al. (2022). "Antimycobacterial effect of plant derived phthalate against Mycobacterium tuberculosis H37Ra." Physiological and Molecular Plant Pathology 117: 101761. | Title |
| 1643 | Przybysz, A., et al. (2015). "Scientific abstract: chronic pain measurement invariance of the oswestry disability index (ODI) across different pain populations." Regional anesthesia and pain medicine 40(5). | Title |
| 1644 | Pugazhendhi, A., et al. (2025). "Deciphering the importance of nanoencapsulation to improve the availability of bioactive molecules in food sources to the human body." Food Chemistry 464: 141762. | Title |
| 1645 | Pulakuntla, S., et al. (2024). "Molecular docking and dynamics analysis to reveal the therapeutic potential of Dostarlimab against novel immune targets in liver cancer." 61(11): 740-755. | Title |

| | | |
|---|---|---|
| 1646 | Puolakka, K., et al. (2006). "Monetary value of lost productivity over a five year follow up in early rheumatoid arthritis estimated on the basis of official register data on patients' sickness absence and gross income: experience from the FIN-RACo trial." Ann Rheum Dis 65(7): 899-904. | Title |
| 1647 | Purohit, P. J., et al. (2019). "Antimicrobial disposition during pediatric continuous renal replacement therapy using an ex vivo model." 47(9): e767-e773. | Title |
| 1648 | Puspitasari, A. D., et al. (2021). "Community knowledge and attitude in recognizing asthma symptoms and using medication for asthma attacks: a cross-sectional study." 32(4): 467-472. | Title |
| 1649 | Puspitasari, H. P., et al. (2021). "Challenges in the provision of natural medicines by community pharmacists in East Java Province, Indonesia." 32(4): 875-880. | Title |
| 1650 | Qadir, A., et al. (2020). "Nanostructured lipidic carriers for dual drug delivery in the management of psoriasis: Systematic optimization, dermatokinetic and preclinical evaluation." Journal of Drug Delivery Science and Technology 57: 101775. | Title |
| 1651 | Qasim, M., et al. (2023). "Molecular mechanism of Ferula asafoetida for the treatment of asthma: Network pharmacology and molecular docking approach." Saudi Journal of Biological Sciences 30(2): 103527. | Title |
| 1652 | Qayoom, H., et al. (2023). "Decoding the molecular mechanism of stypoldione against breast cancer through network pharmacology and experimental validation." Saudi Journal of Biological Sciences 30(12): 103848. | Title |
| 1653 | Qin, N., et al. (2024). "Enhanced long-acting simvastatin delivery via effervescent powder-carrying hollow microneedles and nanocrystal-loaded microneedles." Int J Pharm 665: 124691. | Title |
| 1654 | Raal, F., et al. (2012). "Low-density lipoprotein cholesterol–lowering effects of AMG 145, a monoclonal antibody to proprotein convertase subtilisin/kexin type 9 serine protease in patients with heterozygous familial hypercholesterolemia: the reduction of LDL-C with PCSK9 inhibition in heterozygous familial hypercholesterolemia disorder (RUTHERFORD) randomized trial." 126(20): 2408-2417. | Title |
| 1655 | Raal, F. J., et al. (2019). "Treatment effect of alirocumab according to age group, smoking status, and hypertension: Pooled analysis from 10 randomized ODYSSEY studies." Journal of Clinical Lipidology 13(5): 735-743. | Title |
| 1656 | Rachmawati, H., et al. (2004). "Pharmacokinetic and biodistribution profile of recombinant human IL-10 following intravenous administration in rats with extensive liver fibrosis." 21: 39. | Animal |

| | | |
|---|---|---|
| 1657 | Radcliff, K., et al. (2011). "Predictors of reoperation in lumbar stenosis and degenerative spondylolisthesis surgery: a subgroup analysis of the sport study." Spine journal 11(10): 68S. | Title |
| 1658 | Radcliff, K., et al. (2011). "Does opioid pain medication use affect the outcome of patients with lumbar disc herniation? A subgroup analysis of the SPORT study." Spine journal 11(10): 86S. | Title |
| 1659 | Radcliff, K., et al. (2011). "Does iliac crest autograft affect the outcome of fusion in the setting of degenerative spondylolisthesis? A subgroup analysis of the SPORT study." Spine journal 11(10): 60S. | Title |
| 1660 | Radcliff, K., et al. (2011). "Does weakness affect the outcome of patients treated for lumbar disc herniation? A subgroup analysis of the SPORT study." Spine journal 11(10): 85S-86S. | Title |
| 1661 | Rademaker, M., et al. (2013). "Isotretinoin 5 mg/day for persistent adult acne." Australasian journal of dermatology 54: 20. | Title |
| 1662 | Radominski, S. C., et al. (2017). "Tofacitinib, an oral Janus kinase inhibitor, for the treatment of Latin American patients with rheumatoid arthritis: pooled efficacy and safety analyses of Phase 3 and long-term extension studies." Reumatologia clinica 13(4): 201-209. | Title |
| 1663 | Radovanovic, D., et al. (2016). "Formoterol fumarate + glycopyrrolate for the treatment of chronic obstructive pulmonary disease." Expert Rev Respir Med 10(10): 1045-1055. | Title |
| 1664 | Radue, E., et al. (2013). "Brain atrophy and disease-free status over four years: analyses of the FREEDOMS core and extension trial data." Multiple sclerosis (Houndmills, Basingstoke, England) 19(11): 481. | Title |
| 1665 | Radwan, M. A., et al. (2011). "Monitoring metformin in cardiac patients exposed to contrast media using ultra-high-performance liquid chromatography tandem mass-spectrometry." Ther Drug Monit 33(6): 742-749. | Title |
| 1666 | Raef, H., et al. (2016). "Sorafenib efficacy and safety in advanced well differentiated thyroid cancer (WDTC), early local experience in Saudi Arabia." Thyroid 26: A154. | Title |
| 1667 | Rafeeq, M. M., et al. (2018). "Effect of genetic polymorphisms in SREBF-SCAP pathway on therapeutic response to rosuvastatin in Saudi metabolic syndrome patients." Pharmacogenomics 19(3): 185-196. | Abstract |
| 1668 | Rafighi, D., et al. (2022). "Review on Pathogenicity and Drug-Resistance Mechanisms at Acinetobacter Baumannii." 17(3): 65-75. | Title |
| 1669 | Raftopoulos, R. E., et al. (2012). "A phase II double-blind, randomised, placebo-controlled trial of neuroprotection with phenytoin in acute optic neuritis." Multiple sclerosis (Houndmills, Basingstoke, England) 18(4): 442-443. | Title |
| 1670 | Ragab, A. R., et al. (2013). "Cyclosporine toxicity and toxicokinetics profiles in renal transplant recipients." 3(1): 2161-0495.1000154. | Title |

| | | |
|---|---|---|
| 1671 | Raganato, A. "WSP Chief Executive." | Title |
| 1672 | Ragavendran, C., et al. (2024). "Green-route synthesis of ZnO nanoparticles via Solanum surattense leaf extract: Characterization, biomedical applications and their ecotoxicity assessment of zebrafish embryo model." South African Journal of Botany 167: 643-662. | Title |
| 1673 | Raghu, M. S., et al. (2023). "Design, synthesis and molecular docking studies of 5,6-difluoro-1H-benzo[d]imidazole derivatives as effective binders to GABAA receptor with potent anticonvulsant activity." Journal of Molecular Structure 1285: 135502. | Title |
| 1674 | Rahman, P., et al. (2013). "Ustekinumab improves physical function, quality of life and work productivity of patients with active psoriatic arthritis who were naive to MTX, despite MTX therapy or previously treated with anti-TNF: results from psummit I and psummit II." Ann Rheum Dis 72. | Title |
| 1675 | Raja, H. N., et al. (2023). "Sodium alginate-based smart gastro-retentive drug delivery system of revaprazan loaded SLNs; Formulation and characterization." International Journal of Biological Macromolecules 253: 127402. | Title |
| 1676 | Raja, K. (2022). Biomedical Text Mining, Springer. | Title |
| 1677 | Rajab, A. A. H., et al. (2024). "In vitro and in vivo assessment of the competence of a novel lytic phage vB_EcoS_UTEC10 targeting multidrug resistant Escherichia coli with a robust biofilm eradication activity." Microbial Pathogenesis 197: 107058. | Title |
| 1678 | Rajaratnam, K., et al. (2013). "Platelet rich plasma following arthroscopic repair of rotator cuff tears: a double blind randomized controlled trial." Arthroscopy - journal of arthroscopic and related surgery 29(10): e46. | Title |
| 1679 | Rajimon, K. J., et al. (2025). "Comprehensive assessment of schiff base derived from 4-Chloroaniline and 2-Formylphenol: Molecular architecture, experimental with computational bioactivity profiling, emphasizing anticancer efficacy against pulmonary and mammary carcinoma cell models." Journal of Molecular Structure 1322: 140590. | Title |
| 1680 | Rajpurohit, M., et al. (2024). "Fabrication and characterisation of nabumetone transferosomal gel for effective topical delivery." Journal of Molecular Structure 1312: 138430. | Title |
| 1681 | Ram Kumar, A., et al. (2025). "Exploring the potential of diosgenin as a promising antitumor agent through comprehensive spectroscopic characterization, solvent–solute interactions, topological properties, Hirshfeld surface, and molecular docking interactions with 2NZT and 2I1V proteins." Spectrochimica Acta Part A: Molecular and Biomolecular Spectroscopy 327: 125349. | Title |
| 1682 | Ramadon, D., et al. (2020). "Development, Evaluation, and Pharmacokinetic Assessment of Polymeric Microarray Patches for Transdermal Delivery of Vancomycin Hydrochloride." Molecular Pharmaceutics 17(9): 3353-3368. | Title |

| | | |
|---|---|---|
| 1683 | Ramalho, J., et al. (2015). "High Signal Intensity in Globus Pallidus and Dentate Nucleus on Unenhanced T1-weighted MR Images: Evaluation of Two Linear Gadolinium-based Contrast Agents." Radiology 276(3): 836-844. | Title |
| 1684 | Ramatillah, D. L., et al. (2023). "Factors Contributing to Chronic Kidney Disease following COVID-19 Diagnosis in Pre-Vaccinated Hospitalized Patients."  11(2): 433. | Title |
| 1685 | Ramsay, R. E., et al. (2007). "Special issues in the management of young children, older adults, and the developmentally disabled." J Child Neurol 22(5 Suppl): 53s-60s. | Title |
| 1686 | Ramzan, M., et al. (2024). "Hansen solubility parameters and quality-by-design oriented optimized cationic nanoemulsion for transdermal drug delivery of tolterodine tartrate." Int J Pharm 664: 124611. | Title |
| 1687 | Rana, M. A., et al. (2014). "Intra-pleural colistin methanesulfonate therapy for pleural infection caused by carbapenem-resistant acinetobacter baumannii: a successful case report."  6(3). | Title |
| 1688 | Rangaraju, S., et al. (2016). "Differences in quality of life across modified rankin scale categories in IMS-3." Stroke 47(no pagination). | |
| 1689 | Rao, H., et al. (2023). "Phytochemical screening, biological evaluation, and molecular docking studies of aerial parts of Trigonella hamosa (branched Fenugreek)." Arabian Journal of Chemistry 16(7): 104795. | Title |
| 1690 | Rapoport, A. M., et al. (2010). "Innovative delivery systems for migraine: the clinical utility of a transdermal patch for the acute treatment of migraine." CNS Drugs 24(11): 929-940. | Title |
| 1691 | Raseta, N., et al. (2018). "Eating habits and standard body parameters among students at university of banja luka."  19(1): 41-49. | Title |
| 1692 | Rasool, M., et al. (2022). "New challenges in the use of nanomedicine in cancer therapy."  13(1): 759-773. | Title |
| 1693 | Rasool, M. F., et al. (2021). "Development and evaluation of physiologically based pharmacokinetic drug-disease models for predicting captopril pharmacokinetics in chronic diseases." Sci Rep 11(1): 8589. | Title |
| 1694 | Rasool, M. F., et al. (2020). "Investigating the Role of Altered Systemic Albumin Concentration on the Disposition of Theophylline in Adult and Pediatric Patients with Asthma by Using the Physiologically Based Pharmacokinetic Approach." Drug Metab Dispos 48(7): 570-579. | Title |
| 1695 | Rastegar-Kashkouli, A., et al. (2024). "Systematic Review and Meta-Analysis on the Prevalence and Antibiotic Susceptibility Pattern in Pseudomonas aeruginosa Isolated from Cystic Fibrosis Patients." | Title |
| 1696 | Rauch, S., et al. (2024). "Highly specific SARS-CoV-2 main protease (Mpro) mutations against the clinical antiviral ensitrelvir selected in a safe, VSV-based system." Antiviral Research 231: 105969. | Title |

| | | |
|---|---|---|
| 1697 | Read, N. W., et al. (1980). "Chronic diarrhea of unknown origin." Gastroenterology 78(2): 264-271. | Title |
| 1698 | Reddy, S., et al. (2010). "Clinical utility of desvenlafaxine 50 mg/d for treating MDD: a review of two randomized placebo-controlled trials for the practicing physician." Curr Med Res Opin 26(1): 139-150. | Title |
| 1699 | Reder, A., et al. (2016). "Long-term effect of fingolimod on disability: a categorical trend analysis over 8 years." Eur J Neurol 23: 69-70. | Title |
| 1700 | Reder, A. T., et al. (2012). "Survival outcomes and cause of death from the 21-year long-term follow-up study." Multiple sclerosis (Houndmills, Basingstoke, England) 18(12): 1865. | Title |
| 1701 | Redfern, J., et al. (2009). "Stop stroke: cluster randomised controlled trial of a patient/carer and general practitioner intervention to improve risk factor management after Stroke." Cerebrovascular diseases (Basel, Switzerland) 27: 66. | Title |
| 1702 | Rehman, A., et al. (2024). "Enhancing antiviral therapies through nonlinear control of Hepatitis C virus dynamics." Biomedical Signal Processing and Control 97: 106727. | Title |
| 1703 | Rehman, Z., et al. (2022). "Combination of levetiracetam with sodium selenite prevents pentylenetetrazole-induced kindling and behavioral comorbidities in rats." Saudi Pharmaceutical Journal 30(5): 494-507. | Title |
| 1704 | Repovic, P., et al. (2015). "Disease activity during the first year predicts clinical long-term outcomes: impact of fingolimod in the transforms phase III trial and its extension." Neurology 84. | Title |
| 1705 | Research, I. A. T. C. G. o. t. E. O. f. and T. o. C. J. A. o. I. Medicine (1993). "Efficacy and toxicity of single daily doses of amikacin and ceftriaxone versus multiple daily doses of amikacin and ceftazidime for infection in patients with cancer and granulocytopenia." 119(7_Part_1): 584-593. | Title |
| 1706 | Resnick, I. B., et al. (2005). "Nonmyeloablative stem cell transplantation and cell therapy for malignant and non-malignant diseases." Transplant Immunology 14(3): 207-219. | Title |
| 1707 | Reuter, B., et al. (2016). "Access, timing and intensity of very early stroke rehabilitation-insights from the Baden-Württemberg stroke registry." Cerebrovascular diseases (Basel, Switzerland) 41: 308. | Title |
| 1708 | Rex, D. K. (2009). "Endoscopist-directed propofol." Techniques in Gastrointestinal Endoscopy 11(4): 177-180. | Title |
| 1709 | Reyes, S. T., et al. (2021). "Effects of the sigma-1 receptor agonist blarcamesine in a murine model of fragile X syndrome: neurobehavioral phenotypes and receptor occupancy." Sci Rep 11(1): 17150. | Title |
| 1710 | Reynolds, J., et al. (2007). "Abatacept: a novel treatment for moderate-to-severe rheumatoid arthritis." Pharmacotherapy 27(12): 1693-1701. | Title |

| | | |
|---|---|---|
| 1711 | Rezaei, N., et al. (2023). Tuberculosis: integrated studies for a complex disease 2050. Tuberculosis: Integrated Studies for a Complex Disease, Springer: 1063-1098. | Title |
| 1712 | Rezki, N., et al. (2020). "Novel scaffold hopping of potent benzothiazole and isatin analogues linked to 1,2,3-triazole fragment that mimic quinazoline epidermal growth factor receptor inhibitors: Synthesis, antitumor and mechanistic analyses." Bioorganic Chemistry 103: 104133. | Title |
| 1713 | Riaz, B. and Y.-C. J. P. M. C. Chen (2023). "Role of Genetic Polymorphisms in the Efficacy and Adverse Effects of Metformin in the Treatment of Type 2 Diabetes Mellitus." 3(1): 63-77. | Title |
| 1714 | Ricci, G., et al. (2019). "Statins: Pharmacokinetics, Pharmacodynamics and Cost-Effectiveness Analysis." Curr Vasc Pharmacol 17(3): 213-221. | Title |
| 1715 | Righi, E., et al. (2017). "Global prevalence of carbapenem resistance in neutropenic patients and association with mortality and carbapenem use: systematic review and meta-analysis." 72(3): 668-677. | Title |
| 1716 | Rinne, U. K. (1987). "Madopar HBS in the long-term treatment of parkinsonian patients with fluctuations in disability." Eur Neurol 27 Suppl 1: 120-125. | Title |
| 1717 | RISK-ADAPTED, C. R. F. B. and P. S.-T. P. J. N.-O. HR (2014). "reserved. For permissions, please e-mail: journals. permissions@ oup. com." 16: i10-i13. | Title |
| 1718 | Rissardo, J. P., et al. (2022). "Pimavanserin and Parkinson's Disease Psychosis: A Narrative Review." Brain Sci 12(10). | Title |
| 1719 | Ritter, R. (2010). Interpretation of human biomonitoring data with population pharmacokinetic modeling for persistent chemicals, ETH Zurich. | Title |
| 1720 | Rizvi, S. A. H., et al. (2013). "Pediatric Kidney Transplantation in the Developing World: Challenges and Solutions." American Journal of Transplantation 13(9): 2441-2449. | Title |
| 1721 | Roberts, J. A., et al. (2019). "Defining optimal dosing of ciprofloxacin in patients with septic shock." J Antimicrob Chemother 74(6): 1662-1669. | Title |
| 1722 | Roberts, J. A., et al. (2019). "Defining optimal dosing of ciprofloxacin in patients with septic shock." 74(6): 1662-1669. | Title |
| 1723 | Roberts, J. K., et al. (2014). "Dance (darbe administration in newborns undergoing cooling for encephalopathy): safety and pharmacokinetic trial." Journal of investigative medicine 62(1): 210. | Title |
| 1724 | Robinson, I. C. (2005). "African American Pharmacists in Health Care." | Title |

| | | |
|---|---|---|
| 1725 | Rodrigues, S. O., et al. (2024). "Macrolide resistance outcomes after the Covid-19 pandemic: A one health approach investigation." Biomedicine & Pharmacotherapy 180: 117437. | Title |
| 1726 | Rodríguez-Gascón, A., et al. (2021). "The role of PK/PD analysis in the development and evaluation of antimicrobials." 13(6): 833. | Title |
| 1727 | Romagnuolo, J., et al. (2013). "Can patient and pain characteristics predict manometric sphincter of Oddi dysfunction (SOD) in patients with clinically suspected SOD enrolled in the episod trial?" Gastrointestinal endoscopy 77(5): AB288-AB289. | Title |
| 1728 | Rondot, P., et al. (1987). "Clinical trial of Madopar HBS in parkinsonian patients with fluctuating drug response after long-term levodopa therapy." Eur Neurol 27 Suppl 1: 114-119. | Title |
| 1729 | Roperch, J. P., et al. (2016). "Promoter hypermethylation of HS3ST2, SEPTIN9 and SLIT2 combined with FGFR3 mutations as a sensitive/specific urinary assay for diagnosis and surveillance in patients with low or high-risk non-muscle-invasive bladder cancer." BMC Cancer 16(1): 704. | Title |
| 1730 | Rose, F. C. (1985). "Problems in the management of stroke." Ann Acad Med Singap 14(1): 12-15. | Title |
| 1731 | Rosenberg, L. J., et al. (2020). "Results From a Survey of American Geriatrics Society Members' Views on Physician-Assisted Suicide." Journal of the American Geriatrics Society 68(1): 23-30. | Title |
| 1732 | Roshdy, A., et al. (2022). "Intensivists' perceptions and attitudes towards infectious diseases management in the ICU: An international survey." Medicina Intensiva 46(10): 549-558. | Title |
| 1733 | Rostom, S. A. F., et al. (2009). "Design and synthesis of some thiazolyl and thiadiazolyl derivatives of antipyrine as potential non-acidic anti-inflammatory, analgesic and antimicrobial agents." Bioorganic & Medicinal Chemistry 17(2): 882-895. | Title |
| 1734 | Rother, M., et al. (2007). "Efficacy and safety of epicutaneous ketoprofen in Transfersome (IDEA-033) versus oral celecoxib and placebo in osteoarthritis of the knee: multicentre randomised controlled trial." Ann Rheum Dis 66(9): 1178-1183. | Title |
| 1735 | Rottier, W. C., et al. (2012). "Effects of confounders and intermediates on the association of bacteraemia caused by extended-spectrum β-lactamase-producing Enterobacteriaceae and patient outcome: a meta-analysis." 67(6): 1311-1320. | Title |
| 1736 | Roushan, M. H., et al. (2010). "Comparison of the efficacy of gentamicin for 5 days plus doxycycline for 8 weeks versus streptomycin for 2 weeks plus doxycycline for 45 days in the treatment of human brucellosis: a randomized clinical trial." 65(5): 1028-1035. | Title |
| 1737 | Rubab, S., et al. (2021). "Enhanced neuroprotective and antidepressant activity of curcumin-loaded nanostructured lipid carriers in lipopolysaccharide-induced depression and anxiety rat model." Int J Pharm 603: 120670. | Title |

| | | |
|---|---|---|
| 1738 | Rubab, S., et al. (2020). "Determination of the GC–MS analysis of seed oil and assessment of pharmacokinetics of leaf extract of Camellia sinensis L." Journal of King Saud University - Science 32(7): 3138-3144. | Title |
| 1739 | Rudick, R., et al. (2013). "Six-year natalizumab safety and efficacy data from the STRATA study." Multiple sclerosis (Houndmills, Basingstoke, England) 19(11): 250-251. | Title |
| 1740 | Rudick, R. A., et al. (2012). "Correlation between EDSS and MSFC in the FREEDOMS study." Multiple sclerosis (Houndmills, Basingstoke, England) 18(4): 416. | Title |
| 1741 | Ruijter, B., et al. (2015). "Telstar-treatment of electroencephalographic status epilepticus after cardiopulmonary resuscitation." International journal of stroke 10: 426. | Title |
| 1742 | Ruiz-Giménez, J., et al. (2010). "Antiepileptic treatment in patients with epilepsy and other comorbidities." Seizure 19(7): 375-382. | Title |
| 1743 | Rutecki, P. A. and B. E. Gidal (2002). "Antiepileptic drug treatment in the developmentally disabled: treatment considerations with the newer antiepileptic drugs." Epilepsy Behav 3(6s1): 24-31. | Title |
| 1744 | Ryan, P., et al. (2024). "Hepatitis B virus (HBV) viremia despite tenofovir disoproxil fumarate-containing antiretroviral therapy in persons with HBV/HIV coinfection." Journal of Clinical Virology 175: 105733. | Title |
| 1745 | S, M., et al. (2024). "Structural, Spectral, Pharmacokinetics Analysis (in-Silico), Drug-Likeness, NCI Analysis (ELF, LOL, IRI & DORI) & Molecular Docking Computations of 2-Hydroxy 2-Phenyl Acetophenone a DFT Approaches." Polycyclic Aromatic Compounds. | Title |
| 1746 | Saadullah, M., et al. (2024). "Biological and in silico investigation of isolated novel bioactive compound from Conocarpus lancifolius." Journal of King Saud University - Science 36(4): 103121. | Title |
| 1747 | Saati, A. A., et al. (2021). "A Saudi Arabian public health perspective of tuberculosis." 18(19): 10042. | Title |
| 1748 | Saber, S., et al. (2022). "Nifuroxazide-loaded cubosomes exhibit an advancement in pulmonary delivery and attenuate bleomycin-induced lung fibrosis by regulating the STAT3 and NF-κB signaling: A new challenge for unmet therapeutic needs." Biomedicine & Pharmacotherapy 148: 112731. | Title |
| 1749 | Sackley, C., et al. (2005). "The reliability of balance, mobility and self-care measures in a population of adults with a learning disability known to a physiotherapy service." Clinical rehabilitation 19(2): 216-223. | Title |
| 1750 | Sadat, S. M., et al. (2021). "Nano-delivery of a novel inhibitor of polynucleotide kinase/phosphatase (PNKP) for targeted sensitization of colorectal cancer to radiation-induced DNA damage." 11: 772920. | Title |

| | | |
|---|---|---|
| 1751 | Saeza, I., et al. (2012). "Organization of TeleHomeCare at the Home Setting: different approaches to specific phenotypes." European geriatric medicine 3: S128-S129. | Title |
| 1752 | Safdar, A., et al. (2021). "Progress in oncology biosimilars till 2020: Scrutinizing comparative studies of biosimilar monoclonal antibodies." 27(5): 1195-1204. | Title |
| 1753 | Sagah, G. A. and M. M. J. T. R. Fayed (2023). "Body mass index as a predictor of the outcomes of acute clozapine toxicity: a cross-sectional study." 12(4): 599-607. | Title |
| 1754 | Saghari, M., et al. (1988). "Published in Clinical Pharmacology and Therapeutics, 2022 Mar 1, doi: 10.1002/cpt. 2539." 26(3): 166. | Title |
| 1755 | Said, K. B., et al. (2023). "Profiles of independent-comorbidity groups in senior COVID-19 patients reveal low fatality associated with standard care and low-dose hydroxychloroquine over antivirals." 1215-1229. | Title |
| 1756 | Sakkat, A., et al. (2021). "Temperature control in critically ill patients with fever: A meta-analysis of randomized controlled trials." Journal of Critical Care 61: 89-95. | Title |
| 1757 | Salamun, et al. (2021). "Larvicidal toxicity and parasporal inclusion of native Bacillus thuringiensis BK5. 2 against Aedes aegypti." 32(4): 379-384. | Title |
| 1758 | Saleem, T., et al. (2024). "Exploring the pharmacokinetics of second-generation cephalosporin, cefaclor: a systematic review in healthy and diseased populations." Xenobiotica 54(4): 171-181. | Title |
| 1759 | Saleh Alanazi, S. H., et al. (2024). "Calotropis procera: A double edged sword against glioblastoma, inhibiting glioblastoma cell line growth by targeting histone deacetylases (HDAC) and angiogenesis." Heliyon 10(2): e24406. | Title |
| 1760 | Saleh Faisal, M., et al. (2024). "Distribution pattern of UGT1A6 and UGT2B7 gene polymorphism and its impact on the pharmacokinetics of valproic acid and carbamazepine: Prospective genetic association study conducted in Pakistani patients with epilepsy." Gene 892: 147886. | Title |
| 1761 | Saleh, R. M., et al. (2015). "Cardioprotective role of tadalafil against cisplatin-induced cardiovascular damage in rats." European Journal of Pharmacology 765: 574-581. | Title |
| 1762 | Salem, M., et al. (2022). "Secondary antiviral metabolites from fungi with special reference to coronaviruses." Biocell 46(8): 1979-1988. | Title |
| 1763 | Salmanton-García, J., et al. (2023). "The current state of laboratory mycology in Asia/Pacific: A survey from the European Confederation of Medical Mycology (ECMM) and International Society for Human and Animal Mycology (ISHAM)." International Journal of Antimicrobial Agents 61(3): 106718. | Title |

| | | |
|---|---|---|
| 1764 | Sambunaris, A., et al. (2012). "A phase iii, double-blind, placebo-controlled flexible-dose study of levomilnacipran sr in patients with major depressive disorder." Neuropsychopharmacology 38: S322-S323. | Title |
| 1765 | Samie, M., et al. (2023). "Drug/bioactive eluting chitosan composite foams for osteochondral tissue engineering." International Journal of Biological Macromolecules 229: 561-574. | Title |
| 1766 | Samodelov, S. L., et al. (2024). "L-carnitine co-administration prevents colistin-induced mitochondrial permeability transition and reduces the risk of acute kidney injury in mice." 14(1): 16444. | Title |
| 1767 | Samtani, M., et al. (2013). "Disease progression modeling of bapineuzumab utilizing Alzheimer's disability assessment for dementia scores." Alzheimer's & dementia 9(4): P284. | Title |
| 1768 | Sanai, F., et al. (2018). "A randomized open-label trial of paritaprevir plus ritonavir plus ombitasvir in the treatment of HCV genotype 4-infected patients with stage 4-5 chronic kidney disease." Hepatology (Baltimore, Md.) 68: 399A-400A. | Title |
| 1769 | Sanai, F. M., et al. (2013). "Safety and efficacy of peginterferon-α2a plus ribavirin treatment in renal transplant recipients with chronic hepatitis C." J Hepatol 58(6): 1096-1103. | Title |
| 1770 | Sánchez-Martín, A., et al. (2016). "Gene–gene interactions between DRD3, MRP4 and CYP2B6 polymorphisms and its influence on the pharmacokinetic parameters of efavirenz in HIV infected patients." Drug Metabolism and Pharmacokinetics 31(5): 349-355. | Title |
| 1771 | Sanga, P., et al. (2016). "Efficacy, Safety, and Tolerability of Fulranumab as an Adjunctive Therapy in Patients With Inadequately Controlled, Moderate-to-Severe Chronic Low Back Pain: a Randomized, Double-blind, Placebo-controlled, Dose-ranging, Dose-loading Phase II Study." Clin Ther 38(6): 1435-1450. | Title |
| 1772 | Santiano, R. A. S., et al. (2019). "Shoulder positioning in acute stroke: effect on spasticity and range of motion-a pilot study." Clinical neurology 59: S425. | Title |
| 1773 | Sapkota, B., et al. (2021). "Dosage individualization proposed for anti-gout medications among the patients with gout." 16(9): e0257082. | Title |
| 1774 | Sarges, R. (1981). 6 Hypoglycaemic Drugs. Progress in Medicinal Chemistry. G. P. Ellis and G. B. West, Elsevier. 18: 191-223. | Title |
| 1775 | Sarisozen, C., et al. (2016). "Nanomedicine based curcumin and doxorubicin combination treatment of glioblastoma with scFv-targeted micelles: In vitro evaluation on 2D and 3D tumor models." European Journal of Pharmaceutics and Biopharmaceutics 108: 54-67. | Title |
| 1776 | Sasso, R., et al. (2015). "Efficacy of a novel synthetic small peptide in anterior cervical arthrodesis: a randomized, controlled, multicenter study with 24-month follow-up." Spine journal 15(10 SUPPL. 1): S243-S244. | Title |

| | | |
|---|---|---|
| 1777 | Sathianarayanan, S., et al. (2022). "A new approach against Helicobacter pylori using plants and its constituents: A review study." Microbial Pathogenesis 168: 105594. | Title |
| 1778 | Saviano, A., et al. (2022). "Anti-inflammatory and immunomodulatory activity of Mangifera indica L. reveals the modulation of COX-2/mPGES-1 axis and Th17/Treg ratio." Pharmacol Res 182: 106283. | Title |
| 1779 | Saviano, A., et al. (2024). "A reverse translational approach reveals the protective roles of Mangifera indica in inflammatory bowel disease." Journal of Autoimmunity 144: 103181. | Title |
| 1780 | Savic, R. M., et al. (2018). "Intermittent preventive treatment for malaria in pregnancy: optimization of target concentrations of dihydroartemisinin-piperaquine." 67(7): 1079-1088. | Title |
| 1781 | Saw, M. M., et al. (2015). "The effects of a six-week physiotherapist-led exercise and education intervention in patients with osteoarthritis, awaiting an arthroplasty in South Africa." Physiotherapy (united kingdom) 101: eS1343. | Title |
| 1782 | Sawy, A. M., et al. (2021). "Insights of doxorubicin loaded graphene quantum dots: Synthesis, DFT drug interactions, and cytotoxicity." Materials Science and Engineering: C 122: 111921. | Title |
| 1783 | Saxonhouse, S. J. and A. B. Curtis (2003). "Risks and benefits of rate control versus maintenance of sinus rhythm." Am J Cardiol 91(6a): 27d-32d. | Title |
| 1784 | Schachter, E. N. (2006). "Cilomilast." Drugs Today (Barc) 42(4): 237-247. | Title |
| 1785 | Schaeuble, B., et al. (2009). "Efficacy of prolonged-release methylphenidate in a randomized controlled trial in adults with ADHD: secondary endpoints." European neuropsychopharmacology 19: S679-S680. | Title |
| 1786 | Scharff, F. B., et al. (2020). "The PTSD help app in a Danish PTSD population: research protocol of a randomized controlled feasibility trial." Pilot and feasibility studies 6(1): 92. | Title |
| 1787 | Scheltens, P., et al. (2012). "Bapineuzumab IV phase 3 results." Journal of nutrition, health & aging 16(9): 797. | Title |
| 1788 | Schiff, M., et al. (2014). "Clinical responses by baseline RA disease duration in the ample (abatacept versus adalimumab comparison in biologic-naive RA patients with background methotrexate) trial: 2-year results." Ann Rheum Dis 73. | Title |
| 1789 | Schmaranzer, F., et al. (2017). "How Does the dGEMRIC Index Change After Surgical Treatment for FAI? A Prospective Controlled Study: preliminary Results." Clinical orthopaedics and related research 475(4): 1080-1099. | Title |

| | | |
|---|---|---|
| 1790 | Schmidt, C. O., et al. (2010). "Assessing a risk tailored intervention to prevent disabling low back pain--protocol of a cluster randomized controlled trial." BMC musculoskeletal disorders 11: 5. | Title |
| 1791 | Schnake, K., et al. (2013). "Randomised clinical and radiological trial comparing PEEK with titanium-coated PEEK-cages for PLIF surgery." European spine journal 22(11): 2594. | Title |
| 1792 | Scholl-Bürgi, S., et al. (2008). "Amino acid cerebrospinal fluid/plasma ratios in children: influence of age, gender, and antiepileptic medication." Pediatrics 121(4): e920-926. | Title |
| 1793 | Scholz, J., et al. (2004). "Increased systemic levels of norsalsolinol derivatives are induced by levodopa treatment and do not represent biological markers of Parkinson's disease." J Neurol Neurosurg Psychiatry 75(4): 634-636. | Title |
| 1794 | Schoretsanitis, G., et al. (2018). "TDM in psychiatry and neurology: A comprehensive summary of the consensus guidelines for therapeutic drug monitoring in neuropsychopharmacology, update 2017; a tool for clinicians<sup/>." World J Biol Psychiatry 19(3): 162-174. | Title |
| 1795 | Schreiner, A., et al. (2017). "Switching from oral atypical antipsychotic monotherapy to paliperidone palmitate once-monthly in non-acute patients with schizophrenia: a prospective, open-label, interventional study." Psychopharmacology (Berl) 234(1): 3-13. | Title |
| 1796 | Schroth, R. J., et al. (2021). "Dental outcomes for children receiving asfotase alfa for hypophosphatasia." Bone 152: 116089. | Title |
| 1797 | Schulten, H. J. (2018). "Pleiotropic Effects of Metformin on Cancer." Int J Mol Sci 19(10). | Title |
| 1798 | Schultz, T. J., et al. (2021). "Home infusions of natalizumab for people with multiple sclerosis: a pilot randomised crossover trial." Annals of clinical and translational neurology 8(8): 1610-1621. | Title |
| 1799 | Schwab-Stone, M., et al. (2001). "Cultural Considerations in The Treatment Of Children and Adolescents: Operationalizing the Importance of Culture in Treatment." Child and Adolescent Psychiatric Clinics of North America 10(4): 729-743. | Title |
| 1800 | Schwartzbach, C., et al. (2014). "The effects of GSK239512 on lesion remyelination in a relapsing remitting MS population: design of a phase 2a imaging study." Multiple sclerosis (Houndmills, Basingstoke, England) 20(1): 385. | Title |
| 1801 | Scott, E., et al. (2014). "Reductions in depression, anxiety and pain catastrophizing predict fewer pain disability days and lower pain intensity among primary care patients." Journal of pain 15(4): S12. | Title |
| 1802 | Scott, L. J. and D. P. Figgitt (2004). "Mitoxantrone: a review of its use in multiple sclerosis." CNS Drugs 18(6): 379-396. | Title |
| 1803 | Segal, J. L. and S. R. Brunnemann (1989). "Clinical pharmacokinetics in patients with spinal cord injuries." Clin Pharmacokinet 17(2): 109-129. | Title |

| | | |
|---|---|---|
| 1804 | Sehra, G. e., et al. (2025). "Elucidating the resistance mechanisms and binding pattern of novel Oxa-48-like carbapenemases covalent inhibitors: A hybrid experimental and in silico approach." Journal of Molecular Structure 1321: 140073. | Title |
| 1805 | Seleim, S. M., et al. (2023). "Biofilm formation by acinetobacter species isolated from intensive care units: Unveiling the impact on antibiotic resistance." 32(4): 33-43. | Title |
| 1806 | Selmaj, K., et al. (2012). "Safety and tolerability of BG-12 in patients with relapsing-remitting multiple sclerosis: an integrated analysis of the placebo-controlled studies." Multiple sclerosis (Houndmills, Basingstoke, England) 18(4): 200-201. | Title |
| 1807 | Serag, A., et al. (2024). "Synchronous spectrofluorimetry and chemometric modeling: A synergistic approach for analyzing simeprevir and daclatasvir, with application to pharmacokinetics evaluation." Spectrochimica Acta Part A: Molecular and Biomolecular Spectroscopy 315: 124245. | Title |
| 1808 | Serseg, T., et al. (2022). "Discovery of inhibitors against SARS-CoV-2 associated fungal coinfections via virtual screening, ADMET evaluation, PASS, molecular docking, dynamics and pharmacophore studies." 29(1): 337-350. | Title |
| 1809 | Severs, D., et al. (2015). "Intravenous solutions in the care of patients with volume depletion and electrolyte abnormalities." Am J Kidney Dis 66(1): 147-153. | Title |
| 1810 | Sfikas, N., et al. (2013). "Effect of fingolimod in patients with no disability as measured by edss at baseline: post-Hoc analyses of freedoms I and II." Neurology 80(1). | Title |
| 1811 | Sgarbura, O., et al. (2019). "Oxaliplatin use in pressurized intraperitoneal aerosol chemotherapy (PIPAC) is safe and effective: A multicenter study." European Journal of Surgical Oncology 45(12): 2386-2391. | Title |
| 1812 | Shafi, H., et al. (2023). "Super disintegrating oromucosal nanofiber patch of zolmitriptan for rapid delivery and efficient brain targeting." Chemical Engineering Journal 463: 142481. | Title |
| 1813 | Shafiq, S., et al. (2024). "A novel approach to insulin delivery via oral route: Milk fat globule membrane derived liposomes as a delivery vehicle." Saudi Journal of Biological Sciences 31(3): 103945. | Title |
| 1814 | Shah, R., et al. (2021). "Formulation development and characterization of lumefantrine nanosuspension for enhanced antimalarial activity." 32(7): 833-857. | Title |
| 1815 | Shah, Z. A., et al. (2022). "In silico view of MTA1 biochemical signatures in breast malignancy for improvement in immunosurveillance." 34(3): 101843. | Title |

| | | |
|---|---|---|
| 1816 | Shahien, R. and K. Beiruti (2012). "Preventive agents for migraine: focus on the antiepileptic drugs." J Cent Nerv Syst Dis 4: 37-49. | Title |
| 1817 | Shahzad, N., et al. (2024). "Therapeutic strategy of biological macromolecules based natural bioactive compounds of diabetes mellitus and future perspectives: A systematic review." Heliyon 10(2): e24207. | Title |
| 1818 | Shahzad Qamar, A., et al. (2022). "A review on the clinical pharmacokinetics of hydralazine." Expert Opin Drug Metab Toxicol 18(10): 707-714. | Title |
| 1819 | Shaik, N. A., et al. (2019). "Molecular designing, virtual screening and docking study of novel curcumin analogue as mutation (S769L and K846R) selective inhibitor for EGFR." Saudi Journal of Biological Sciences 26(3): 439-448. | Title |
| 1820 | Shakeel, E., et al. (2018). "Decoding the antineoplastic efficacy of Aplysin targeting Bcl-2: A de novo perspective." Comput Biol Chem 77: 390-401. | Title |
| 1821 | Shakeel, K., et al. (2022). "B-artemether and lumefantrine dual drug loaded lipid nanoparticles: Physicochemical characterization, pharmacokinetic evaluation and biodistribution study." 10(3): 210-219. | Title |
| 1822 | Shaldam, M. A., et al. (2024). "Novel sulfonamide-tethered Schiff bases as anti-proliferative agents with VEGFR-2 inhibitory activity: Synthesis, biological assessment, and molecular dynamic simulations." Journal of Molecular Structure 1309: 138148. | Title |
| 1823 | Shama, A. A. A., et al. (2023). "Effect of dexmedetomidine, dexamethasone, and ondansetron on postoperative nausea and vomiting in children undergoing dental rehabilitation: A randomized controlled trial." 26(1): 1. | Title |
| 1824 | Sharif, A. F., et al. (2024). "Assessment of co-ingestion effects on poisoning patterns, drug-drug interactions, and adverse outcomes in acute toxic exposure." Toxicology Reports 13: 101705. | Title |
| 1825 | Sharif, A. F., et al. (2023). "Development and validation of a risk prediction nomogram for disposition of acute clozapine intoxicated patients to intensive care unit." 42: 09603271231186154. | Title |
| 1826 | Sharifipour, E., et al. (2014). "Free communications 9: large clinical trials 2 citalopram effect on ischemic stroke functional outcome (coiso): randomized clinical trials." International journal of stroke 9: 39. | Title |
| 1827 | Sharma, A., et al. (2020). "Severe acute respiratory syndrome coronavirus-2 (SARS-CoV-2): a global pandemic and treatment strategies." International Journal of Antimicrobial Agents 56(2): 106054. | Title |
| 1828 | Sharma, A., et al. (2024). "Mitochondrial signaling pathways and their role in cancer drug resistance." Cellular Signalling 122: 111329. | Title |

| | | |
|---|---|---|
| 1829 | Sharma, S., et al. (2021). "Nanotechnology Driven Approaches for the Management of Parkinson's Disease: Current Status and Future Perspectives." Curr Drug Metab 22(4): 287-298. | Title |
| 1830 | Sharmin, E., et al. (2017). "Linseed polyol-assisted, microwave-induced synthesis of nano CuO embedded in polyol-polyester matrix: antifungal behavior and coating properties." Progress in Organic Coatings 105: 200-211. | Title |
| 1831 | Shaukat, A., et al. (2023). "Mechanism of the antidiabetic action of Nigella sativa and Thymoquinone: a review." Front Nutr 10: 1126272. | Title |
| 1832 | Shaw, J. R., et al. (2020). "Predictors of preprocedural direct oral anticoagulant levels in patients having an elective surgery or procedure." Blood Advances 4(15): 3520-3527. | Title |
| 1833 | Shen, X., et al. (2020). "Pharmacokinetic study of eight bioactive components following oral administration of Zhiqiao Gancao decoction and observation of its clinical efficacy." Biomed Chromatogr 34(2): e4706. | Title |
| 1834 | Shenoy, N. (2023). "Pharmacogenetics of Efavirenz and Neuropsychiatric Side Effects in Asian Populations." | Title |
| 1835 | Sherazi, A. W., et al. (2024). "A Systematic Critical Review of Clinical Pharmacokinetics of Torasemide." Ther Drug Monit 46(3): 309-320. | Title |
| 1836 | Sheridan, S. J. J. o. P. P. (2015). "A review of a self-diagnosed diabetic case study." 7(3): 132-136. | Title |
| 1837 | Sheriffdeen, M. M., et al. (2019). "Caffeine/Angelica dahurica and caffeine/Salvia miltiorrhiza metabolic inhibition in humans: In vitro and in vivo studies." Complementary Therapies in Medicine 46: 87-94. | Title |
| 1838 | Sherwin, C. M., et al. (2023). "OPEN ACCESS EDITED BY." 196. | Title |
| 1839 | Shiammala, P. N., et al. (2023). "Exploring the artificial intelligence and machine learning models in the context of drug design difficulties and future potential for the pharmaceutical sectors." Methods 219: 82-94. | Title |
| 1840 | Shibl, A. M. (1992). "The in vitro activity of aztreonam against local clinical isolates in Saudi Arabia." Current Therapeutic Research 52(1): 106-112. | Title |
| 1841 | Shibuya, M., et al. (1992). "Effect of AT877 on cerebral vasospasm after aneurysmal subarachnoid hemorrhage. Results of a prospective placebo-controlled double-blind trial." J Neurosurg 76(4): 571-577. | Title |
| 1842 | Shiekmydeen, J., et al. (2023). "A Randomized, Two-Treatments, Two-Periods, Crossover, Open label, Laboratory-Blind, Single Dose Bioequivalence Study between Vildagliptin/Metformin 50 mg/1000 mg Film Coated Tablets (Sensityn®) and Galvusmet®50 mg/1000 mg Film Coated Tablets in healthy adults under fed conditions." European pharmaceutical journal. | Title |

| | | |
|---|---|---|
| 1843 | Shiekmydeen, J., et al. (2024). "Optimization of wet granulation process for manufacturing Rivaroxban generic immediate-release tablets using PBPK modeling and simulations." 12(2): 77. | Title |
| 1844 | Shields, A., et al. (2012). "Disability status, not income, is associated with greater anxiety and distress in stem cell transplant patients." Psycho-oncology 21: 106. | Title |
| 1845 | Shields, R. K., et al. (2024). "A multicenter, observational study to compare the effectiveness of Ceftazidime-Avibactam versus Ceftolozane-Tazobactam for multidrug-resistant Pseudomonas aeruginosa infections in the United States (CACTUS)." | Title |
| 1846 | Shields, R. K., et al. (2017). "Defining the incidence and risk factors of colistin-induced acute kidney injury by KDIGO criteria." 12(3): e0173286. | Title |
| 1847 | Shilbayeh, S. (2014). "The impact of genetic polymorphisms on time required to attain the target tacrolimus levels and subsequent pharmacodynamic outcomes in pediatric kidney transplant patients." Saudi J Kidney Dis Transpl 25(2): 266-277. | Title |
| 1848 | Shilbayeh, S., et al. (2013). "The impact of CYP3A5 and MDR1 polymorphisms on tacrolimus dosage requirements and trough concentrations in pediatric renal transplant recipients." Saudi J Kidney Dis Transpl 24(6): 1125-1136. | Title |
| 1849 | Shilbayeh, S. A. R., et al. (2024). "The Frequency of CYP2D6 and CYP3A4/5 Genotypes and The Impact of Their Allele Translation and Phenoconversion-Predicted Enzyme Activity on Risperidone Pharmacokinetics in Saudi Children with Autism." Biochem Genet 62(4): 2907-2932. | Title |
| 1850 | Shilbayeh, S. A. R. and S. A. E. R. J. C. P. Ismail (2020). "Patient experience with an educational mobile health application: A pilot study on usability and feasibility in a Saudi population." 7(1): 1843883. | Title |
| 1851 | Shilbayeh, S. J. S. J. o. K. D. and Transplantation (2014). "The impact of genetic polymorphisms on time required to attain the target tacrolimus levels and subsequent pharmacodynamic outcomes in pediatric kidney transplant patients." 25(2): 266-277. | Title |
| 1852 | Shimizu, T., et al. (2003). "Bioinformatics Research on Inter-racial Difference in Drug Metabolism I. Analysis on Frequencies of Mutant Alleles and Poor Metabolizers on CYP2D6 and CYP2C19." Drug Metabolism and Pharmacokinetics 18(1): 48-70. | Title |
| 1853 | Shirley, M. (2017). "Daclizumab: A Review in Relapsing Multiple Sclerosis." Drugs 77(4): 447-458. | Title |
| 1854 | Shirzad-Yazdi, N., et al. (2024). "Drug-related problems among pediatric intensive care units: prevalence, risk factors, and clinical pharmacists' interventions." 24(1): 714. | Title |

| | | |
|---|---|---|
| 1855 | Shishkova, V. and A. Remennik (2015). "Association between BDNF-196 g>a and BDNF-270 C>T polymorphisms, BDNF concentration, and telmisartan treatment in rehabilitation period outcome after ischemic stroke." Journal of hypertension 33: e439. | Title |
| 1856 | Shishkova, V., et al. (2015). "Association between BDNF-196 G>A and BDNF-270 C>T polymorphisms, BDNF concentration, and cerebrolysin treatment outcome after ischemic stroke." International journal of stroke 10: 170. | Title |
| 1857 | Shneker, B. F. and N. B. Fountain (2003). "Epilepsy." Dis Mon 49(7): 426-478. | Title |
| 1858 | Shukkoor, M. S. A., et al. (2022). A Text Mining Protocol for Extracting Drug–Drug Interaction and Adverse Drug Reactions Specific to Patient Population, Pharmacokinetics, Pharmacodynamics, and Disease. Biomedical Text Mining, Springer: 259-282. | Title |
| 1859 | Sianoya, A. C., et al. (2024). "Targeting the Filipino gut microbiota in the management of hypertension." Egypt Heart J 76(1): 7. | Title |
| 1860 | Siddiqi, A., et al. (2009). "Therapeutic drug monitoring of amikacin in preterm and term infants." 50(5): 486. | Title |
| 1861 | Sidhom, P. A., et al. (2023). "Mechanistic Insight of Synthesized 1,4-Dihydropyridines as an Antidiabetic Sword against Reactive Oxygen Species." Journal of Medicinal Chemistry 66(1): 991-1010. | Title |
| 1862 | Siefker-Radtke, A. O., et al. (2022). "Efficacy and safety of erdafitinib in patients with locally advanced or metastatic urothelial carcinoma: long-term follow-up of a phase 2 study." The Lancet Oncology 23(2): 248-258. | Title |
| 1863 | Siemonsen, S., et al. (2015). "Resistance training increases cortical thickness in RRMS-results of a pilot RCT." Multiple sclerosis (Houndmills, Basingstoke, England) 23(11): 504. | Title |
| 1864 | Sigurgeirsson, B., et al. (2014). "Effect of secukinumab on psoriasis symptoms and physical functioning compared with placebo and etanercept in subjects with moderate-to-severe plaque psoriasis and concomitant psoriatic arthritis: a subanalysis from the phase 3 fixture study." Scandinavian journal of rheumatology 43: 64-65. | Title |
| 1865 | Sii-Felice, K., et al. (2018). "Hemoglobin disorders: lentiviral gene therapy in the starting blocks to enter clinical practice." Experimental Hematology 64: 12-32. | Title |
| 1866 | Sikka, S., et al. (2021). "Diosgenin attenuates tumor growth and metastasis in transgenic prostate cancer mouse model by negatively regulating both NF-κB/STAT3 signaling cascades." European Journal of Pharmacology 906: 174274. | Title |
| 1867 | Silberstein, S. (2017). "AVP-825: a novel intranasal delivery system for low-dose sumatriptan powder in the treatment of acute migraine." Expert Rev Clin Pharmacol 10(8): 821-832. | Title |
| 1868 | Silberstein, S. D. (2017). "Topiramate in Migraine Prevention: a 2016 Perspective." Headache 57(1): 165-178. | Title |

| | | |
|---|---|---|
| 1869 | Silva, R. R., et al. (2013). "Extended-release dexmethylphenidate 30 mg/d versus 20 mg/d: duration of attention, behavior, and performance benefits in children with attention-deficit/hyperactivity disorder." Clin Neuropharmacol 36(4): 117-121. | Title |
| 1870 | Simpson, D. M., et al. (2014). "Dalfampridine in patients with chronic post-ischemic stroke deficits: results from a phase 2 study." Stroke 45. | Title |
| 1871 | Simuni, T., et al. (2014). "STEADY-PD III. A phase 3 study of isradipine as a disease modifying agent in in patients with early Parkinson's disease." Movement disorders 29: S265. | Title |
| 1872 | Simuni, T., et al. (2015). "STEADY-PD III. A phase 3 study of isradipine as a disease modifying agent in patients with early Parkinson's disease. Study design and status update." Movement disorders 30: S124. | Title |
| 1873 | Simuni, T., et al. (2018). "A Phase 2a study of nilotinib in patients with advanced and early Parkinson's disease. Study design." Neurology 90(15). | Title |
| 1874 | Simuni, T., et al. (2019). "NILO-PD: a phase 2a study of nilotinib in patients with advanced Parkinson's disease: study design and status update." Movement disorders 34: S5-S6. | Title |
| 1875 | Sindhu, R. K., et al. (2021). "Impacting the Remedial Potential of Nano Delivery-Based Flavonoids for Breast Cancer Treatment." Molecules 26(17). | Title |
| 1876 | Singer, B., et al. (2014). "Patient-reported outcomes after therapy switch to fingolimod: post-HOC subgroup analysis of the epoc study." Neurology 82(10). | Title |
| 1877 | Singh, G., et al. (2014). "Combination treatment with glucosamine-chondroitin sulfate reduces pain, disability and nsaid consumption in patients with chronic low back pain: final results from a large, community-based, pilot, open prospective interventional study." Ann Rheum Dis 73. | Title |
| 1878 | Singh, I., et al. (2020). "Curative Efficacy of Fosfomycin Tromethamine Versus Ciprofloxacin in the Initial Therapy of Uncomplicated UTI—a Prospective Open-Label Randomised Controlled Clinical Study." 82(3): 331-337. | Title |
| 1879 | Singh, S., et al. (2019). Implementation and impact of an antimicrobial stewardship program at a tertiary care center in South India. Open forum infectious diseases, Oxford University Press US. | Title |
| 1880 | Singh, S., et al. (2024). "Integrating Nanotechnological Advancements of Disease-Modifying Anti-Rheumatic Drugs into Rheumatoid Arthritis Management." 17(2): 248. | Title |

| | | |
|---|---|---|
| 1881 | Singh, S. K., et al. (2021). "A brief overview about the use of different bioactive liposome-based drug delivery systems in Peritoneal Dialysis and some other diseases." 2(2): 022006. | Title |
| 1882 | Sjövall, F., et al. (2018). "Maximally effective dosing regimens of meropenem in patients with septic shock." J Antimicrob Chemother 73(1): 191-198. | Title |
| 1883 | Skoro-Sajer, N. and I. Lang (2008). "The role of treprostinil in the management of pulmonary hypertension." Am J Cardiovasc Drugs 8(4): 213-217. | Title |
| 1884 | Slark, J. S. (2012). "A randomized controlled trial (RCT) to investigate risk awareness in secondary stroke prevention." Cerebrovascular diseases (Basel, Switzerland) 33: 817-818. | Title |
| 1885 | Slotkin, J. R., et al. (2016). "Sustained Local Release of Methylprednisolone From a Thiol-Acrylate Poly(Ethylene Glycol) Hydrogel for Treating Chronic Compressive Radicular Pain." Spine (Phila Pa 1976) 41(8): E441-448. | Title |
| 1886 | Smith, S. L., et al. (2021). "Evaluating the delivery of Problem Management plus in primary care settings in rural Rwanda: a study protocol using a pragmatic randomised hybrid type 1 effectiveness-implementation design." BMJ open 11(12): e054630. | Title |
| 1887 | Snibbe, J. C. and R. A. Gambardella (2005). "Treatment options for osteoarthritis." Orthopedics 28(2 Suppl): s215-220. | Title |
| 1888 | So, A., et al. (2011). "Rapid improvement in health-related quality of life in gouty arthritis patients treated with canakinumab (ACZ885) compared to triamcinolone acetonide." Rheumatology 50: iii85. | Title |
| 1889 | So, C. L., et al. (2015). "The effects of a community aquatic exercise programme for chinese people with knee osteoarthriris." Physiotherapy (united kingdom) 101: eS1415-eS1416. | Title |
| 1890 | Sobh, E. A., et al. (2024). "New thieno[2,3-d]pyrimidine derivatives as EGFRWT and EGFRT790M inhibitors: Design, synthesis, antiproliferative activities, docking studies, ADMET, toxicity, MD simulation studies." Journal of Heterocyclic Chemistry 61(2): 285-304. | Title |
| 1891 | Soliman, H. F. J. A. S. J. o. A. (2015). "Bispectral index-guided induction of anesthesia by ketofol infusion provides the same cardiovascular stability like that of etomidate infusion." 8(3). | Title |
| 1892 | Soliman, R. and A. J. A. o. C. A. Ragheb (2018). "Assessment of the effect of two regimens of milrinone infusion in pediatric patients undergoing Fontan procedure: a randomized study." 21(2): 134-140. | Title |
| 1893 | Soliman, R., et al. (2012). "Comparison of early and late intravenous infusion of milrinone in pediatric patients undergoing cardiac surgery." 1: 6-12. | Title |

| | | |
|---|---|---|
| 1894 | Solleti, V. S., et al. (2015). "Antimicrobial properties of liposomal azithromycin for Pseudomonas infections in cystic fibrosis patients." 70(3): 784-796. | Title |
| 1895 | Soltani Banavandi, M. J. and N. J. T. P. J. Satarzadeh (2020). "Association between VKORC1 gene polymorphism and warfarin dose requirement and frequency of VKORC1 gene polymorphism in patients from Kerman province." 20(4): 574-578. | Title |
| 1896 | Soltanimehr, E., et al. (2019). "Efficacy of diode and CO2 lasers along with calcium and fluoride-containing compounds for the remineralization of primary teeth." BMC oral health 19(1): N.PAG. | Title |
| 1897 | Soman, T., et al. (2015). "Efficacy of delayed-release dimethyl fumarate in young adults with RRMS: an integrated analysis of DEFINE and CONFIRM." Multiple sclerosis (Houndmills, Basingstoke, England) 23(11): 760-761. | Title |
| 1898 | Song, X., et al. (2021). "Competence mining of vancomycin (VAN) in the management of infections due to bacterial strains with high VAN minimum inhibitory concentrations (MICs): a novel dosing strategy based on pharmacokinetic/pharmacodynamic modeling." 12: 649757. | Title |
| 1899 | Song, Y., et al. (2023). "Update on the impacts of COVID-19 and vaccine on female reproductive system, pregnancies, and neonatal outcomes: a narrative review." 6. | Title |
| 1900 | Sorbye, L. W., et al. (2008). "Unintended weight loss in the elderly living at home: the Aged in Home Care project (AdHOC)." Journal of nutrition, health & aging 12(1): 10-16. | Title |
| 1901 | Soriano, A., et al. (2021). "Ceftazidime-avibactam for the treatment of serious gram-negative infections with limited treatment options: a systematic literature review." 10: 1989-2034. | Title |
| 1902 | Sorlí, L., et al. (2013). "Trough colistin plasma level is an independent risk factor for nephrotoxicity: a prospective observational cohort study." 13: 1-9. | Title |
| 1903 | Sormani, M. P., et al. (2011). "Advantages of early treatment: a meta-regression analysis of interferon beta-1a trials in multiple sclerosis." Multiple sclerosis (Houndmills, Basingstoke, England) 17(10): S461. | Title |
| 1904 | Sormani, M. P., et al. (2015). "Predicting treatment response to teriflunomide in the TEMSO study using the modified Rio score." Multiple sclerosis (Houndmills, Basingstoke, England) 23(11): 587-588. | Title |
| 1905 | SORRELL, T. C., et al. (1982). "Vancomycin therapy for methicillin-resistant Staphylococcus aureus." 97(3): 344-350. | Title |
| 1906 | Soul, J. S., et al. (2019). "Recommendations for the design of therapeutic trials for neonatal seizures." Pediatr Res 85(7): 943-954. | Title |

| | | |
|---|---|---|
| 1907 | Souqiyyeh, M. Z. and F. A. Shaheen (2009). "Survey of attitude of physicians on updates in the management of anemia in chronic kidney disease patients." Saudi J Kidney Dis Transpl 20(3): 410-416. | Title |
| 1908 | Spacca, G., et al. (2005). "Analgesic efficacy of a lecithin-vehiculated diclofenac epolamine gel in shoulder periarthritis and lateral epicondylitis: a placebo-controlled, multicenter, randomized, double-blind clinical trial." Drugs Exp Clin Res 31(4): 147-154. | Title |
| 1909 | Spapen, H., et al. (2019). "Treatment of ventilator-associated pneumonia with high-dose colistin under continuous veno-venous hemofiltration." 7(3): 100-105. | Title |
| 1910 | Spelman, T., et al. (2015). "Risk of early relapse following switch to oral agents for multiple sclerosis." Multiple sclerosis (Houndmills, Basingstoke, England) 23(11): 270-271. | Title |
| 1911 | Spina, E., et al. (1994). "CYP2D6-related oxidation polymorphism in Italy." Pharmacol Res 29(3): 281-289. | Title |
| 1912 | Sreeharsha, N., et al. (2024). "Ultrasonication-mediated synthesis of diblock polymer-based nanoparticles for advanced drug delivery systems: Insights and optimization." Ultrasonics Sonochemistry 111: 107137. | Title |
| 1913 | Sreeharsha, N., et al. (2025). "Formulation optimization of chitosan surface coated solid lipid nanoparticles of griseofulvin: A Box-Behnken design and in vivo pharmacokinetic study." European Journal of Pharmaceutical Sciences 204: 106951. | Title |
| 1914 | Srivastava, V., et al. (2021). "Piperidine based 1,2,3-triazolylacetamide derivatives induce cell cycle arrest and apoptotic cell death in Candida auris." Journal of Advanced Research 29: 121-135. | Title |
| 1915 | Starling, A. J., et al. (2023). "INP104: a drug evaluation of a nonoral product for the acute treatment of migraine." Pain Manag 13(5): 283-298. | Title |
| 1916 | Migraine is a very common headache disorder that often presents with pain and gastrointestinal symptoms. There are many available treatments for migraine, but some patients still need an option that works well for them, that is noninvasive, or does not need to be taken orally. Here we provide a drug evaluation of INP104, an approved acute treatment for migraine that combines a drug and a device: the medication dihydroergotamine (DHE) mesylate, which has been used for decades for treating acute symptoms of migraine, and the Precision Olfactory Delivery (POD(®)) device, which delivers DHE mesylate to the hard-to-reach upper regions of the nose. Targeting this region helps medication to be absorbed faster and more consistently. In clinical trials, INP104 demonstrated favorable drug properties, came with few adverse events, and provided fast relief from migraine symptoms. | Title |
| 1917 | Stegemann, S. (2018). "Patient centric drug product design in modern drug delivery as an opportunity to increase safety and effectiveness." Expert Opin Drug Deliv 15(6): 619-627. | Title |

| | | |
|---|---|---|
| 1918 | Steib, J. P. and M. S. Hisey (2014). "FDA IDE clinical trial results through 48 months: one-level TDR versus ACDF." European spine journal 23: S473-S474. | Title |
| 1919 | Stein, D. J., et al. (2003). "Paroxetine in the treatment of post-traumatic stress disorder: pooled analysis of placebo-controlled studies." Expert Opin Pharmacother 4(10): 1829-1838. | Title |
| 1920 | Stein, M. B., et al. (2005). "Efficacy of low and higher dose extended-release venlafaxine in generalized social anxiety disorder: a 6-month randomized controlled trial." Psychopharmacology (Berl) 177(3): 280-288. | Title |
| 1921 | Steinbach, W. J., et al. (2012). "Results from a prospective, international, epidemiologic study of invasive candidiasis in children and neonates." 31(12): 1252-1257. | Title |
| 1922 | Steiner, J. A. (1991). Antihypertensive drugs. Side Effects of Drugs Annual. M. N. G. Dukes and J. K. Aronson, Elsevier. 15: 200-211. | Title |
| 1923 | Steinhoff, B. J., et al. (2024). "Therapeutic strategies during cenobamate treatment initiation: Delphi panel recommendations." Ther Adv Neurol Disord 17: 17562864241256733. | Title |
| 1924 | Steinlein, O. K. J. N.-S. s. a. o. p. (2010). "Gene polymorphisms and their role in epilepsy treatment and prognosis." 382: 109-118. | Title |
| 1925 | Sternieri, E. and A. Ferrari (1992). "[New aspects of therapy in hemicrania]." Ann Ital Med Int 7(3 Suppl): 46s-63s. | Title |
| 1926 | Stevans, J. M., et al. (2021). "Risk Factors Associated with Transition from Acute to Chronic Low Back Pain in US Patients Seeking Primary Care." JAMA network open 4(2): e2037371. | Title |
| 1927 | Stiehm, E. R. (1988). "Human Gamma Globulins as Therapeutic Agents." Advances in Pediatrics 35(1): 1-72. | Title |
| 1928 | Stocchi, F., et al. (2018). "Pharmacokinetic drug evaluation of CVT-301 for the treatment of Parkinson's disease." Expert Opin Drug Metab Toxicol 14(12): 1189-1195. | Title |
| 1929 | Stockis, A., et al. (2014). "Brivaracetam Single and Multiple Rising Oral Dose Study in Healthy Japanese Participants: Influence of CYP2C19 Genotype." Drug Metabolism and Pharmacokinetics 29(5): 394-399. | Title |
| 1930 | Stockmann, C., et al. (2014). "Considerations in the pharmacologic treatment and prevention of neonatal sepsis." 16: 67-81. | Title |
| 1931 | Størset, E. (2012). Population pharmacokinetics of tacrolimus in kidney transplant recipients-A model for individual dosing, The University of Bergen. | Title |
| 1932 | Strand, V., et al. (2012). "Factors that impact work productivity in the preserve trial: a randomized controlled trial of combination etanercept-methotrexate therapy in patients with moderately active rheumatoid arthritis." Arthritis Rheum 64: S777. | Title |

| | | |
|---|---|---|
| 1933 | Strand, V., et al. (2013). "Systematic radiographic progression in individual joints before and after methotrexate-/+ adalimumab treatment in patients with early rheumatoid arthritis." Ann Rheum Dis 72. | Title |
| 1934 | Strauss, W. L., et al. (2002). "Fluorine magnetic resonance spectroscopy measurement of brain fluvoxamine and fluoxetine in pediatric patients treated for pervasive developmental disorders." Am J Psychiatry 159(5): 755-760. | Title |
| 1935 | Student, P. D. "2014 ACCP Virtual Poster Symposium." | Title |
| 1936 | Stukstette, M., et al. (2011). "Determinants of limitations in activities in patients with osteoarthritis of hands." Physiotherapy (united kingdom) 97: eS1184. | Title |
| 1937 | Su, C. C. and C. Remedios (2015). "Clinical and cost effectiveness of additional post-operative physiotherapy on enhancing shoulder range and functional arm use in breast cancer patients." Physiotherapy (united kingdom) 101: eS1271-eS1272. | Title |
| 1938 | Sulaiman, A. A. A., et al. (2022). "Design, Synthesis, and Preclinical Activity in Ovarian Cancer Models of New Phosphanegold(I)-N-heterocyclic Carbene Complexes." Journal of Medicinal Chemistry 65(21): 14424-14440. | Title |
| 1939 | Sulaiman, K. A., et al. (2024). "Evaluation of Apixaban standard dosing in underweight patients with non-valvular atrial fibrillation: a retrospective cohort study." Thromb J 22(1): 43. | Title |
| 1940 | Sullivan, D. A., et al. (2023). "TFOS Lifestyle: Impact of cosmetics on the ocular surface." The Ocular Surface 29: 77-130. | Title |
| 1941 | Sullivan, S., et al. (2012). "Acceptance, catastrophizing, and depressive symptoms in persons with disability-related chronic pain." Journal of pain 13(4): S96. | Title |
| 1942 | Sumrra, S. H., et al. (2022). "Metal incorporated sulfonamides as promising multidrug targets: Combined enzyme inhibitory, antimicrobial, antioxidant and theoretical exploration." Journal of Molecular Structure 1250: 131710. | Title |
| 1943 | Sun, K., et al. (2024). "A novel multivariate logistic model for predicting risk factors of failed treatment with carbapenem-resistant Acinetobacter baumannii ventilator-associated pneumonia." 12: 1385118. | Title |
| 1944 | Suppes, T., et al. (2012). "Major depressive disorder with mixed features: interim baseline characteristics of subjects enrolled in a 6 week, double-blind, placebo-controlled trial of lurasidone." Neuropsychopharmacology 38: S176-S177. | Title |
| 1945 | Suppes, T., et al. (2013). "A randomized, double-blind, placebo-controlled study of ziprasidone in bipolar disorder with co-occurring lifetime panic or generalized anxiety disorder." Bipolar disorders 15: 102. | Title |

| | | |
|---|---|---|
| 1946 | Suryani, I. R., et al. (2024). "Risk of healing impairment following tooth extraction in patients administered with antiresorptive and non-antiresorptive polypharmacy." Journal of Stomatology, Oral and Maxillofacial Surgery 125(2): 101645. | Title |
| 1947 | Swaine, J., et al. (2011). "Recruitment and consent of women with intellectual disabilities in a randomised control trial of a health promotion intervention." Journal of intellectual disability research 55(5): 474-483. | Title |
| 1948 | Swierkot, J. and J. Szechiński (2006). "Methotrexate in rheumatoid arthritis." Pharmacol Rep 58(4): 473-492. | Title |
| 1949 | Sy, S. K., et al. (2013). "A Markov chain model to evaluate the effect of CYP3A5 and ABCB1 polymorphisms on adverse events associated with tacrolimus in pediatric renal transplantation." 15: 1189-1199. | Title |
| 1950 | Taha, E. I. and F. I. J. B. J. o. P. R. Abd-Alla (2016). "Pharmacokinetics and Bioequivalence Study of Two Proton Pump Inhibitor Products." 13(6): 1-8. | Title |
| 1951 | Tahir, H., et al. (2019). "Rapid and sustained improvements in patient reported outcomes with ixekizumab in biologicnaïve and TNF-inadequate responder patients with psoriatic arthritis." Rheumatology (united kingdom) 58: iii147. | Title |
| 1952 | Taiwo, B. J., et al. (2018). "Schistosomiasis: Snail-vector control, molecular modelling and dynamic studies of bioactive N-acetylglycoside saponins from Tetrapleura tetraptera." Comput Biol Chem 77: 363-372. | Title |
| 1953 | Tajari, H., et al. (2009). "The comparison between efficacy of tamsulosin and terazosin in alphaadrenergic blockers after ESWL (a double blind clinical trial)." Urology 74(4): S340-S341. | Title |
| 1954 | Tajari, H., et al. (2009). "The comparison between efficacy of tamsulosin and terazosinin alpha-adrenergic blockers after ESWL (A double blind clinical trial)." Journal of endourology / Endourological Society 23(11): A33-A34. | Title |
| 1955 | Tajima, K. and M. Nanri (2000). "[A pharmacological profile of piracetam (Myocalm), a drug for myoclonus]." Nihon Yakurigaku Zasshi 116(4): 209-214. | Title |
| 1956 | Tajti, J., et al. (2016). "Alleviation of pain in painful diabetic neuropathy." Expert Opin Drug Metab Toxicol 12(7): 753-764. | Title |
| 1957 | Takeuchi, T., et al. (2015). "Pharmacokinetics, efficacy and safety profiles of etanercept monotherapy in Japanese patients with rheumatoid arthritis: review of seven clinical trials." Mod Rheumatol 25(2): 173-186. | Title |
| 1958 | Talasaz, A. H., et al. (2014). "The role of preoperative administration of L-carnitie in the prophylaxis of post CABG atrial fibrillation." European heart journal 35: 433. | Title |

| | | |
|---|---|---|
| 1959 | Talha Zahid, M., et al. (2023). "A physiologically based pharmacokinetic model of cefepime to predict its pharmacokinetics in healthy, pediatric and disease populations." Saudi Pharmaceutical Journal 31(8): 101675. | Title |
| 1960 | Talukder, M. E. K., et al. (2025). "Molecular docking, QSAR, and simulation analyses of EGFR-targeting phytochemicals in non-small cell lung cancer." Journal of Molecular Structure 1321: 139924. | Title |
| 1961 | Tamura, T. (2011). "[Management of myocardial damage in muscular dystrophy]." Brain Nerve 63(11): 1217-1228. | Title |
| 1962 | Tang, X., et al. (2022). "Clinical and genetic features of Chinese pediatric patients with severe congenital protein C deficiency who first presented with purpura fulminans: A case series study and literature review." Thrombosis Research 210: 70-77. | Title |
| 1963 | Tanna, V., et al. (2023). "Exploring Nose to Brain Nano Delivery for Effective Management of Migraine." Curr Drug Deliv 20(2): 144-157. | Title |
| 1964 | Tantawy, M. A., et al. (2023). "Anti-cancer activity, and molecular docking of novel hybrid heterocyclic steroids revealed promising anti-hepatocellular carcinoma agent: Implication of cyclin dependent kinase-2 pathway." Steroids 193: 109187. | Title |
| 1965 | Tarpley, J., et al. (2016). "Identifying potential sliding dichotomy cutpoints for thrombectomy trials using baseline age, deficit severity, and core lesion volume." Stroke 47(no pagination). | Title |
| 1966 | Tasneem, S., et al. (2022). "Heterocyclic moieties as HDAC inhibitors: Role in cancer therapeutics." 22(12): 1648-1706. | Title |
| 1967 | Tassaneeyakul, W., et al. (2006). "CYP2C19 Genetic Polymorphism in Thai, Burmese and Karen Populations." Drug Metabolism and Pharmacokinetics 21(4): 286-290. | Title |
| 1968 | Tawil, A., et al. (2020). "Design of a phase 2, randomized, double-blind, placebo-controlled, 24-week, parallel-group study of the efficacy and safety of losmapimod in treating subjects with facioscapulohumeral muscular dystrophy (FSHD): redux4." Neurology 94(15). | Title |
| 1969 | Tayubi, I. A. and I. H. Madar (2023). "Identification of potential inhibitor targeting KRAS mutation in Papillary Thyroid Carcinoma through molecular docking and dynamic simulation analysis." Computers in Biology and Medicine 152: 106377. | Title |
| 1970 | Tekade, R. K. (2024). Public Health and Toxicology Issues in Drug Research, Volume 2: Toxicity and Toxicodynamics, Elsevier. | Title |
| 1971 | Ten Eick, A. P., et al. (2001). "Safety of antihistamines in children." Drug Saf 24(2): 119-147. | Title |
| 1972 | Tepper, S., et al. (2014). "A randomized, double-blind, double-dummy, active comparator crossover study of breath powered™ nasal delivery of sumatriptan powder (AVP-825) in the treatment of acute migraine (the compass study)." Headache 54(8): 1434. | Title |
| 1973 | Tepper, S. J., et al. (2018). "Characterization of dizziness treatment-emergent adverse events after lasmiditan: findings of the samurai and spartan phase 3 acute migraine treatment trials." Headache 58(Supplement 2): 172-173. | Title |

| | | |
|---|---|---|
| 1974 | Tfelt-Hansen, P. (2012). "Clinical pharmacology of current and future drugs for the acute treatment of migraine: a review and an update." Curr Clin Pharmacol 7(1): 66-72. | Title |
| 1975 | Thabit, A. K., et al. (2016). "Pharmacodynamic and pharmacokinetic profiling of delafloxacin in a murine lung model against community-acquired respiratory tract pathogens." International Journal of Antimicrobial Agents 48(5): 535-541. | Title |
| 1976 | Thabit, A. K., et al. (2017). "Simplifying piperacillin/tazobactam dosing: pharmacodynamics of utilizing only 4.5 or 3.375 g doses for patients with normal and impaired renal function." 30(6): 593-599. | Title |
| 1977 | Thabit, A. K., et al. (2018). "Assessment of in vivo efficacy of eravacycline against Enterobacteriaceae exhibiting various resistance mechanisms: a dose-ranging study and pharmacokinetic/pharmacodynamic analysis." International Journal of Antimicrobial Agents 51(5): 727-732. | Title |
| 1978 | Thabit, A. K., et al. (2016). "In vitro pharmacodynamics of human simulated exposures of telavancin against methicillin-susceptible and-resistant Staphylococcus aureus with and without prior vancomycin exposure." 60(1): 222-228. | Title |
| 1979 | Thase, M. E., et al. (2014). "Efficacy and safety of vilazodone in generalized anxiety disorder: a randomized, double-blind, placebo-controlled trial." Neuropsychopharmacology 39: S344. | Title |
| 1980 | Thiboutot, D. M., et al. (2018). "Practical management of acne for clinicians: An international consensus from the Global Alliance to Improve Outcomes in Acne." Journal of the American Academy of Dermatology 78(2, Supplement 1): S1-S23.e21. | Title |
| 1981 | Thomas, L., et al. (2020). "Influence of single nucleotide polymorphisms on rifampin pharmacokinetics in tuberculosis patients." 9(6): 307. | Title |
| 1982 | Thomas, R. R., et al. (2025). "Isatin derived morpholine and piperazine derivatives as acetylcholinesterase inhibitors." Journal of Molecular Structure 1322: 140503. | Title |
| 1983 | Thomas, S. A., et al. (2010). "Description of a behaviour therapy intervention aimed at improving mood in stroke patients with aphasia." International journal of stroke 5: 12. | Title |
| 1984 | Thome, C., et al. (2014). "Endplate changes observed on CT one year following lumbar microdiscectomy: interim analysis from a multicenter, prospective, randomized clinical trial." European spine journal 23(11): 2524. | Title |
| 1985 | Thompson, D., et al. (2011). "Does targeting cognitive factors improve physiotherapy outcome in patients with chronic neck pain? A randomised controlled trial." Physiotherapy (united kingdom) 97: eS1230-eS1231. | Title |

| | | |
|---|---|---|
| 1986 | Thöne, J. and R. Gold (2013). "Review of laquinimod and its therapeutic potential in multiple sclerosis." Expert Opin Pharmacother 14(18): 2545-2552. | Title |
| 1987 | Thorpy, M. J. (2020). "Recently Approved and Upcoming Treatments for Narcolepsy." CNS Drugs 34(1): 9-27. | Title |
| 1988 | Tietjen, G., et al. (2013). "Headache prevention with cranial electrotherapy stimulation in chronic migraine. A randomized controlled trial." Cephalalgia 33(11): 963-964. | Title |
| 1989 | Tilson, H. H. (1986). "Social policy and drug safety." Clin Geriatr Med 2(1): 165-180. | Title |
| 1990 | Timperley, C. M., et al. (2019). "Advice on assistance and protection by the Scientific Advisory Board of the Organisation for the Prohibition of Chemical Weapons: Part 2. On preventing and treating health effects from acute, prolonged, and repeated nerve agent exposure, and the identification of medical countermeasures able to reduce or eliminate the longer term health effects of nerve agents." Toxicology 413: 13-23. | Title |
| 1991 | Tirri, T. and P. De Negri (2015). "Intra-articular us hip injection of platelet-rich plasma vs viscosupplementation: evaluation of long term pain relief and functional improvement in middle aged patients." Regional anesthesia and pain medicine 40(5): e83. | Title |
| 1992 | To, K., et al. (2011). "Successful combination therapy with vancomycin and arbekacin against infective endocarditis caused by MRSA."  64(6): 389-394. | Title |
| 1993 | Tobeigei, F. H., et al. (2022). "Computational high-throughput screening and in vitro approaches identify CB-006-3; A novel PI3K-BRAFV600E dual targeted inhibitor against melanoma." Oncology Research 29(5): 305-318. | Title |
| 1994 | Toogood, J. H., et al. (1989). "Bioequivalent doses of budesonide and prednisone in moderate and severe asthma." J Allergy Clin Immunol 84(5 Pt 1): 688-700. | Title |
| 1995 | Toppo, E., et al. (2017). "Effect of two andrographolide derivatives on cellular and rodent models of non-alcoholic fatty liver disease." Biomedicine & Pharmacotherapy 95: 402-411. | Title |
| 1996 | Toraih, E. A., et al. (2017). "MicroRNA-target cross-talks: Key players in glioblastoma multiforme." Tumour Biol 39(11): 1010428317726842. | Title |
| 1997 | Tortorici, M. A., et al. (2021). "Pharmacometric analysis linking immunoglobulin exposure to clinical efficacy outcomes in chronic inflammatory demyelinating polyneuropathy." CPT Pharmacometrics Syst Pharmacol 10(8): 839-850. | Title |
| 1998 | Touw, D. J., et al. (2009). "Therapeutic drug monitoring of aminoglycosides in neonates."  48: 71-88. | Title |
| 1999 | Tozan, Y., et al. (2009). "Pre-referral rectal artesunate is cost-effective for treating severe childhood malaria." American journal of tropical medicine and hygiene 81(5): 305. | Title |

| | | |
|---|---|---|
| 2000 | Traeger, A. C., et al. (2014). "Pain education to prevent chronic low back pain: a study protocol for a randomised controlled trial." BMJ open 4(6): e005505. | Title |
| 2001 | Tralongo, F., et al. (2023). "Association between clozapine plasma concentrations and treatment response: a systematic review, meta-analysis and individual participant data meta-analysis."  62(6): 807-818. | Title |
| 2002 | Triarico, S., et al. (2021). "Vincristine-Induced Peripheral Neuropathy (VIPN) in Pediatric Tumors: Mechanisms, Risk Factors, Strategies of Prevention and Treatment." Int J Mol Sci 22(8). | Title |
| 2003 | Trojano, M., et al. (2018). "Novel Assessment of Real-world Effectiveness of Ocrelizumab for Treatment of Patients with Relapsing and Primary Progressive Multiple Sclerosis: design of a Multicenter Non-interventional Study (musicale Study)." Multiple sclerosis and related disorders 26: 256-257. | Title |
| 2004 | Tsai, M.-J., et al. (2011). "Oral Apomorphine Delivery from Solid Lipid Nanoparticles with Different Monostearate Emulsifiers: Pharmacokinetic and Behavioral Evaluations." Journal of Pharmaceutical Sciences 100(2): 547-557. | Title |
| 2005 | Tse, H.-F., et al. (2013). "Stroke prevention in atrial fibrillation—An Asian stroke perspective." Heart Rhythm 10(7): 1082-1088. | Title |
| 2006 | Tulbah, A. S., et al. (2019). "Simvastatin Nanoparticles Reduce Inflammation in LPS-Stimulated Alveolar Macrophages." Journal of Pharmaceutical Sciences 108(12): 3890-3897. | Title |
| 2007 | Tumbarello, M., et al. (2018). "Optimizing therapy in carbapenem-resistant Enterobacteriaceae infections."  31(6): 566-577. | Title |
| 2008 | Tungare, K., et al. (2024). "Nanomaterial in controlling biofilms and virulence of microbial pathogens." Microbial Pathogenesis 192: 106722. | Title |
| 2009 | Turcotte, K., et al. (2014). "Ethnicity and the perception of pain in others: a focus on aboriginal Canadians." Pain research & management 19(3): e95. | Title |
| 2010 | Turkistani, A. (2019). Population Pharmacokinetic and Pharmacodynamic Modelling to Describe the Effects of APAP Overdose on Novel Biomarkers in UK Patients, The University of Liverpool (United Kingdom). | Title |
| 2011 | Turner, C. L., et al. (2009). "Simvastatin in aneurysmal subarachnoid haemorrhage (stash); a phase 111 randomised placebo controlled trial." British journal of neurosurgery 23(3): 264. | Title |
| 2012 | Turnidge, J. and P. Collignon (1999). "Resistance to fusidic acid." International Journal of Antimicrobial Agents 12: S35-S44. | Title |
| 2013 | Turtle, L. and W. Hope (2017). Flucytosine (5-Fluorocytosine; 5-FC). Kucers' The Use of Antibiotics, CRC Press: 2919-2926. | Title |

| | | |
|---|---|---|
| 2014 | Tveito, T., et al. (2012). "Illness perceptions in a population of employees with longterm sick leave for low back pain." Journal of psychosomatic research 72(6): 505. | Title |
| 2015 | Tyagi, R., et al. (2023). "In-vitro and ex-vivo antidiabetic, and antioxidant activities of Box-Behnken design optimized Solanum xanthocarpum extract loaded niosomes." Saudi Pharmaceutical Journal 31(10): 101785. | Title |
| 2016 | Uddin, M. J., et al. (2024). "Monoclonal Antibody Delivery Using 3D Printed Biobased Hollow μNe3dle Arrays for the Treatment of Osteoporosis." Molecular Pharmaceutics. | Title |
| 2017 | Uddin, M. M., et al. (2024). "Unraveling the potential effects of non-synonymous single nucleotide polymorphisms (nsSNPs) on the Protein structure and function of the human SLC30A8 gene on type 2 diabetes and colorectal cancer: An In silico approach." Heliyon 10(17): e37280. | Title |
| 2018 | Upadhyay, S., et al. (2024). "Exploring the ROS-mediated anti-cancer potential in human triple-negative breast cancer by garlic bulb extract: A source of therapeutically active compounds." Journal of Traditional and Complementary Medicine 14(6): 644-655. | Title |
| 2019 | Uppu, J. L., et al. (2024). "Apoptosis-driven synergistic anti-cancer efficacy of ethyl acetate extract of Memecylon sisparense Gamble leaves and doxorubicin in in-vitro and in-vivo models of triple-negative breast cancer." Pathology - Research and Practice 253: 155032. | Title |
| 2020 | Urtiaga, S., et al. (2018). "Tolerability and safety of dimethyl fumarate in relapsing multiple sclerosis: a prospective observational post marketing study." Multiple sclerosis journal 24(2): 524-525. | Title |
| 2021 | Vai, B., et al. (2021). "Mental disorders and risk of COVID-19-related mortality, hospitalisation, and intensive care unit admission: a systematic review and meta-analysis." The Lancet Psychiatry 8(9): 797-812. | Title |
| 2022 | Valado, T., et al. (2019). "HealthySteps: transforming the Promise of Pediatric Care." Future of children 29(1): 99-122. | Title |
| 2023 | Van Der Heijde, D., et al. (2012). "Improvement in physical function, health-related quality of life, and work productivity with adalimumab treatment in nonradiographic axial SPA: WK-52 results from ability-1." Arthritis Rheum 64: S583. | Title |
| 2024 | Van Der Heijde, D., et al. (2014). "Sustained improvement in physical function, health-related quality of life, and work productivity with adalimumab treatment in non-radiographic axial spondyloarthritis." Arthritis & rheumatology 66: S242. | Title |
| 2025 | Van Der Heijde, D. M., et al. (2013). "Improvement in physical function, health-related quality of life, and work productivity with adalimumab treatment in non-radiographic axial spondyloarthritis." Arthritis Rheum 65: S1052. | Title |
| 2026 | van Rongen, A., et al. (2016). "Morbidly obese patients exhibit increased CYP2E1-mediated oxidation of acetaminophen." 55: 833-847. | Title |

| | | |
|---|---|---|
| 2027 | van Rongen, A., et al. (2015). "Population pharmacokinetics of midazolam and its metabolites in overweight and obese adolescents." 80(5): 1185-1196. | Title |
| 2028 | van Schaik, R. H. N. (2008). "CYP450 pharmacogenetics for personalizing cancer therapy." Drug Resistance Updates 11(3): 77-98. | Title |
| 2029 | Vandewalle, B., et al. (2024). "Pharmacokinetic model-based assessment of factor IX prophylaxis treatment regimens in severe hemophilia B." 14(1): 20534. | Title |
| 2030 | Vanover, K., et al. (2015). "Clinical development of ITI-007 for the treatment of schizophrenia." Neuropsychopharmacology 40: S548-S549. | Title |
| 2031 | Vardakas, K. Z., et al. (2016). "Colistin loading dose: evaluation of the published pharmacokinetic and clinical data." 48(5): 475-484. | Title |
| 2032 | Veddeng, S., et al. (2022). "Association between statin use and physical performance in home-dwelling older patients receiving polypharmacy: cross-sectional study." BMC Geriatr 22(1): 242. | Title |
| 2033 | Vela, P., et al. (2015). "Early intervention in musculoskeletal disease can reduce temporary work disability." Ann Rheum Dis 74: 1287-1288. | Title |
| 2034 | Venegas-Rios, H., et al. (2011). "Effectiveness of spinal manipulative therapy in patients from the worker's compensation system with chronic low back pain." Physiotherapy (united kingdom) 97(var.pagings): eS1305-eS1306. | Title |
| 2035 | Venkatesh, G., et al. (2023). "Observations into the reactivity, docking, DFT, and MD simulations of fludarabine and clofarabine in various solvents." 383: 122076. | Title |
| 2036 | Venuti, F., et al. (2023). "Novel beta lactam antibiotics for the treatment of multidrug-resistant Gram-negative infections in children: a narrative review." 11(7): 1798. | Title |
| 2037 | Verma, A., et al. (2021). "A triterpene glochidon from Phyllanthus debilis: Isolation, computational studies, and antidiabetic activity evaluation." Biocatalysis and Agricultural Biotechnology 36: 102138. | Title |
| 2038 | VERMICULARIS, E. "PHLEBOTOMUS ORIENTALIS SALIVARY ANTIGENS-IDENTIFICATION, CHARACTERIZATION AND EXPRESSION." | Title |
| 2039 | Verrest, L., et al. (2021). "Geographical variability in paromomycin pharmacokinetics does not explain efficacy differences between eastern african and Indian visceral leishmaniasis patients." 60: 1463-1473. | Title |
| 2040 | Verrico, C., et al. (2019). "A phase 1 clinical trial to evaluate pharmacodynamic interactions after oral coadministration of alcohol and the highly selective glucocorticoid receptor antagonist, PT150." Neuropsychopharmacology 44: 189-190. | Title |

| | | |
|---|---|---|
| 2041 | Vesper, J., et al. (2016). "Burst or tonic stimulation? Results of a placebo controlled, double blinded, randomized study for the treatment of FBSS patients-2 year follow-up." Pain practice. Conference: 8th world congress of the world institute of pain, WIP 2016. New york city, NY united states. Conference start: 20160520. Conference end: 20160523. Conference publication: (var.pagings) 16: 74-75. | Title |
| 2042 | Vichi, S., et al. (2021). "OpenCYP: An open source database exploring human variability in activities and frequencies of polymophisms for major cytochrome P-450 isoforms across world populations." Toxicology Letters 350: 267-282. | Title |
| 2043 | Vienna, A. "Progress in pharmacokinetics and pharmacodynamics-I." | Title |
| 2044 | Viprakasit, V., et al. (2018). "An open-label, multicenter, single-arm, phase ii study assessing patient preference for the deferasirox film-coated tablet compared to the reference dispersible tablet formulation: the jupiter study." Hemasphere 2: 191. | Title |
| 2045 | Viscoli, C. M., et al. (2019). "Scoring System to Optimize Pioglitazone Therapy After Stroke Based on Fracture Risk." Stroke 50(1): 95-100. | Title |
| 2046 | Vizeshfar, F., et al. (2019). "Role-play versus lecture methods in community health volunteers." Nurse education today 79: 175-179. | Title |
| 2047 | Vlasses, P. H., et al. (2013). "Annual Report of the Accreditation Council for Pharmacy Education." American Journal of Pharmaceutical Education 77(4): 83. | Title |
| 2048 | Vlasses, P. H., et al. (2016). "Annual Report of the Accreditation Council for Pharmacy Education." American Journal of Pharmaceutical Education 80(5): 90. | Title |
| 2049 | Voldsgaard, A., et al. (2015). "Trichuris suis ova therapy in relapsing multiple sclerosis is safe but without signals of beneficial effect." Multiple sclerosis (Houndmills, Basingstoke, England) 21(13): 1723-1729. | Title |
| 2050 | Vollmer, T., et al. (2011). "A randomised, double-blind, placebo-controlled trial of duloxetine for the treatment of central neuropathic pain associated with multiple sclerosis." Multiple sclerosis (Houndmills, Basingstoke, England) 17(10): S476-S477. | Title |
| 2051 | Vouri, S. M., et al. (2021). "Changes in utilization of immediate-release, extended-release, and liquid formulation medications relative to bariatric surgery: a segmented regression analysis." Surgery for Obesity and Related Diseases 17(6): 1089-1094. | Title |
| 2052 | Vu, N.-A. T., et al. (2023). "Sharing Experiences in Pharmacy Education: A Collaboration between Chungnam National University and Hai Phong University of Medicine and Pharmacy." 1(2): 89-99. | Title |
| 2053 | Vyas, D., et al. (2013). "Tofacitinib: The First Janus Kinase (JAK) inhibitor for the treatment of rheumatoid arthritis." Ann Pharmacother 47(11): 1524-1531. | Title |

| | | |
|---|---|---|
| 2054 | Wacharachaisurapol, N., et al. (2021). "No increased acute kidney injury rate through giving an intravenous colistin loading dose in pediatric patients." 106: 91-97. | Title |
| 2055 | Wadhwa, M., et al. (2022). "WHO informal consultation on revision of guidelines on evaluation of similar biotherapeutic products, virtual meeting, 30 June – 2 July 2021." Biologicals 76: 1-9. | Title |
| 2056 | Wahab, S., et al. (2021). "Effectiveness of Azithromycin as add-on Therapy in COVID-19 Management." 21(19): 2860-2873. | Title |
| 2057 | Wahab, S., et al. (2021). "Current trends and future perspectives of nanomedicine for the management of colon cancer." European Journal of Pharmacology 910: 174464. | Title |
| 2058 | Wahid, M., et al. (2017). "Differential pharmacology and clinical utility of sonidegib in advanced basal cell carcinoma." 515-520. | Title |
| 2059 | Wakil, S. M., et al. (2015). "The Affymetrix DMET Plus platform reveals unique distribution of ADME-related variants in ethnic Arabs." Dis Markers 2015: 542543. | Title |
| 2060 | Wallace, J. and D. S. Paauw (2015). "Appropriate prescribing and important drug interactions in older adults." Med Clin North Am 99(2): 295-310. | Title |
| 2061 | Wallis, D., et al. (2013). "Mechanical back pain demonstrates better response to celecoxib than acetaminophen despite lack of mRI-defined inflammatory changes in the spine." Arthritis Rheum 65: S903-S904. | Title |
| 2062 | Walter, R. (2001). Miscellaneous antibacterial drugs. Side Effects of Drugs Annual. J. K. Aronson, Elsevier. 24: 283-313. | Title |
| 2063 | Wanas, H., et al. (2023). "The impact of CYP3A4 and CYP3A5 genetic variations on tacrolimus treatment of living-donor Egyptian kidney transplanted patients." J Clin Lab Anal 37(19-20): e24969. | Title |
| 2064 | Wang, B., et al. (2021). "Nano lipidic carriers for codelivery of sorafenib and ganoderic acid for enhanced synergistic antitumor efficacy against hepatocellular carcinoma." Saudi Pharmaceutical Journal 29(8): 843-856. | Title |
| 2065 | Wang, J. and P. R. Casner (2015). "Comparative analysis of outcomes of an ace unit in a predominantly hispanic population." Journal of the American Geriatrics Society 63: S268. | Title |
| 2066 | Wang, J.-L., et al. (2022). "Prevalence of polymyxin-induced nephrotoxicity and its predictors in critically ill adult patients: A meta-analysis." 10(31): 11466. | Title |
| 2067 | Wang, K., et al. (2021). "A meta-analysis and meta-regression on the prevalence of lipohypertrophy in diabetic patients on insulin therapy." Therapies 76(6): 617-628. | Title |

| | | |
|---|---|---|
| 2068 | Wang, T., et al. (2021). "Pharmacogenetics of tamoxifen therapy in Asian populations: from genetic polymorphism to clinical outcomes." 77: 1095-1111. | Title |
| 2069 | Wang, W. and J. J. A. M. J. Wang (2017). "Progresses in several areas make the professional development of health sciences possible." 10(2): 159. | Title |
| 2070 | Wang, Y., et al. (2021). "A novel UPLC-MS/MS assay for the measurement of linezolid and its metabolite PNU-142300 in human serum and its application to patients with renal insufficiency." 12: 641872. | Title |
| 2071 | Wang, Z., et al. (2022). "Improving the efficacy for meropenem therapy requires a high probability of target attainment in critically ill infants and children." 13: 961863. | Title |
| 2072 | Wani, T. A., et al. (2021). "Binding and drug displacement study of colchicine and bovine serum albumin in presence of azithromycin using multispectroscopic techniques and molecular dynamic simulation." Journal of Molecular Liquids 333: 115934. | Title |
| 2073 | Warsame, M., et al. (2017). "Efficacy of artesunate+ sulphadoxine/pyrimethamine and artemether+ lumefantrine and dhfr and dhps mutations in Somalia: evidence for updating the malaria treatment policy." 22(4): 415-422. | Title |
| 2074 | Watanabe, Y. (2007). "[Molecular imaging for drug development]." Brain Nerve 59(3): 209-214. | Title |
| 2075 | Watkins, L., et al. (2019). "New anti-seizure medication for elderly epileptic patients." Expert Opin Pharmacother 20(13): 1601-1608. | Title |
| 2076 | Weber, J., et al. (2008). "Aripiprazole: in major depressive disorder." CNS Drugs 22(10): 807-813. | Title |
| 2077 | Weber, W. W. (2001). "The legacy of pharmacogenetics and potential applications." Mutation Research/Fundamental and Molecular Mechanisms of Mutagenesis 479(1): 1-18. | Title |
| 2078 | Weinblatt, M. E., et al. (2011). "Effects of the oral syk inhibitor, fostamatinib (r788), on health-related quality of life in a phase II study of active rheumatoid arthritis." Arthritis Rheum 63(10). | Title |
| 2079 | Welsch, P., et al. (2018). "Mirtazapine for fibromyalgia in adults." Cochrane Database Syst Rev 8(8): Cd012708. | Title |
| 2080 | Wenker, S. A., et al. (2024). "Defining the pharmacokinetic/pharmacodynamic index of piperacillin/tazobactam within a hollow-fibre infection model to determine target attainment in intensive care patients." 6(2): dlae036. | Title |
| 2081 | Wensel, T. M., et al. (2014). "Design, implementation, and assessment of an Integrated Pharmacy Applications course series." Currents in Pharmacy Teaching and Learning 6(5): 706-715. | Title |

| | | |
|---|---|---|
| 2082 | Wesley, S. J., et al. (2019). "Multicentre, double-blind, randomised sham-controlled trial: 10khz high-frequency SCS for chronic neuropathic low back pain (modulate-LBP)." Neuromodulation 22(3): E279-E280. | Title |
| 2083 | Westenberg, H. G., et al. (2004). "A double-blind placebo-controlled study of controlled release fluvoxamine for the treatment of generalized social anxiety disorder." J Clin Psychopharmacol 24(1): 49-55. | Title |
| 2084 | Westhovens, R., et al. (2011). "Disease remission, normalized physical function and radiographic non-progression are achieved by the majority of patients with early rheumatoid arthritis treated with abatacept + methotrexate: results from the 2-year agree trial." Rheumatology 50: iii122. | Title |
| 2085 | Westhovens, R., et al. (2013). "Efficacy of abatacept in patients with early (≤6 months) RA: results from agree post-hoc analysis." Annals of the rheumatic disease 71. | Title |
| 2086 | Whibley, D., et al. (2015). "Predictors of poor functional outcome of an episode of distal upper limb pain: the arm pain trial." European journal of epidemiology 30(8): 926. | Title |
| 2087 | Whitener, R., et al. (2022). "Localization of Multi-Lamellar Vesicle Nanoparticles to Injured Brain Tissue in a Controlled Cortical Impact Injury Model of Traumatic Brain Injury in Rodents." Neurotrauma Rep 3(1): 158-167. | Title |
| 2088 | Whitley, R. J. J. H. T. i. I. and I. i. Children (2004). "Congenital cytomegalovirus infection: epidemiology and treatment." 155-160. | Title |
| 2089 | Wier, H. A., et al. (2011). "Rufinamide for pediatric patients with Lennox-Gastaut syndrome: a comprehensive overview." Paediatr Drugs 13(2): 97-106. | Title |
| 2090 | Wiese, M. D., et al. (2020). "Investigational IRAK-4 inhibitors for the treatment of rheumatoid arthritis." Expert Opin Investig Drugs 29(5): 475-482. | Title |
| 2091 | Willcox, M., et al. (2004). Traditional medicinal plants and malaria, CRC press. | Title |
| 2092 | Willekens-Bogaers, M. A. and G. W. Smits (1990). "[Neuroleptics in psychogeriatrics. Evaluation of a treatment proposal]." Tijdschr Gerontol Geriatr 21(3): 99-107. | Title |
| 2093 | William, C., et al. (2016). "A controlled trial of vertebroplasty for acute painful osteoporotic fracture (vapour trial)." Internal medicine journal 46: 20-21. | Title |
| 2094 | Wilson, J. T., et al. (1994). "Paediatric labelling requirements: implications for pharmacokinetic studies." 26: 308-325. | Title |

| | | |
|---|---|---|
| 2095 | Wirth, Y. and B. Rive (2012). "Memantine enhances autonomy in moderate to severe Alzheimer's disease patients already receiving donepezil." Eur J Neurol 19: 474. | Title |
| 2096 | Wolf, D., et al. (2013). "Striatal activation induced by mglur2 positive allosteric modulation correlates with negative symptom reduction in schizophrenia." Neuropsychopharmacology 38: S141-S142. | Title |
| 2097 | Wong, J. B. and C. Wang (2012). "Cost-effectiveness of Tai CHI mind-body exercise for the treatment of fibromyalgia." Arthritis Rheum 64: S1121. | Title |
| 2098 | Woods, E. and A. Federman (2015). "The impact of asthma control on disability in older adults." Journal of the American Geriatrics Society 63: S147-S148. | Title |
| 2099 | Woolfenden, S., et al. (2016). "Who is our cohort: recruitment, representativeness, baseline risk and retention in the "Watch Me Grow" study?" BMC pediatrics 16(1): 46. | Title |
| 2100 | Woon, T. H., et al. (2024). "Evidence of the interactions between immunosuppressive drugs used in autoimmune rheumatic diseases and Chinese herbal medicine: A scoping review." Complementary Therapies in Medicine 80: 103017. | Title |
| 2101 | Wu, D., et al. (2024). "Utilizing nanotechnology and advanced machine learning for early detection of gastric cancer surgery." Environmental Research 245: 117784. | Title |
| 2102 | Wu, R., et al. (2024). "Fixed parameters in the population pharmacokinetic modeling of valproic acid might not be suitable: external validation in Chinese adults with epilepsy or after neurosurgery."  80(11): 1819-1828. | Title |
| 2103 | Wu, X.-L., et al. (2022). "Polymyxin B-associated nephrotoxicity and its predictors: a retrospective study in carbapenem-resistant gram-negative bacterial infections."  13: 672543. | Title |
| 2104 | Wu, Y., et al. (2024). "Insights into the anticancer effects of galangal and galangin: A comprehensive review." Phytomedicine 135: 156085. | Title |
| 2105 | Xiang, Y., et al. (2006). "Training patients with schizophrenia with the community re-entry module: a controlled study." Social psychiatry and psychiatric epidemiology 41(6): 464-469. | Title |
| 2106 | Xie, J., et al. (2017). "Population pharmacokinetics of tigecycline in critically ill patients with severe infections." Antimicrob Agents Chemother 61(8): 10.1128/aac. 00345-00317. | Title |
| 2107 | Xin, Y., et al. (2016). "The efficacy and safety of sodium hyaluronate injection (Adant®) in treating degenerative osteoarthritis: a multi-center, randomized, double-blind, positive-drug parallel-controlled and non-inferiority clinical study." Int J Rheum Dis 19(3): 271-278. | Title |

| | | |
|---|---|---|
| 2108 | Xu, Y., et al. (2012). "In vitro activity of phenylmercuric acetate against ocular pathogenic fungi." 67(8): 1941-1944. | Title |
| 2109 | Yadalam, P. K., et al. (2022). "Assessing the therapeutic potential of agomelatine, ramelteon, and melatonin against SARS-CoV-2." Saudi Journal of Biological Sciences 29(5): 3140-3150. | Title |
| 2110 | Yaldizli, O., et al. (2015). "Brain and cervical spinal cord atrophy in primary progressive multiple sclerosis: results from a placebo-controlled phase III trial (INFORMS)." Multiple sclerosis (Houndmills, Basingstoke, England) 23(11): 30-31. | Title |
| 2111 | Yamada, W., et al. (2022). "Combination therapy to kill Mycobacterium tuberculosis in its nonreplicating persister phenotype." 66(10): e00695-00622. | Title |
| 2112 | Yan, L., et al. (2020). "Pharmacodynamic modeling and exposure-response assessment of inebilizumab in subjects with neuromyelitis optica spectrum disorders." Multiple sclerosis journal 26(3 SUPPL): 94-95. | Title |
| 2113 | Yan, L., et al. (2021). "Pharmacodynamic modeling and exposure response assessment of inebilizumab in subjects with neuromyelitis optica spectrum disorders." Neurology 96(15 SUPPL 1). | Title |
| 2114 | Yan, L. L., et al. (2016). "A randomized controlled trial on rehabilitation through caregiver-delivered nurse-organized service programs for disabled stroke patients in rural china (the RECOVER trial): design and rationale." International journal of stroke 11(7): 823-830. | Title |
| 2115 | Yang, D., et al. (2024). "Stimuli-sensitive biomimetic nanoparticles for the inhibition of breast cancer recurrence and pulmonary metastasis." International Journal of Pharmaceutics: X 7: 100252. | Title |
| 2116 | Yang, J., et al. (2019). "The mediating effect of coping styles and self-efficacy between perceived stress and satisfaction with QOL in Chinese adolescents with type 1 diabetes." Journal of advanced nursing (john wiley & sons, inc.) 75(7): 1439-1449. | Title |
| 2117 | Yang, L., et al. (2020). "Elevated blood pressure in childhood or adolescence and cardiovascular outcomes in adulthood: a systematic review." 75(4): 948-955. | Title |
| 2118 | Yang, L. P. and L. J. Scott (2012). "Clobazam : in patients with Lennox-Gastaut syndrome." CNS Drugs 26(11): 983-991. | Title |
| 2119 | Yao, J. C., et al. (2016). "Everolimus for the treatment of advanced, non-functional neuroendocrine tumours of the lung or gastrointestinal tract (RADIANT-4): a randomised, placebo-controlled, phase 3 study." The Lancet 387(10022): 968-977. | Title |
| 2120 | Yassin, M. T., et al. (2020). "Anticandidal and anti-carcinogenic activities of Mentha longifolia (Wild Mint) extracts in vitro." Journal of King Saud University - Science 32(3): 2046-2052. | Title |

| | | |
|---|---|---|
| 2121 | Young, C. S., et al. (1994). "Neuroleptic medication for dystonia. Reciprocal relationship between effects on motor function and mood." Br J Psychiatry 165(3): 384-386. | Title |
| 2122 | Young, D., et al. (2019). "Effects of Physical Activity Intervention for Chinese People With Severe Mental Illness." Research on social work practice 29(7): 796-807. | Title |
| 2123 | Young, W. B., et al. (2017). "Effects of onabotulinumtoxinatreatment on disability and quality of life in patients with chronic migraine with baseline allodynia: a COMPEL subanalysis." Headache 57: 170-171. | Title |
| 2124 | Yousef, A.-M. F. "Curriculum Vitae AL-Motassem Fahmi Yousef, PhD." | Title |
| 2125 | Yu, H., et al. (2016). "Rapid molecular diagnostics of severe primary immunodeficiency determined by using targeted next-generation sequencing." Journal of Allergy and Clinical Immunology 138(4): 1142-1151.e1142. | Title |
| 2126 | Yu, J., et al. (2008). "Bioequivalence and comparison of pharmacokinetic properties of 4-mg tablet formulations of rosiglitazone hydrochloride and rosiglitazone maleate: A single-dose, randomized, open-label, two-period crossover study in healthy adult male Chinese volunteers." Clin Ther 30(12): 2272-2279. | Title |
| 2127 | Yu, M., et al. (2016). "Nanotechnology for protein delivery: Overview and perspectives." Journal of Controlled Release 240: 24-37. | Title |
| 2128 | Yu, X., et al. (2024). "Association between polypharmacy and cognitive impairment in older adults: A systematic review and meta-analysis." Geriatric Nursing 59: 330-337. | Title |
| 2129 | Yue, Y., et al. (2020). "Cross-sectional study of drug utilisation in a Chinese neonatal unit."  48(5): 0300060520914197. | Title |
| 2130 | Yusuf, M., et al. (2021). "Brain targeted Polysorbate-80 coated PLGA thymoquinone nanoparticles for the treatment of Alzheimer's disease, with biomechanistic insights." Journal of Drug Delivery Science and Technology 61: 102214. | Title |
| 2131 | Zacks, S. and M. W. Fried (2000). 17 Hepatitis C and renal disease. Biomedical Research Reports. T. J. Liang and J. H. Hoofnagle, Academic Press. 2: 329-349. | Title |
| 2132 | Zacks, S. L. and M. W. Fried (2001). "HEPATITIS B AND C AND RENAL FAILURE." Infectious Disease Clinics of North America 15(3): 877-899. | Title |
| 2133 | Zafar, S., et al. (2020). "Co-encapsulation of docetaxel and thymoquinone in mPEG-DSPE-vitamin E TPGS-lipid nanocapsules for breast cancer therapy: Formulation optimization and implications on cellular and in vivo toxicity." European Journal of Pharmaceutics and Biopharmaceutics 148: 10-26. | Title |

| | | |
|---|---|---|
| 2134 | Zaghloul, I. Y., et al. (2007). "The effect of chronic cadmium exposure on the pharmacokinetics of theophylline and ciprofloxacin in rats." Journal of Trace Elements in Medicine and Biology 21(2): 132-137. | Title |
| 2135 | Zaib, S., et al. (2024). "Fabrication and evaluation of anticancer potential of diosgenin incorporated chitosan-silver nanoparticles; in vitro, in silico and in vivo studies." International Journal of Biological Macromolecules 254: 127975. | Title |
| 2136 | Zaidi, S. T. R. and J. A. Roberts (2016). Drug dosing in obesity, Springer. | Title |
| 2137 | Zaina, F., et al. (2010). "Clinical and kinematic evaluation of osteopathy vs specific exercises in obese non-specific chronic low back pain females patients: a randomized controlled trial." Spine (Phila Pa 1976). | Title |
| 2138 | Zainab, A., et al. (2017). "Invasive Candidiasis in pediatric patients at King Fahad Medical City in Central Saudi Arabia." 38(11): 1118-1124. | Title |
| 2139 | Zaki, N. M. and A. A. Albarraq (2014). "Use, attitudes and knowledge of medications among pregnant women: A Saudi study." Saudi Pharmaceutical Journal 22(5): 419-428. | Title |
| 2140 | Zaki, R. M., et al. (2022). "Formulation and evaluation of transdermal gel containing tacrolimus-loaded spanlastics: in vitro, ex vivo and in vivo studies." 14(8): 1528. | Title |
| 2141 | Zakraoui, M., et al. (2024). "Ifanosine: Olea europaea L. and Hyphaene thebaica L. combination, from traditional utilization to rational formulation: Preclinical and clinical efficacy on hypertensives patients." Journal of Ethnopharmacology 325: 117834. | Title |
| 2142 | Zamir, A., et al. (2022). "Clinical Pharmacokinetics of Metoprolol: A Systematic Review." Clin Pharmacokinet 61(8): 1095-1114. | Title |
| 2143 | Zamir, A., et al. (2023). "Physiologically Based Pharmacokinetic Model To Predict Metoprolol Disposition in Healthy and Disease Populations." ACS Omega 8(32): 29302-29313. | Title |
| 2144 | Zang, Y., et al. (2019). "Global burden of late-stage chronic kidney disease resulting from dietary exposure to cadmium, 2015." Environmental Research 169: 72-78. | Title |
| 2145 | Zang, Y.-N., et al. (2022). "Published population pharmacokinetic models of valproic acid in adult patients: A systematic review and external validation in a Chinese sample of inpatients with bipolar disorder." 15(5): 621-635. | Title |
| 2146 | Zanini, B., et al. (2013). "Search for atoxic cereals: a single blind, cross-over study on the safety of a single dose of Triticum monococcum, in patients with celiac disease." BMC Gastroenterol 13: 92. | Title |
| 2147 | Zareei, S., et al. (2025). "Phenyldiazenyl-phenoxy-1,2,3-triazol-acetamide derivatives as new dual cholinesterase Inhibitors: Design, synthesis, in vitro, and in silico enzymatic inhibition evaluations." Journal of Molecular Structure 1321: 139686. | Title |

| | | |
|---|---|---|
| 2148 | Zargar, S., et al. (2024). "Elucidation of molecular mechanisms, pathways, and diseases modulated by arsenicals through toxogenomics and multi-omics analysis." Journal of Trace Elements in Medicine and Biology 86: 127561. | Title |
| 2149 | Závada, J., et al. (2016). "A tailored approach to reduce dose of anti-TNF drugs may be equally effective, but substantially less costly than standard dosing in patients with ankylosing spondylitis over 1 year: a propensity score-matched cohort study." Ann Rheum Dis 75(1): 96-102. | Title |
| 2150 | Zecca, C., et al. (2012). "Natalizumab de-escalation to interferon beta-1b in multiple sclerosis patients." Clinical neurophysiology 123(10): e111-e112. | Title |
| 2151 | Zeidan, A. M., et al. (2017). "Hypomethylating agent (HMA) therapy use and survival in older patients with higher risk myelodysplastic syndromes (HR-MDS) in the United States (USA): a large population-based study." Journal of clinical oncology 35(15). | Title |
| 2152 | Zelenitsky, S., et al. (2013). "Vancomycin pharmacodynamics and survival in patients with methicillin-resistant Staphylococcus aureus-associated septic shock." International Journal of Antimicrobial Agents 41(3): 255-260. | Title |
| 2153 | Zerbini, C., et al. (2015). "Efficacy and safety of tofacitinib monotherapy versus combination therapy in a latin american subpopulation of patients with rheumatoid arthritis: a pooled phase 3 analysis." Arthritis & rheumatology 67(no pagination). | Title |
| 2154 | Zeuzem, S. J. A. o. i. m. (2004). "Heterogeneous virologic response rates to interferon-based therapy in patients with chronic hepatitis C: who responds less well?" 140(5): 370-381. | Title |
| 2155 | Zgierska, A. E., et al. (2016). "Mindfulness Meditation-Based Intervention Is Feasible, Acceptable, and Safe for Chronic Low Back Pain Requiring Long-Term Daily Opioid Therapy." Journal of alternative and complementary medicine (New York, N.Y.) 22(8): 610-620. | Title |
| 2156 | Zhang, F., et al. (2015). "Long-term impact of apremilast on physical function in patients with psoriatic arthritis using the Haq-Di assessment." Ann Rheum Dis 74: 1168. | Title |
| 2157 | Zhang, F., et al. (2014). "Impact of apremilast on physical function in patients with psoriatic arthritis." Value in health 17(3): A41. | Title |
| 2158 | Zhang, H., et al. (2021). "Assessing the Efficacy and Safety of Tirofiban in Combination with Dual-Antiplatelet Therapy in Progressive Ischemic Stroke Patients." Journal of cardiovascular pharmacology 78(3): 448-452. | Title |
| 2159 | Zhang, M., et al. (1994). "Community-based psychiatric rehabilitation in Shanghai. Facilities, services, outcome, and culture-specific characteristics." British journal of psychiatry. Supplement(24): 70-79. | Title |

| | | |
|---|---|---|
| 2160 | Zhang, N., et al. (2023). "The impact of the COVID-19 pandemic on people with epilepsy and epilepsy specialists." Epilepsy & Behavior 147: 109389. | Title |
| 2161 | Zhang, Q., et al. (2023). "Biopharmaceutical, preclinical pharmacokinetic and pharmaco-dynamic investigations of an orally administered novel 3-nbutylphthalide prodrug for ischemic stroke treatment." Eur J Pharm Sci 180: 106308. | Title |
| 2162 | Zhang, T., et al. (2024). "Therapeutic Drug Monitoring of Vancomycin in Pediatric Patients: Defining a Therapeutic Drug Window." | Title |
| 2163 | Zhang, W., et al. (2020). "Oral drugs used to treat persistent pulmonary hypertension of the newborn." 13(12): 1295-1308. | Title |
| 2164 | Zhang, X., et al. (2019). "Evaluation of a WeChat-based life review programme for cancer patients: a quasi-experimental study." Journal of advanced nursing (john wiley & sons, inc.) 75(7): 1563-1574. | Title |
| 2165 | Zhang, Y., et al. (2023). "Frontiers and Hotspots Evolution of Drug-Drug Interaction Prediction Models: A Bibliometric Study from 2013 to 2022." 46(11): 1195-1196. | Title |
| 2166 | Zhao, C., et al. (2024). "Quantifying combined effects of colistin and ciprofloxacin against Escherichia coli in an in silico pharmacokinetic-pharmacodynamic model." 14(1): 11706. | Title |
| 2167 | Zhou, M., et al. (2023). "Edited by: Yuetian Yu, Shanghai Jiao Tong University, China Reviewed by." 101. | Title |
| 2168 | Zhou, Y., et al. (2022). "External validation of vancomycin population pharmacokinetic models in ten cohorts of infected Chinese patients." Journal of Global Antimicrobial Resistance 30: 163-172. | Title |
| 2169 | Zhu, B., et al. (2019). "Phase 2 affinity trial evaluates opicinumab in a targeted population of patients with relapsing multiple sclerosis: rationale, design and baseline characteristics." Neurology 92(15). | Title |
| 2170 | Zhu, B., et al. (2018). "Phase 2 AFFINITY trial evaluates opicinumab in a targeted population of patients with relapsing multiple sclerosis: rationale, design and baseline characteristics." Multiple sclerosis journal 24(2): 914-915. | Title |
| 2171 | Zhu, H. and K. Kannan (2019). "Inter-day and inter-individual variability in urinary concentrations of melamine and cyanuric acid." Environment International 123: 375-381. | Title |
| 2172 | Zigler, J., et al. (2011). "Five-year follow-up of the prodisc-l versus fusion ide patient cohorts." Spine journal 11(10): 109S. | Title |
| 2173 | Zikri, A. and K. El Masri (2019). "Use of Ceftolozane/tazobactam for the Treatment of Multidrug-resistant Pseudomonas aeruginosa Pneumonia in a Pediatric Patient with Combined Immunodeficiency (CID): A Case Report from a Tertiary Hospital in Saudi Arabia." Antibiotics (Basel) 8(2). | Title |

| | | |
|---|---|---|
| 2174 | Zilahi, G., et al. (2016). "What's new in multidrug-resistant pathogens in the ICU?"  6: 1-11. | Title |
| 2175 | Zilov, A., et al. (2013). "Insulin detemir in the management of type 2 diabetes in non-Western countries: Safety and effectiveness data from the A1chieve observational study." Diabetes Research and Clinical Practice 101(3): 317-325. | Title |
| 2176 | Zowawi, H. M., et al. (2013). "β-Lactamase production in key gram-negative pathogen isolates from the Arabian Peninsula."  26(3): 361-380. | Title |
| 2177 | Zulfiqar, B., et al. (2022). "Immunotherapy and targeted therapy for lung cancer: Current status and future perspectives."  13: 1035171. | Title |

**Supplementary Table S2: Quality assessment of included articles based on JADAD Scoring**

| | JADAD Questions | | | | | | |
|---|---|---|---|---|---|---|---|
| **Sr #** | **Reference** | **Was the study described as a randomized** | **Was the method used to generate the sequence of randomization described and appropriate** | **Was the study described as double-blind** | **Was the method of double blinding described and appropriate** | **Was there a description of withdrawals and dropouts** | **JADAD score** |
| 1- | Alsultan et. al (2020) [1] | 0 | 0 | 0 | 0 | 0 | 0 |
| 2- | Alqahtani et. al (2019) [2] | 0 | 0 | 0 | 0 | 0 | 0 |
| 3- | Islam et. al (2013) [3] | 0 | 0 | 0 | 0 | 0 | 0 |
| 4- | El-Yazigi et. al (1990) [4] | 0 | 0 | 0 | 0 | 0 | 0 |
| 5- | Alqahtani et. al (2018) [5] | 0 | 0 | 0 | 0 | 0 | 0 |
| 6- | Alqahtani et. al (2018) [6] | 0 | 0 | 0 | 0 | 0 | 0 |
| 7- | Alqahtani et. al (2021) [7] | 0 | 0 | 0 | 0 | 0 | 0 |
| 8- | Alqahtani et. al (2020) [8] | 0 | 0 | 0 | 0 | 0 | 0 |

| | | | | | | | |
|---|---|---|---|---|---|---|---|
| 9- | Alqahtani et. al (2021) [9] | 0 | 0 | 0 | 0 | 0 | 0 |

0= No, 1= Yes

**Supplementary Table S3: Quality assessment of included articles based on the Critical Appraisal Skill Program (CASP)**

| CASP Questions | | | | | | | | | | | | |
|---|---|---|---|---|---|---|---|---|---|---|---|---|
| **Sr #** | **Reference** | **1** | **2** | **3** | **4** | **5** | **6** | **7** | **8** | **9** | **10** | **CASP Score** |
| 1- | Alsultan et. al (2020) [1] | Y | Y | Y | Y | Y | Y | Y | Y | Y | Y | 10 |
| 2- | Alqahtani et. al (2019) [2] | Y | Y | Y | Y | Y | Y | Y | Y | Y | Y | 10 |
| 3- | Islam et. al (2013) [3] | Y | Y | Y | CT | Y | Y | Y | N | Y | Y | 8 |
| 4- | El-Yazigi et. al (1990) [4] | Y | Y | Y | CT | Y | Y | Y | N | Y | Y | 8 |
| 5- | Alqahtani et. al (2018) [5] | Y | Y | Y | CT | CT | Y | Y | N | Y | Y | 7 |
| 6- | Alqahtani et. al (2018) [6] | Y | Y | Y | Y | Y | Y | Y | N | Y | Y | 9 |
| 7- | Alqahtani et. al (2021) [7] | Y | Y | Y | Y | Y | Y | Y | N | Y | Y | 9 |
| 8- | Alqahtani et. al (2020) [8] | Y | Y | Y | CT | Y | Y | Y | N | Y | Y | 8 |
| 9- | Alqahtani et. al (2021) [9] | Y | Y | Y | Y | Y | Y | Y | Y | Y | Y | 10 |

Y= Yes, N= No, CT= can't tell

Questions:

1: Was there a clear statement of the aims of the research?

2: Is a qualitative methodology appropriate?

3: Was the research design appropriate to address the aims of the research?

4: Are the study's theoretical underpinnings clear, consistent, and conceptually coherent?

5: Was the recruitment strategy appropriate to the aims of the search?

6: Was the data collected in a way that addressed the research issue?

7: Has the relationship between researchers and participants been adequately considered?

8: Have ethical issues been taken into consideration?

9: Was the data analysis sufficiently rigorous?

10: Is there a clear statement of findings?

**Supplementary Table S4: Quality assessment of included articles based on Critical Appraisal Clinical Pharmacokinetic (CACPK) tool**

| **Sr #** | **Delphi Questionnaire** | | | | | | | | | |
|---|---|---|---|---|---|---|---|---|---|---|
| | | Alsultan et. al (2020) [1] | Alqahtani et. al (2019) [2] | Islam et. al (2013) [3] | El-Yazigi et. al (1990) [4] | Alqahtani et. al (2018) [5] | Alqahtani et. al (2018) [6] | Alqahtani et. al (2021) [7] | Alqahtani et. al (2020) [8] | Alqahtani et. al (2021) [9] |
| | **Background** | | | | | | | | | |
| 1 | Was a clear description of | Y | Y | Y | Y | Y | Y | Y | Y | Y |

| | | | | | | | | | | |
|---|---|---|---|---|---|---|---|---|---|---|
| | the objectives of the study provided? | | | | | | | | | |
| 2 | Was a valid and comprehensive rationale provided to support the purpose of the study? | Y | Y | Y | Y | Y | Y | Y | Y | Y |
| | **Design** | | | | | | | | | |
| 3 | Was the chosen study design appropriately selected and justified? | Y | Y | Y | Y | Y | Y | Y | Y | Y |
| 4 | Was the dosing (dose, route of administration, dosing interval) of the drug in the study justified for the intended study? | Y | Y | Y | Y | Y | Y | N | Y | Y |

| | | | | | | | | | | |
|---|---|---|---|---|---|---|---|---|---|---|
| 5 | Were the endpoints of the study appropriate to answer the objectives of the study? | Y | Y | Y | Y | Y | Y | Y | Y | Y |
| 6 | Were the exclusion criteria of participants included AND appropriate for the intended outcomes of the study? | N | N | Y | N | Y | N | N | N | Y |
| 7 | Where applicable, were the relevant baseline characteristics of the participants adequately described? | Y | Y | Y | Y | Y | Y | Y | Y | Y |

| | | | | | | | | | | |
|---|---|---|---|---|---|---|---|---|---|---|
| 8 | Were plausible interacting covariates described a priori or in post hoc evaluation? | Y | Y | N | Y | N | Y | Y | N | N |
| 9 | Was the description of the used sample analysis methods or citations of prior validation studies provided in the publication or affiliated appendix | Y | Y | Y | Y | Y | Y | Y | Y | Y |
| | **Sampling** | | | | | | | | | |
| 10 | Was the method of data sampling appropriate for the study? | Y | Y | Y | Y | Y | Y | IDK | Y | Y |

| | | | | | | | | | | |
|---|---|---|---|---|---|---|---|---|---|---|
| 11 | Was a clear description of the sampling site and the sampling interval (the exact times at which samples are obtained) provided and justified | Y | N | N | Y | Y | Y | N | Y | Y |
| 12 | Was the number of half-lives elapsed within the sampling period appropriate for the analyzed drug? | Y | Y | IDK | Y | Y | Y | IDK | Y | Y |
| 13 | Were sample storage conditions appropriate and described in a manner that | Y | N | N | Y | Y | Y | IDK | N | Y |

| | | | | | | | | | | |
|---|---|---|---|---|---|---|---|---|---|---|
| | could be accurately replicated? | | | | | | | | | |
| 14 | If applicable, was there a clear description of the pharmacokinetic model, its development, validation and justification for use? | Y | Y | N | N | Y | Y | Y | Y | Y |
| 15 | Was the described population pharmacokinetic approach validation method appropriate for the analysis? | Y | Y | IDK | IDK | Y | Y | Y | Y | Y |

| | | | | | | | | | | |
|---|---|---|---|---|---|---|---|---|---|---|
| 16 | Were the essential pharmacokinetic parameters required to make the results applicable in clinical settings addressed? | Y | Y | Y | Y | Y | Y | Y | Y | Y |
| 17 | Were the pharmacokinetic equations used to calculate patient pharmacokinetic parameters disclosed or cited within the article? | Y | Y | N | N | N | N | N | Y | N |
| | **Applied Statistics** | | | | | | | | | |

| | | | | | | | | | | |
|---|---|---|---|---|---|---|---|---|---|---|
| 18 | Were the chosen statistical tests and software to perform the statistical analysis appropriate to achieve the study objectives? | Y | Y | Y | Y | Y | Y | Y | Y | Y |
| | **Results** | | | | | | | | | |
| 19 | Were all patients enrolled in the study accounted for? | Y | Y | Y | Y | Y | Y | Y | Y | Y |
| 20 | In the event of missing data or outliers, was the process for analysis justified and appropriate? | Y | Y | Y | Y | Y | Y | Y | Y | Y |

| | | | | | | | | | | |
|---|---|---|---|---|---|---|---|---|---|---|
| 21 | Were appropriate summary statistics to describe centrality and variance used to document the pharmacokinetic results? | Y | Y | Y | Y | Y | Y | Y | Y | Y |
| | **DELPHI Score** | 20 | 18 | 14 | 17 | 19 | 19 | 14 | 18 | 19 |

Y: Yes, N: No, IDK: I don't know

**Supplementary Table S5: Risk of bias assessment of included articles based on the Cochrane Collaboration tool (CCT)**

| Sr# | Reference | Random sequence Generation (selection bias) | Allocation concealment (selection bias) | Blinding of participants and researchers (performed bias) | Blinding of outcome assessment (detection bias) | Incomplete outcome data (attrition bias) | Selective reporting (reporting bias) | Other bias | Bias Score |
|---|---|---|---|---|---|---|---|---|---|
| 1- | Alsultan et. al (2020) [1] | LR | LR | LR | LR | UR | LR | UR | 5 |
| 2- | Alqahtani et. al (2019) [2] | LR | LR | UR | LR | LR | LR | LR | 6 |
| 3- | Islam et. al (2013) [3] | LR | LR | UR | LR | LR | LR | UR | 5 |
| 4- | El-Yazigi et. al (1990) [4] | LR | LR | LR | LR | LR | LR | UR | 6 |
| 5- | Alqahtani et. al (2018) [5] | LR | LR | UR | LR | LR | LR | LR | 6 |
| 6- | Alqahtani et. al (2018) [6] | LR | LR | LR | LR | UR | LR | LR | 6 |
| 7- | Alqahtani et. al (2021) [7] | LR | LR | LR | LR | UR | LR | UR | 6 |
| 8- | Alqahtani et. al (2020) [8] | LR | LR | UR | LR | LR | LR | LR | 6 |
| 10- | Alqahtani et. al (2021) [9] | LR | LR | LR | LR | UR | LR | LR | 6 |

LR: Low risk. UR: Un-reported risk

**Supplementary Table S6: Revised version of Risk of Bias Assessment for Nonrandomized Studies (RoBANS 2) Tool**

| Sr# | Reference | Comparability of target group | Target group selection | Confounders | Measurement of intervention/ exposure | Blinding of assessors | Blinding of outcome assessments | Incomplete outcome data | Selective outcome reporting | Total Risk of Bias |
|---|---|---|---|---|---|---|---|---|---|---|
| 1 | Alsultan et. al (2020) [1] | LR | LR | UR | LR | LR | LR | LR | UR | 6 |
| 2 | Alqahtani et. al (2019) [2] | LR | LR | LR | UR | UR | LR | LR | UR | 5 |
| 3 | Islam et. al (2013) [3] | LR | LR | UR | LR | UR | LR | LR | LR | 6 |
| 4 | El-Yazigi et. al (1990) [4] | LR | LR | LR | UR | LR | LR | LR | LR | 7 |
| 5 | Alqahtani et. al (2018) [5] | LR | LR | UR | LR | UR | LR | LR | LR | 6 |
| 6 | Alqahtani et. al (2018) [6] | LR | LR | UR | LR | LR | LR | LR | LR | 7 |
| 7 | Alqahtani et. al (2021) [7] | LR | LR | UR | LR | LR | LR | LR | UR | 6 |
| 8 | Alqahtani et. al (2020) [8] | LR | LR | UR | LR | UR | LR | LR | UR | 5 |
| 10 | Alqahtani et. al (2021) [9] | LR | LR | LR | LR | LR | LR | LR | LR | 8 |

LR: Low risk. UR: Un-reported risk

## PRISMA 2020 CHECKLIST

| Section and Topic | Item # | Checklist item | Location where item is reported |
|---|---|---|---|
| **TITLE** | | | |
| Title | 1 | Identify the report as a systematic review. | 1 |
| **ABSTRACT** | | | |
| Abstract | 2 | See the PRISMA 2020 for Abstracts checklist. | 1 |
| **INTRODUCTION** | | | |
| Rationale | 3 | Describe the rationale for the review in the context of existing knowledge. | 2 |
| Objectives | 4 | Provide an explicit statement of the objective(s) or question(s) the review addresses. | 2 |
| **METHODS** | | | |
| Eligibility criteria | 5 | Specify the inclusion and exclusion criteria for the review and how studies were grouped for the syntheses. | 8 |
| Information sources | 6 | Specify all databases, registers, websites, organisations, reference lists, and other sources searched or consulted to identify studies. Specify the date when each source was last searched or consulted. | 8 |
| Search strategy | 7 | Present the full search strategies for all databases, registers and websites, including any filters and limits used. | 8 |
| Selection process | 8 | Specify the methods used to decide whether a study met the inclusion criteria of the review, including how many reviewers screened each record and each report retrieved, whether they worked independently, and if applicable, details of automation tools used in the process. | 8 |
| Data collection process | 9 | Specify the methods used to collect data from reports, including how many reviewers collected data from each report, whether they worked independently, any processes for obtaining or confirming data from study investigators, and if applicable, details of automation tools used in the process. | 8 |
| Data items | 10a | List and define all outcomes for which data were sought. Specify whether all results that were compatible with each outcome domain in each study were sought (e.g. for all | 9 |

| Section and Topic | Item # | Checklist item | Location where item is reported |
|---|---|---|---|
| | | measures, time points, analyses), and if not, the methods used to decide which results to collect. | |
| | 10b | List and define all other variables for which data were sought (e.g. participant and intervention characteristics, funding sources). Describe any assumptions made about any missing or unclear information. | 9 |
| Study risk of bias assessment | 11 | Specify the methods used to assess risk of bias in the included studies, including details of the tool(s) used, how many reviewers assessed each study and whether they worked independently, and if applicable, details of automation tools used in the process. | 9 |
| Effect measures | 12 | Specify for each outcome the effect measure(s) (e.g. risk ratio, mean difference) used in the synthesis or presentation of results. | 8,9 |
| Synthesis methods | 13a | Describe the processes used to decide which studies were eligible for each synthesis (e.g. tabulating the study intervention characteristics and comparing against the planned groups for each synthesis (item #5)). | 8,9 |
| | 13b | Describe any methods required to prepare the data for presentation or synthesis, such as handling of missing summary statistics, or data conversions. | 8,9 |
| | 13c | Describe any methods used to tabulate or visually display results of individual studies and syntheses. | 8,9 |
| | 13d | Describe any methods used to synthesize results and provide a rationale for the choice(s). If meta-analysis was performed, describe the model(s), method(s) to identify the presence and extent of statistical heterogeneity, and software package(s) used. | 8,9 |
| | 13e | Describe any methods used to explore possible causes of heterogeneity among study results (e.g. subgroup analysis, meta-regression). | N/A |
| | 13f | Describe any sensitivity analyses conducted to assess robustness of the synthesized results. | N/A |

| Section and Topic | Item # | Checklist item | Location where item is reported |
|---|---|---|---|
| Reporting bias assessment | 14 | Describe any methods used to assess risk of bias due to missing results in a synthesis (arising from reporting biases). | 9 |
| Certainty assessment | 15 | Describe any methods used to assess certainty (or confidence) in the body of evidence for an outcome. | N/A |
| **RESULTS** | | | |
| Study selection | 16a | Describe the results of the search and selection process, from the number of records identified in the search to the number of studies included in the review, ideally using a flow diagram. | 2 |
| | 16b | Cite studies that might appear to meet the inclusion criteria, but which were excluded, and explain why they were excluded. | 3,4 |
| Study characteristics | 17 | Cite each included study and present its characteristics. | 3.4 |
| Risk of bias in studies | 18 | Present assessments of risk of bias for each included study. | 4 |
| Results of individual studies | 19 | For all outcomes, present, for each study: (a) summary statistics for each group (where appropriate) and (b) an effect estimate and its precision (e.g. confidence/credible interval), ideally using structured tables or plots. | 4,5,6 |
| Results of syntheses | 20a | For each synthesis, briefly summarise the characteristics and risk of bias among contributing studies. | 3,4 |
| | 20b | Present results of all statistical syntheses conducted. If meta-analysis was done, present for each the summary estimate and its precision (e.g. confidence/credible interval) and measures of statistical heterogeneity. If comparing groups, describe the direction of the effect. | N/A |
| | 20c | Present results of all investigations of possible causes of heterogeneity among study results. | N/A |
| | 20d | Present results of all sensitivity analyses conducted to assess the robustness of the synthesized results. | N/A |
| Reporting biases | 21 | Present assessments of risk of bias due to missing results | 4 |

| Section and Topic | Item # | Checklist item | Location where item is reported |
|---|---|---|---|
| | | (arising from reporting biases) for each synthesis assessed. | |
| Certainty of evidence | 22 | Present assessments of certainty (or confidence) in the body of evidence for each outcome assessed. | N/A |
| **DISCUSSION** | | | |
| Discussion | 23a | Provide a general interpretation of the results in the context of other evidence. | 6,7 |
| | 23b | Discuss any limitations of the evidence included in the review. | 7 |
| | 23c | Discuss any limitations of the review processes used. | 7 |
| | 23d | Discuss implications of the results for practice, policy, and future research. | 7 |
| **OTHER INFORMATION** | | | |
| Registration and protocol | 24a | Provide registration information for the review, including register name and registration number, or state that the review was not registered. | N/A |
| | 24b | Indicate where the review protocol can be accessed, or state that a protocol was not prepared. | N/A |
| | 24c | Describe and explain any amendments to information provided at registration or in the protocol. | N/A |
| Support | 25 | Describe sources of financial or non-financial support for the review, and the role of the funders or sponsors in the review. | 9 |
| Competing interests | 26 | Declare any competing interests of review authors. | 9 |
| Availability of data, code and other materials | 27 | Report which of the following are publicly available and where they can be found: template data collection forms; data extracted from included studies; data used for all analyses; analytic code; any other materials used in the review. | 9 |

*From:* Page MJ, McKenzie JE, Bossuyt PM, Boutron I, Hoffmann TC, Mulrow CD, et al. The PRISMA 2020 statement: an updated guideline for reporting systematic reviews. BMJ 2021;372:n71. doi: 10.1136/bmj.n71

References


1. Alsultan, A., et al., *Population pharmacokinetics of busulfan in Saudi pediatric patients undergoing hematopoietic stem cell transplantation.* Int J Clin Pharm, 2020. **42**(2): p. 703-712.
2. Alqahtani, S., N. Alandas, and A. Alsultan, *Estimation of apparent clearance of valproic acid in adult Saudi patients.* Int J Clin Pharm, 2019. **41**(4): p. 1056-1061.
3. Islam, S., et al., *Population pharmacokinetics of Carbamazepine and optimising its use in Saudi epileptic children.* International Research Journal of Medicine Medical Sciences, 2013. **1**(4): p. 85-93.
4. el-Yazigi, A., et al., *Steady state pharmacokinetics of propranolol in Saudi Arabian patients and comparison with data for different populations.* J Clin Pharmacol, 1990. **30**(2): p. 144-50.
5. Alqahtani, S.A., et al., *Population Pharmacokinetic Model for Vancomycin Used in Open Heart Surgery: Model-Based Evaluation of Standard Dosing Regimens.* Antimicrob Agents Chemother, 2018. **62**(7).
6. Alqahtani, S.A., et al., *Population Pharmacokinetic Model-Based Evaluation of Standard Dosing Regimens for Cefuroxime Used in Coronary Artery Bypass Graft Surgery with Cardiopulmonary Bypass.* Antimicrob Agents Chemother, 2018. **62**(4).
7. Alqahtani, S., et al., *Estimation of Tacrolimus Clearance in Saudi Adult Kidney Transplant Recipients.* Saudi J Kidney Dis Transpl, 2021. **32**(1): p. 101-110.
8. Alqahtani, S., et al., *Optimization of Vancomycin Dosing Regimen in Cancer Patients using Pharmacokinetic/Pharmacodynamic Modeling.* Pharmacotherapy, 2020. **40**(12): p. 1192-1200.
9. Alqahtani, S., et al., *Assessment of Micafungin Dosage Regimens in Patients with Cancer Using Pharmacokinetic/Pharmacodynamic Modeling and Monte Carlo Simulation.* Antibiotics (Basel), 2021. **10**(11).
